# A mix-and-read drop-based *in vitro* two-hybrid method for screening high-affinity peptide binders

**DOI:** 10.1038/srep22575

**Published:** 2016-03-04

**Authors:** Naiwen Cui, Huidan Zhang, Nils Schneider, Ye Tao, Haruichi Asahara, Zhiyi Sun, Yamei Cai, Stephan A. Koehler, Tom F. A. de Greef, Alireza Abbaspourrad, David A. Weitz, Shaorong Chong

**Affiliations:** 1 School of Engineering and Applied Sciences, Harvard University, Cambridge, MA 02138, USA; 2 Department of Cell Biology, Key Laboratory of Cell Biology, Ministry of Public Health, and Key Laboratory of Medical Cell Biology, Ministry of Education, China Medical University, Shenyang 110001, China; 3 Ecole Supérieure de Biotechnologie de Strasbourg, Boulevard Sébastien Brant, 67400, Illkirch, France; 4 New England Biolabs, Inc. 240 County Road, Ipswich, MA 01938, USA; 5 School of Mechatronics Engineering, Harbin Institute of Technology, Harbin 150001, China; 6 School of Pharmacy, Second Military Medical University, Shanghai 200433, China; 7 Institute for Complex Molecular Systems, Department of Biomedical Engineering Eindhoven University of Technology Den Dolech 2, 5600 MB Eindhoven, The Netherlands; 8 Department of Physics, Harvard University, Cambridge, MA02138, USA

## Abstract

Drop-based microfluidics have recently become a novel tool by providing a stable linkage between phenotype and genotype for high throughput screening. However, use of drop-based microfluidics for screening high-affinity peptide binders has not been demonstrated due to the lack of a sensitive functional assay that can detect single DNA molecules in drops. To address this sensitivity issue, we introduced *in vitro* two-hybrid system (IVT2H) into microfluidic drops and developed a streamlined mix-and-read drop-IVT2H method to screen a random DNA library. Drop-IVT2H was based on the correlation between the binding affinity of two interacting protein domains and transcriptional activation of a fluorescent reporter. A DNA library encoding potential peptide binders was encapsulated with IVT2H such that single DNA molecules were distributed in individual drops. We validated drop-IVT2H by screening a three-random-residue library derived from a high-affinity MDM2 inhibitor PMI. The current drop-IVT2H platform is ideally suited for affinity screening of small-to-medium-sized libraries (10^3^–10^6^). It can obtain hits within a single day while consuming minimal amounts of reagents. Drop-IVT2H simplifies and accelerates the drop-based microfluidics workflow for screening random DNA libraries, and represents a novel alternative method for protein engineering and *in vitro* directed protein evolution.

Protein-protein interactions (PPIs) regulate cellular physiology by influencing interactome networks. Between 40,000 and 200,000 PPIs have been predicted to exist within the human interactome, and their malfunction is one of the fundamental causes of human diseases^1^,^2^. One promising therapeutic strategy involves the use of peptide drugs with high target-specific affinities to regulate certain PPIs^3^. Compared with protein and small-molecule drugs, therapeutic peptides offer the advantages of better cell penetration, less immunogenicity and greater specificity^4^,^5^. More than 60 synthetic therapeutic peptides have recently reached pharmaceutical markets^6^. For example, Degarelix (Firmagon), a gonadotrophin-releasing hormone receptor blocker, has been shown effective for the treatment of men with advanced hormone-sensitive prostate cancer^7^.

Currently, molecular display represents the most widely used high-throughput techniques for screening high-affinity peptide binders. Taking the advantage of living cells’ ability to express a DNA library and display the protein or peptide products on their surfaces, *in vivo* display systems utilize phage, bacterium and yeast to establish a physical link between the binding affinity to a target molecule (phenotype) and the DNA sequence (genotype) of the displayed molecule. However, such *in vivo* systems often suffer from serious drawbacks, such as low transformation efficiency, expression bias, toxicity of fusion proteins and interference of other surface proteins during selection^8^,^9^,^10^. In comparison, cell-free display systems, such as ribosome and mRNA display, contain only the essential elements for protein expression and thus provide an *in vitro* solution to address some of these issues. However, cell-free display systems also have their own drawbacks. For instance, in the ribosome display method, potential protein or peptide binders are expressed from a DNA library and through ribosomes form a linkage with their coding mRNAs^11^,^12^. The binders with high affinities towards a target are selected through “biopanning”, a series of washing and amplification cycles. Such selection conditions can destabilize the mRNA-binder complex. The mRNA display method improves the stability by establishing a covalent linkage between the binder and its coding mRNA. Nevertheless, the RNA-binder complexes are inherently unstable which can severely restricts the screening conditions^13^,^14^. Moreover, the selection of high-affinity binders using biopanning in both ribosome and mRNA display methods can be biased by the dissociation kinetics of the binder to the target molecule. A recently reported bead display method circumvents some of these drawbacks by displaying binder-DNA conjugates on monoclonal beads, which are subsequently screened using flow cytometer^15^,^16^,^17^. It is unclear whether the binding to the target molecule is affected by the attachment of the binder to a large heterogeneous bead surface. Drop-based microfluidics has become a novel tool for high throughput screening in recent years. It provides a stable linkage between phenotype and genotype by partitioning single cells into picoliter drops and allows fluorescence-activated drop sorting (FADS)^18^,^19^. However, the use of such drop-based microfluidic method to screen high-affinity binders from a random DNA library has never been shown, largely due to the fact that there is no functional assay sensitive enough to detect single DNA molecules for protein binding in drops.

Here we combined drop-based microfluidics with our recently developed *in vitro* two-hybrid system (IVT2H) – an *in vitro* assay for detection of protein-protein interaction^20^, and demonstrated a simple and cost-effective screening platform, which we named “drop-IVT2H”. We encapsulated single DNA molecules of a peptide library in picoliter drops with the IVT2H reagents containing plasmids expressing the target protein. Drops were incubated off-chip to allow the expression of both binder and target proteins. The binding of a high-affinity binder to the target protein activated the GFP expression *in situ*, resulting in highly fluorescent drops (bright drops). These bright drops were isolated by the FADS device and the high-affinity binders were subsequently identified by DNA sequencing. We demonstrate that this mix-and-read drop-IVT2H has allowed successful enrichment of high-affinity peptide binders in a p53-MDM2 binding model.

## Materials and Methods

### IVT2H reagents and DNA constructs

IVT2H reagents are described previously^20^,^21^,^22^. Briefly, IVT2H contained 144 nM purified E. coli RNA polymerase core enzyme, 1.2 μM purified recombinant E. coli IHF, 0.8 units/μl murine RNase inhibitor, the PURExpress^®^ *in vitro* protein synthesis system (New England Biolabs), 0.2 ng/μl (45 pM) plasmid DNA expressing σ54, 4.4 nM linear reporter DNA expressing GFP and 0.2 ng/μl (60 pM) plasmid DNA expressing hybrid fusion protein AD-MDM2, in which the activation domain (AD, residues 1–296) of PspF was fused to the full-length human MDM2 protein. The genes for the wild-type p53 peptide (p53p, residues 17–26: ETFSDLWKLLPE) and the peptide inhibitor (PMI: TSFAEYWNLLSP) were synthesized and fused to the N-terminus of the DNA binding domain Cro (DB). The linear DNA constructs expressing p53p-DB, PMI-DB or the PMI library-DB were used in drop-based microfluidics experiments.

### Construction of the full-length PMI Library

The PMI library was constructed by randomizing the hydrophobic triad, FWL of the PMI sequence (underlined: TSFAEYWNLLSP)^23^,^24^. The DNA fragments containing T7 promoter and the randomized PMI sequences were assembled from four overlapping oligonucleotides (fw1, fw2, rv1 and rv2). MNN codon (antisense codon, M = A, C and N = A, T, G, C) was introduced in rv2 to replace each of FWL residues with 20 amino acids. The primer fw2 and rv2 were modified with 5′ phosphorylation (Integrated DNA Technologies). Equal molar amounts of the oligonucleotides were annealed in NEB buffer 3 by gradually cooling the primer mixtures from 95 °C to 23 °C. T4 DNA polymerase and T4 DNA ligase were added to perform gap filling in the presence of 200 μM dNTP at 23 °C for 30 min followed by heat inactivation at 75 °C for 20 min in the presence of 10 mM EDTA. The resulting DNA fragments were fused to the DNA fragments containing DB and T7 terminator by overlapping PCR. The scheme for constructing the full-length PMI library is shown in Figure S1. The random full-length PMI DNA library should contain 20 × 20 × 20 = 8000 PMI variants. The actual frequencies of PMI variants were determined by deep-sequencing analysis (Supplementary Table S2).

### Microfluidic Device Fabrication

We fabricated polydimethylsiloxane (PDMS) microfluidic devices using standard soft lithographic methods. The microfluidic channel walls were rendered hydrophobic by treating them with Aquapel (PPG)^25^. To fabricate the device for sorting experiments, we filled the designed channels with Indalloy 19 (51In, 32.5 Bi, 16.5 Sn; 0.020 inch diameter), a low melting point metal alloy (Indium Corporation). We made electrical connections using eight-pin terminal blocks (Phoenix Contact)^26^. The microfluidic setup and design are shown in Supplementary Figure S4.

### Drop encapsulation and off-chip incubation

Linear DNA fragments expressing p53p-DB, PMI-DB, FSL-DB, FWR-DB (0.5 ng/μl) or the PMI library (1 ng/μl) were freshly diluted and added to the IVT2H reagents (25 μl) such that the final concentration of the binder templates was 10.8 fg/μl (8 fM). The solution was kept on ice to minimize transcription and translation before encapsulation into drops. A microfluidic device containing a flow-focusing junction with a cross section of 15 × 15 μm^2^ was used to encapsulate this solution into ∼3 million monodisperse drops with diameter of 24 μm (7.2 picoliter) in HFE-7500 fluorinated oil (3 M), containing 1% (w/w) Krytox-PEG diblock co-polymer surfactant (RAN Biotech)^27^,^28^. These 24 μm drops formed a cylindrical shape after the flow-focusing junction with a cross section of only 15 × 15 μm^2^. The drops had a larger diameter than that of the channel and were squeezed between the walls of the channel.

Rather than driving the flow using syringe pumps, we applied a house vacuum (−0.4 PSI) (Model 4172K12, McMaster-Carr Supply Company, Elmhurst, IL) at the outlet of the device to suck the reagents that were placed directly into the inlets through the microfluidic channels. The applied vacuum was not electrically controlled. Rather, the house vacuum was connected to the microfluidic drop maker through a vacuum regulator valve, which regulates the vacuum applied to the device. Simply by opening the valve and keeping the vacuum at a consistent value, we were able to generate monodispersed drops at a stable frequency. This vacuum-driven setup is robust, easy-to-use and low-cost. In addition, there are no initial transients in drop-size and no dead volumes of reagents, which are often seen inside the syringe, tubing and device in the pump-driven setup. These benefits make the vacuum-driven setup very suitable for encapsulation of a small volume of reagents for expensive biological assays. A recently published paper has described the detailed application of this setup and its advantages^29^.

**Figure 1:**
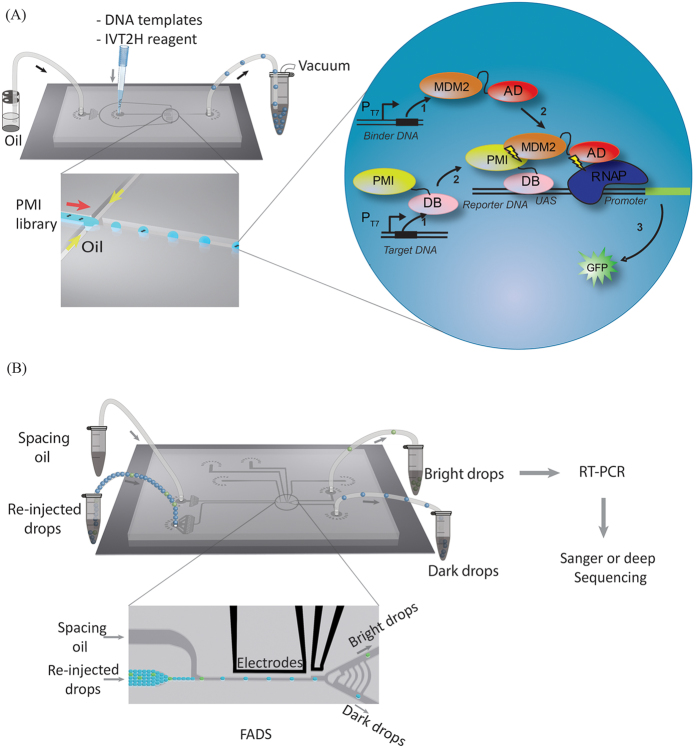
A schematic workflow for screening high-affinity binders using drop-based *in vitro* two-hybrid method (drop-IVT2H). (**A**) Single DNA templates and IVT2H reagents were encapsulated in drops on a microfluidic chip by applying vacuum to generate monodisperse drops. The binder DNA template was distributed as single copy in drops based on Poisson statistics. Each drop contained multiple copies of the reporter and target DNA templates. Drops were then collected for off-chip incubation. *Inset*, the IVT2H reaction during off-chip incubation. PMI-DB and AD-MDM2 were expressed from the binder and target templates, respectively. PMI-DB bound the upstream activation sequence (UAS) on the reporter DNA. The interaction of PMI and MDM2 recruited AD to the promoter-bound RNA polymerase (RNAP) and activated the expression of the reporter gene, producing the fluorescent GFP. (**B**) After incubation, drops were re-injected into a fluorescence-activated drop sorting device (FADS). Co-flowing spacing oil ensured equal separation of drops. Both bright and dark drops were isolated and collected in separate tubes for RT-PCR followed by Sanger or deep sequencing.

The generated water-in-oil emulsion was collected in a PCR tube directly, covered with mineral oil and incubated at 37 °C for 6 hr. This drop-making procedure is schematically shown in Fig. 1A. The actual image of the setup and the microfluidic chip designs are shown in Supplementary Figure S4. The typical drop generation rate was about several thousand drops/sec.

### FADS and drop collection

To detect and isolate bright drops containing PMI or high-affinity MDM2 binders, we used FADS as described previously^30^. The incubated drops were re-injected into a microfluidic sorter at a flow rate of 20 μL/h and evenly spaced by HFE-7500 oil with surfactant flowing at a rate of 180 μL/h. During this drop re-injection phase, we used two micro-liter constant-flow-rate syringe pumps and set the flow rates of the pumps at 20 μL/h and 180 μL/h to push the drops and spacing oil with surfactant into the microfluidic sorting device, respectively. Since drops and oil with surfactant are injected at different rates, only the pump system allows such controls in flow rates. The actual flow rates inside the microfluidic channels may be slightly different but have not been measured. Similar flow rate settings for drop sorting have been used in previous publications^19^,^31^,^32^.

The microfluidic device for Fluorescence Activated Drop Sorting (FADS) comprises a re-injection inlet that introduces drops and an oil inlet that spaces drops. The channels from these inlets intersect at a spacing junction, which is followed by a Y-shaped sorting junction connected to a sorting channel and a waste channel. The sorting channel is designed to have a higher fluidic resistance than the waste channel. As a result, all drops flow into the waste channel when sorting is not activated. We recorded the drop fluorescence as they passed through the detection region onto which a laser was aligned and their fluorescence was focused onto a photomultiplier tube (Hamamatsu). A custom computer LabView program running on a real-time field-programmable gate array card (National Instruments) digitized the photomultiplier tube signal, as shown in Fig. 1B. All drops were gated based on detector pulse width to exclude outliers, such as merged or split drops. When the fluorescence intensity of the drop was above a set threshold, the sorting electric field was turned on, resulting in a dielectrophoretic force that moved drops toward the sorting channel.

For no template, p53p-DB and PMI-DB, we examined ∼100,000 drops. For the PMI library, we examined ∼1,000,000 drops. For PMI-DB, we isolated five bright drops. To prevent evaporation and facilitate liquid handling for downstream processing of a small number of sorted drops, we preloaded the collection tip with 30 μl of carrier drops containing ddH_2_O. These five bright drops were mixed with 30 μl of carrier drops and distributed into 30 wells of a well-plate to ensure separation of individual bright drops. For the PMI library, we collected 13 bright drops together. As the quality control, we also collect 32,000 dark drops.

### RT-PCR amplification of binder templates in sorted drops

We broke the drop emulsion by adding 20% of 1H,1H,2H,2H-perfluorooctanol (PFO) (Alfa Aesar) followed by vortex and centrifugation. To prepare the samples distributed in 30 wells for Sanger sequencing, we added 5 μl of ddH_2_O to each well in order to facilitate transfer of the aqueous phase into 25 μL of the single-step RT-PCR cocktail. This cocktail contained 1 μL of OneStep RT-PCR Enzyme Mix with 1 × buffer (Qiagen), 400 μM dNTPs, and 0.25 μM forward and reverse primers. To prepare the samples from the PMI library for deep sequencing, we directly transfered the aqueous phase into the RT-PCR cocktail. Thermocycling conditions were 50 °C for 30 min, 95 °C for 10 min, 35 cycles of 95 °C for 30 s, 58 °C for 30 s, and 72 °C for 40 s, followed by 72 °C for 5 min. The PCR products were run on a 2% agarose gel and purified using GenElute™ Gel Extraction Kit (Sigma), either sent out for Sanger sequencing or processed for Deep sequencing.

### Deep Sequencing and data analyses

Illumina-specific adaptor sequences were attached to the 5′- and 3′-ends of the PCR fragments in two consecutive steps of PCR according to the manufacturer’s instructions. The sequencing condition was set to a read length of 56 base-pairs that covered the PMI peptide sequence region. Sequencing was run on an Illumina Genome Analyzer II (GAII) platform at the sequencing core facility at NEB. To analyse the sequencing data, we first removed Illumina adapter sequence and low quality bases (Q < 20) from the 3′ end of the raw reads by Cutadapt^33^. Each read was scanned for constant regions and the random mutated codons were extracted based on sequence syntax by custom perl script. The extracted DNA codons were translated to amino acid sequence using custom perl script. Next the peptide diversity at each mutagenesis position as well as the genotype (DNA sequence) diversity corresponding to each phenotype (peptide) was analysed using *R* software^34^ and listed in Supplementary Table S2.

## Results and Discussion

### The principle and strategy of the drop-IVT2H screening method

The drop-IVT2H screening method (Fig. 1) is based on the IVT2H system developed for the detection of protein-protein interaction in a bulk solution^20^. IVT2H contains a minimal set of components necessary for transcription activation and protein translation. IVT2H is designed to express two fusion proteins containing a bait and a prey, respectively. In the event of a high-affinity binding between the bait and the prey, the expression of a fluorescence reporter such as GFP is activated, resulting a detectable signal. This well-controlled *in vitro* system avoids the complications associated with the classic yeast two-hybrid (Y2H) system, such as efflux pumps, failure in transporting both fusion proteins to nucleus^35^,^36^,^37^,^38^,^39^, and high false positive rates (estimated to be from 44% to 91%) due to the intracellular complexity^40^. However, unlike other *in vitro* methods, such as ribosome and mRNA display, there is no mechanism in IVT2H to link the genotype (DNA sequence) with the phenotype (binding). Therefore, IVT2H is not suitable for screening a random DNA library. High throughput screening of individual binders using IVT2H in plate-based assay format would lead to prohibitive costs in the experimental reagents and other consumables.

Drop-based microfluidics have dramatically impacted high throughput screening methods by decreasing the assay volume from microliter to picoliter and at the same increasing the assay speed by orders of magnitude. For instance, using drop-based microfluidics, one can perform 10^8^ assays in drops in a single day at a cost of a few dollars. In stark contrast, even with the state-of-art high-throughput robotic instruments, it would take ∼2 years at a cost of ∼15 million dollars to perform the same number of assays in microtiter plates^18^. To enable the IVT2H method for high-throughput screening at reasonable costs, we compartmentalize single DNA molecules into picoliter drops with the IVT2H reagents. This not only creates a genotype-phenotype linkage between the DNA sequence of a high-affinity binder and its fluorescence signal, but also allows high throughput screening and sorting^41^,^42^,^43^ (Fig. 1). By using drops, the volume for each IVT2H assay decreases from microliter to picoliter and thus the cost for each assay decreases by six orders of magnitude. Simultaneously, the effective concentration of a single DNA in a picoliter is significantly higher than that in a bulk solution. The lowest concentration for a binder template in bulk IVT2H was previously found to be 2 pM^20^. The concentration of a single DNA in a picoliter drop is equivalent to 1 pM, which turned out to be sufficient for detection. In addition, the on-line compartmentalization procedure is very fast at 4000 drops per second, and highly automatic, which dramatically minimize the usage of consumable materials. In spite of these advantages of drop-based microfluidics, single DNA templates in drops often produce too few proteins in cell-free systems to be effectively assayed. To date this has only been possible for one template encoding a green fluorescent protein^44^. A current solution is to amplify single DNA templates using in-drop PCR, followed by drop-fusion to add reagents for protein synthesis and enzymatic assays. However such multi-step procedure significantly complicates the workflow^43^. By adapting IVT2H to drop-based microfluidics, we showed that we were able to perform a one-pot reaction for screening high-affinity binders at the single-DNA level.

### Detection of a known high-affinity peptide binder at the single-DNA level

To demonstrate the detection of a single DNA template in drop-IVT2H, we used a synthetic duodecimal peptide known as PMI as a model. Discovered by phage display, PMI binds to MDM2 with high affinity, thereby inhibiting the interaction of human p53 and MDM2 protein^23^. We fused PMI (binder) to the DNA binding domain (DB) to create the binder DNA template PMI-DB, and fused MDM2 (binding target) to the activation domain (AD) to create the target DNA template AD-MDM2. After expression of the hybrid fusion proteins from these templates, binding of PMI to MDM2 recruited AD to the promoter-bound RNA polymerase, thereby activating the reporter GFP expression (Fig. 1A inlet). We have previously demonstrate that in bulk IVT2H, PMI (Kd for MDM2 = 3.2 nM) resulted in higher fluorescence than the wild-type p53 peptide (p53p, Kd for MDM2 = 46 nM)^20^, which is consistent with the higher affinity of PMI compared to p53p^23^.

**Figure 2:**
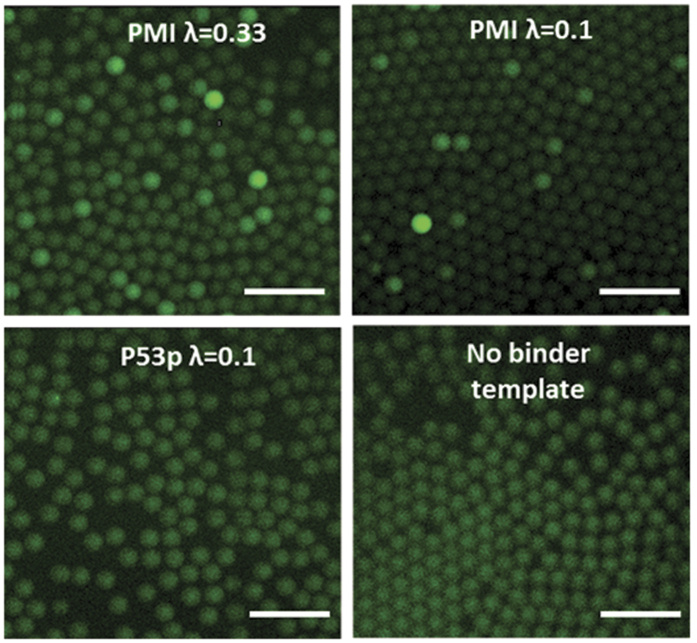
Fluorescence images of drops after off-chip incubation. (**a**) PMI at λ = 0.33 DNA per drop, (**b**) PMI at λ = 0.1 DNA per drop, (**c**) p53p at λ = 0.1 DNA per drop, and (**d**) no binder template. Scale bars are 100 μm.

**Figure 3:**
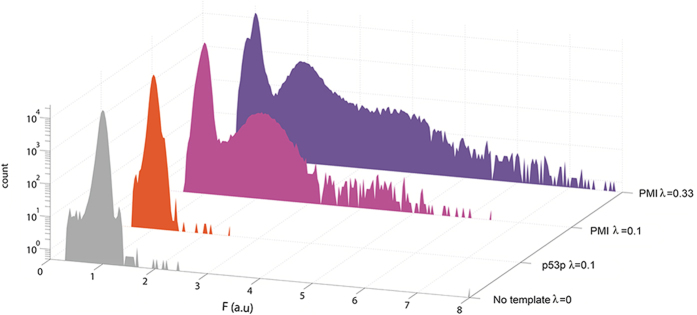
Histograms of the normalized drop fluorescence of sorted drops containing PMI, p53 or no binder template. For PMI, both λ = 0.33 and λ = 0.1 were measured; for p53p, only λ = 0.1 was measured; no binder template drops were also measured as a control. The Y-axis (count) is the number of drops that were counted. The X-axis is the normalized fluorescence intensity of each drop (F (a.u.)). The fluorescence of drops was normalized by the fluorescence of the population peak of drops with no binder template.

To allow the detection of a binder at the single template level, we Poisson loaded the binder template, p53p-DB or PMI-DB DNA, with IVT2H expressing AD-MDM2 in drops. We observed that only PMI-DB DNA template showed some bright drops (with noticeably higher fluorescence than that of the majority of drops), while p53p-DB DNA template showed drops with a similar low fluorescence as that of no binder template (Fig. 2). In the case of PMI-DB, loading at λ = 0.33 generated more bright drops and drops with higher brightness than at λ = 0.1, possibly due to the presence of two or more PMI-DB templates in single drops at λ = 0.33 (Fig. 2). To examine these drops in a high-throughput and quantitative way, we used our FADS device to measure the fluorescence of each drop that passed the laser detector and generated the fluorescence histograms (Fig. 3). Due to the Poisson loading, a majority of drops did not contain any binder template, but they exhibited a low level of fluorescence, since there was a basal activity of the RNA polymerase in IVT2H. Accordingly, in each set of experiments, we used the fluorescence of drops with no binder template to normalize the fluorescence of drops with binder templates. The histogram of p53p-DB exhibited a fluorescence distribution with one major peak at the normalized fluorescence of 1.0, which was identical to that of drops with no binder template. The data suggest that at the single DNA template level, the low-affinity binding of p53p to MDM2 did not generate detectable increase in drop fluorescence. In contrast, the histogram for PMI-DB exhibited a bimodal distribution with a second major peak at the normalized fluorescence of ∼2.0 (Fig. 3). The data suggest that the high-affinity binding of PMI to MDM2 resulted in bright drops with increased fluorescence compared to those with no binder template or with p53p-DB.

According to Poisson statistics at λ = 0.1 DNA per drop, 90.5% drops should contain no binder template, which corresponds to the first major peak at the fluorescence of 1.0 in the histograms, 9.0% drops should contain one binder template, corresponding to the observed second major peak with fluorescence at ∼2.0, and 0.5% drops should contain two or more binder templates, corresponding to signals with fluorescence >3.5 (Fig. 3). Setting a threshold value of the normalized fluorescence at 1.3, we counted that 4.9% of all sorted drops were bright drops, which could contain one or multiple binder templates. The discrepancy between the predicted and measured frequencies of bright drops, 9.5% vs 4.9%, could be due to the uncertainty of determining the absolute concentration of the binder DNA template. We verified that our Poisson loading was consistent with Poisson statistics by tripling the PMI-DB binder template concentration and observed additional peak signals corresponding to drops with two or more binder templates (PMI-DB λ = 0.33, Fig. 3 and Supplementary Figure S2).

To examine if bright drops indeed contained the PMI-DB template, we isolated five bright drops and broke the emulsion to retrieve the aqueous phase. In this aqueous solution there were two categories of nucleic acids carrying the genotype information, the input DNA template and the transcribed mRNAs. Since we estimated hundreds of mRNAs were generated from the single DNA template, amplification from mRNA should be more efficient and less biased than from DNA. We therefore performed RT-PCR rather than PCR to amplify a 350 bp region of the mRNA encoding PMI-DB. We successfully obtained the 350 bp amplicon from three of the five drops (Supplementary Figure S3) and subsequently confirmed their sequences by Sanger sequencing.

Taken together, these results suggest that drop-IVT2H increased the assay sensitivity, making it possible to detect the high-affinity binding at the single template level. We established the protocols to isolate bright drops and retrieved their sequence information. These experiments provided the basis for using drop-IVT2H to screen a random library for sequences that encode high-affinity binders.

### Enrichment of a high-affinity binder from a randomized mutant library using drop-IVT2H

The previous structural and biochemical studies have indicated that in the PMI sequence (TSFAEYWNLLSP), all three underlined residues of the hydrophobic triad, FWL, are most critical for binding to MDM2^23^. Therefore, we synthesized the PMI library from oligonucleotides containing NNK codons at these three positions, with N representing an equal mixture of A, G, C, and T, and K representing an equal mixture of G and T. This NNK library allows to avoid the stop codons UAA and AGA, as well as to reduce the total redundancy of codons and consequently screening efforts. The resulting randomized mutant PMI library should contain 8,000 sequences in total, with only one FWL (PMI) sequence. We assembled the random PMI library DNA fragment containing a T7 promoter from synthesized oligonucleotides (Supplementary Figure S1). To construct the full-length PMI library for drop-IVT2H, we performed overlapping PCR to add DB and T7 terminator to the random PMI library DNA fragment (Supplementary Figure S1).

In the next step, we encapsulated this full-length PMI library in drops with the IVT2H solution that also contained the target DNA template AD-MDM2 and the reporter DNA. To ensure the encapsulation of single templates, we diluted the PMI library DNA until Poisson distribution parameter λ = 0.1 DNA per drop^45^. We believe λ = 0.1 was the optimal condition for screening the PMI library, considering both the single molecule distribution and the sorting throughput. To screen a random DNA library, it is important that each DNA molecule of the library is well separated in drops, that is, one DNA molecule per drop. Multiple DNA molecules per drop would create a false positive due to the possibility that more proteins are made. Based on Poisson distribution, at λ = 0.1, we need to screen approximately 10 times more drops than the number of DNA molecules of a library, since ∼9% of drops containing one DNA molecule, while 90% of drops are empty and less than 0.5% of drops containing two or more DNA molecules. If λ < 0.1 is used, more drops are empty, we need to screen even more drops in order to cover the entire library, which limits the size of the DNA library we are currently capable of screening. If λ > 0.1 is used, there would be significant percentages of drops containing two or more DNA molecules, thus increasing the probability of false positives. For instance, at λ = 0.33, 3% of drops contain two DNA molecules.

We generated a total of three million drops from a 25 μl mixture of the IVT2H solution and the PMI library. The total number of drops with single PMI templates (*N*_PMI_) can be calculated as *N*_PMI_ = *N*_Drop_ λ/*N*_Library_, where *N*_Drop_ is the total number of microfluidics-generated drops and *N*_Library_ is the DNA library size. In the current case, *N*_Drop_ = 3 × 10^6^, *N*_Library_ = 8000, and λ = 0.1. Based on the above formula, there should be 38 PMI drops among three million generated drops, that is, 1 PMI drop per 1.2 × 10^5^ drops. If PMI is the only high-affinity binder in the random PMI library, we should observe one bright drop in every 1.2 × 10^5^ drops. However, if there are high-affinity binders other than PMI in the library, we should see the ratio of bright drops increases by multiplication. Different from the PMI-DB-only experiments described in the previous section, the random PMI library is not expected to have many high-affinity binders, thus the possibility of two high-affinity binders encapsulated in the same drop is extremely rare. Therefore, a bright drop is likely to contain just one high-affinity binder template and its significantly increased fluorescence is contributed only to this single molecule.

**Figure 4:**
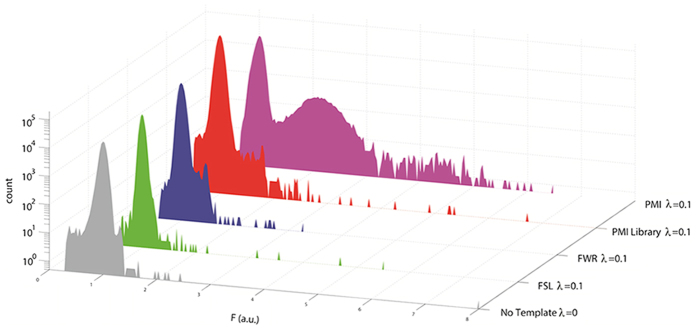
Histograms of the normalized drop fluorescence of sorted drops containing the random PMI library, PMI, its variants (FWR and FSL), or no binder template. DNA templates were diluted to λ = 0.1 DNA per drop. The Y-axis (count) is the number of drops that were counted. The X-axis is the normalized fluorescence intensity of each drop (F (a.u.)). The fluorescence of drops was normalized by the fluorescence of the population peak of drops with no binder template.

After off-chip incubation of the collected drops, we re-injected ∼1 million drops for detection and sorting in the FADS device. We observed 13 bright drops with a similar range of the normalized fluorescence as “pure” PMI when encapsulated at λ = 0.1 (Fig. 4). The calculated ratio of these bright drops to total sorted drops is one in 1.3 × 10^5^, which is nearly identical to our previous estimate for the PMI drops. This data suggest that PMI was the only high-affinity binder in the random library. We cannot exclude the possibility that a low-affinity binder was also co-encapsulated in the same drop as PMI even at λ = 0.1. However, it is not possible that every PMI drop contained the same low-affinity binder. Therefore, we were still able to distinguish the high-affinity binder from the low-affinity one by using the deep sequencing tool. In a separate sorting experiment, we collected 32,000 dark drops as a control.

**Figure 5:**
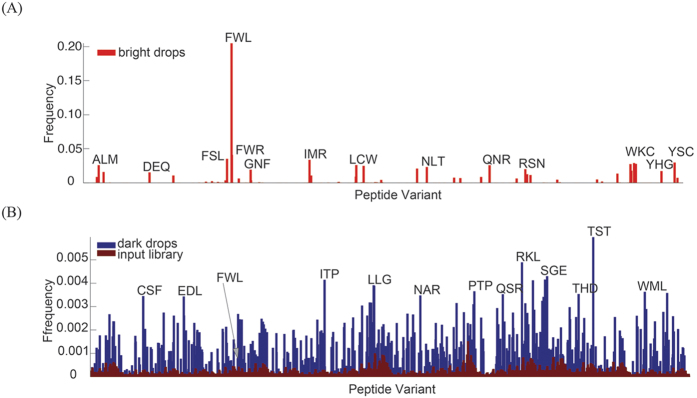
Frequency histogram representation of deep sequencing data from collected drops. The relative frequencies of peptide sequences from bright drops are shown in red (**A**). The relative frequencies of peptide sequences from dark drops (blue) and input library (dark red) are shown in (**B**). The PMI and some frequent peptide variants are indicated by three-residue sequences, such as FWL, FSL and FWR, corresponding to the randomized positions within the peptide sequence. The PMI sequence is FWL.

To confirm that the bright drops indeed contained the PMI template, we broke the emulsion of the collected bright drops and amplified mRNA via RT-PCR. Though dark drops did not generate significantly increased fluorescence, they contain mRNA with similar sequences as bright drops. Therefore, we also amplified mRNA from dark drops as a control. The resulting 350 bp DNA fragments from both bright and dark drops along with the random library were analysed by deep sequencing. The sequencing data of the input library confirmed that all 8000 intended sequences were present in the library (Supplementary Table S2), and therefore they were all assayed in drop-IVT2H. Based on the deep sequencing data, we plotted the frequencies of three residues that occur at the positions of the hydrophobic triad against all 8000 peptide sequences and generated the frequency histograms (Fig. 5). The histogram of bright drops indicates that the PMI sequence, FWL, occurred at 21%, which was significantly higher than other sequences (Fig. 5A, red). In comparison, the frequency of FWL in the histogram of the input library was only 0.043% (Fig. 5B, blue). Therefore, the isolation of bright drops by drop-IVT2H resulted in a 488-fold enrichment of the PMI sequence. In the control experiment, the frequency of FWL in the histogram of dark drops was 0.079% (Fig. 5B, dark red), indicating that there was no significant enrichment of the PMI sequence in dark drops.

The frequency histogram of bright drops also reveals a number of other peptide sequences that occurred at significant frequencies (Fig. 5A, labelled in three letters). We speculate that these sequences arose from *in vitro* transcription by T7 RNA polymerase as soon as the random library was mixed with the IVT2H reagents and before drop encapsulation. These mRNA transcripts were randomly distributed in drops and later amplified with varying biases by RT-PCR, which resulted in a wide range of frequencies observed in the deep sequencing data for bright drops. To confirm that slightly enriched sequences, such as FSL and FWR, were not high-affinity binders, we constructed individual DNA templates encoding FSL-DB and FWR-DB for drop-IVT2H analysis. Histograms of the normalized drop fluorescence show that the fluorescence distributions of FWR and FSL at the single-template level (λ = 0.1) were almost identical to that with no template (Fig. 4). Taken together, these results show that high-affinity binder templates were significantly enriched in bright drops, and it is feasible to use the drop-IVT2H system to sort and isolate high-affinity binder sequences from a random DNA library.

## Conclusion

We developed a drop-IVT2H method that enabled robust and high-throughput screening of high-affinity peptide binders at the single DNA template level and without pre-amplification and multistep drop manipulation. This mix-and-read drop-based microfluidic platform potentially provides a simple, fast and cost-effective way to discover peptide-based drugs, such as anti-microbial^46^ and anti-thrombotic peptides^47^ that can either interact with specific target proteins, or alter target protein-protein interactions. Unlike the majority of conventional antibiotics, antimicrobial peptides may also have the ability to enhance immunity by functioning as immune modulators^48^.

The major advantage of drop-IVT2H is that the expression of the binder and the target is coupled to the detection of the binding interaction in a continuous and streamlined drop-based microfluidic workflow. Unlike molecular display methods, there are no interruptive steps in drop-IVT2H, such as cell cultures, target immobilization, washing and elution. In fact, what distinguishes drop-IVT2H from all other screening methods is that drop-IVT2H does not require target purification and immobilization, and directly detects the binary binding interaction in solution rather than on a solid or cell surface (Table S1). Though in this work we demonstrated the screening of a peptide binder, drop-IVT2H can also be used to screen protein binders such as single-chain antibodies, antibody mimetics or protein ligands. Here a potential limitation is whether IVT2H can produce correctly-folded proteins. *In vitro* systems often have the disadvantage of expressing large and complex proteins due to the lack of appropriate folding environments compared to cell-based systems. Another limitation of drop-IVT2H is that it is difficult to screen the binding interaction that involves eukaryotic post-translational modifications such as glycosylation, ubiquitination, phosphorylation, etc., since IVT2H is derived from a prokaryotic cell. Nevertheless, recent advances in drop-based microfluidics have expanded to encapsulation and analysis of single cells in drops^49^. We have also demonstrated the potential use of a modified IVT2H for single-cell protein analysis in drop-based microfluidics^31^.

In this work, we screened and sorted 10^6^ drops in just half an hour, which was sufficient to assay a library size of 10^5^ molecules. With the state-of-art microfluidic techniques, we should be able to increase the library size to 10^6^, which may be suitable for screening a target-focused library where a few specific changes are made by site-directed mutagenesis^50^,^51^. To screen an even larger library that is comparable to other methods (Table S1), it may be possible to improve the sorting speed or load multiple DNA templates into each drop, followed by successive rounds of screening for further enrichments. Alternatively, drop-IVT2H could be modified to incorporate an alternative amplification scheme (10). Instead of activating a fluorescent reporter, drop-IVT2H may express a polymerase upon the protein-protein interaction to amplify the high-affinity binder template, which is then identified by deep sequencing. In spite of these challenges, drop-IVT2H represents a novel method that has far reaching potentials not only for screening high-affinity binders, but also for high throughput studies of protein-protein interactions.

## Additional Information

**How to cite this article**: Cui, N. *et al.* A mix-and-read drop-based *in vitro* two-hybrid method for screening high-affinity peptide binders. *Sci. Rep.* **6**, 22575; doi: 10.1038/srep22575 (2016).

## Supplementary Material

Supplementary Information
