# Supplementary material for: A mix-and-read drop-based in vitro two-hybrid method for screening high-affinity peptide binders
Source: Sci Rep. 2016 Mar 4;6:22575. doi: 10.1038/srep22575 (PMC4778045; doi:10.1038/srep22575)
Supplement: Supplementary Information [file srep22575-s1.pdf]

Supplementary information

A mix-and-read drop-based *in vitro* two-hybrid method for screening high-affinity peptide binders

Naiwen Cui[1], Huidan Zhang[1], Nils Schneider[1], Ye Tao, Haruichi Asahara, Zhiyi Sun, Yamei Cai, Stephan A. Koehler, Tom F. A. de Greef, Alireza Abbaspourrad, David A. Weitz*, Shaorong Chong*

[1] These authors contributed equally to the study.

* Corresponding authors

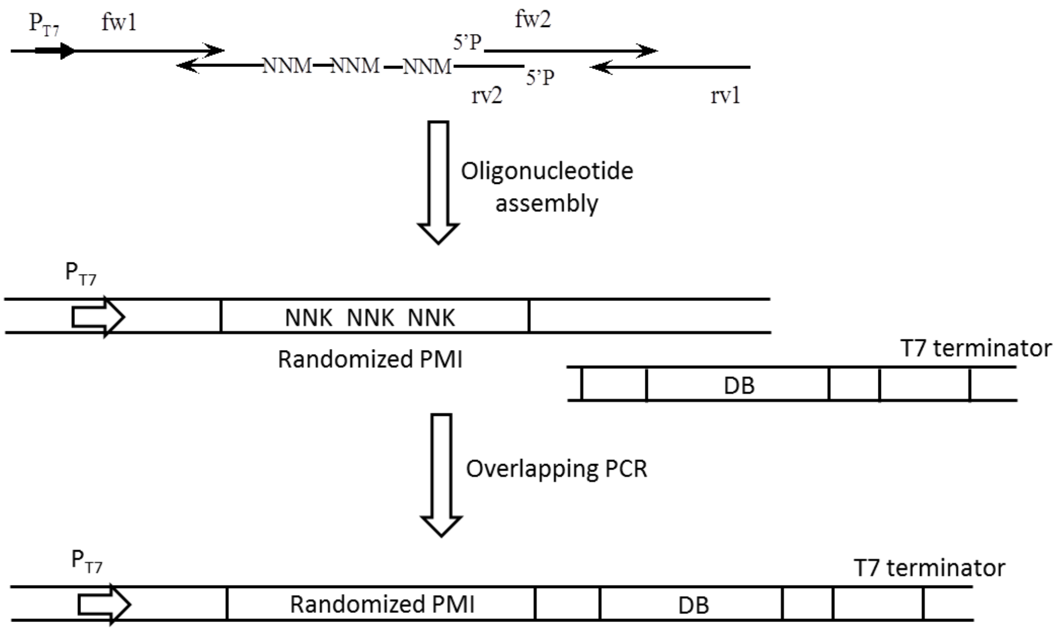

Supplementary Figure S1. The scheme for constructing the full-length PMI library via oligonucleotide synthesis and overlapping PCR. P_{T7}: T7 promoter. NNK represents a randomized residue of the hydrophobic triad in the PMI sequence. MNN is antisense codon of NNK. DB: DNA binding domain.

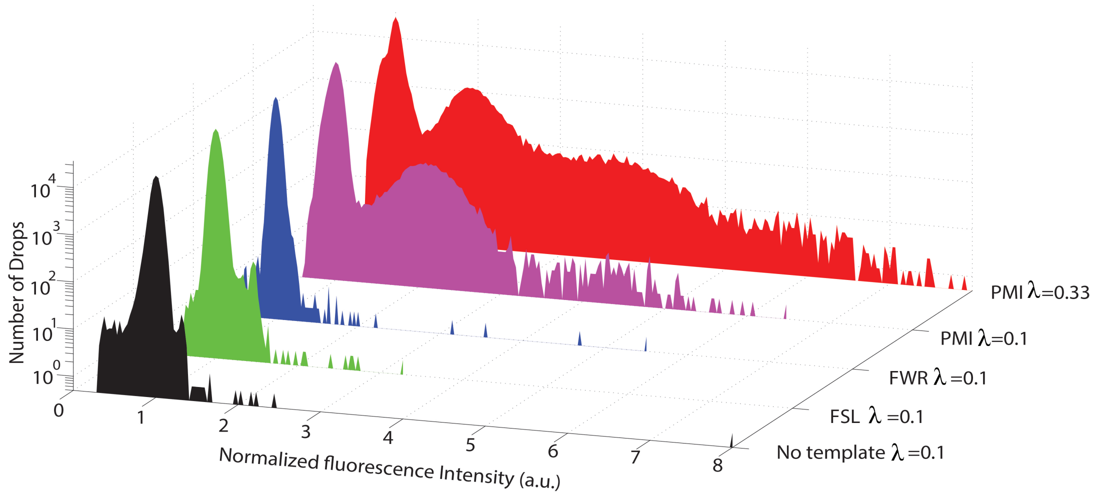

Supplementary Figure S2. Histograms of normalized drop fluorescence for no binder template (no template), FSL, FWR, PMI templates at λ= 0.1 DNA per drop and PMI template at λ= 0.33 DNA per drop. The fluorescence of the population peak of drops with no template is used for normalization.

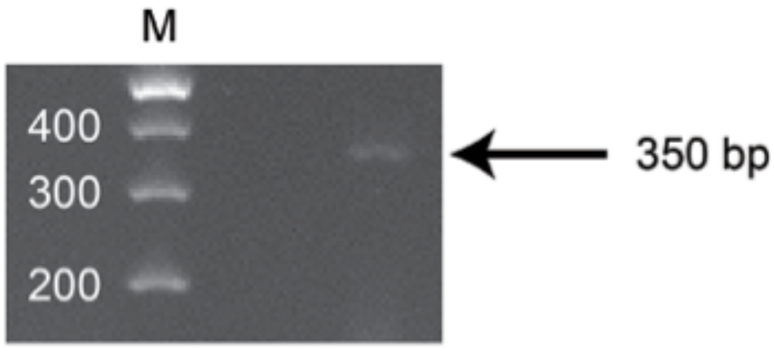

Supplementary Figure S3. Agarose gel image of the DNA fragment from RT-PCR amplification of a 350 bp region of mRNA encoding PMI-DB. M: 100 bp DNA marker.

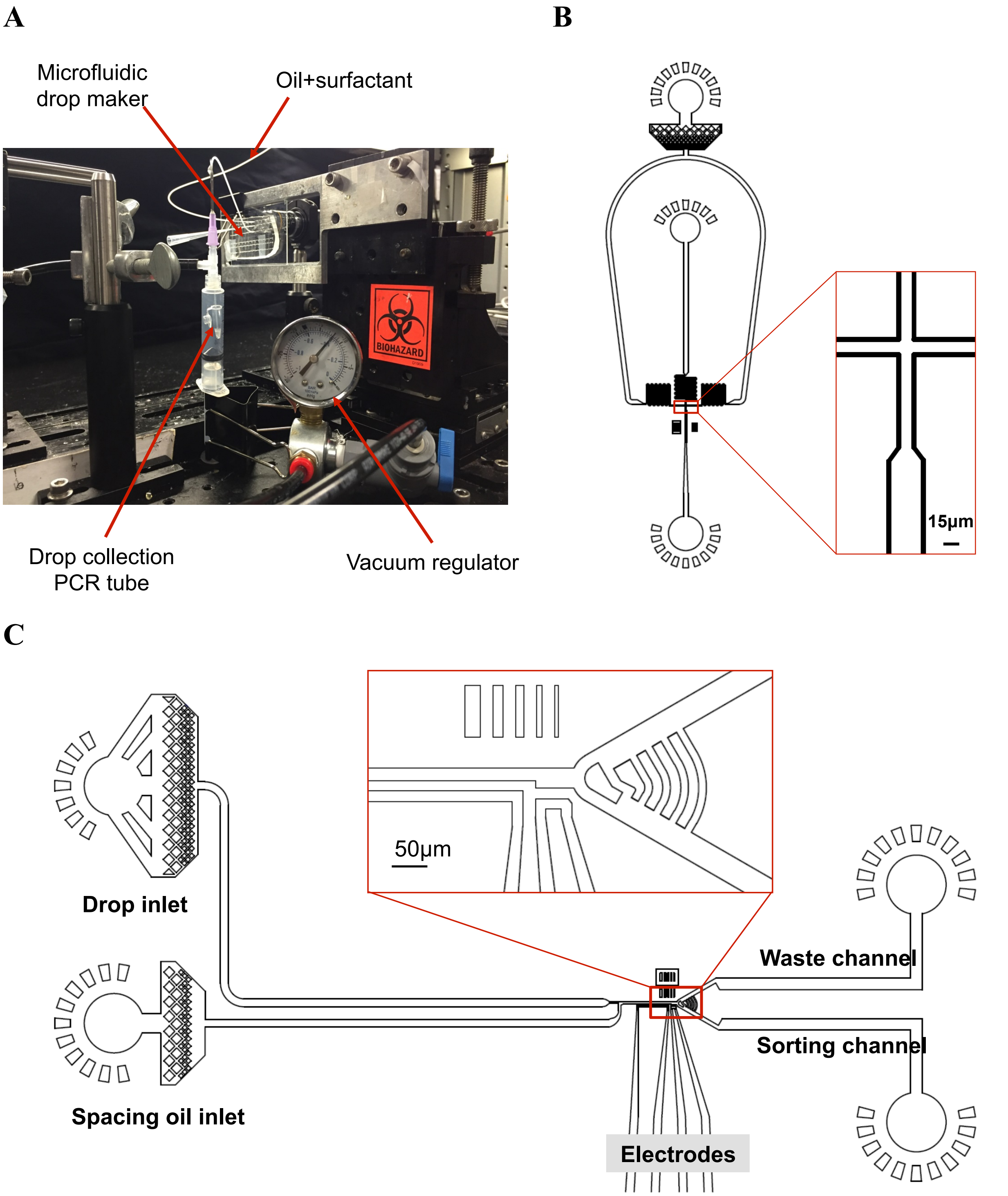

Supplementary Figure S4. The microfluidic device setup and design. (A) the photo image of the drop-making and collection setup; (b) a schematic diagram of the drop-making device design; (C) a schematic diagram of the re-injection and sorting design.

Table S1. Deep sequencing data showing frequencies of three-residue sequences before soring (input library) and after sorting (dark and bright drops).

**Input library**	**Frequency**	**Dark drops**	**Frequency**	**Bright drops**	**Frequency**
PPP	0.001520457	TST	0.00596993	FWL	0.205117503
PPR	0.001115407	RKL	0.00489078	FWR	0.04112783
LLL	0.001004387	SGE	0.00429911	FSL	0.035290452
PPA	0.000949744	SES	0.00415386	IMR	0.033877197
LLR	0.000945841	ITP	0.00414601	YSC	0.029907611
LRL	0.000942805	RSS	0.00412246	WKC	0.029032965
PRP	0.000925892	LLG	0.00391156	WLG	0.027912589
LLS	0.000921121	LLP	0.00389229	WGQ	0.027603245
LSL	0.000919387	SFW	0.00376132	LCW	0.026173276
LRR	0.000898137	PTP	0.00365497	ALM	0.026098434
PRR	0.000889463	WML	0.00362928	QNR	0.025934532
LSR	0.000855203	YGL	0.00358182	LHS	0.024957852
RLR	0.000835254	THD	0.00353721	NLT	0.023275917
SLS	0.000835254	QSR	0.00353043	NEH	0.020965065
SLL	0.00083482	NAR	0.00347797	RSN	0.020136321
RLS	0.000809667	CSF	0.00344799	GNF	0.01932554
SLR	0.000804463	EDL	0.00343086	WHH	0.017819731
LRS	0.000801861	SDT	0.00341409	YHG	0.01734848
RLL	0.000800127	SDA	0.00324423	APS	0.015919259
RRR	0.000798392	LHQ	0.00317856	DEQ	0.015394871
SSL	0.000795356	LIP	0.00316928	VTY	0.013623625
LSS	0.000785815	PFT	0.00315251	RTS	0.012690103
SRL	0.000778009	RMQ	0.00314181	RYA	0.011467944
SRR	0.000772805	PTA	0.00310219	INT	0.010807094
RRL	0.0007663	LCN	0.00304296	DYP	0.010791877
SRS	0.00076153	TAE	0.00297587	LCN	0.009135138
SSR	0.000744183	QNW	0.00293483	AKH	0.008689582
SSS	0.000742014	WNL	0.00289807	QHA	0.008675612
RSL	0.000738111	RTP	0.00286488	PIY	0.00770916
RSS	0.00073681	YDW	0.0027746	YTY	0.007417529
RSR	0.000717729	PLQ	0.00275961	PPD	0.007019622
PRA	0.000712958	RDL	0.00274391	RLT	0.006445339
PLR	0.000709055	DLA	0.00274355	GEH	0.00642588
RRS	0.000706453	RPA	0.0027307	VEL	0.004967721
LPR	0.000697346	MPA	0.00269894	STT	0.004704778
PPS	0.000679999	FWR	0.00268467	LWL	0.004363751
PRL	0.00067306	APA	0.00267253	FRL	0.003641033
LPS	0.000660484	MST	0.00266183	GNL	0.00359987
LPL	0.000657882	EDQ	0.002624	FGL	0.002368979
LLT	0.000653111	LGA	0.00261758	VHS	0.001905461

RPR	0.000650943	DSV	0.00261437	FCL	0.001782971
PRS	0.000650076	WGD	0.00260758	FWM	0.001661977
SPR	0.000647474	YLI	0.00258689	KMG	0.001451923
PPL	0.000641836	TFL	0.00255477	FLL	0.001268312
VLL	0.000641402	VMK	0.00255298	FRR	0.000972189
ARR	0.000633163	KSP	0.00253871	LRL	0.000882879
LLP	0.000632295	QCE	0.00252872	FWP	0.000791822
LRP	0.000631428	TLV	0.00251373	LSL	0.000752904
PLS	0.000630127	GRH	0.00248233	GTY	0.000744921
LSP	0.000627091	TAL	0.00246448	LWR	0.000741429
LTR	0.000627091	FYQ	0.00245628	SWL	0.000694029
LLA	0.000623622	GCD	0.00244735	LWW	0.00067482
TLS	0.000623622	INT	0.00244129	LCL	0.000646131
VLR	0.000623622	ARQ	0.00237241	FWS	0.000645382
LRT	0.000620586	ASA	0.00236992	FWV	0.000623179
LST	0.000620152	WVH	0.00236992	LGL	0.000599978
PLL	0.000619719	QTS	0.00236171	KLT	0.000571788
TLL	0.000619285	GSF	0.00235707	LGQ	0.000567547
LPP	0.000617117	LKT	0.00233994	FSF	0.000563306
PSR	0.000616683	KLV	0.00230818	VWL	0.000557818
VSL	0.000616249	ELL	0.00230425	FGR	0.000528131
RLT	0.000614515	DWL	0.00230211	LCR	0.000520896
SPL	0.000614515	NNA	0.00229997	FCR	0.000510169
VLS	0.000612346	MFR	0.00229854	FMR	0.000504181
LGL	0.000609311	PPH	0.00229248	FHS	0.000494951
LTL	0.000608877	PRA	0.00224858	IMS	0.000480981
LTS	0.00060801	LST	0.00224608	FPL	0.000473746
PSL	0.000604106	RPR	0.00224608	FWQ	0.000439319
ALR	0.000602372	QPR	0.00224573	RKC	0.000436325
SLT	0.000598469	RNA	0.00224359	FWW	0.000431835
TLR	0.000598469	GST	0.00223752	GYA	0.000430088
ALL	0.0005963	NSC	0.00222039	LCC	0.000430088
LVR	0.0005963	RPM	0.00217935	FSC	0.000419361
RPL	0.000590663	ARP	0.00215259	LKC	0.000414122
SLP	0.000588928	WGQ	0.0021508	WKR	0.000410131
RTS	0.000587193	LSA	0.00214331	GKC	0.000404393
LVL	0.000585892	FMS	0.00213688	FCW	0.000403395
LRA	0.000581555	KLM	0.00212297	LLG	0.00040115
TRR	0.000580688	PDW	0.00212261	KEH	0.000400651
ALS	0.000580254	SRI	0.00211155	LNS	0.00038668
ASL	0.000579821	RDR	0.00208835	WFG	0.000383188
SPS	0.000579387	KSD	0.00207907	WGH	0.000378947
GLL	0.000578953	GTS	0.00205516	ILR	0.000376203
LVS	0.000578953	FKL	0.00205017	ATY	0.000360486

GRL	0.00057852	CTL	0.00204481	FLR	0.00035874
VRR	0.00057852	GAD	0.00204481	SIV	0.000356993
GLR	0.000574617	WIS	0.00204018	YSW	0.000352503
ARL	0.000574183	YPR	0.0020391	ETY	0.000345767
RPP	0.000573749	GTN	0.00202733	RLG	0.00034427
TRL	0.000573749	LPQ	0.00201948	WKW	0.000342275
VRL	0.000573316	HTA	0.00200734	YSR	0.00033953
RTL	0.000572882	GAS	0.00200449	FSR	0.000339031
VRS	0.000572015	LSW	0.00200271	IWL	0.000333793
PPG	0.000571581	ENM	0.00200199	EEQ	0.000330549
RRP	0.000571581	EEY	0.00198022	LWN	0.000322816
PSP	0.000569846	IRN	0.00197987	YWL	0.000318325
SST	0.000568545	HST	0.00197737	FSW	0.000314084
SPP	0.000566811	VQT	0.00197344	ALL	0.000313835
LAL	0.000566377	IQR	0.00193776	FSS	0.000312089
STS	0.000565943	YLW	0.00193704	ALI	0.000309095
RLP	0.00056551	ENH	0.00193312	PNR	0.000307099
VSR	0.000565076	FKQ	0.00191991	LSN	0.000304355
RPS	0.000563341	SAQ	0.00191991	WIG	0.000300862
LLV	0.000562474	KDH	0.00191492	EYP	0.000298368
GLS	0.000561606	YFL	0.00191349	FWT	0.000293129
LGR	0.000559872	GVW	0.00190564	FTL	0.000292131
APR	0.000559438	LAC	0.00190207	LMR	0.000280655
RST	0.000557703	EPW	0.00190171	GLG	0.000274668
PSS	0.000555535	MTS	0.00190029	LRR	0.000274668
RRT	0.000555535	LYG	0.00189208	RST	0.000274169
ARS	0.000550331	TLA	0.00188244	RHH	0.000270177
RTR	0.000550331	GQA	0.00187566	SLM	0.000269179
SLA	0.000546428	ISV	0.00187388	NMR	0.00026893
LAS	0.000545127	RWF	0.00186853	FWG	0.000267932
TSL	0.00054426	GRV	0.00186745	GEQ	0.000266685
VSS	0.000543392	HFH	0.00186424	WWL	0.000265188
LLG	0.000539489	HLT	0.00182677	RGQ	0.000263941
SRP	0.000539489	RGF	0.00181999	FLM	0.000263691
TSR	0.000539489	RST	0.00181892	FRM	0.000262444
APP	0.000539056	RAD	0.00180572	LYS	0.000261196
STL	0.000538622	AWK	0.00180286	WNC	0.000258702
RRA	0.000537321	ALP	0.00177003	LQS	0.000257704
PLP	0.000536887	AGP	0.00176967	QSR	0.000255708
RSP	0.000536453	PPW	0.00176432	LLL	0.000253712
RLA	0.000535152	IFL	0.00175826	RSH	0.000253712
SSP	0.000533418	CLQ	0.00175005	DEH	0.000251218
GRS	0.00053255	VRH	0.00173898	LHH	0.000251218
GSL	0.000531249	LWR	0.00173542	RSS	0.000249222

LAR	0.000530382	VEP	0.00172828	GKA	0.000247974
SRT	0.000529515	ARD	0.00172578	QKR	0.000246977
TRS	0.000529081	NWH	0.00172043	NWD	0.000242486
TPR	0.000527346	MRT	0.00170508	GGQ	0.000240989
STR	0.000526045	KCV	0.00170151	WGL	0.00024049
LSA	0.000524744	MLG	0.00169723	AHS	0.000239991
GRR	0.000524311	RDC	0.00169259	RPN	0.000239742
SVR	0.000521275	LGE	0.00168974	WKS	0.000238245
ASR	0.000520408	SLF	0.00168867	FHG	0.000237996
RAS	0.000518673	SFL	0.00168046	KNR	0.000235002
RVS	0.000518673	HTW	0.00166476	FYL	0.000234503
SVL	0.000518673	MRR	0.00164906	GHH	0.000229763
ASS	0.000517372	VSP	0.00164763	GTS	0.000229514
SRA	0.000513902	IIV	0.00164691	IIR	0.000228516
RAR	0.000513469	WTT	0.00163906	FKL	0.000228266
TSS	0.000512601	RTH	0.00163371	LRW	0.000227767
SAL	0.000511734	QDM	0.00163264	WWQ	0.000227518
LSV	0.000510867	QRC	0.00162622	FAL	0.000226769
PTR	0.000510433	HSV	0.00162158	FGQ	0.000226769
LGS	0.000507397	SPK	0.00162086	YSS	0.000226769
LRV	0.000507397	GSV	0.00160052	CWL	0.000220034
SAS	0.000504362	NMV	0.00160017	FEL	0.000218786
RVL	0.000503928	PRP	0.00159374	WNH	0.00021704
SVS	0.000503061	FST	0.00159267	PCW	0.000216541
PRG	0.000502193	SCS	0.00158411	YYC	0.000215294
SSA	0.000501326	YHL	0.00157911	WLR	0.000214046
SAR	0.000499591	TGN	0.00157447	NFT	0.000210803
LRG	0.000499158	SYA	0.00157376	VLM	0.000210803
PAR	0.000491351	ANA	0.0015734	GKF	0.000210554
GSR	0.00049005	EGT	0.00156162	LWQ	0.00020756
RLV	0.000487882	YFR	0.00155841	GTF	0.000205065
RRG	0.000487015	IAT	0.00155734	WLV	0.000204067
SLG	0.000487015	RMH	0.00154378	WTC	0.000203319
ARP	0.000486581	PRV	0.00154271	LCS	0.000201323
RVR	0.000486581	MCG	0.00153308	LNR	0.000201323
RSA	0.00048528	GKL	0.00152986	LCG	0.000200575
GSS	0.000483979	MPV	0.00152558	LHP	0.000200076
ARA	0.000481811	YHA	0.00152273	WGR	0.000200076
PLA	0.000480076	IRQ	0.00151238	MMR	0.00019808
RAL	0.000480076	PIV	0.00151238	VSL	0.000197831
PPT	0.000479642	CLW	0.00150702	FSV	0.000196833
SLV	0.000479642	TGS	0.00150381	QNS	0.000196833
APS	0.000479209	ANN	0.00150132	YNG	0.000194837
APL	0.000478341	KFP	0.00149703	LWV	0.000194588

PLT	0.000477474	ELG	0.00148419	ALT	0.000194089
LPA	0.00047704	DSM	0.00147848	VRL	0.000194089
RGL	0.000476607	SWS	0.00146848	FSM	0.000193839
RLG	0.000474005	CLP	0.00146349	WKG	0.000191843
RPA	0.000470969	TPC	0.00145243	NEQ	0.000191594
PTS	0.000467499	DGH	0.00145171	HNR	0.00018885
LSG	0.000466632	FNF	0.001451	GNS	0.000187852
PRT	0.000465765	MAR	0.001451	QTR	0.000187103
PTL	0.000465765	LPF	0.00144457	RNR	0.000186854
PTP	0.000465331	TFR	0.00144386	GLM	0.000186604
TLT	0.000464464	DGT	0.00143708	WVG	0.000186355
ALT	0.000463163	LRG	0.0014353	FRV	0.000186106
SRG	0.000463163	NRF	0.00142923	WLA	0.000185856
SSV	0.000463163	LKQ	0.00142744	GPS	0.000185357
SRV	0.000459693	RVL	0.00142602	LWM	0.000184359
TPL	0.000459693	WRL	0.00141924	LRS	0.000182862
TRP	0.000459693	NFM	0.00141067	GYP	0.000182114
SGL	0.00045926	RLH	0.00140853	WNF	0.000181116
SGS	0.00045579	GID	0.00140461	WGK	0.00017937
TPS	0.00045579	EIT	0.0013939	LHA	0.000178372
FRL	0.000454489	LNW	0.00138712	RKS	0.000178122
PSA	0.000453188	IPE	0.00137998	QNC	0.000177873
LTT	0.00045102	PLL	0.00137713	AMM	0.000177624
TPP	0.000450586	DIS	0.0013625	YSG	0.000176626
VTL	0.000450586	TRT	0.00135928	LKR	0.000175877
RRV	0.000450153	KYS	0.00135429	YSL	0.000175877
RSV	0.000448418	TGL	0.00134215	PSN	0.000175378
SPA	0.000447551	EMY	0.00132253	ICW	0.000175129
ATR	0.000447117	GDS	0.00132146	SRL	0.00017463
RGR	0.000446249	YES	0.0013186	APM	0.000174131
RGS	0.000445382	AST	0.00131717	YLT	0.000174131
FSL	0.00044278	LVA	0.00131646	ATS	0.000173881
TTL	0.000442346	LVI	0.00131325	DLM	0.000173632
VLT	0.000442346	TVL	0.00129826	PLM	0.000171636
NLR	0.000440612	LSS	0.00128613	GNC	0.000171137
SGR	0.000439311	CVV	0.00128541	GNV	0.00016964
VPR	0.000437576	EQL	0.00128506	LHL	0.000167645
PAP	0.000433673	SNF	0.00128113	LGR	0.000167395
NLS	0.000432372	RYS	0.00127863	LKL	0.000167395
LTP	0.000431505	KCF	0.00127756	IRR	0.000166896
FWL	0.000431071	NSP	0.00127756	APP	0.000165898
TSP	0.000431071	PHR	0.00126579	FRS	0.000164402
LPT	0.000428469	DAL	0.00126365	LSW	0.000164152
APA	0.000426734	TFC	0.00126043	NGH	0.000163653

VLP	0.000426301	ALQ	0.00126008	GEL	0.000163404
FLL	0.000425867	YST	0.00126008	RTN	0.000162905
LAP	0.000425867	NNV	0.00125508	GQA	0.000162156
GLT	0.000424566	LND	0.00124866	LSR	0.000161657
PGR	0.000424566	AEL	0.00124152	TMR	0.000160659
VTR	0.000424132	HCW	0.00123688	WEQ	0.000160161
ATL	0.000423698	KPL	0.00123545	FRP	0.000159911
TST	0.000422831	PRW	0.0012351	APY	0.000158913
VPL	0.000422831	DHF	0.00123153	WLC	0.000158414
VST	0.00042153	HWF	0.00122867	IMM	0.000157915
DLS	0.000421096	INY	0.00122832	VMR	0.000157915
GTL	0.000421096	RDQ	0.0012276	WRQ	0.000157416
TLP	0.000421096	RTK	0.00122653	GKH	0.000156668
VRP	0.000420229	YTF	0.00122618	VGL	0.000156418
TTR	0.000419795	SLA	0.00122296	FQL	0.000156169
HLS	0.000418494	MNL	0.00121797	NLM	0.00015592
LWL	0.000418494	QWI	0.00121725	LLR	0.000155171
TLA	0.000418061	KPY	0.00121404	GNI	0.000154922
PAL	0.000415892	GSL	0.00121333	ITR	0.000154922
TRT	0.000415025	QPH	0.00121261	FFL	0.000153924
VRT	0.000414591	TGA	0.00121083	WGP	0.000153674
ART	0.000414158	TRD	0.00120476	NLR	0.000152926
GTR	0.000414158	PVI	0.00120369	WQH	0.000152177
ALP	0.000411556	FGA	0.00120262	WHS	0.000151928
LTA	0.000410255	SHG	0.00120084	LWP	0.00015093
AST	0.000409387	NLK	0.00119263	WHG	0.000150182
SPT	0.000408954	NKT	0.00118906	GSF	0.000149932
VPS	0.000408086	PEP	0.00118906	WYH	0.000149184
ATS	0.000407653	HTQ	0.00118763	LPS	0.000148685
TTS	0.000406352	WLS	0.00118585	GEA	0.000147188
RSG	0.000405918	DCC	0.00118192	MTS	0.000146939
NLL	0.000404617	RTR	0.00117621	VEQ	0.000145941
SSG	0.000404617	AGA	0.00117122	NAH	0.000145442
ASP	0.00040375	GSN	0.00117122	LYL	0.000144943
RTT	0.000403316	ERN	0.00116694	GKL	0.000143945
PST	0.000402882	TCV	0.00116408	YPC	0.000143945
VTS	0.000402882	SIT	0.00116301	IMG	0.000143196
PAS	0.000402015	HLV	0.0011598	NST	0.000142947
NLT	0.000401147	PPP	0.00115623	WTS	0.000142947
VVR	0.000401147	SIE	0.00115623	RTY	0.000141201
GTS	0.000400714	WTP	0.00114874	SSL	0.000140702
VSP	0.000399846	NGT	0.00114731	ARM	0.000139704
TRA	0.000398979	MLL	0.00114089	LYA	0.000139205
STP	0.000398545	PSA	0.00113981	SPS	0.000138706

ALA	0.000397678	WGE	0.00113553	ALV	0.000138457
LAT	0.000397244	GLS	0.00113196	VNF	0.000138207
DLL	0.000396377	KTP	0.00112304	CGQ	0.000137209
PRV	0.000396377	NCH	0.00112233	YYG	0.000136461
LVT	0.00039551	MNT	0.00112161	HLT	0.000136211
VVL	0.00039551	VTR	0.00112126	VTH	0.000136211
RTP	0.000395076	QYN	0.00111805	WSN	0.000135962
AAR	0.000394642	MGH	0.00110377	DLT	0.000135712
HLL	0.000394209	WWR	0.0011027	RAN	0.000135712
PLG	0.000394209	DRA	0.00110056	YHW	0.000135463
VLA	0.000393341	SAD	0.00109556	FRQ	0.000133966
YLL	0.000393341	DPR	0.00109414	WLD	0.000133966
DLR	0.000392908	QDR	0.00109128	LGW	0.000133717
RPT	0.000392474	TIY	0.00108771	LWS	0.000133717
GPR	0.000391607	REP	0.00108557	PPS	0.000133717
LGT	0.000389872	NTL	0.00108521	TLT	0.000133717
RPG	0.000388137	LFN	0.00107736	WVQ	0.000133717
LAA	0.000387704	VLS	0.00107629	FNT	0.000133467
PTA	0.000386403	PTR	0.0010738	VWR	0.000132968
STT	0.000386403	HNQ	0.0010688	NDH	0.000132469
VRA	0.000385535	HFL	0.00105881	YHR	0.000132469
FLR	0.000384668	NMG	0.00105452	ALR	0.00013222
GPL	0.000384234	ERT	0.00105417	YEQ	0.00013222
GRT	0.000384234	FAY	0.00105203	YSD	0.00013222
LVP	0.000384234	ARE	0.0010456	IWR	0.000131471
ASA	0.000383801	LRP	0.00103632	ILT	0.000131222
LPV	0.000383801	LRW	0.00103311	LTY	0.000131222
GRP	0.000383367	MYT	0.0010324	RLS	0.000131222
VGL	0.000382933	LTD	0.00102669	TLM	0.000129975
TPA	0.0003825	LLM	0.00102383	WLS	0.000129725
HSL	0.000381199	RQL	0.00102241	GSA	0.000129476
VVS	0.000380765	PRR	0.00102027	LSS	0.000128977
YLS	0.000380765	KCQ	0.00101884	CKC	0.000128727
FRR	0.000380331	HPH	0.00101349	RSD	0.000127729
PVR	0.000380331	GGP	0.00101313	PPA	0.000126981
KLR	0.00037903	EPN	0.00100456	FTY	0.000126731
KLL	0.000378597	SGD	0.00099207	QNA	0.000126731
PAA	0.000378597	NPL	0.00098387	PYA	0.000126482
HLR	0.000378163	YRS	0.00097066	NEN	0.000126232
RTA	0.000377295	SGV	0.00097031	YDW	0.000126232
NSS	0.000376428	LTL	0.00096888	NSC	0.000125983
TVL	0.000375561	SSD	0.00096888	PLY	0.000124985
GST	0.00037426	SRA	0.00096031	PCN	0.000124486
LPG	0.00037426	NLD	0.00095996	PPG	0.000124486

STA	0.00037426	YLF	0.00095996	CLG	0.000123987
PGL	0.000373826	SSI	0.00095817	FML	0.000123239
VAL	0.000372525	PAG	0.00095603	WEC	0.000122989
GRA	0.000371658	IRL	0.00095389	LPL	0.000122241
LVA	0.000371658	GML	0.00094925	KTS	0.000121742
HSR	0.00037079	GTK	0.0009439	RPS	0.000121742
AGR	0.000370357	MEP	0.0009414	WAQ	0.000121742
FLS	0.000369923	EMF	0.00093854	WRC	0.000121492
TLV	0.000369923	DAR	0.00093747	FWK	0.000120994
KLS	0.000369056	CTS	0.00092998	WRH	0.000120744
LCL	0.000369056	AYS	0.00092249	LSD	0.000120495
TVR	0.000367755	YTY	0.00091356	RSK	0.000120495
RAP	0.000367321	SSR	0.00091071	FLT	0.000120245
YLR	0.000366887	LSL	0.00088716	APA	0.000119996
GLA	0.00036602	YSA	0.00088502	RSY	0.000119746
LGA	0.00036602	FPE	0.00088252	LLS	0.000119497
LHS	0.00036602	SCR	0.00088252	VPS	0.000119247
LVG	0.000365586	QNN	0.00088038	LDW	0.000118748
NRL	0.000365586	WTA	0.00087788	LTL	0.000118748
ALV	0.000365153	SSW	0.00087752	IVR	0.000117002
ARV	0.000364719	GNP	0.00087431	SLT	0.000117002
HRR	0.000364719	LGR	0.00087324	EPS	0.000116753
VSA	0.000364719	ARI	0.00086146	HSC	0.000116254
GLP	0.000364285	SLS	0.00085968	WRG	0.000116254
YSL	0.000363852	SMS	0.00085861	DEL	0.000115505
GAL	0.000363418	IKT	0.00085789	YEH	0.000115505
HSS	0.000362984	SHT	0.00085111	QSN	0.000115256
TSA	0.000362984	NVM	0.0008372	SSC	0.000115256
AAL	0.000361683	CVH	0.00083327	FCS	0.000114757
NSR	0.00036125	RHN	0.00083291	RTP	0.000114757
GSP	0.000360816	CPF	0.00082578	RTC	0.000114507
RAT	0.000360382	YKY	0.00082328	SWR	0.000114507
AVR	0.000359949	NTM	0.00082292	LNT	0.000114258
TLG	0.000359949	IIR	0.00082007	VLL	0.000114008
PGS	0.000359515	RMP	0.00081721	LDS	0.000113509
ELL	0.000358648	MKY	0.00081471	FWH	0.00011301
GGL	0.000358648	GTF	0.00080972	IMT	0.00011301
GGR	0.000358648	ARR	0.000809	LRV	0.00011301
NSL	0.000358214	IGC	0.00080615	WRL	0.000112761
VAR	0.000358214	IHD	0.00080365	LRP	0.000112262
LGP	0.00035778	TSC	0.00080294	QDR	0.000112262
VLV	0.00035778	CVN	0.00080222	QHR	0.000112013
ARG	0.000356479	GRF	0.00079866	WPG	0.000111763
DSL	0.000356479	INH	0.00079723	YQG	0.000111763

PLV	0.000356046	RQS	0.0007958	DSC	0.000111514
YSR	0.000356046	LPT	0.00079223	ISL	0.000111264
VRV	0.000355612	APS	0.00078902	SLL	0.000111264
DRL	0.000354744	FWL	0.00078545	RLN	0.000111015
TGR	0.000353443	INM	0.00078438	ASS	0.000110516
SAP	0.00035301	HLN	0.00078224	RNF	0.000110266
PSG	0.000352576	SIR	0.00077867	YHA	0.000110266
ALG	0.000352142	EMP	0.0007776	YHV	0.000110266
VLG	0.000351275	GSS	0.00077653	GLT	0.000109518
VGS	0.000350841	TID	0.00077582	FCV	0.000109268
SVT	0.000350408	PES	0.00077546	PHA	0.000109268
PVS	0.000349974	SDQ	0.00077296	APT	0.000109019
GAS	0.00034954	EAN	0.00077046	NLK	0.000108769
VSV	0.00034954	IES	0.00076582	ENR	0.00010852
HRL	0.000349107	NPN	0.00076404	WPH	0.000108271
FSS	0.000348239	LPS	0.00076333	IKR	0.000107772
HRS	0.000347806	WVN	0.00076226	AQM	0.000107273
LVV	0.000347806	GCL	0.00076047	GHS	0.000106774
AGL	0.000347372	SSN	0.00076047	GKS	0.000106774
AVS	0.000347372	PGL	0.00074977	PFY	0.000106524
GSA	0.000346938	LAN	0.00074905	LSF	0.000106275
KRR	0.000346505	RWL	0.00074441	SMR	0.000106275
NRR	0.000346505	VGE	0.00074334	IMW	0.000106025
RAA	0.000346505	VTL	0.00074263	NWT	0.000105776
TVS	0.000346071	KKP	0.00073728	YTC	0.000105776
AAS	0.000345637	SYM	0.00073014	GSN	0.000105526
DSR	0.000345637	DPH	0.00072621	WRR	0.000105526
TGL	0.00034477	LRL	0.00072586	LHT	0.000105277
AGS	0.000344336	EQS	0.00072514	GWA	0.000105027
VGR	0.000344336	TGK	0.00071194	WGE	0.000104778
APT	0.000343903	GIG	0.00070873	STS	0.00010378
LHR	0.000343903	ICQ	0.00070694	GWL	0.000103032
LTV	0.000342602	FRD	0.0007048	LCH	0.000103032
RVT	0.000342168	LAF	0.00070337	ALS	0.000102283
TAL	0.000342168	EPI	0.00070302	FRG	0.000102283
SVP	0.000341734	DLW	0.0007023	GSL	0.000102034
TAS	0.000341734	LHG	0.00068946	SHS	0.000102034
ELR	0.000341301	FAR	0.00068553	LKW	0.000101535
LGV	0.000341301	FQI	0.00068553	FKR	0.000101036
GLV	0.000340867	PAA	0.00068125	IKT	0.000100287
TAR	0.000340867	FNR	0.00067518	WSD	0.000100287
LTG	0.000340433	TLS	0.00067375	YSY	9.97885E-05
SAT	0.000340433	WDM	0.00066947	FWF	9.82917E-05
KSR	0.000339566	PPA	0.00066911	RHA	9.80422E-05

LKL	0.000339566	YAY	0.0006684	LSV	9.75433E-05
LLM	0.000339132	LPE	0.00066091	WHQ	9.75433E-05
LLW	0.000339132	DRY	0.00066055	GGH	9.67948E-05
VAS	0.000339132	EYL	0.00066019	LNL	9.67948E-05
LLC	0.000338699	ASC	0.00065734	LLV	9.65454E-05
YRL	0.000338699	LQS	0.00065448	GDF	9.60464E-05
FSR	0.000338265	LRR	0.00065199	NKH	9.60464E-05
KRL	0.000338265	NAN	0.00064449	NNT	9.55475E-05
PVL	0.000338265	YQG	0.00064057	FHL	9.5298E-05
RGT	0.000338265	HQS	0.00063379	WHN	9.5298E-05
TRV	0.000338265	RCG	0.000632	VCW	9.50485E-05
KRS	0.000337398	NHV	0.00063093	AEQ	9.47991E-05
NRS	0.000337398	PIH	0.00062986	WDH	9.47991E-05
LDL	0.000336964	IMA	0.00062165	APF	9.43001E-05
LNR	0.000336964	FGP	0.00061309	FLG	9.43001E-05
GLG	0.00033653	LKL	0.00060452	GYF	9.43001E-05
KSS	0.00033653	PVC	0.00060381	LSC	9.43001E-05
YRR	0.00033653	DKI	0.00060202	QYA	9.43001E-05
GPS	0.000336097	FNI	0.00060167	RTT	9.43001E-05
GVL	0.000336097	LYM	0.0006006	FEQ	9.40507E-05
WLL	0.000335663	RAA	0.00059917	LTS	9.40507E-05
QLR	0.000334362	PMH	0.0005956	QNG	9.38012E-05
LGG	0.000333495	GEI	0.00059453	VKY	9.35517E-05
GVR	0.000333061	GKF	0.00058739	APC	9.33022E-05
PPV	0.000333061	GPM	0.00058597	GHF	9.33022E-05
GVS	0.000332193	AIS	0.00058275	YYP	9.33022E-05
AVL	0.00033176	ADG	0.0005799	FGV	9.30528E-05
LML	0.00033176	FLS	0.00057812	RLA	9.28033E-05
PSV	0.00033176	DES	0.00057633	VPY	9.28033E-05
VPP	0.000331326	TDR	0.00057098	GNY	9.23044E-05
DRS	0.000330892	PAS	0.00056884	SLG	9.23044E-05
GAR	0.000330892	RLN	0.00056562	SGQ	9.18054E-05
QLS	0.000330892	SVS	0.00056027	DYT	9.13065E-05
LKS	0.000330459	IGA	0.00055706	QNH	9.13065E-05
SAA	0.000330459	VRR	0.00055349	GLA	9.1057E-05
TRG	0.000330459	QNF	0.00054885	WNL	9.1057E-05
PTT	0.000329591	PAT	0.00054778	LQL	9.08075E-05
VRG	0.000329158	EVK	0.00054528	VTS	9.05581E-05
TTT	0.000328724	VLQ	0.00053957	FLV	9.03086E-05
GRV	0.00032829	RSQ	0.00053565	QNL	9.03086E-05
LNS	0.00032829	RKK	0.00053386	QNP	9.03086E-05
LMR	0.000327857	REE	0.00053279	LHW	9.00591E-05
ELS	0.000326556	WLL	0.00053137	WQC	9.00591E-05
VSG	0.000326556	EEN	0.00053065	LWH	8.98096E-05

ESR	0.000326122	CPR	0.00052601	WHR	8.98096E-05
TPT	0.000326122	IDV	0.00051781	HEH	8.93107E-05
ILL	0.000325688	RLI	0.00051245	RCN	8.93107E-05
LAG	0.000325688	VCA	0.00050853	RKR	8.93107E-05
PGA	0.000325688	VRW	0.00050567	VYP	8.93107E-05
KSL	0.000324821	TVC	0.0005021	ARS	8.90612E-05
RVA	0.000324387	EMS	0.00049961	EKH	8.88118E-05
SPG	0.000324387	PNL	0.00049854	FCP	8.88118E-05
SVA	0.000323086	ARK	0.00049604	LRN	8.88118E-05
ILR	0.000322653	ADP	0.00049497	LYW	8.80633E-05
LAV	0.000322653	CIK	0.00049033	RYS	8.78139E-05
RVP	0.000322653	PRT	0.00048997	SYA	8.78139E-05
DSS	0.000322219	LSR	0.00048961	GKY	8.75644E-05
LDR	0.000320484	YKG	0.00048961	GNH	8.73149E-05
LWR	0.000320484	PSS	0.00048854	FYP	8.70655E-05
LNL	0.000319617	ANI	0.00048819	FWI	8.6816E-05
RGP	0.000319617	SRT	0.00048676	YRL	8.6816E-05
SGT	0.000319617	SGP	0.00048462	YSF	8.6816E-05
YSS	0.000319617	NIP	0.00048283	PWL	8.65665E-05
GRG	0.000319183	FSL	0.00048034	WSL	8.65665E-05
LDS	0.000319183	PNY	0.00047712	KHA	8.58181E-05
PGP	0.000319183	QSI	0.00047641	PPE	8.58181E-05
FRS	0.00031875	TYP	0.00047463	LRQ	8.55686E-05
RTV	0.00031875	KND	0.00047106	LWC	8.55686E-05
WLR	0.00031875	LHF	0.00045857	ANH	8.53192E-05
ASV	0.000318316	KQT	0.00045785	RCW	8.53192E-05
GSV	0.000318316	WYT	0.00045678	LCT	8.45708E-05
LFL	0.000318316	LLV	0.000455	SHG	8.45708E-05
TTP	0.000318316	SPM	0.00045464	INS	8.40718E-05
LHL	0.000317882	MNS	0.00045143	QYR	8.40718E-05
QRR	0.000317882	NES	0.00045	FWA	8.38223E-05
TGS	0.000317882	RLR	0.00044965	WLH	8.38223E-05
DRR	0.000317449	RNK	0.00044715	WLL	8.38223E-05
HPR	0.000317449	YGT	0.00044715	RMS	8.30739E-05
LRM	0.000317449	ITT	0.00044643	RWN	8.30739E-05
LYL	0.000317449	ERF	0.00044179	LFL	8.28245E-05
SAV	0.000317015	SSF	0.00044072	VTD	8.2575E-05
WLS	0.000316581	PIG	0.00044037	HCW	8.23255E-05
SVV	0.000315714	VIS	0.00043965	NVH	8.23255E-05
TSV	0.00031528	PSL	0.00043466	RYP	8.23255E-05
ESL	0.000314847	VVE	0.00043252	FRW	8.13276E-05
SGA	0.000314847	RLV	0.00042895	QSD	8.13276E-05
LFS	0.000313979	IHA	0.00042788	VCL	8.13276E-05
LIR	0.000313979	RFW	0.00042716	PIH	8.10782E-05

CLL	0.000312678	SGL	0.00042538	DEK	8.08287E-05
LMS	0.000312678	LYE	0.00042395	FKC	8.08287E-05
LSW	0.000312678	RAN	0.00042288	LWT	8.08287E-05
STV	0.000311811	YPS	0.00042217	MNT	8.08287E-05
WSL	0.000311811	ALY	0.00042074	AEH	8.05792E-05
GTT	0.000311377	KRF	0.00041717	FCG	8.05792E-05
SGP	0.000311377	TLN	0.0004161	FCC	8.03297E-05
ILS	0.000310944	PPR	0.00041467	FST	8.03297E-05
QLL	0.000310944	SSG	0.00041182	SSD	8.03297E-05
GGS	0.00031051	GTT	0.00041075	SKC	7.98308E-05
LKR	0.00031051	AAL	0.00040789	IRL	7.95813E-05
LLQ	0.000310076	PTN	0.00040789	VTN	7.95813E-05
CSL	0.000309643	REA	0.00040718	WSC	7.95813E-05
SPV	0.000309643	MIG	0.00040682	LWG	7.93319E-05
APG	0.000309209	TKS	0.00040432	PTY	7.93319E-05
LCR	0.000309209	RYG	0.0004029	AVM	7.88329E-05
LRW	0.000309209	THM	0.00039968	CSL	7.88329E-05
SLM	0.000309209	QSH	0.00039897	FLS	7.88329E-05
SVG	0.000309209	MHC	0.00039861	DDQ	7.8334E-05
ATP	0.000308775	PLF	0.00039861	HHG	7.80845E-05
ESS	0.000308775	LSP	0.00039826	WKY	7.80845E-05
TSG	0.000307908	ESK	0.0003979	DYH	7.75856E-05
YRS	0.000307474	TRV	0.00039754	FRT	7.75856E-05
STG	0.00030704	RLL	0.00039362	LAL	7.75856E-05
ERL	0.000306607	DSF	0.0003929	PMY	7.75856E-05
RPV	0.000306607	LHS	0.00038862	RSA	7.75856E-05
SAG	0.000306607	VRL	0.00038612	GAH	7.73361E-05
LSM	0.000305739	LMW	0.00038541	ATH	7.70866E-05
HLT	0.000305306	MDL	0.00038541	LGN	7.70866E-05
HPP	0.000305306	VHP	0.00038505	NHG	7.70866E-05
SLC	0.000305306	YLR	0.0003822	FNL	7.68371E-05
TTA	0.000305306	NSV	0.00037899	NEP	7.68371E-05
FGL	0.000304872	TNQ	0.00037899	AQH	7.65877E-05
LLH	0.000304872	RYT	0.00037792	LEL	7.63382E-05
RGA	0.000304872	DCV	0.00037649	RSI	7.63382E-05
SMR	0.000304005	ELC	0.00037185	CYA	7.58393E-05
LQL	0.000303137	LER	0.00037185	YWR	7.58393E-05
RTG	0.000302704	GLR	0.00037114	ITT	7.55898E-05
CLR	0.00030227	WQS	0.00036757	AKQ	7.53403E-05
IRL	0.000301403	RLS	0.00036578	DAQ	7.53403E-05
CLS	0.000300969	NGR	0.00036364	WHP	7.50908E-05
LRC	0.000300969	PLG	0.00036221	LRG	7.45919E-05
QSR	0.000300969	KIP	0.00035972	GKR	7.43424E-05
ISL	0.000300535	TCM	0.00035865	YFC	7.43424E-05

LRQ	0.000300535	SGG	0.00035829	YTH	7.43424E-05
LSQ	0.000300535	GNL	0.00035615	GDH	7.4093E-05
QSS	0.000300535	WTL	0.00035615	DGQ	7.3594E-05
ERS	0.000300102	KGP	0.00035401	RNA	7.3594E-05
LSC	0.000300102	RSA	0.00035329	WKL	7.3594E-05
RAG	0.000299668	TWV	0.00035258	WLT	7.3594E-05
LYS	0.000298801	PLR	0.00035044	CSC	7.33445E-05
QRL	0.000298801	FAI	0.00034937	FQS	7.33445E-05
SDR	0.000298801	RSL	0.00034865	VNT	7.33445E-05
RDL	0.000298367	VAV	0.00034865	YPG	7.33445E-05
ATT	0.0002975	HGR	0.00034616	FGS	7.30951E-05
GPP	0.0002975	VCS	0.0003433	FWC	7.30951E-05
LFR	0.0002975	SKP	0.00034152	RYT	7.28456E-05
LLK	0.0002975	LTS	0.00034009	TPS	7.28456E-05
ASG	0.000297066	SSL	0.00033759	QQA	7.25961E-05
LLN	0.000296632	FKP	0.00033688	YCC	7.25961E-05
VTP	0.000296632	FTR	0.00033616	FYS	7.23467E-05
NPR	0.000295765	GAL	0.00033616	AEL	7.20972E-05
WRL	0.000295765	PTS	0.00033545	PID	7.20972E-05
AAP	0.000294898	GRS	0.00033509	SPL	7.20972E-05
LIL	0.000294898	LAP	0.00033438	NMT	7.18477E-05
PAT	0.000294898	QST	0.00033331	QIR	7.15982E-05
RLM	0.000294898	MCR	0.00033117	YGL	7.15982E-05
NTS	0.000294464	EGM	0.00033045	SGL	7.13488E-05
QRS	0.000294464	LCL	0.0003301	LCV	7.10993E-05
RVV	0.000294464	LNL	0.0003301	FGM	7.08498E-05
PAG	0.00029403	RAP	0.00032903	LRH	7.08498E-05
SDL	0.00029403	NHF	0.00032831	WEH	7.08498E-05
LYR	0.000293597	CES	0.00032474	FGP	7.06004E-05
RLC	0.000292729	NWA	0.00032474	GNR	7.06004E-05
RVG	0.000292729	VGA	0.00032296	GSS	7.06004E-05
GPA	0.000291862	CTN	0.0003226	WNR	7.06004E-05
SCL	0.000291862	GRA	0.00032225	AKN	7.03509E-05
HRP	0.000291428	NMM	0.00032082	QHS	7.01014E-05
LCS	0.000290995	YFC	0.00032082	AYP	6.98519E-05
NVL	0.000290995	YDY	0.0003201	LTT	6.98519E-05
QSL	0.000290561	SAK	0.00031939	PQD	6.98519E-05
SLW	0.000290561	SSS	0.00031796	RNC	6.98519E-05
ATA	0.000290127	WSF	0.00031689	FSP	6.96025E-05
KLT	0.000290127	GVK	0.00031654	LSH	6.96025E-05
SIL	0.000290127	LRT	0.00031618	FYR	6.9353E-05
SNL	0.000290127	AIT	0.00031297	LWK	6.9353E-05
RNR	0.000289694	TSL	0.00031225	CHH	6.91035E-05
RNS	0.000289694	RYA	0.0003119	ILL	6.91035E-05

VTT	0.000289694	LAG	0.00031118	RYE	6.88541E-05
HST	0.00028926	SSC	0.00031047	RTA	6.86046E-05
PPQ	0.000288826	SLL	0.00030833	WMC	6.86046E-05
RAV	0.000288826	YRN	0.00030547	ILM	6.83551E-05
SKL	0.000288826	SSA	0.00030476	GYS	6.81057E-05
HPL	0.000287525	RVP	0.0003019	RFT	6.81057E-05
LIS	0.000287525	AGI	0.00030083	WKF	6.81057E-05
MLR	0.000287092	RPT	0.00030083	ARL	6.76067E-05
ERR	0.000286658	KPA	0.00030012	NER	6.76067E-05
IRR	0.000286658	VIP	0.00029905	SKH	6.76067E-05
RGG	0.000286658	LRA	0.00029834	VKL	6.76067E-05
LES	0.000286224	SFK	0.00029762	NEL	6.73572E-05
PTG	0.000286224	VMS	0.00029727	WYA	6.73572E-05
SWL	0.000286224	PPK	0.00029691	GIF	6.71078E-05
WSS	0.000286224	ISA	0.00029584	IST	6.71078E-05
RNL	0.00028579	PFG	0.00029512	LWI	6.71078E-05
SDS	0.00028579	HPV	0.00029477	WHY	6.71078E-05
YLT	0.000285357	ALV	0.00029441	WWR	6.71078E-05
GSG	0.000284923	FRP	0.00029263	FNS	6.68583E-05
RSM	0.000284923	NTK	0.00029263	MLT	6.68583E-05
DVL	0.000284056	FRL	0.00028977	YDG	6.66088E-05
WRR	0.000284056	IPP	0.0002887	QHG	6.58604E-05
LLE	0.000283622	GGR	0.00028834	YAC	6.58604E-05
RKR	0.000283622	RSR	0.00028834	AAS	6.56109E-05
SHL	0.000283622	QRA	0.00028763	HHA	6.56109E-05
HLP	0.000282755	STV	0.00028585	PVY	6.56109E-05
RKL	0.000282755	LQN	0.00028549	EEH	6.53615E-05
RMS	0.000282755	NAV	0.00028478	NEY	6.53615E-05
APV	0.000282321	PGA	0.00028406	GEP	6.5112E-05
FPL	0.000282321	HSF	0.0002837	PSD	6.5112E-05
RHR	0.000282321	AQT	0.00028049	DYS	6.46131E-05
SNR	0.000282321	QLL	0.00027907	ESD	6.46131E-05
RDR	0.000281887	CDL	0.00027871	FPS	6.46131E-05
WRS	0.000281887	PLH	0.00027835	LKS	6.46131E-05
GTA	0.000281454	VPA	0.00027478	YYR	6.43636E-05
ISR	0.000281454	DPL	0.00027371	APL	6.41141E-05
LSK	0.00028102	KEW	0.00027193	PIS	6.41141E-05
ALM	0.000280586	FYK	0.00027157	YHE	6.38646E-05
SHR	0.000280586	ISS	0.00027157	AKY	6.36152E-05
DLP	0.000280153	LWL	0.00027157	HYA	6.36152E-05
CRL	0.000279719	PDE	0.00027157	VTL	6.36152E-05
SCR	0.000279285	GSP	0.00027121	RTF	6.33657E-05
DTL	0.000278852	RFL	0.00027121	MHS	6.31162E-05
RDS	0.000278852	LSD	0.00026872	SRM	6.31162E-05

SKR	0.000278852	LAS	0.00026836	ATT	6.28668E-05
RLK	0.000278418	VKL	0.00026836	FEH	6.28668E-05
RRM	0.000277984	GMD	0.000268	KSD	6.28668E-05
SIR	0.000277984	LKS	0.00026693	RSL	6.28668E-05
NTR	0.000277551	AHH	0.00026658	SLR	6.28668E-05
RFL	0.000277551	KDT	0.00026658	AKR	6.26173E-05
WSR	0.000277551	HAS	0.0002655	GNA	6.26173E-05
DLT	0.000277117	AKE	0.00026515	INN	6.26173E-05
LEL	0.000277117	CGM	0.00026515	LRC	6.26173E-05
NTL	0.000277117	RLP	0.00026372	WDW	6.26173E-05
RQS	0.000277117	RCS	0.00026194	LYR	6.23678E-05
MLS	0.000276683	NSR	0.00026122	YCL	6.23678E-05
RKS	0.000276683	KLA	0.00026087	PPV	6.21183E-05
SKS	0.000276683	FKH	0.00025944	VRR	6.21183E-05
SML	0.000276683	LFT	0.00025872	LCD	6.18689E-05
SFS	0.00027625	ESL	0.00025837	DEP	6.16194E-05
SFL	0.000275816	PSP	0.0002573	FLP	6.16194E-05
SMS	0.000275816	QPF	0.00025623	FTS	6.16194E-05
RFS	0.000275382	WVV	0.00025587	IMK	6.16194E-05
YPR	0.000275382	LML	0.00025551	VLR	6.16194E-05
NSP	0.000274949	RNQ	0.0002548	WSH	6.13699E-05
SSM	0.000274949	FSF	0.00025337	YRG	6.13699E-05
HLA	0.000274515	WSW	0.00025266	PKH	6.11205E-05
NLA	0.000274515	AFY	0.00025159	PPN	6.11205E-05
QPR	0.000274515	PGI	0.00025123	GYL	6.0871E-05
RIL	0.000274515	LLR	0.0002498	CRL	6.06215E-05
VPT	0.000274515	NKL	0.00024838	LYP	6.06215E-05
CRR	0.000274081	PAR	0.00024802	PLT	6.0372E-05
RLW	0.000273648	MTI	0.00024766	CGL	6.01226E-05
VTA	0.000273648	SRV	0.00024588	DER	6.01226E-05
SRC	0.00027278	TLL	0.00024552	DYL	6.01226E-05
SRQ	0.00027278	SPR	0.00024374	GTL	6.01226E-05
RLH	0.000272347	SMF	0.00024302	RAS	6.01226E-05
SEL	0.000272347	AAH	0.00024231	YTS	6.01226E-05
SYL	0.000272347	YIP	0.00024124	DHG	5.98731E-05
KTR	0.000271913	SVT	0.00024052	SPT	5.98731E-05
LQS	0.000271913	CKS	0.00023945	SEH	5.96236E-05
RMR	0.000271913	LLS	0.00023945	FMM	5.93742E-05
VAT	0.000271913	TRF	0.00023874	REH	5.91247E-05
LQR	0.000271479	TPT	0.00023838	WNS	5.91247E-05
LRK	0.000271479	DLM	0.00023731	FSQ	5.88752E-05
MLL	0.000271479	SDP	0.0002366	PLD	5.88752E-05
SHS	0.000271046	VLH	0.0002366	PSY	5.88752E-05
CSR	0.000270612	ARA	0.00023624	LTR	5.86257E-05

HPS	0.000270612	LFR	0.00023517	WKH	5.86257E-05
NPL	0.000270612	LPR	0.00023446	GTH	5.83763E-05
SLH	0.000270612	VHF	0.00023446	KFT	5.83763E-05
LWS	0.000270178	WLV	0.00023446	NLP	5.83763E-05
RGV	0.000270178	GLL	0.0002341	YLG	5.83763E-05
AAT	0.000269745	SRN	0.0002341	LRT	5.81268E-05
FTL	0.000269745	VDM	0.0002341	PPH	5.81268E-05
IRS	0.000269745	LPP	0.00023374	GKG	5.78773E-05
LRH	0.000269745	NPM	0.00023267	PLL	5.78773E-05
SRW	0.000269745	RMR	0.00023267	PNY	5.78773E-05
TAT	0.000269745	WRT	0.00023232	FCM	5.76279E-05
HTL	0.000269311	LYD	0.0002316	FGH	5.76279E-05
SLQ	0.000269311	GRL	0.00023125	GRL	5.76279E-05
ISS	0.000268444	PST	0.00023089	IEH	5.76279E-05
KTL	0.000268444	RLY	0.00023089	IML	5.76279E-05
RHL	0.00026801	TMS	0.00023053	LLT	5.76279E-05
RIS	0.00026801	VWL	0.00023053	SKT	5.76279E-05
RLQ	0.000267576	FRR	0.00022982	AKP	5.73784E-05
SGG	0.000267576	ALM	0.00022875	IGL	5.73784E-05
SNS	0.000267576	AMV	0.00022875	PPY	5.73784E-05
DTS	0.000267143	TLG	0.00022839	SDW	5.73784E-05
HSP	0.000267143	KWH	0.00022803	TPD	5.73784E-05
RQL	0.000267143	YTL	0.00022768	TTS	5.73784E-05
SSW	0.000267143	EFS	0.00022732	PTT	5.71289E-05
LLD	0.000266709	SWR	0.00022625	RYG	5.68794E-05
SRM	0.000266709	HDL	0.00022554	RRS	5.663E-05
CRS	0.000266275	RTS	0.00022518	NVT	5.63805E-05
GAT	0.000266275	TRR	0.00022447	WHL	5.63805E-05
SGV	0.000266275	YDS	0.00022447	LFG	5.6131E-05
SYS	0.000266275	RNR	0.00022375	VSF	5.6131E-05
AAA	0.000265842	SGT	0.00022375	MMS	5.58816E-05
DPL	0.000265842	WSL	0.0002234	RNL	5.58816E-05
FPR	0.000265842	YTP	0.0002234	RNS	5.58816E-05
REL	0.000265842	WGG	0.00022268	SRR	5.58816E-05
RRC	0.000265842	LGD	0.00022232	AWL	5.56321E-05
LNT	0.000265408	RPP	0.00022232	FNR	5.56321E-05
MSS	0.000265408	HSR	0.00022125	GST	5.56321E-05
SLE	0.000264974	NRP	0.00022125	LCK	5.56321E-05
SFR	0.000264541	VST	0.00022125	LST	5.56321E-05
SLN	0.000264541	FPR	0.00022054	QHP	5.56321E-05
SYR	0.000264541	GVY	0.00021983	LQP	5.53826E-05
VVT	0.000264541	LSM	0.00021947	LSY	5.53826E-05
CSS	0.000264107	YTG	0.00021911	SSS	5.53826E-05
LLI	0.000263673	STP	0.00021876	GHA	5.51331E-05

RIR	0.000263673	FLT	0.00021769	LSM	5.51331E-05
VVA	0.000263673	VSS	0.00021769	PLR	5.51331E-05
DRP	0.000263239	ELP	0.00021733	YKY	5.51331E-05
KST	0.000263239	FRW	0.0002159	WHD	5.48837E-05
LER	0.000263239	GLP	0.0002159	WTY	5.48837E-05
LSN	0.000263239	KGW	0.0002159	GLL	5.46342E-05
RML	0.000263239	LTK	0.00021554	NLA	5.46342E-05
RRW	0.000262806	NPP	0.00021554	TEH	5.46342E-05
SIS	0.000262806	RDA	0.00021483	FGW	5.43847E-05
RCS	0.000262372	RGP	0.00021447	FTF	5.43847E-05
HTR	0.000261938	TRY	0.00021447	PTD	5.41353E-05
AVT	0.000261505	KRA	0.00021412	RYV	5.41353E-05
KPR	0.000261505	FSC	0.00021376	DYR	5.38858E-05
NPS	0.000261505	RVW	0.00021376	LSP	5.38858E-05
YVR	0.000261505	EGS	0.0002134	ALK	5.36363E-05
GPT	0.000261071	YNP	0.00021305	GEN	5.36363E-05
HRT	0.000261071	NGD	0.00021269	RWT	5.36363E-05
YTR	0.000261071	RVS	0.00021269	GLS	5.33868E-05
DRT	0.000260637	YSR	0.00021269	INP	5.33868E-05
FLT	0.000260637	RKN	0.00021233	VKH	5.33868E-05
TPV	0.000260637	GPW	0.00021198	FHV	5.31374E-05
DTR	0.000260204	VHM	0.00021198	GQH	5.31374E-05
FGR	0.000260204	IRY	0.00021162	LCQ	5.31374E-05
KLP	0.000260204	RWE	0.00021162	RMR	5.31374E-05
NAS	0.00025977	GPF	0.00021126	NWL	5.28879E-05
NRT	0.00025977	HFW	0.00021126	RLP	5.28879E-05
YST	0.00025977	VVM	0.00021126	RLR	5.28879E-05
AGP	0.000259336	DVR	0.0002109	WNG	5.28879E-05
FLP	0.000259336	NSS	0.0002109	ARH	5.26384E-05
GTP	0.000259336	SRS	0.0002109	ENF	5.26384E-05
RYS	0.000259336	TRC	0.0002109	GTC	5.26384E-05
SLI	0.000259336	ASL	0.00021019	ILS	5.26384E-05
DST	0.000258903	GPT	0.00021019	ITS	5.26384E-05
LSH	0.000258903	GSE	0.00021019	LFW	5.26384E-05
MRL	0.000258903	RIQ	0.00020983	LHQ	5.26384E-05
NST	0.000258903	RPK	0.00020912	VWW	5.26384E-05
TVT	0.000258903	PDV	0.00020876	CHG	5.2389E-05
VPA	0.000258903	RPL	0.00020876	NLS	5.2389E-05
VVV	0.000258469	TTT	0.00020876	QDW	5.2389E-05
DLA	0.000258035	SSM	0.00020841	RPH	5.2389E-05
LRE	0.000258035	KPG	0.00020769	SPD	5.2389E-05
RSQ	0.000258035	PRS	0.00020734	VTC	5.2389E-05
RYL	0.000258035	VYH	0.00020734	NED	5.21395E-05
HVL	0.000257602	YLA	0.00020734	SST	5.21395E-05

LRN	0.000257168	SVL	0.00020662	CWR	5.189E-05
RCR	0.000257168	DFG	0.00020627	RGH	5.189E-05
RLE	0.000257168	SRW	0.00020627	RWL	5.189E-05
RSW	0.000256734	KSA	0.0002052	PLS	5.16405E-05
FRT	0.000256301	DMY	0.00020484	WSS	5.16405E-05
RRD	0.000256301	DPA	0.00020484	FQR	5.13911E-05
RSN	0.000256301	LSQ	0.00020484	FTR	5.13911E-05
SER	0.000256301	SFC	0.00020484	LRM	5.13911E-05
SQL	0.000255867	FSY	0.00020448	VAY	5.13911E-05
SLY	0.000255433	AML	0.00020377	VRY	5.13911E-05
SSQ	0.000255433	FGL	0.00020341	VSY	5.13911E-05
SWR	0.000255433	GKR	0.00020341	GLV	5.11416E-05
DPR	0.000255	GRT	0.00020341	GPY	5.11416E-05
SSC	0.000254566	KFT	0.00020341	GTN	5.11416E-05
SLK	0.000254132	GRR	0.0002027	LCY	5.11416E-05
TTG	0.000254132	SLP	0.00020234	MCN	5.11416E-05
YTS	0.000254132	YLM	0.00020234	MWL	5.11416E-05
PRQ	0.000253699	PCY	0.00020198	TNT	5.11416E-05
RHS	0.000253699	SGA	0.00020198	VTF	5.11416E-05
YVL	0.000253699	HPG	0.00020163	YTD	5.11416E-05
FSP	0.000253265	LHT	0.00020163	AKL	5.08921E-05
NAL	0.000253265	RFQ	0.00020163	FWN	5.08921E-05
RFR	0.000253265	LWT	0.00020127	GLI	5.08921E-05
FST	0.000252831	SQR	0.00020056	LLW	5.08921E-05
HVR	0.000252831	SRG	0.0002002	VGQ	5.08921E-05
NAR	0.000252831	WFA	0.0002002	DKQ	5.06427E-05
RSC	0.000252831	ALL	0.00019984	LDL	5.06427E-05
SSK	0.000252831	SEL	0.00019984	NYP	5.06427E-05
YPL	0.000252831	LTA	0.00019949	PIN	5.06427E-05
AGA	0.000251964	PNR	0.00019949	FHR	5.03932E-05
LSE	0.000251964	ALT	0.00019913	LCP	5.03932E-05
LRD	0.00025153	ETG	0.00019913	QSC	5.03932E-05
RES	0.00025153	FYP	0.00019877	YLM	5.03932E-05
SLD	0.00025153	GHG	0.00019877	LSQ	5.01437E-05
SLF	0.00025153	LRV	0.00019806	YNR	4.96448E-05
DPS	0.000251097	YIA	0.00019734	QCN	4.93953E-05
NVR	0.000251097	LKF	0.00019699	VNL	4.93953E-05
VVP	0.000251097	NKY	0.00019699	GTD	4.91458E-05
NRA	0.000250663	SNR	0.00019699	KDW	4.91458E-05
SES	0.000250663	YFT	0.00019663	FPR	4.88964E-05
VGP	0.000250663	TRM	0.00019627	FYA	4.88964E-05
LLF	0.000250229	TYC	0.00019627	VAL	4.88964E-05
SRE	0.000250229	HNV	0.00019592	VPL	4.88964E-05
MSL	0.000249796	LNP	0.00019592	LWF	4.86469E-05

NVS	0.000249362	RLG	0.00019556	RNH	4.86469E-05
RCL	0.000249362	PVL	0.0001952	VYL	4.86469E-05
RSK	0.000249362	EEL	0.00019485	FMS	4.83974E-05
YLA	0.000249362	VCE	0.00019449	SHH	4.83974E-05
DVR	0.000248928	VKP	0.00019449	IIS	4.8148E-05
DVS	0.000248928	VMR	0.00019449	LRF	4.8148E-05
EPR	0.000248928	RSI	0.00019378	YPY	4.8148E-05
HTS	0.000248928	SYC	0.00019378	DYA	4.78985E-05
SQR	0.000248928	REL	0.00019342	LWA	4.78985E-05
YSP	0.000248928	STL	0.00019342	PTS	4.78985E-05
FVR	0.000248495	AAD	0.00019306	STR	4.78985E-05
KRA	0.000248495	GHL	0.00019306	FRH	4.7649E-05
LLY	0.000248495	YFF	0.00019235	LMS	4.7649E-05
PPH	0.000248495	LMN	0.00019163	VSS	4.7649E-05
RRK	0.000248495	EPL	0.00019092	VWM	4.7649E-05
TPG	0.000248495	TDT	0.00019021	AKD	4.73995E-05
YLP	0.000248495	GKT	0.00018985	SCL	4.73995E-05
AVA	0.000247627	HNT	0.00018949	YLR	4.73995E-05
FTR	0.000247627	KLR	0.00018914	GKD	4.71501E-05
LMT	0.000247627	CVR	0.00018842	LGP	4.71501E-05
SCS	0.000247627	TLP	0.00018842	PRL	4.71501E-05
TAP	0.000247627	TQT	0.00018842	VRM	4.71501E-05
VGT	0.000247627	TTI	0.00018842	GLR	4.69006E-05
EPL	0.000247194	FGS	0.00018807	LNC	4.69006E-05
GVT	0.000247194	FQR	0.00018807	WHA	4.69006E-05
DSP	0.00024676	GVC	0.00018807	RTR	4.66511E-05
HAS	0.00024676	LLY	0.00018807	NMS	4.64017E-05
HRA	0.00024676	PKP	0.00018807	SNT	4.64017E-05
NLP	0.00024676	PVS	0.00018807	YLL	4.64017E-05
PVP	0.00024676	RRP	0.00018807	GDL	4.61522E-05
VAP	0.00024676	CTT	0.00018771	GED	4.61522E-05
HVS	0.000246326	HRM	0.00018771	GNP	4.61522E-05
QPL	0.000246326	LLE	0.00018771	IWT	4.61522E-05
TAA	0.000246326	CSA	0.00018628	FYV	4.59027E-05
KLA	0.000245893	DRG	0.00018628	PTR	4.59027E-05
LSF	0.000245893	LLD	0.00018557	RSC	4.59027E-05
MRS	0.000245893	YLY	0.00018557	DVQ	4.54038E-05
TVP	0.000245893	KAN	0.00018485	EDW	4.54038E-05
AGT	0.000245459	SVG	0.00018236	GGL	4.54038E-05
DAS	0.000245459	TAH	0.00018236	LHG	4.54038E-05
FRP	0.000245459	LIT	0.000182	LTC	4.54038E-05
KRT	0.000245459	GLQ	0.00018164	SSR	4.54038E-05
PTV	0.000245459	RPS	0.00018164	WPS	4.54038E-05
RYR	0.000245459	HTK	0.00018129	YEL	4.54038E-05

HLV	0.000245025	LRY	0.00018129	YST	4.54038E-05
RQR	0.000245025	SSE	0.00018129	PRR	4.51543E-05
RLF	0.000244592	DLI	0.00018057	PHR	4.46554E-05
VTV	0.000244592	HAR	0.00018021	RFG	4.46554E-05
ERT	0.000244158	LRS	0.00018021	VHR	4.46554E-05
PGT	0.000244158	RRN	0.00018021	YGQ	4.46554E-05
QPP	0.000244158	RWS	0.00018021	DTY	4.44059E-05
YAL	0.000243724	SYN	0.00018021	NTY	4.44059E-05
FLA	0.000243291	EDA	0.00017986	QHE	4.44059E-05
WLT	0.000242857	CWR	0.0001795	SLS	4.44059E-05
GAA	0.000242423	GSA	0.0001795	VMY	4.44059E-05
KVL	0.000242423	DRV	0.00017914	WLW	4.44059E-05
RRQ	0.000242423	RGL	0.00017914	FER	4.41564E-05
TGP	0.000242423	FRC	0.00017879	FSD	4.41564E-05
VVG	0.000242423	VRA	0.00017843	FTT	4.41564E-05
YTL	0.000242423	LEM	0.00017807	PPR	4.41564E-05
SSH	0.00024199	LPC	0.00017772	TTT	4.41564E-05
TTV	0.00024199	LEC	0.00017736	INA	4.39069E-05
VPG	0.00024199	TTS	0.00017736	LML	4.39069E-05
ELT	0.000241556	YFV	0.000177	LSG	4.39069E-05
QRP	0.000241556	RTQ	0.00017665	VRS	4.39069E-05
RRE	0.000241556	LGC	0.00017593	LDR	4.36575E-05
HSA	0.000241122	SVR	0.00017593	RTH	4.36575E-05
KPL	0.000241122	GTA	0.00017558	SPP	4.36575E-05
TLM	0.000241122	ILG	0.00017558	FFR	4.3408E-05
DLV	0.000240688	TDD	0.00017558	GER	4.3408E-05
GVA	0.000240255	DAP	0.00017522	GNT	4.3408E-05
LSD	0.000240255	WLQ	0.00017522	VMS	4.3408E-05
TGT	0.000240255	LLL	0.00017486	VNS	4.3408E-05
YVS	0.000240255	LSH	0.00017486	GEY	4.31585E-05
FPS	0.000239821	LVM	0.00017486	HYP	4.31585E-05
GAP	0.000239821	RVA	0.00017486	VEF	4.31585E-05
LKT	0.000239821	NKS	0.00017451	PIC	4.29091E-05
MSR	0.000239821	NRL	0.00017451	VWP	4.29091E-05
PHR	0.000239821	NRM	0.00017451	GSD	4.26596E-05
VGV	0.000239821	PPV	0.00017451	NSS	4.26596E-05
VPV	0.000239387	IMS	0.00017415	PLG	4.26596E-05
RSH	0.000238954	LSV	0.00017415	STY	4.26596E-05
VWL	0.000238954	YTS	0.00017415	FSY	4.24101E-05
YPS	0.000238954	GFS	0.00017379	LGV	4.24101E-05
DAR	0.00023852	NVV	0.00017379	PSS	4.24101E-05
ETR	0.00023852	SST	0.00017379	FCT	4.21606E-05
RRN	0.00023852	LTM	0.00017343	LNP	4.21606E-05
NRP	0.000238086	LAH	0.00017308	PPL	4.21606E-05

RLN	0.000238086	MPP	0.00017308	TLR	4.21606E-05
LHT	0.000237653	PCT	0.00017308	FPF	4.19112E-05
PGG	0.000237653	VLC	0.00017272	FQV	4.19112E-05
QPS	0.000237653	PGG	0.00017236	PHS	4.19112E-05
EST	0.000237219	VIV	0.00017236	PMR	4.19112E-05
FVL	0.000237219	IMD	0.00017201	SRT	4.19112E-05
LRF	0.000237219	ALR	0.00017129	WSF	4.19112E-05
TGA	0.000237219	LKK	0.00017129	RSR	4.16617E-05
DAL	0.000236785	MWW	0.00017129	FKS	4.14122E-05
LYT	0.000236785	RSY	0.00017129	LNW	4.14122E-05
MRR	0.000236785	VTS	0.00017129	QIY	4.14122E-05
RER	0.000236785	FNT	0.00017058	WSG	4.14122E-05
SRN	0.000236785	KGN	0.00017022	KST	4.11628E-05
WLG	0.000236785	WLP	0.00017022	GKP	4.09133E-05
AVP	0.000236352	DIP	0.00016987	GVH	4.09133E-05
NLV	0.000236352	KLP	0.00016987	IWM	4.09133E-05
QTR	0.000236352	LLH	0.00016987	LSI	4.09133E-05
SQS	0.000236352	WTC	0.00016987	LYT	4.09133E-05
PLQ	0.000235918	YSG	0.00016987	PKR	4.09133E-05
ETS	0.000235484	FVS	0.00016951	QRA	4.09133E-05
LNA	0.000235484	MLA	0.00016951	VWV	4.09133E-05
YAR	0.000235484	CLS	0.00016915	WVH	4.09133E-05
GGA	0.000234617	CLT	0.00016915	KDH	4.06638E-05
PRW	0.000234617	APG	0.0001688	LCA	4.06638E-05
GPG	0.000234183	RMA	0.0001688	LRA	4.06638E-05
PPW	0.000234183	DVS	0.00016844	PPP	4.06638E-05
VAV	0.000234183	THA	0.00016844	VYS	4.06638E-05
HAR	0.00023375	YAS	0.00016844	YCR	4.06638E-05
HLG	0.00023375	KRH	0.00016808	ELT	4.04143E-05
LNP	0.00023375	RNT	0.00016808	FSI	4.04143E-05
NSA	0.00023375	TMN	0.00016772	GYH	4.04143E-05
RWR	0.00023375	ASF	0.00016737	GHL	4.01649E-05
ILT	0.000233316	DTM	0.00016737	HEQ	4.01649E-05
KPS	0.000233316	TPG	0.00016701	PST	4.01649E-05
YRT	0.000233316	VDG	0.00016701	GKT	3.99154E-05
ATG	0.000232882	DTL	0.00016665	LCM	3.99154E-05
PVA	0.000232882	GPK	0.00016665	RIG	3.99154E-05
RLD	0.000232882	MGT	0.00016665	FSG	3.96659E-05
LCT	0.000232449	QIT	0.00016665	FVL	3.96659E-05
LRY	0.000232449	AMR	0.0001663	KKR	3.96659E-05
RWL	0.000232449	RIL	0.00016594	PSR	3.96659E-05
RWS	0.000232015	GAK	0.00016558	TKH	3.96659E-05
SRK	0.000232015	IAP	0.00016558	WDQ	3.96659E-05
TLC	0.000232015	KSV	0.00016558	LHR	3.94165E-05

VLM	0.000232015	TRP	0.00016558	LPR	3.94165E-05
KVR	0.000231581	MSL	0.00016523	QSS	3.9167E-05
NGL	0.000231581	KRD	0.00016487	VQS	3.9167E-05
RSD	0.000231581	RRS	0.00016487	WWP	3.9167E-05
AAG	0.000231148	YAK	0.00016487	ESC	3.89175E-05
QTS	0.000231148	KPN	0.00016451	FYG	3.89175E-05
SWS	0.000231148	YRF	0.00016451	LNF	3.89175E-05
AGG	0.000230714	YVT	0.00016451	LSA	3.89175E-05
QTL	0.000230714	FLV	0.00016416	NKR	3.89175E-05
LRI	0.00023028	GIV	0.00016416	NLL	3.89175E-05
QST	0.00023028	IMG	0.00016416	QRR	3.89175E-05
VGG	0.00023028	LYK	0.00016416	KKH	3.8668E-05
HAL	0.000229847	PSG	0.00016416	LSK	3.8668E-05
LPC	0.000229847	AHT	0.0001638	SMS	3.8668E-05
LPQ	0.000229847	RIE	0.0001638	VDL	3.8668E-05
RLI	0.000229847	HHS	0.00016344	WAH	3.8668E-05
FAL	0.000229413	IPV	0.00016344	IHT	3.84186E-05
GGP	0.000229413	VSA	0.00016344	RLI	3.84186E-05
HGR	0.000229413	GAY	0.00016237	RPT	3.84186E-05
KAL	0.000229413	DCI	0.00016201	YTN	3.84186E-05
YRP	0.000229413	PSR	0.00016201	ANF	3.81691E-05
DSA	0.000228979	LFL	0.00016166	IIT	3.81691E-05
LTW	0.000228979	LRH	0.00016166	RKA	3.81691E-05
RRH	0.000228979	LTC	0.00016166	RKG	3.81691E-05
KAR	0.000228546	WGL	0.0001613	WQS	3.81691E-05
LSI	0.000228546	LHL	0.00016023	WWH	3.81691E-05
YRA	0.000228546	LPG	0.00016023	FQG	3.79196E-05
DSV	0.000228112	NAS	0.00016023	FRC	3.79196E-05
FTS	0.000228112	QNR	0.00016023	GSC	3.79196E-05
VDL	0.000228112	AIQ	0.00015987	LLM	3.79196E-05
VGA	0.000228112	VIL	0.00015987	PCR	3.79196E-05
FLV	0.000227678	NST	0.00015952	RKL	3.79196E-05
FRA	0.000227678	HSH	0.00015916	TIY	3.79196E-05
KLV	0.000227678	WLW	0.00015916	VGR	3.79196E-05
RNT	0.000227678	HKV	0.0001588	VWA	3.79196E-05
TNR	0.000227678	HLH	0.0001588	WCL	3.79196E-05
DGL	0.000227245	TYE	0.0001588	WMG	3.79196E-05
FAS	0.000227245	YGK	0.0001588	WVR	3.79196E-05
KSP	0.000227245	PEI	0.00015845	APH	3.76702E-05
GGT	0.000226811	PYP	0.00015845	KLR	3.76702E-05
KAS	0.000226811	EDE	0.00015809	PSL	3.76702E-05
PHL	0.000226811	VNS	0.00015809	DCP	3.74207E-05
VTG	0.000226811	RLK	0.00015773	GTA	3.74207E-05
PVT	0.000226377	WMC	0.00015773	APD	3.71712E-05

QAL	0.000226377	GCA	0.00015738	INI	3.71712E-05
FWR	0.000225944	ILR	0.00015738	LFR	3.71712E-05
HTP	0.000225944	CVL	0.00015702	LLP	3.71712E-05
LHP	0.000225944	ESD	0.00015702	PPT	3.71712E-05
PPC	0.000225944	FTD	0.00015702	QLT	3.71712E-05
SNA	0.000225944	NDA	0.00015702	TST	3.71712E-05
NGR	0.00022551	RWH	0.00015702	DSP	3.69217E-05
THS	0.00022551	SNP	0.00015702	FPV	3.69217E-05
ANL	0.000225076	TTR	0.00015702	FSH	3.69217E-05
GTV	0.000225076	NRN	0.00015631	FVR	3.69217E-05
LYA	0.000225076	PLT	0.00015631	PNS	3.69217E-05
HRV	0.000224643	TLQ	0.00015631	QHV	3.69217E-05
KVS	0.000224643	TNT	0.00015631	CEH	3.66723E-05
PAV	0.000224643	WYL	0.00015631	DWL	3.66723E-05
SSE	0.000224643	HMD	0.00015595	FHA	3.66723E-05
VMR	0.000224209	LYL	0.00015595	FHW	3.66723E-05
VSM	0.000223775	RRQ	0.00015595	STK	3.66723E-05
YGR	0.000223775	ASK	0.00015559	VEH	3.66723E-05
FVS	0.000223342	RWP	0.00015559	VWS	3.66723E-05
KRP	0.000223342	TAG	0.00015559	YCW	3.66723E-05
LIT	0.000223342	WNR	0.00015559	FHP	3.64228E-05
SIT	0.000223342	EKL	0.00015523	NQH	3.64228E-05
TLW	0.000223342	PWG	0.00015523	WER	3.64228E-05
VAG	0.000223342	AKP	0.00015488	GYT	3.61733E-05
VCL	0.000223342	INS	0.00015488	FGG	3.59239E-05
EPS	0.000222908	LTG	0.00015488	FRF	3.56744E-05
LFT	0.000222908	VWF	0.00015488	GAY	3.56744E-05
LHA	0.000222908	DIA	0.00015452	PSC	3.56744E-05
PRC	0.000222908	GWP	0.00015452	ETS	3.54249E-05
QLT	0.000222908	TVR	0.00015452	FCQ	3.54249E-05
SSN	0.000222908	RPY	0.00015416	FIR	3.54249E-05
VNL	0.000222908	WTS	0.00015416	RDA	3.54249E-05
WTR	0.000222908	VQR	0.00015381	RGR	3.54249E-05
GVP	0.000222474	AMH	0.00015345	WSR	3.54249E-05
HGL	0.000222474	CYQ	0.00015345	ETD	3.51754E-05
PQR	0.000222474	NIT	0.00015345	FDL	3.51754E-05
KTS	0.000222041	PDY	0.00015345	FLQ	3.51754E-05
VCS	0.000222041	TCA	0.00015345	FMW	3.51754E-05
KSA	0.000221607	TTW	0.00015345	GKW	3.51754E-05
NLG	0.000221607	GTV	0.00015309	KMR	3.51754E-05
TVA	0.000221607	LYP	0.00015309	RAH	3.51754E-05
ELA	0.000221173	SRL	0.00015309	SKR	3.51754E-05
PMR	0.000221173	THC	0.00015309	WRS	3.51754E-05
QLA	0.000221173	LIF	0.00015274	FLW	3.4926E-05

RKT	0.000221173	REH	0.00015274	GEF	3.4926E-05
VAA	0.000221173	LKG	0.00015238	GSY	3.4926E-05
VLK	0.000221173	TKQ	0.00015238	KLG	3.4926E-05
VML	0.000221173	TMD	0.00015238	RNT	3.4926E-05
YLG	0.000221173	MSI	0.00015202	RSP	3.4926E-05
YLV	0.000221173	DSC	0.00015167	YYW	3.4926E-05
DLG	0.00022074	LTV	0.00015167	FSN	3.46765E-05
EVL	0.00022074	YVW	0.00015167	GPA	3.46765E-05
PLH	0.00022074	ACR	0.00015131	LYN	3.46765E-05
FSV	0.000220306	GWV	0.00015131	QDA	3.46765E-05
GPV	0.000220306	LGL	0.00015095	RCA	3.46765E-05
PHS	0.000220306	NGC	0.0001506	WFR	3.46765E-05
PQP	0.000220306	NLS	0.0001506	GSV	3.4427E-05
HGS	0.000219872	ESG	0.00015024	LTF	3.4427E-05
FLG	0.000219439	STT	0.00015024	PCC	3.4427E-05
NSV	0.000219439	TTG	0.00015024	PKC	3.4427E-05
QVR	0.000219439	GLA	0.00014988	QHT	3.4427E-05
RSE	0.000219439	LTP	0.00014988	RKT	3.4427E-05
SRH	0.000219439	DTR	0.00014917	RKW	3.4427E-05
KLG	0.000219005	HGV	0.00014917	RMT	3.4427E-05
LDT	0.000219005	IME	0.00014917	YER	3.4427E-05
LPH	0.000219005	KEF	0.00014917	DNP	3.41776E-05
AAV	0.000218571	MKH	0.00014917	FHH	3.41776E-05
GTG	0.000218571	EFR	0.00014881	FKW	3.41776E-05
LKP	0.000218571	PTT	0.00014845	FSA	3.41776E-05
NTT	0.000218571	SWV	0.00014845	GES	3.41776E-05
RPQ	0.000218571	FTC	0.00014774	LIR	3.41776E-05
SRI	0.000218571	NKV	0.00014774	PLI	3.41776E-05
TKL	0.000218571	WFT	0.00014774	YAY	3.41776E-05
YSA	0.000218571	GKN	0.00014738	YDR	3.41776E-05
AHL	0.000218138	LPA	0.00014738	CLL	3.39281E-05
GNL	0.000218138	MHT	0.00014738	FKT	3.39281E-05
LCP	0.000218138	NNQ	0.00014738	HSR	3.39281E-05
QRA	0.000218138	PVP	0.00014738	PSH	3.39281E-05
WST	0.000218138	LRN	0.00014703	QPA	3.39281E-05
CVL	0.000217704	IYL	0.00014667	RLH	3.39281E-05
SSF	0.000217704	TQA	0.00014667	WTT	3.39281E-05
TAG	0.000217704	YDQ	0.00014667	FRA	3.36786E-05
TVG	0.000217704	CRH	0.00014631	ISR	3.36786E-05
VYL	0.000217704	LDL	0.00014631	SWM	3.36786E-05
GVG	0.00021727	FGN	0.00014596	VCR	3.36786E-05
LPM	0.00021727	FRS	0.00014596	YHS	3.36786E-05
DRA	0.000216836	LHK	0.00014596	YWT	3.36786E-05
FAR	0.000216836	NAG	0.00014596	EHA	3.34291E-05

IRT	0.000216836	STM	0.00014596	HTY	3.34291E-05
RLY	0.000216836	FWW	0.0001456	IYT	3.34291E-05
SYT	0.000216836	HTS	0.0001456	KSC	3.34291E-05
WPR	0.000216836	INV	0.00014524	LGH	3.34291E-05
YGL	0.000216836	PLA	0.00014524	LKF	3.34291E-05
LMP	0.000216403	FML	0.00014489	SRQ	3.34291E-05
LVW	0.000216403	FVA	0.00014489	WFV	3.34291E-05
QLP	0.000216403	AVR	0.00014453	GYG	3.31797E-05
TVV	0.000216403	RNL	0.00014453	NMG	3.31797E-05
ATV	0.000215969	DFC	0.00014417	PRD	3.31797E-05
FGS	0.000215969	YIC	0.00014417	EKY	3.29302E-05
LTQ	0.000215969	DLG	0.00014382	GQS	3.29302E-05
RRF	0.000215969	MGF	0.00014346	ISS	3.29302E-05
SSD	0.000215969	NLT	0.00014346	RLL	3.29302E-05
WVL	0.000215969	PYV	0.00014346	WRP	3.29302E-05
EVS	0.000215535	VNT	0.00014346	YYA	3.29302E-05
GAV	0.000215535	NDG	0.0001431	FRK	3.26807E-05
HPA	0.000215535	WGA	0.0001431	GPF	3.26807E-05
IPR	0.000215535	YVR	0.0001431	NSR	3.26807E-05
NGS	0.000215535	KVG	0.00014274	WFA	3.26807E-05
TMR	0.000215535	LLA	0.00014239	WHC	3.26807E-05
VLW	0.000215535	MPD	0.00014239	WRW	3.26807E-05
VNS	0.000215535	WIE	0.00014239	ATD	3.24313E-05
DRV	0.000215102	YDL	0.00014239	FYT	3.24313E-05
PVG	0.000215102	LMC	0.00014203	LCI	3.24313E-05
AMR	0.000214234	TVS	0.00014203	RCC	3.24313E-05
LWP	0.000214234	DGQ	0.00014167	RWS	3.24313E-05
SRD	0.000214234	DNT	0.00014167	SRS	3.24313E-05
VRM	0.000214234	KIF	0.00014167	HKR	3.21818E-05
ALC	0.000213801	TLR	0.00014167	LHY	3.21818E-05
ESP	0.000213801	VCQ	0.00014167	VCA	3.21818E-05
SNT	0.000213801	CMD	0.00014132	VLS	3.21818E-05
ANS	0.000213367	CLA	0.00014096	WQQ	3.21818E-05
QRT	0.000213367	DLH	0.00014096	API	3.19323E-05
VLN	0.000213367	HKG	0.00014096	AQL	3.19323E-05
ILP	0.000212933	HVY	0.00014096	GRH	3.19323E-05
LDP	0.000212933	RRW	0.00014096	GTT	3.19323E-05
TGG	0.000212933	HNP	0.0001406	LAW	3.19323E-05
TLK	0.000212933	RGR	0.0001406	RPY	3.19323E-05
ESA	0.0002125	ECA	0.00014025	RSQ	3.19323E-05
ETL	0.0002125	GLM	0.00014025	WTL	3.19323E-05
EVR	0.0002125	LYH	0.00014025	YRR	3.19323E-05
IPS	0.0002125	PPS	0.00014025	ETH	3.16828E-05
KGL	0.0002125	SDC	0.00014025	IKS	3.16828E-05

KGR	0.0002125	ELR	0.00013989	LGG	3.16828E-05
PLM	0.0002125	FLG	0.00013989	NYT	3.16828E-05
PLW	0.0002125	GYD	0.00013989	PIF	3.16828E-05
QAS	0.0002125	LAD	0.00013989	RLD	3.16828E-05
RRI	0.0002125	GAR	0.00013953	SWP	3.16828E-05
TCL	0.0002125	AES	0.00013918	WSA	3.16828E-05
AVG	0.000212066	GTP	0.00013918	ASL	3.14334E-05
HSV	0.000212066	VMP	0.00013918	FQP	3.14334E-05
IVL	0.000212066	YVC	0.00013918	GHG	3.14334E-05
SKT	0.000212066	LYV	0.00013882	INR	3.14334E-05
VIS	0.000212066	MDT	0.00013882	PRS	3.14334E-05
VYR	0.000212066	PRL	0.00013882	SLV	3.14334E-05
HTA	0.000211632	IVD	0.00013846	SPC	3.14334E-05
PNL	0.000211632	KAR	0.00013846	WYL	3.14334E-05
VKL	0.000211632	MWE	0.00013846	YSH	3.14334E-05
CTR	0.000211199	VGR	0.00013846	GKV	3.11839E-05
ITL	0.000211199	AYE	0.00013811	GRF	3.11839E-05
PLD	0.000211199	VRV	0.00013775	HPD	3.11839E-05
PWL	0.000211199	VSE	0.00013775	LQA	3.11839E-05
RDT	0.000211199	WMR	0.00013775	PWW	3.11839E-05
VHS	0.000211199	KNS	0.00013739	VMM	3.11839E-05
ALK	0.000210765	HLF	0.00013703	ARP	3.09344E-05
ARQ	0.000210765	LLI	0.00013703	MTY	3.09344E-05
DPP	0.000210765	TDL	0.00013703	SRP	3.09344E-05
ELP	0.000210765	QNL	0.00013668	WMR	3.09344E-05
LPW	0.000210765	RAW	0.00013668	YYL	3.09344E-05
LTC	0.000210765	VVK	0.00013668	DEE	3.0685E-05
RSI	0.000210765	ESV	0.00013632	EPY	3.0685E-05
SFT	0.000210765	SVY	0.00013632	FCA	3.0685E-05
VDS	0.000210765	TLD	0.00013632	FTP	3.0685E-05
GRM	0.000210331	VGP	0.00013632	GMR	3.0685E-05
LET	0.000210331	GIS	0.00013596	LLA	3.0685E-05
LTM	0.000210331	SHQ	0.00013596	RPD	3.0685E-05
NRV	0.000210331	QSG	0.00013561	FHT	3.04355E-05
VLC	0.000210331	RYN	0.00013561	FLF	3.04355E-05
CST	0.000209898	SNG	0.00013561	GYV	3.04355E-05
CVR	0.000209898	GGT	0.00013525	SMT	3.04355E-05
PIR	0.000209898	PLP	0.00013525	YPR	3.04355E-05
GGG	0.000209464	RTL	0.00013525	FDW	3.0186E-05
PNS	0.000209464	SFT	0.00013525	FTH	3.0186E-05
ADL	0.00020903	VFG	0.00013525	LGS	3.0186E-05
ALW	0.00020903	ESA	0.00013489	LTW	3.0186E-05
AMS	0.00020903	TRA	0.00013489	SPR	3.0186E-05
ERA	0.00020903	YNM	0.00013454	YGR	3.0186E-05

HPT	0.00020903	KPF	0.00013418	AMY	2.99365E-05
IST	0.00020903	RQT	0.00013382	ARR	2.99365E-05
LFA	0.00020903	GTL	0.00013347	ATN	2.99365E-05
PHP	0.00020903	LSY	0.00013347	ICL	2.99365E-05
PQS	0.00020903	SCL	0.00013347	MRL	2.99365E-05
YAS	0.00020903	SRR	0.00013347	REQ	2.99365E-05
CLT	0.000208597	IST	0.00013311	VLT	2.99365E-05
KGS	0.000208597	ITR	0.00013311	CRR	2.96871E-05
RMT	0.000208597	ASW	0.00013275	LER	2.96871E-05
DGS	0.000208163	DFN	0.00013275	LPA	2.96871E-05
DVT	0.000208163	LVL	0.00013275	LTH	2.96871E-05
FRV	0.000208163	WER	0.00013275	LYG	2.96871E-05
GKL	0.000208163	WIG	0.00013275	MLR	2.96871E-05
GKR	0.000208163	CKP	0.0001324	RAQ	2.96871E-05
RMA	0.000208163	HEH	0.0001324	RNG	2.96871E-05
VFL	0.000208163	KPR	0.0001324	RYH	2.96871E-05
VKR	0.000208163	KST	0.0001324	WDR	2.96871E-05
VNR	0.000208163	SRQ	0.0001324	CSF	2.94376E-05
AHR	0.000207729	WVD	0.0001324	FRI	2.94376E-05
GVV	0.000207729	DNF	0.00013204	GVQ	2.94376E-05
LQT	0.000207729	FLL	0.00013204	GWV	2.94376E-05
PFL	0.000207729	LPL	0.00013204	KSS	2.94376E-05
PRM	0.000207729	MMD	0.00013204	LSE	2.94376E-05
QSP	0.000207729	RTA	0.00013204	LYH	2.94376E-05
QSA	0.000207296	VLN	0.00013168	PHH	2.94376E-05
RYA	0.000207296	YKF	0.00013168	PWR	2.94376E-05
SSI	0.000207296	ARH	0.00013132	QTS	2.94376E-05
ANR	0.000206862	CRS	0.00013132	RQA	2.94376E-05
GAG	0.000206862	NCC	0.00013132	RSF	2.94376E-05
LDA	0.000206862	PRG	0.00013097	VRT	2.94376E-05
LSY	0.000206862	YAA	0.00013097	WGG	2.94376E-05
LVM	0.000206862	HHD	0.00013061	WQR	2.94376E-05
SDA	0.000206862	PCD	0.00013061	WTP	2.94376E-05
GNS	0.000206428	PLS	0.00013061	AMR	2.91881E-05
LIP	0.000206428	RTT	0.00013061	FCN	2.91881E-05
ALQ	0.000205995	SGW	0.00013025	FTW	2.91881E-05
EAL	0.000205995	IQF	0.0001299	GFG	2.91881E-05
GKS	0.000205995	RMY	0.0001299	KEQ	2.91881E-05
PLC	0.000205995	SHA	0.0001299	LGM	2.91881E-05
VHL	0.000205995	WSI	0.0001299	WTF	2.91881E-05
SRF	0.000205561	AMG	0.00012954	YHC	2.91881E-05
TRW	0.000205561	FEW	0.00012954	FAR	2.89387E-05
RDA	0.000205127	HTV	0.00012954	GLD	2.89387E-05
RYT	0.000205127	KML	0.00012954	RTL	2.89387E-05

TAV	0.000205127	SPT	0.00012918	VES	2.89387E-05
VIR	0.000205127	TNN	0.00012918	WLP	2.89387E-05
WLA	0.000205127	VDN	0.00012918	YSV	2.89387E-05
WTS	0.000205127	GYV	0.00012883	FGT	2.86892E-05
CPL	0.000204694	VAY	0.00012883	FPM	2.86892E-05
LCA	0.000204694	QKQ	0.00012847	GIG	2.86892E-05
LWT	0.000204694	RGV	0.00012847	IEL	2.86892E-05
PNR	0.000204694	RIG	0.00012811	LWY	2.86892E-05
SSY	0.000204694	TRK	0.00012811	NGQ	2.86892E-05
ERP	0.00020426	LNK	0.00012776	NTS	2.86892E-05
LVC	0.00020426	LTF	0.00012776	PLH	2.86892E-05
PKL	0.00020426	GSK	0.0001274	QTC	2.86892E-05
PLE	0.00020426	QVF	0.0001274	RYR	2.86892E-05
THL	0.00020426	REN	0.0001274	SLI	2.86892E-05
TLQ	0.00020426	WMP	0.0001274	WLQ	2.86892E-05
YSV	0.00020426	EIV	0.00012704	YTL	2.86892E-05
GLM	0.000203826	HTR	0.00012704	APR	2.84397E-05
IVS	0.000203826	IPY	0.00012704	CGR	2.84397E-05
PGV	0.000203826	RCD	0.00012704	GWP	2.84397E-05
PQL	0.000203826	KRV	0.00012669	IGR	2.84397E-05
QVL	0.000203826	FFA	0.00012633	ILG	2.84397E-05
RNA	0.000203826	KRY	0.00012633	IMC	2.84397E-05
TGV	0.000203826	LTH	0.00012633	IWW	2.84397E-05
FSA	0.000203393	FTF	0.00012597	LCF	2.84397E-05
GMR	0.000203393	HLP	0.00012597	WQG	2.84397E-05
ITR	0.000203393	VAP	0.00012597	YQR	2.84397E-05
LMA	0.000203393	IGG	0.00012562	YYS	2.84397E-05
LPK	0.000203393	EYI	0.00012526	EHG	2.81903E-05
RSY	0.000203393	LFI	0.00012526	ESS	2.81903E-05
SNP	0.000203393	LMG	0.00012526	FKQ	2.81903E-05
TNS	0.000203393	AYK	0.0001249	GKI	2.81903E-05
TQR	0.000203393	LHV	0.0001249	ICR	2.81903E-05
TSM	0.000203393	LWF	0.0001249	KTY	2.81903E-05
VLQ	0.000203393	RSP	0.0001249	LFV	2.81903E-05
VMS	0.000203393	STC	0.0001249	VWQ	2.81903E-05
WTL	0.000203393	ART	0.00012454	WPR	2.81903E-05
DSG	0.000202959	FLP	0.00012454	YHL	2.81903E-05
GMS	0.000202959	KNV	0.00012454	GAS	2.79408E-05
NTP	0.000202959	MYV	0.00012454	LKT	2.79408E-05
PCR	0.000202959	NMI	0.00012454	PNH	2.79408E-05
THR	0.000202959	RSK	0.00012454	STP	2.79408E-05
TMS	0.000202959	HPL	0.00012419	VSR	2.79408E-05
TNL	0.000202959	KIG	0.00012419	WSQ	2.79408E-05
AKL	0.000202525	LLK	0.00012419	AQS	2.76913E-05

HSG	0.000202525	LKA	0.00012383	ASC	2.76913E-05
IRP	0.000202525	LRQ	0.00012383	AYS	2.76913E-05
RKP	0.000202525	SLG	0.00012383	LQH	2.76913E-05
RRY	0.000202525	SML	0.00012383	QSH	2.76913E-05
VEL	0.000202525	GRD	0.00012347	WTR	2.76913E-05
YPP	0.000202525	QCN	0.00012347	DHP	2.74418E-05
CRT	0.000202092	RVK	0.00012347	FYW	2.74418E-05
IPL	0.000202092	TCC	0.00012347	GSH	2.74418E-05
LAW	0.000202092	TRG	0.00012347	RKH	2.74418E-05
PWR	0.000202092	MLQ	0.00012276	RPA	2.74418E-05
TML	0.000202092	SSK	0.00012276	RRL	2.74418E-05
WRT	0.000202092	AVT	0.0001224	SKL	2.74418E-05
WVS	0.000202092	ALW	0.00012205	STL	2.74418E-05
AHS	0.000201658	HSI	0.00012205	VCP	2.74418E-05
DGR	0.000201658	IFA	0.00012205	WGD	2.74418E-05
VES	0.000201658	KLG	0.00012205	YSA	2.74418E-05
VWR	0.000201658	VNV	0.00012205	DQQ	2.71924E-05
WPL	0.000201658	LTT	0.00012133	FTM	2.71924E-05
ARM	0.000201224	NIG	0.00012098	HLR	2.71924E-05
CTL	0.000201224	FTG	0.00012062	LPD	2.71924E-05
LFV	0.000201224	HFV	0.00012062	LPF	2.71924E-05
LYP	0.000201224	IEN	0.00012062	LPW	2.71924E-05
NSG	0.000201224	KLL	0.00012062	RHS	2.71924E-05
RSF	0.000201224	LAM	0.00012062	RPR	2.71924E-05
SRY	0.000201224	PYN	0.00012062	RWW	2.71924E-05
VCR	0.000201224	QPV	0.00012062	WDC	2.71924E-05
VLE	0.000201224	QSK	0.00012062	WNW	2.71924E-05
AML	0.000200791	TYD	0.00012062	WPC	2.71924E-05
LGM	0.000200791	FQL	0.00012026	YMY	2.71924E-05
LVK	0.000200791	FGR	0.00011991	YPS	2.71924E-05
PML	0.000200791	GKS	0.00011991	EYA	2.69429E-05
VHR	0.000200791	WLT	0.00011991	FCF	2.69429E-05
AVV	0.000200357	YVP	0.00011991	LIS	2.69429E-05
GLC	0.000200357	AQP	0.00011955	NWR	2.69429E-05
KSV	0.000200357	DDN	0.00011955	PNC	2.69429E-05
LVN	0.000200357	LNT	0.00011955	SSA	2.69429E-05
LVQ	0.000200357	VQN	0.00011955	AQT	2.66934E-05
PDR	0.000200357	APL	0.00011919	ENL	2.66934E-05
VFS	0.000200357	DQI	0.00011919	FAS	2.66934E-05
VRK	0.000200357	ECI	0.00011919	FNP	2.66934E-05
ARW	0.000199923	IEM	0.00011919	GPH	2.66934E-05
ELV	0.000199923	IRI	0.00011919	KAH	2.66934E-05
GLQ	0.000199923	DNA	0.00011883	LLC	2.66934E-05
PRD	0.000199923	FTA	0.00011883	QNW	2.66934E-05

QVS	0.000199923	GPS	0.00011883	RKN	2.66934E-05
SVW	0.000199923	KSS	0.00011883	SAT	2.66934E-05
TRM	0.000199923	PWS	0.00011883	SGR	2.66934E-05
WGR	0.00019949	ITA	0.00011848	VKS	2.66934E-05
AQR	0.000199056	DSA	0.00011812	WYG	2.66934E-05
ELG	0.000199056	LDD	0.00011812	ALN	2.6444E-05
ISP	0.000199056	DGD	0.00011776	CCL	2.6444E-05
LAC	0.000199056	RRL	0.00011776	CLR	2.6444E-05
LTN	0.000199056	MKS	0.00011741	IDT	2.6444E-05
QAR	0.000199056	RRR	0.00011741	KNS	2.6444E-05
RNP	0.000199056	LGV	0.00011705	LAR	2.6444E-05
SMT	0.000199056	HCA	0.00011669	LTP	2.6444E-05
CTS	0.000198622	SYP	0.00011669	PLN	2.6444E-05
ILA	0.000198622	FSS	0.00011634	QEH	2.6444E-05
LGQ	0.000198622	GVN	0.00011598	RNP	2.6444E-05
PFR	0.000198622	NFT	0.00011598	WGT	2.6444E-05
TFL	0.000198622	RHY	0.00011598	WIC	2.6444E-05
AWL	0.000198189	AHS	0.00011562	YWM	2.6444E-05
CLV	0.000198189	CKC	0.00011562	ALF	2.61945E-05
PKS	0.000198189	FTL	0.00011562	ENP	2.61945E-05
RCP	0.000198189	QAL	0.00011562	IRM	2.61945E-05
RCT	0.000198189	RLM	0.00011562	LRY	2.61945E-05
SDT	0.000198189	CAY	0.00011527	LVR	2.61945E-05
SHT	0.000198189	PKS	0.00011527	QPR	2.61945E-05
AYS	0.000197755	TLT	0.00011527	RVG	2.61945E-05
CLP	0.000197755	EMM	0.00011491	VRQ	2.61945E-05
EAS	0.000197755	EHH	0.00011455	WEW	2.61945E-05
GNR	0.000197755	ELT	0.00011455	WLE	2.61945E-05
LNG	0.000197755	GDL	0.00011455	WYP	2.61945E-05
SPQ	0.000197755	EDP	0.0001142	ANS	2.5945E-05
TKS	0.000197755	EVA	0.0001142	KLS	2.5945E-05
WGL	0.000197755	HWV	0.0001142	LIY	2.5945E-05
GEL	0.000197321	WLM	0.0001142	LPP	2.5945E-05
GML	0.000197321	ESH	0.00011384	NNR	2.5945E-05
IVR	0.000197321	HNL	0.00011384	NVR	2.5945E-05
LAQ	0.000197321	IGW	0.00011384	PTN	2.5945E-05
PIL	0.000197321	YTN	0.00011384	SKS	2.5945E-05
PMS	0.000197321	DTD	0.00011348	VCS	2.5945E-05
RTM	0.000197321	SFR	0.00011348	WGY	2.5945E-05
AIR	0.000196888	LNH	0.00011312	YWP	2.5945E-05
CAL	0.000196888	HTT	0.00011241	FPP	2.56955E-05
CPR	0.000196888	NGG	0.00011241	PSA	2.56955E-05
IGL	0.000196888	NNK	0.00011241	RQH	2.56955E-05
KPA	0.000196888	PPL	0.00011241	SIY	2.56955E-05

PCL	0.000196888	YLL	0.00011241	SLA	2.56955E-05
TDL	0.000196888	YSC	0.00011241	VRH	2.56955E-05
TKR	0.000196888	CAE	0.00011205	WMS	2.56955E-05
VIL	0.000196888	GTR	0.00011205	WNY	2.56955E-05
ACL	0.000196454	LWA	0.00011205	WYS	2.56955E-05
AGV	0.000196454	RTV	0.00011205	GSP	2.54461E-05
AKR	0.000196454	NSL	0.0001117	NGL	2.54461E-05
DRG	0.000196454	QVS	0.0001117	PEH	2.54461E-05
FRG	0.000196454	SGS	0.0001117	RFA	2.54461E-05
HRG	0.000196454	VWN	0.0001117	RYN	2.54461E-05
LHG	0.000196454	ERH	0.00011134	STA	2.54461E-05
LKA	0.000196454	GPA	0.00011134	VVL	2.54461E-05
PLN	0.000196454	MLD	0.00011134	WIR	2.54461E-05
PSM	0.000196454	RMW	0.00011134	WSY	2.54461E-05
TDS	0.000196454	RSN	0.00011134	WWV	2.54461E-05
TRK	0.000196454	DDS	0.00011098	YLS	2.54461E-05
VRQ	0.000196454	ELS	0.00011098	DHS	2.51966E-05
AEL	0.00019602	WRK	0.00011098	FLA	2.51966E-05
ALH	0.00019602	YVH	0.00011098	LEW	2.51966E-05
DTP	0.00019602	PGP	0.00011063	PCL	2.51966E-05
GDL	0.00019602	CNW	0.00011027	PGQ	2.51966E-05
ILV	0.00019602	DIM	0.00011027	SSP	2.51966E-05
ITS	0.00019602	GMA	0.00011027	VQL	2.51966E-05
LCG	0.00019602	PIR	0.00011027	YLA	2.51966E-05
MLT	0.00019602	RWK	0.00011027	AST	2.49471E-05
PER	0.00019602	WEP	0.00011027	DKH	2.49471E-05
PHA	0.00019602	YQS	0.00011027	EHS	2.49471E-05
PVV	0.00019602	TPA	0.00010991	GCL	2.49471E-05
SPC	0.00019602	LQH	0.00010956	RKY	2.49471E-05
TFS	0.00019602	AWA	0.0001092	TWL	2.49471E-05
TLN	0.00019602	HLA	0.0001092	VCC	2.49471E-05
ARC	0.000195587	ANR	0.00010884	VST	2.49471E-05
CLA	0.000195587	DDY	0.00010884	VWT	2.49471E-05
FPP	0.000195587	DGM	0.00010884	WST	2.49471E-05
MTS	0.000195587	SAH	0.00010884	ASP	2.46977E-05
RFT	0.000195587	LKV	0.00010849	EKR	2.46977E-05
SCT	0.000195587	SSH	0.00010849	FNF	2.46977E-05
VSW	0.000195587	WGH	0.00010849	FTC	2.46977E-05
CPS	0.000195153	DGI	0.00010813	KER	2.46977E-05
GDS	0.000195153	HVS	0.00010813	KKC	2.46977E-05
KRV	0.000195153	IYW	0.00010813	KMS	2.46977E-05
LQP	0.000195153	QYS	0.00010813	QKC	2.46977E-05
NPT	0.000195153	SDS	0.00010813	RDH	2.46977E-05
PCS	0.000195153	EAV	0.00010777	ALA	2.44482E-05

RTW	0.000195153	ESR	0.00010777	CKL	2.44482E-05
TIR	0.000195153	ETW	0.00010777	CKR	2.44482E-05
IAS	0.000194719	FAG	0.00010777	DDP	2.44482E-05
LIA	0.000194719	LGG	0.00010777	FFG	2.44482E-05
PPE	0.000194719	PVQ	0.00010777	FFV	2.44482E-05
PRH	0.000194719	RPN	0.00010777	KDR	2.44482E-05
SPW	0.000194719	DRE	0.00010742	KGH	2.44482E-05
STW	0.000194719	EPR	0.00010742	LGC	2.44482E-05
TRH	0.000194719	ATC	0.00010706	LPQ	2.44482E-05
VKS	0.000194719	FCL	0.00010706	LYV	2.44482E-05
GLW	0.000194285	PPT	0.00010706	PGL	2.44482E-05
LPE	0.000194285	RLT	0.00010706	QKL	2.44482E-05
NRG	0.000194285	LWN	0.0001067	RLC	2.44482E-05
SPM	0.000194285	TAT	0.0001067	VRF	2.44482E-05
SQT	0.000194285	WAA	0.0001067	YAW	2.44482E-05
WPS	0.000194285	GWE	0.00010634	YGH	2.44482E-05
CAS	0.000193852	RAL	0.00010634	ART	2.41987E-05
GHL	0.000193852	CYS	0.00010599	LFS	2.41987E-05
LWV	0.000193852	PLI	0.00010599	NKT	2.41987E-05
PKR	0.000193852	ALS	0.00010563	PRA	2.41987E-05
RHP	0.000193852	GPL	0.00010563	RVT	2.41987E-05
RTQ	0.000193852	IYY	0.00010563	RYL	2.41987E-05
TQL	0.000193852	PDR	0.00010563	SPA	2.41987E-05
VDR	0.000193852	YEV	0.00010563	STC	2.41987E-05
VLD	0.000193852	RRG	0.00010527	TMS	2.41987E-05
VRW	0.000193852	RTD	0.00010527	CSS	2.39492E-05
WVR	0.000193852	DYG	0.00010492	FDS	2.39492E-05
ADR	0.000193418	RSE	0.00010492	GLH	2.39492E-05
MTR	0.000193418	VTT	0.00010492	GRS	2.39492E-05
RTC	0.000193418	VVA	0.00010492	GYD	2.39492E-05
SPH	0.000193418	FVT	0.0001042	LEQ	2.39492E-05
VYS	0.000193418	LIQ	0.0001042	LES	2.39492E-05
AWR	0.000192984	RII	0.0001042	LGK	2.39492E-05
GSW	0.000192984	SPL	0.0001042	NET	2.39492E-05
PSW	0.000192984	AEM	0.00010385	NSL	2.39492E-05
RTH	0.000192984	CTF	0.00010385	PPC	2.39492E-05
AKS	0.000192551	DLV	0.00010385	PQA	2.39492E-05
AQL	0.000192551	GCT	0.00010385	RCG	2.39492E-05
GIL	0.000192551	PTL	0.00010385	RWR	2.39492E-05
RPC	0.000192551	FQP	0.00010349	VRP	2.39492E-05
STQ	0.000192551	KLI	0.00010349	YAR	2.39492E-05
CVS	0.000192117	PKL	0.00010349	DDH	2.36998E-05
LGC	0.000192117	FSR	0.00010313	FGA	2.36998E-05
PYR	0.000192117	KRT	0.00010313	HCR	2.36998E-05

RPH	0.000192117	CCC	0.00010278	KLL	2.36998E-05
RQP	0.000192117	MRK	0.00010278	KVG	2.36998E-05
SQP	0.000192117	ATD	0.00010242	LKQ	2.36998E-05
TER	0.000192117	ETR	0.00010242	LPV	2.36998E-05
WRP	0.000192117	FGY	0.00010242	LQT	2.36998E-05
LTH	0.000191683	RKC	0.00010242	PSK	2.36998E-05
PSC	0.000191683	SFD	0.00010242	SVR	2.36998E-05
QTP	0.000191683	ALN	0.00010206	WGS	2.36998E-05
TFR	0.000191683	STQ	0.00010206	WGW	2.36998E-05
TIL	0.000191683	KLS	0.00010171	WLM	2.36998E-05
TLE	0.000191683	NFL	0.00010171	YPL	2.36998E-05
VER	0.000191683	PNA	0.00010171	FMG	2.34503E-05
WLP	0.000191683	RKP	0.00010171	NIR	2.34503E-05
CRP	0.00019125	AET	0.00010135	QYP	2.34503E-05
GLN	0.00019125	EIP	0.00010135	RHR	2.34503E-05
PYS	0.00019125	LKI	0.00010135	RQT	2.34503E-05
QLG	0.00019125	WFY	0.00010099	RYD	2.34503E-05
TDR	0.00019125	YRT	0.00010099	SRH	2.34503E-05
TQS	0.00019125	GPR	0.00010063	SWS	2.34503E-05
YRV	0.00019125	VLI	0.00010063	WQL	2.34503E-05
EAR	0.000190816	VNE	0.00010063	DLS	2.32008E-05
LAM	0.000190816	WLG	0.00010063	GAQ	2.32008E-05
VRN	0.000190816	CHL	0.00010028	GLC	2.32008E-05
EGL	0.000190382	LYN	0.00010028	GLF	2.32008E-05
GRW	0.000190382	RDI	0.00010028	IFR	2.32008E-05
GSM	0.000190382	DVY	9.9921E-05	LKH	2.32008E-05
HTT	0.000190382	NLC	9.9921E-05	LLQ	2.32008E-05
IAL	0.000190382	SVW	9.9921E-05	LQR	2.32008E-05
LTE	0.000190382	ASP	9.9564E-05	MNS	2.32008E-05
MLA	0.000190382	GRG	9.9564E-05	PTH	2.32008E-05
RKA	0.000190382	LPH	9.9564E-05	SHA	2.32008E-05
RPM	0.000190382	MKM	9.9564E-05	SRG	2.32008E-05
TLH	0.000190382	NNL	9.9564E-05	VHT	2.32008E-05
TLI	0.000190382	FHN	9.9207E-05	AMH	2.29514E-05
VLH	0.000190382	HCL	9.9207E-05	DLL	2.29514E-05
WSA	0.000190382	LAR	9.9207E-05	FKP	2.29514E-05
GWL	0.000189949	LSG	9.9207E-05	GGR	2.29514E-05
KRG	0.000189949	CMF	9.8494E-05	GHP	2.29514E-05
LCV	0.000189949	PPQ	9.8494E-05	ILW	2.29514E-05
RTK	0.000189949	WFS	9.8494E-05	LNH	2.29514E-05
SFA	0.000189949	GGL	9.8137E-05	NLH	2.29514E-05
WSP	0.000189949	PPY	9.8137E-05	QPS	2.29514E-05
GCL	0.000189515	QNC	9.8137E-05	RKP	2.29514E-05
PFS	0.000189515	VKS	9.8137E-05	RQS	2.29514E-05

QPA	0.000189515	GCK	9.778E-05	STM	2.29514E-05
SHA	0.000189515	KNT	9.778E-05	VCN	2.29514E-05
VRC	0.000189515	LFS	9.778E-05	FFS	2.27019E-05
WLV	0.000189515	RSF	9.778E-05	GKQ	2.27019E-05
DAT	0.000189081	TMV	9.778E-05	GSG	2.27019E-05
IGR	0.000189081	TSS	9.778E-05	IRS	2.27019E-05
LAH	0.000189081	IFC	9.7423E-05	LMW	2.27019E-05
LHV	0.000189081	LTR	9.7423E-05	NAR	2.27019E-05
LPD	0.000189081	DTH	9.7066E-05	NTR	2.27019E-05
PDL	0.000189081	GGA	9.7066E-05	PRN	2.27019E-05
RHA	0.000189081	GWL	9.7066E-05	PTP	2.27019E-05
SIP	0.000189081	SDW	9.7066E-05	RGL	2.27019E-05
SVM	0.000189081	YKL	9.7066E-05	RIS	2.27019E-05
TSC	0.000189081	ELN	9.6353E-05	WWA	2.27019E-05
GGV	0.000188648	FLN	9.6353E-05	YDC	2.27019E-05
LAD	0.000188648	MSS	9.6353E-05	EDQ	2.24524E-05
LPN	0.000188648	TSA	9.6353E-05	GGP	2.24524E-05
PRK	0.000188648	DPM	9.5996E-05	GRG	2.24524E-05
AQS	0.000188214	FPI	9.5996E-05	KTR	2.24524E-05
ARH	0.000188214	LLT	9.5996E-05	LYC	2.24524E-05
LTK	0.000188214	RSG	9.5996E-05	NLN	2.24524E-05
MST	0.000188214	SPA	9.5996E-05	PTA	2.24524E-05
NPP	0.000188214	VPL	9.5996E-05	PWN	2.24524E-05
QGS	0.000188214	WWC	9.5996E-05	RGK	2.24524E-05
RIT	0.000188214	TTP	9.5639E-05	RPG	2.24524E-05
RVW	0.000188214	MFP	9.5282E-05	WFL	2.24524E-05
SET	0.000188214	PQR	9.5282E-05	WTW	2.24524E-05
CSP	0.00018778	RGA	9.5282E-05	YKC	2.24524E-05
DTT	0.00018778	RLA	9.5282E-05	ENH	2.22029E-05
FTT	0.00018778	ARL	9.4925E-05	FAF	2.22029E-05
GRC	0.00018778	GNI	9.4925E-05	FWE	2.22029E-05
LFP	0.00018778	LQP	9.4925E-05	LFN	2.22029E-05
STM	0.00018778	GRP	9.4568E-05	LHC	2.22029E-05
YSG	0.00018778	KSR	9.4568E-05	LPT	2.22029E-05
GIS	0.000187347	RML	9.4568E-05	QKH	2.22029E-05
KPP	0.000187347	SGQ	9.4211E-05	SRV	2.22029E-05
PRE	0.000187347	TGT	9.4211E-05	VYA	2.22029E-05
AIS	0.000186913	VHY	9.4211E-05	WGN	2.22029E-05
PSQ	0.000186913	ELA	9.3854E-05	WTG	2.22029E-05
RVM	0.000186913	GLG	9.3854E-05	ASA	2.19535E-05
SMP	0.000186913	KAI	9.3854E-05	ETC	2.19535E-05
ESV	0.000186479	YWY	9.3854E-05	FRY	2.19535E-05
GHR	0.000186479	FAF	9.3498E-05	GNG	2.19535E-05
GYS	0.000186479	NWI	9.3498E-05	MSL	2.19535E-05

MPS	0.000186479	SRK	9.3498E-05	PHG	2.19535E-05
MTL	0.000186479	TQP	9.3498E-05	RSG	2.19535E-05
PYL	0.000186479	YLS	9.3498E-05	RWC	2.19535E-05
RPN	0.000186479	GGI	9.3141E-05	SWV	2.19535E-05
WAL	0.000186479	LNR	9.3141E-05	VSC	2.19535E-05
ISV	0.000186046	PTH	9.3141E-05	WTH	2.19535E-05
ASW	0.000185612	GGH	9.2784E-05	YTF	2.19535E-05
GQS	0.000185612	LPN	9.2784E-05	ALG	2.1704E-05
LDG	0.000185612	QWV	9.2784E-05	CTY	2.1704E-05
LYV	0.000185612	FNL	9.2427E-05	ENS	2.1704E-05
RQT	0.000185612	GFT	9.2427E-05	FDR	2.1704E-05
SAC	0.000185612	INF	9.2427E-05	FPG	2.1704E-05
SKP	0.000185612	TVV	9.2427E-05	GPL	2.1704E-05
TRC	0.000185612	CFT	9.207E-05	GRA	2.1704E-05
TRQ	0.000185612	AGT	9.1713E-05	GRC	2.1704E-05
WAR	0.000185612	HYN	9.1713E-05	GRQ	2.1704E-05
IGS	0.000185178	MTP	9.1713E-05	KLA	2.1704E-05
LYG	0.000185178	MTR	9.1713E-05	KTC	2.1704E-05
PDS	0.000185178	RAS	9.1713E-05	LAQ	2.1704E-05
PLK	0.000185178	SVI	9.1713E-05	LLH	2.1704E-05
QRV	0.000185178	TIR	9.1713E-05	LPH	2.1704E-05
RMP	0.000185178	LFF	9.1356E-05	LTN	2.1704E-05
STC	0.000185178	RAR	9.1356E-05	PFT	2.1704E-05
VSH	0.000185178	RLQ	9.1356E-05	PKL	2.1704E-05
AYL	0.000184745	TFP	9.1356E-05	QRD	2.1704E-05
FCL	0.000184745	TKY	9.1356E-05	QWL	2.1704E-05
LGK	0.000184745	SGR	9.1E-05	RFS	2.1704E-05
MPL	0.000184745	VHL	9.1E-05	RHP	2.1704E-05
RET	0.000184745	ANG	9.0643E-05	SPM	2.1704E-05
RIA	0.000184745	KTS	9.0643E-05	VML	2.1704E-05
YTP	0.000184745	LYR	9.0643E-05	VTR	2.1704E-05
GQL	0.000184311	EIA	9.0286E-05	WFC	2.1704E-05
HAT	0.000184311	KFL	9.0286E-05	WPL	2.1704E-05
LQA	0.000184311	KLN	9.0286E-05	YHD	2.1704E-05
MRP	0.000184311	YLQ	9.0286E-05	YSE	2.1704E-05
SFP	0.000184311	API	8.9929E-05	ATP	2.14545E-05
SWT	0.000184311	AWP	8.9929E-05	CSW	2.14545E-05
TWL	0.000184311	RCQ	8.9929E-05	DSL	2.14545E-05
VSN	0.000184311	FFL	8.9572E-05	DSR	2.14545E-05
ARE	0.000183877	PIP	8.9572E-05	ETN	2.14545E-05
AYR	0.000183877	EDI	8.9215E-05	FLI	2.14545E-05
LMG	0.000183877	PRE	8.9215E-05	FPW	2.14545E-05
GCR	0.000183444	VRP	8.9215E-05	GFA	2.14545E-05
NVT	0.000183444	VSL	8.9215E-05	GTP	2.14545E-05

PPD	0.000183444	WSS	8.9215E-05	HTR	2.14545E-05
YTT	0.000183444	GSG	8.8858E-05	LPC	2.14545E-05
CSV	0.00018301	HRY	8.8858E-05	LPY	2.14545E-05
FPT	0.00018301	RDS	8.8858E-05	LQQ	2.14545E-05
GRH	0.00018301	SRM	8.8858E-05	MRR	2.14545E-05
RTN	0.00018301	APP	8.8502E-05	PNA	2.14545E-05
SIA	0.00018301	FSA	8.8502E-05	PRV	2.14545E-05
VQL	0.00018301	GPN	8.8502E-05	QLA	2.14545E-05
WAS	0.00018301	IMR	8.8502E-05	VPF	2.14545E-05
GSN	0.000182576	ISM	8.8502E-05	WDG	2.14545E-05
LGW	0.000182576	KLT	8.8145E-05	YFS	2.14545E-05
RFP	0.000182576	MCV	8.8145E-05	AKS	2.12051E-05
WRA	0.000182576	QSS	8.8145E-05	DSS	2.12051E-05
FSG	0.000182143	ALA	8.7788E-05	GDQ	2.12051E-05
LTD	0.000182143	GPP	8.7788E-05	GMS	2.12051E-05
SKA	0.000182143	LTY	8.7788E-05	GPC	2.12051E-05
SMA	0.000182143	QLG	8.7788E-05	HSS	2.12051E-05
VRE	0.000182143	SET	8.7788E-05	ISW	2.12051E-05
VSE	0.000182143	WKL	8.7788E-05	KRD	2.12051E-05
ADS	0.000181709	PRQ	8.7431E-05	MIR	2.12051E-05
ERV	0.000181709	RFN	8.7431E-05	RGP	2.12051E-05
GQR	0.000181709	WPA	8.7431E-05	RRQ	2.12051E-05
MPR	0.000181709	YIV	8.7431E-05	RWH	2.12051E-05
QRG	0.000181709	FRQ	8.7074E-05	SNS	2.12051E-05
RYP	0.000181709	LGQ	8.7074E-05	VKT	2.12051E-05
SHG	0.000181709	TFF	8.7074E-05	VVR	2.12051E-05
VQS	0.000181709	VNY	8.7074E-05	YFL	2.12051E-05
AFL	0.000181275	YRL	8.7074E-05	EDR	2.09556E-05
AIL	0.000181275	CIS	8.6717E-05	EYS	2.09556E-05
ASH	0.000181275	EVV	8.6717E-05	FCH	2.09556E-05
EGS	0.000181275	FWS	8.6717E-05	FQT	2.09556E-05
GWR	0.000181275	KFF	8.6717E-05	FRN	2.09556E-05
LAK	0.000181275	LGT	8.6717E-05	GQL	2.09556E-05
QGL	0.000181275	PIA	8.6717E-05	GQT	2.09556E-05
RPW	0.000181275	RGC	8.6717E-05	GSR	2.09556E-05
STH	0.000181275	SEP	8.6717E-05	GVG	2.09556E-05
TLD	0.000181275	SRP	8.6717E-05	KHG	2.09556E-05
TYR	0.000181275	VVC	8.6717E-05	KTT	2.09556E-05
ASE	0.000180842	GES	8.636E-05	KWT	2.09556E-05
KAT	0.000180842	NTP	8.636E-05	LNA	2.09556E-05
SAW	0.000180842	WFP	8.636E-05	PAD	2.09556E-05
SDP	0.000180842	CSL	8.6004E-05	PIR	2.09556E-05
TCR	0.000180842	LGK	8.6004E-05	QTY	2.09556E-05
YGS	0.000180842	TDH	8.6004E-05	RHN	2.09556E-05

ALI	0.000180408	FEY	8.5647E-05	RIR	2.09556E-05
EGR	0.000180408	TAA	8.5647E-05	RKF	2.09556E-05
GIR	0.000180408	APT	8.529E-05	SDR	2.09556E-05
KTT	0.000180408	ARS	8.529E-05	SSG	2.09556E-05
LPY	0.000180408	DTP	8.529E-05	TLL	2.09556E-05
PES	0.000180408	LYS	8.529E-05	VKR	2.09556E-05
PRN	0.000180408	GEL	8.4933E-05	VYT	2.09556E-05
RPD	0.000180408	RGD	8.4933E-05	WEL	2.09556E-05
SHP	0.000180408	SEA	8.4933E-05	WRV	2.09556E-05
TYS	0.000180408	CSG	8.4576E-05	ALP	2.07061E-05
VSC	0.000180408	LKY	8.4576E-05	ALQ	2.07061E-05
CGR	0.000179974	VND	8.4576E-05	AQI	2.07061E-05
GLK	0.000179974	ASS	8.4219E-05	ARQ	2.07061E-05
GRK	0.000179974	SKR	8.4219E-05	CKW	2.07061E-05
NPA	0.000179974	TFS	8.4219E-05	FYC	2.07061E-05
NVA	0.000179974	VRQ	8.4219E-05	GWT	2.07061E-05
SNV	0.000179974	YSK	8.4219E-05	KMT	2.07061E-05
TEL	0.000179974	CLY	8.3862E-05	LAS	2.07061E-05
VRH	0.000179974	IAS	8.3862E-05	LPG	2.07061E-05
YAT	0.000179974	WKI	8.3862E-05	NTT	2.07061E-05
PIS	0.000179541	DKV	8.3506E-05	QST	2.07061E-05
RPY	0.000179541	FSP	8.3506E-05	SPG	2.07061E-05
RVC	0.000179541	WAS	8.3506E-05	WLN	2.07061E-05
SCA	0.000179541	GSD	8.3149E-05	YRV	2.07061E-05
SKV	0.000179541	LQF	8.3149E-05	YRY	2.07061E-05
TLF	0.000179541	TPK	8.3149E-05	YSP	2.07061E-05
ALN	0.000179107	EEK	8.2792E-05	CFG	2.04566E-05
IRA	0.000179107	GWD	8.2792E-05	EEL	2.04566E-05
LVE	0.000179107	RKR	8.2792E-05	FKM	2.04566E-05
MVL	0.000179107	SNA	8.2792E-05	HSW	2.04566E-05
WGS	0.000179107	TGE	8.2792E-05	PRW	2.04566E-05
GFS	0.000178673	WPR	8.2792E-05	RRR	2.04566E-05
MGL	0.000178673	CQS	8.2435E-05	RTD	2.04566E-05
PEL	0.000178673	EMQ	8.2435E-05	TLS	2.04566E-05
PPM	0.000178673	FLR	8.2435E-05	WCG	2.04566E-05
RHT	0.000178673	GGD	8.2435E-05	WML	2.04566E-05
RKV	0.000178673	GNF	8.2435E-05	WPA	2.04566E-05
RTD	0.000178673	KNA	8.2435E-05	YLP	2.04566E-05
VLF	0.000178673	NDF	8.2435E-05	YTR	2.04566E-05
GDR	0.00017824	WLN	8.2435E-05	ENC	2.02072E-05
LDV	0.00017824	ADR	8.2078E-05	FES	2.02072E-05
LEV	0.00017824	CEK	8.2078E-05	GQP	2.02072E-05
LMV	0.00017824	FND	8.2078E-05	KKS	2.02072E-05
LNV	0.00017824	VRT	8.2078E-05	LEH	2.02072E-05

TCS	0.00017824	EQR	8.1721E-05	LIG	2.02072E-05
YRG	0.00017824	HIR	8.1721E-05	PMH	2.02072E-05
ASC	0.000177806	LCR	8.1721E-05	PYP	2.02072E-05
ASK	0.000177806	RIP	8.1721E-05	QNT	2.02072E-05
ASM	0.000177806	RPH	8.1721E-05	SIG	2.02072E-05
CAR	0.000177806	WLD	8.1721E-05	TLI	2.02072E-05
QGR	0.000177806	AMN	8.1364E-05	VYR	2.02072E-05
RDP	0.000177806	CVE	8.1364E-05	WKT	2.02072E-05
SDG	0.000177806	LLQ	8.1364E-05	YSN	2.02072E-05
VRD	0.000177806	GNS	8.1007E-05	AKT	1.99577E-05
VWS	0.000177806	KLE	8.1007E-05	AYA	1.99577E-05
HAP	0.000177372	LSF	8.1007E-05	EYR	1.99577E-05
KTP	0.000177372	PAP	8.1007E-05	FGK	1.99577E-05
PSH	0.000177372	SLT	8.1007E-05	FSK	1.99577E-05
QLV	0.000177372	HSA	8.0651E-05	FYQ	1.99577E-05
QSG	0.000177372	RAQ	8.0651E-05	GML	1.99577E-05
RPK	0.000177372	RFR	8.0651E-05	GPD	1.99577E-05
SPD	0.000177372	SRD	8.0651E-05	HCC	1.99577E-05
TRD	0.000177372	GNA	8.0294E-05	HST	1.99577E-05
VLI	0.000177372	MPE	8.0294E-05	HYR	1.99577E-05
GCS	0.000176939	NSA	8.0294E-05	LMG	1.99577E-05
IAR	0.000176939	PGE	8.0294E-05	LRD	1.99577E-05
LVD	0.000176939	YSS	8.0294E-05	NYA	1.99577E-05
STK	0.000176939	LIC	7.9937E-05	QLS	1.99577E-05
SVC	0.000176939	MPG	7.9937E-05	RMC	1.99577E-05
TIS	0.000176939	SWL	7.9937E-05	RMG	1.99577E-05
VLY	0.000176939	TRH	7.9937E-05	SHR	1.99577E-05
GES	0.000176505	TSP	7.958E-05	SQL	1.99577E-05
GYL	0.000176505	FNK	7.9223E-05	SWQ	1.99577E-05
LAN	0.000176505	PGS	7.9223E-05	VEV	1.99577E-05
RVK	0.000176505	TIK	7.9223E-05	VLG	1.99577E-05
SAM	0.000176505	HRR	7.8866E-05	WKA	1.99577E-05
TNT	0.000176505	KFR	7.8866E-05	WKQ	1.99577E-05
KSG	0.000176071	NPR	7.8866E-05	WLI	1.99577E-05
LEP	0.000176071	AGL	7.8509E-05	YKR	1.99577E-05
LGN	0.000176071	CNR	7.8509E-05	AML	1.97082E-05
VSK	0.000176071	LSC	7.8509E-05	GDA	1.97082E-05
AES	0.000175638	TSR	7.8509E-05	GMM	1.97082E-05
GLE	0.000175638	WPF	7.8509E-05	IRT	1.97082E-05
GLH	0.000175638	DWA	7.8153E-05	IWP	1.97082E-05
KVT	0.000175638	CKL	7.7796E-05	LAC	1.97082E-05
RAC	0.000175638	LYQ	7.7796E-05	LIL	1.97082E-05
RWT	0.000175638	NHD	7.7796E-05	LRK	1.97082E-05
SYP	0.000175638	NMA	7.7796E-05	QCR	1.97082E-05

VFR	0.000175638	QTR	7.7796E-05	RAD	1.97082E-05
ALE	0.000175204	SLN	7.7796E-05	RHG	1.97082E-05
ARD	0.000175204	SYV	7.7796E-05	SFL	1.97082E-05
ARN	0.000175204	TAR	7.7796E-05	SWT	1.97082E-05
GLD	0.000175204	GMR	7.7439E-05	VSP	1.97082E-05
PWS	0.000175204	LFP	7.7439E-05	WDS	1.97082E-05
RVQ	0.000175204	LNQ	7.7439E-05	WIS	1.97082E-05
STN	0.000175204	RTN	7.7439E-05	WVL	1.97082E-05
TYL	0.000175204	SDY	7.7439E-05	AHA	1.94588E-05
DPT	0.00017477	CKV	7.7082E-05	CTL	1.94588E-05
GRQ	0.00017477	DSL	7.7082E-05	DST	1.94588E-05
VMT	0.00017477	NAL	7.7082E-05	FNI	1.94588E-05
VRF	0.00017477	ATA	7.6725E-05	GIL	1.94588E-05
ACR	0.000174337	DLS	7.6725E-05	GSI	1.94588E-05
LTY	0.000174337	GTH	7.6725E-05	KPS	1.94588E-05
RQA	0.000174337	QTP	7.6725E-05	LFC	1.94588E-05
SCP	0.000174337	RKS	7.6725E-05	PCS	1.94588E-05
SPN	0.000174337	RNP	7.6725E-05	PYS	1.94588E-05
VSQ	0.000174337	VLL	7.6725E-05	QKS	1.94588E-05
CSA	0.000173903	LKR	7.6368E-05	RCR	1.94588E-05
GHS	0.000173903	PLV	7.6368E-05	RPP	1.94588E-05
NAT	0.000173903	RFC	7.6368E-05	SNR	1.94588E-05
PLY	0.000173903	TIN	7.6368E-05	SPH	1.94588E-05
PRY	0.000173903	HWL	7.6011E-05	VRW	1.94588E-05
RAQ	0.000173903	LAA	7.6011E-05	FAV	1.92093E-05
GFL	0.000173469	VEM	7.6011E-05	FAW	1.92093E-05
HTG	0.000173469	YYS	7.6011E-05	FTV	1.92093E-05
KTA	0.000173469	FPL	7.5655E-05	ITL	1.92093E-05
MLP	0.000173469	IRR	7.5655E-05	IWS	1.92093E-05
PLI	0.000173469	LGS	7.5655E-05	KNG	1.92093E-05
YTA	0.000173469	PPG	7.5655E-05	LGT	1.92093E-05
ARK	0.000173036	SLW	7.5655E-05	MKR	1.92093E-05
GER	0.000173036	ISI	7.5298E-05	NNA	1.92093E-05
SEA	0.000173036	NSN	7.5298E-05	RRG	1.92093E-05
TPW	0.000173036	QSV	7.5298E-05	WAR	1.92093E-05
TSW	0.000173036	WKC	7.5298E-05	YPP	1.92093E-05
HPV	0.000172602	ARV	7.4941E-05	ALY	1.89598E-05
LVF	0.000172602	FTP	7.4941E-05	ANR	1.89598E-05
NTV	0.000172602	GQL	7.4941E-05	GQG	1.89598E-05
RAN	0.000172602	LTI	7.4941E-05	GYR	1.89598E-05
TSK	0.000172602	PDA	7.4941E-05	IMQ	1.89598E-05
WRV	0.000172602	STS	7.4941E-05	KNT	1.89598E-05
DVV	0.000172168	TFW	7.4941E-05	LVL	1.89598E-05
GSH	0.000172168	YLP	7.4941E-05	MLL	1.89598E-05

HPG	0.000172168	GQT	7.4584E-05	PLV	1.89598E-05
HVT	0.000172168	GSR	7.4584E-05	PNP	1.89598E-05
KPT	0.000172168	PSW	7.4584E-05	PTC	1.89598E-05
LKV	0.000172168	TII	7.4584E-05	QSL	1.89598E-05
LQV	0.000172168	TPS	7.4584E-05	QTH	1.89598E-05
RAW	0.000172168	EDH	7.4227E-05	RWQ	1.89598E-05
RIP	0.000172168	LCT	7.4227E-05	SRW	1.89598E-05
RVH	0.000172168	LNV	7.4227E-05	SSV	1.89598E-05
SDV	0.000172168	PGR	7.4227E-05	VRV	1.89598E-05
STE	0.000172168	SNT	7.4227E-05	WPD	1.89598E-05
SVN	0.000172168	YFA	7.4227E-05	YAL	1.89598E-05
DTA	0.000171734	CKK	7.387E-05	YFG	1.89598E-05
FTP	0.000171734	GIT	7.387E-05	ANL	1.87103E-05
NVP	0.000171734	LCW	7.387E-05	CTS	1.87103E-05
PSD	0.000171734	LNS	7.387E-05	EYH	1.87103E-05
PSE	0.000171734	MRL	7.387E-05	FWY	1.87103E-05
SCG	0.000171734	SKL	7.387E-05	GAL	1.87103E-05
SGC	0.000171734	TKL	7.387E-05	GLP	1.87103E-05
MAS	0.000171301	KSL	7.3513E-05	HSL	1.87103E-05
RAD	0.000171301	LGP	7.3513E-05	IRG	1.87103E-05
RAM	0.000171301	LYA	7.3513E-05	KDC	1.87103E-05
REP	0.000171301	PKR	7.3513E-05	KGQ	1.87103E-05
RWP	0.000171301	PSV	7.3513E-05	KHH	1.87103E-05
SAK	0.000171301	RRT	7.3513E-05	LTM	1.87103E-05
VNT	0.000171301	RVH	7.3513E-05	NNH	1.87103E-05
VQR	0.000171301	SKM	7.3513E-05	PLA	1.87103E-05
ACS	0.000170867	TAD	7.3513E-05	PRP	1.87103E-05
ASN	0.000170867	VTP	7.3513E-05	QPC	1.87103E-05
CRV	0.000170867	AEP	7.3157E-05	QYS	1.87103E-05
HVA	0.000170867	IPH	7.3157E-05	RGN	1.87103E-05
LAE	0.000170867	NNT	7.3157E-05	RRC	1.87103E-05
MAL	0.000170867	PNK	7.3157E-05	SCR	1.87103E-05
MLV	0.000170867	RKQ	7.3157E-05	SSY	1.87103E-05
PNP	0.000170867	WGV	7.3157E-05	TTR	1.87103E-05
SVQ	0.000170867	ITS	7.28E-05	VTQ	1.87103E-05
ALD	0.000170433	PAL	7.28E-05	WSP	1.87103E-05
ANT	0.000170433	PRH	7.28E-05	YMR	1.87103E-05
GRD	0.000170433	QRR	7.28E-05	AHP	1.84609E-05
MGR	0.000170433	SAS	7.28E-05	AQP	1.84609E-05
ASD	0.00017	SLY	7.28E-05	ARA	1.84609E-05
ILG	0.00017	DSG	7.2443E-05	GRV	1.84609E-05
MRT	0.00017	GMY	7.2443E-05	GVL	1.84609E-05
RTY	0.00017	GRN	7.2443E-05	GYC	1.84609E-05
RVN	0.00017	LHP	7.2443E-05	IPL	1.84609E-05

VSD	0.00017	RYR	7.2443E-05	LKY	1.84609E-05
WRG	0.00017	SIS	7.2443E-05	RFL	1.84609E-05
YPT	0.00017	SQK	7.2443E-05	RLQ	1.84609E-05
AWS	0.000169566	VVN	7.2443E-05	RQN	1.84609E-05
LEA	0.000169566	YLV	7.2443E-05	SAS	1.84609E-05
PLF	0.000169566	YYH	7.2443E-05	SFT	1.84609E-05
PQA	0.000169566	GEP	7.2086E-05	WGA	1.84609E-05
RCA	0.000169566	GGS	7.2086E-05	YNC	1.84609E-05
SMV	0.000169566	PKT	7.2086E-05	AHH	1.82114E-05
SVE	0.000169566	PPD	7.2086E-05	AWR	1.82114E-05
CRA	0.000169132	QLR	7.2086E-05	CWQ	1.82114E-05
FGT	0.000169132	QSN	7.2086E-05	DNR	1.82114E-05
GSC	0.000169132	TPR	7.2086E-05	EER	1.82114E-05
ITT	0.000169132	VAN	7.2086E-05	GET	1.82114E-05
LPF	0.000169132	ELQ	7.1729E-05	GRT	1.82114E-05
SQA	0.000169132	FVP	7.1729E-05	GWR	1.82114E-05
SVK	0.000169132	LES	7.1729E-05	IGH	1.82114E-05
SYA	0.000169132	PPC	7.1729E-05	KHS	1.82114E-05
AER	0.000168699	PYA	7.1729E-05	MTT	1.82114E-05
ALF	0.000168699	VRY	7.1729E-05	NAC	1.82114E-05
CLG	0.000168699	AYL	7.1372E-05	QSG	1.82114E-05
LVH	0.000168699	GNH	7.1372E-05	SPV	1.82114E-05
RDV	0.000168699	GRQ	7.1372E-05	VKF	1.82114E-05
SAN	0.000168699	KGH	7.1372E-05	WSI	1.82114E-05
SGW	0.000168699	MWG	7.1372E-05	WYC	1.82114E-05
TES	0.000168699	RMN	7.1372E-05	CYL	1.79619E-05
TSD	0.000168699	AGR	7.1015E-05	FAQ	1.79619E-05
CGS	0.000168265	GAA	7.1015E-05	GAP	1.79619E-05
LWA	0.000167831	IPA	7.1015E-05	GHY	1.79619E-05
RAH	0.000167831	KTL	7.1015E-05	GRR	1.79619E-05
RWA	0.000167831	PRD	7.1015E-05	KLM	1.79619E-05
ARF	0.000167398	SAL	7.1015E-05	KNH	1.79619E-05
LIV	0.000167398	TFT	7.1015E-05	LKG	1.79619E-05
NGT	0.000167398	WGR	7.1015E-05	NFR	1.79619E-05
RNG	0.000167398	YAR	7.1015E-05	PNG	1.79619E-05
SCV	0.000167398	GAN	7.0659E-05	QDC	1.79619E-05
TRN	0.000167398	GGQ	7.0659E-05	RRH	1.79619E-05
YVT	0.000167398	GSH	7.0659E-05	SGH	1.79619E-05
AFS	0.000166964	LAL	7.0659E-05	SKG	1.79619E-05
GFR	0.000166964	LMS	7.0659E-05	SLP	1.79619E-05
ISA	0.000166964	MNI	7.0659E-05	SNC	1.79619E-05
SPE	0.000166964	QTA	7.0659E-05	SSF	1.79619E-05
SWP	0.000166964	VRG	7.0659E-05	WAP	1.79619E-05
PRF	0.00016653	WNT	7.0659E-05	YAH	1.79619E-05

PWP	0.00016653	CLK	7.0302E-05	YFR	1.79619E-05
SPK	0.00016653	IRV	7.0302E-05	YKG	1.79619E-05
APQ	0.000166097	MWA	7.0302E-05	YQL	1.79619E-05
DPV	0.000166097	RDW	7.0302E-05	YWQ	1.79619E-05
ESG	0.000166097	RIA	7.0302E-05	CEQ	1.77125E-05
HGT	0.000166097	ALF	6.9945E-05	CGP	1.77125E-05
HVP	0.000166097	GKA	6.9945E-05	EAY	1.77125E-05
LIG	0.000166097	GVR	6.9945E-05	GDS	1.77125E-05
REA	0.000166097	LNA	6.9945E-05	GLN	1.77125E-05
DLM	0.000165663	NGL	6.9945E-05	KSP	1.77125E-05
GRI	0.000165663	PSD	6.9945E-05	MPS	1.77125E-05
RNV	0.000165663	VKT	6.9945E-05	PRM	1.77125E-05
VKT	0.000165663	YSL	6.9945E-05	PRY	1.77125E-05
RFA	0.000165229	IND	6.9588E-05	QEQ	1.77125E-05
RPE	0.000165229	SAT	6.9588E-05	RAY	1.77125E-05
SAQ	0.000165229	SLR	6.9588E-05	RHT	1.77125E-05
VFT	0.000165229	FTS	6.9231E-05	SEL	1.77125E-05
AKT	0.000164796	RAH	6.9231E-05	TLG	1.77125E-05
DVP	0.000164796	SQP	6.9231E-05	WDL	1.77125E-05
FPA	0.000164796	STA	6.9231E-05	WEP	1.77125E-05
GYR	0.000164796	SYT	6.9231E-05	WNP	1.77125E-05
IRV	0.000164796	WNS	6.9231E-05	WTD	1.77125E-05
KVA	0.000164796	EAL	6.8874E-05	YCS	1.77125E-05
LVY	0.000164796	EIS	6.8874E-05	YHH	1.77125E-05
MRA	0.000164796	GTI	6.8874E-05	AMI	1.7463E-05
MSA	0.000164796	RHM	6.8874E-05	CSR	1.7463E-05
NTA	0.000164796	VKI	6.8874E-05	FCI	1.7463E-05
RGW	0.000164796	IRT	6.8517E-05	FKH	1.7463E-05
SAD	0.000164796	LIS	6.8517E-05	GKN	1.7463E-05
SWA	0.000164796	PFR	6.8517E-05	ITP	1.7463E-05
GWS	0.000164362	GDQ	6.816E-05	KLK	1.7463E-05
LGH	0.000164362	ILL	6.816E-05	KSA	1.7463E-05
NVG	0.000164362	KIN	6.816E-05	LGE	1.7463E-05
PSK	0.000164362	QAV	6.816E-05	LLN	1.7463E-05
PSN	0.000164362	QSP	6.816E-05	RRN	1.7463E-05
CGL	0.000163928	RDP	6.816E-05	SAQ	1.7463E-05
DPA	0.000163928	LWS	6.7804E-05	SGP	1.7463E-05
LFG	0.000163928	RRH	6.7804E-05	SSW	1.7463E-05
SYV	0.000163928	EST	6.7447E-05	VMT	1.7463E-05
TKT	0.000163928	FRV	6.7447E-05	WQP	1.7463E-05
EPA	0.000163495	GKP	6.7447E-05	YCG	1.7463E-05
EPP	0.000163495	AGS	6.709E-05	YSM	1.7463E-05
LWG	0.000163495	GMK	6.709E-05	YTG	1.7463E-05
NAA	0.000163495	GVS	6.709E-05	AIY	1.72135E-05

RGC	0.000163495	RNS	6.709E-05	AQQ	1.72135E-05
RGM	0.000163495	RQA	6.709E-05	ASF	1.72135E-05
STD	0.000163495	VEV	6.709E-05	AWM	1.72135E-05
LVI	0.000163061	YSP	6.709E-05	IAL	1.72135E-05
QNR	0.000163061	APR	6.6733E-05	IGQ	1.72135E-05
WSG	0.000163061	LRC	6.6733E-05	IKL	1.72135E-05
FAT	0.000162627	LYT	6.6733E-05	KVH	1.72135E-05
FFL	0.000162627	NTR	6.6733E-05	LRI	1.72135E-05
ETA	0.000162194	PNW	6.6733E-05	NAY	1.72135E-05
GLI	0.000162194	EDF	6.6376E-05	PSW	1.72135E-05
GSQ	0.000162194	GNT	6.6376E-05	RDR	1.72135E-05
RAK	0.000162194	KPS	6.6376E-05	RRA	1.72135E-05
RTE	0.000162194	NEH	6.6376E-05	RRP	1.72135E-05
TLY	0.000162194	PDG	6.6376E-05	RWP	1.72135E-05
YPA	0.000162194	QLT	6.6376E-05	SLH	1.72135E-05
AIT	0.00016176	WIR	6.6376E-05	VFL	1.72135E-05
ETT	0.00016176	CSS	6.6019E-05	WHT	1.72135E-05
GNT	0.00016176	FHP	6.6019E-05	WLF	1.72135E-05
LAF	0.00016176	LFG	6.6019E-05	WPQ	1.72135E-05
LKG	0.00016176	PDS	6.6019E-05	WVE	1.72135E-05
LTF	0.00016176	PGV	6.6019E-05	YFT	1.72135E-05
LTI	0.00016176	QWE	6.6019E-05	YPD	1.72135E-05
PRI	0.00016176	NGM	6.5662E-05	YPW	1.72135E-05
QPT	0.00016176	PYT	6.5662E-05	AKC	1.6964E-05
RMG	0.00016176	RSD	6.5662E-05	CWS	1.6964E-05
SNG	0.00016176	TRL	6.5662E-05	DAL	1.6964E-05
TPM	0.00016176	TTA	6.5662E-05	DGL	1.6964E-05
TRI	0.00016176	FAS	6.5306E-05	ESY	1.6964E-05
YSC	0.00016176	KRC	6.5306E-05	FPD	1.6964E-05
FGA	0.000161326	LCS	6.5306E-05	GYM	1.6964E-05
GSD	0.000161326	PIC	6.5306E-05	KED	1.6964E-05
QSV	0.000161326	PRC	6.5306E-05	LTD	1.6964E-05
TRY	0.000161326	RSC	6.5306E-05	NWM	1.6964E-05
ALY	0.000160893	YIH	6.5306E-05	PLF	1.6964E-05
APH	0.000160893	DGL	6.4949E-05	TLV	1.6964E-05
PTM	0.000160893	LMP	6.4949E-05	VEW	1.6964E-05
SHV	0.000160893	MPT	6.4949E-05	VLI	1.6964E-05
SIV	0.000160893	RAT	6.4949E-05	VTT	1.6964E-05
TSQ	0.000160893	RPQ	6.4949E-05	WCH	1.6964E-05
VPM	0.000160893	SAA	6.4949E-05	WCR	1.6964E-05
VTK	0.000160893	SLQ	6.4949E-05	WPN	1.6964E-05
HAA	0.000160459	KPV	6.4592E-05	WYR	1.6964E-05
NLM	0.000160459	SPS	6.4592E-05	YEW	1.6964E-05
PKT	0.000160459	TGR	6.4592E-05	YHQ	1.6964E-05

PSI	0.000160459	WRI	6.4592E-05	YKL	1.6964E-05
PTH	0.000160459	WSG	6.4592E-05	YWV	1.6964E-05
SVH	0.000160459	YLT	6.4592E-05	AHT	1.67146E-05
TSH	0.000160459	DSE	6.4235E-05	ASY	1.67146E-05
FVT	0.000160025	EEQ	6.4235E-05	AVL	1.67146E-05
GLF	0.000160025	ERA	6.4235E-05	CGH	1.67146E-05
GLY	0.000160025	MLR	6.4235E-05	CWP	1.67146E-05
GYA	0.000160025	PFL	6.4235E-05	DAH	1.67146E-05
HML	0.000160025	QTW	6.4235E-05	EPC	1.67146E-05
LPI	0.000160025	RIS	6.4235E-05	FIL	1.67146E-05
LQG	0.000160025	RSH	6.4235E-05	GPN	1.67146E-05
NGP	0.000160025	CCT	6.3878E-05	GQM	1.67146E-05
RHG	0.000160025	ESP	6.3878E-05	HMR	1.67146E-05
SMG	0.000160025	FKT	6.3878E-05	ICC	1.67146E-05
TPQ	0.000160025	LHH	6.3878E-05	IFL	1.67146E-05
TRE	0.000160025	LHI	6.3878E-05	IIG	1.67146E-05
GRN	0.000159592	QLS	6.3878E-05	IWQ	1.67146E-05
HGA	0.000159592	VGM	6.3878E-05	KNA	1.67146E-05
IMR	0.000159592	WDL	6.3878E-05	MLS	1.67146E-05
MSP	0.000159592	DLL	6.3521E-05	PEP	1.67146E-05
SAE	0.000159592	FRT	6.3521E-05	PEQ	1.67146E-05
SEP	0.000159592	GSI	6.3521E-05	PMS	1.67146E-05
VCA	0.000159592	KGT	6.3521E-05	QHH	1.67146E-05
VRI	0.000159592	LAY	6.3521E-05	QLR	1.67146E-05
VYT	0.000159592	PIK	6.3521E-05	RAK	1.67146E-05
AFR	0.000159158	RGQ	6.3521E-05	RTG	1.67146E-05
AQP	0.000159158	TRS	6.3521E-05	RVR	1.67146E-05
NPV	0.000159158	DST	6.3164E-05	RWA	1.67146E-05
PNT	0.000159158	FLC	6.3164E-05	SSM	1.67146E-05
LAI	0.000158724	FRA	6.3164E-05	TPT	1.67146E-05
MRV	0.000158724	HPR	6.3164E-05	VSM	1.67146E-05
QGA	0.000158724	KTR	6.3164E-05	VWH	1.67146E-05
RAE	0.000158724	PHA	6.3164E-05	WAD	1.67146E-05
RDG	0.000158724	PQC	6.3164E-05	WFS	1.67146E-05
SIG	0.000158724	RRA	6.3164E-05	WTA	1.67146E-05
ASQ	0.000158291	ECL	6.2808E-05	WWT	1.67146E-05
ERG	0.000158291	FTT	6.2808E-05	YQV	1.67146E-05
GRY	0.000158291	GAP	6.2808E-05	ANP	1.64651E-05
MVR	0.000158291	PTQ	6.2808E-05	ATC	1.64651E-05
SPY	0.000158291	RRF	6.2808E-05	CTC	1.64651E-05
STF	0.000158291	ATS	6.2451E-05	DVL	1.64651E-05
TPC	0.000158291	ERL	6.2451E-05	ELM	1.64651E-05
ANP	0.000157857	HIN	6.2451E-05	GHV	1.64651E-05
NMR	0.000157857	NLV	6.2451E-05	GRY	1.64651E-05

RFV	0.000157857	RKT	6.2451E-05	HHR	1.64651E-05
SGE	0.000157857	SWN	6.2451E-05	KEP	1.64651E-05
VRY	0.000157857	TGP	6.2451E-05	KIG	1.64651E-05
VSY	0.000157857	TYK	6.2451E-05	LNG	1.64651E-05
APC	0.000157423	YGF	6.2451E-05	PKS	1.64651E-05
ASY	0.000157423	YRA	6.2451E-05	PRH	1.64651E-05
IPA	0.000157423	FSV	6.2094E-05	QER	1.64651E-05
KGT	0.000157423	ISL	6.2094E-05	QGR	1.64651E-05
MVS	0.000157423	EML	6.1737E-05	RGT	1.64651E-05
RGD	0.000157423	GKY	6.1737E-05	SID	1.64651E-05
SAH	0.000157423	KDL	6.1737E-05	SPY	1.64651E-05
STI	0.000157423	PTW	6.1737E-05	YDQ	1.64651E-05
TPH	0.000157423	YWL	6.1737E-05	ALH	1.62156E-05
TWR	0.000157423	FLA	6.138E-05	ARY	1.62156E-05
TWS	0.000157423	GVL	6.138E-05	DPS	1.62156E-05
CRG	0.00015699	KCA	6.138E-05	FEP	1.62156E-05
MPP	0.00015699	MFG	6.138E-05	FYM	1.62156E-05
NLC	0.00015699	MFW	6.138E-05	GIV	1.62156E-05
RAF	0.00015699	RGS	6.138E-05	GTR	1.62156E-05
RKG	0.00015699	ARN	6.1023E-05	GVM	1.62156E-05
VSF	0.00015699	EGL	6.1023E-05	KAS	1.62156E-05
VSI	0.00015699	ENL	6.1023E-05	KEN	1.62156E-05
WTT	0.00015699	KSC	6.1023E-05	KSR	1.62156E-05
DAP	0.000156556	MSR	6.1023E-05	LLD	1.62156E-05
DTV	0.000156556	NLA	6.1023E-05	MLG	1.62156E-05
HLM	0.000156556	RLW	6.1023E-05	PVH	1.62156E-05
LAY	0.000156556	ASR	6.0666E-05	QLG	1.62156E-05
NAP	0.000156556	KGV	6.0666E-05	RPL	1.62156E-05
RVD	0.000156556	SQS	6.0666E-05	RVQ	1.62156E-05
RVE	0.000156556	TEI	6.0666E-05	SKQ	1.62156E-05
SPI	0.000156556	WII	6.0666E-05	SPF	1.62156E-05
SVY	0.000156556	YHG	6.0666E-05	VEM	1.62156E-05
SYG	0.000156556	GSM	6.031E-05	WRF	1.62156E-05
TMT	0.000156556	IFR	6.031E-05	WRY	1.62156E-05
VCT	0.000156556	LNF	6.031E-05	WWS	1.62156E-05
YAP	0.000156556	PNH	6.031E-05	EEP	1.59662E-05
FTA	0.000156122	AFR	5.9953E-05	FCK	1.59662E-05
GSE	0.000156122	DRL	5.9953E-05	FNG	1.59662E-05
LGI	0.000156122	EWL	5.9953E-05	FPQ	1.59662E-05
QTA	0.000156122	FRG	5.9953E-05	FTQ	1.59662E-05
RHV	0.000156122	LSN	5.9953E-05	GAN	1.59662E-05
THT	0.000156122	LVG	5.9953E-05	HWL	1.59662E-05
ETP	0.000155689	MTT	5.9953E-05	IMN	1.59662E-05
FVV	0.000155689	NRS	5.9953E-05	KTA	1.59662E-05

KTV	0.000155689	PDC	5.9953E-05	LVS	1.59662E-05
PNA	0.000155689	PEL	5.9953E-05	MMG	1.59662E-05
QTT	0.000155689	PNP	5.9953E-05	NGR	1.59662E-05
RCV	0.000155689	RER	5.9953E-05	NLG	1.59662E-05
RMV	0.000155689	RHK	5.9953E-05	PFN	1.59662E-05
SGQ	0.000155689	SSP	5.9953E-05	PND	1.59662E-05
SVI	0.000155689	SSV	5.9953E-05	PNT	1.59662E-05
VDT	0.000155689	VLG	5.9953E-05	PYG	1.59662E-05
WTP	0.000155689	WVL	5.9953E-05	RLM	1.59662E-05
YVP	0.000155689	FCR	5.9596E-05	RNV	1.59662E-05
ARI	0.000155255	FEQ	5.9596E-05	SAR	1.59662E-05
DVA	0.000155255	FWT	5.9596E-05	SYL	1.59662E-05
QAT	0.000155255	LIR	5.9596E-05	VQR	1.59662E-05
SAF	0.000155255	LNG	5.9596E-05	WES	1.59662E-05
SVD	0.000155255	FGE	5.9239E-05	WSW	1.59662E-05
YVA	0.000155255	FGT	5.9239E-05	WYQ	1.59662E-05
APW	0.000154821	FWM	5.9239E-05	YQA	1.59662E-05
DGA	0.000154821	LKP	5.9239E-05	AAY	1.57167E-05
DGT	0.000154821	NLM	5.9239E-05	AKM	1.57167E-05
FGP	0.000154821	SFS	5.9239E-05	ANY	1.57167E-05
GRE	0.000154821	SKS	5.9239E-05	AQR	1.57167E-05
GRF	0.000154821	TAV	5.9239E-05	DSW	1.57167E-05
GSK	0.000154821	TPL	5.9239E-05	ESA	1.57167E-05
LGF	0.000154821	WLR	5.9239E-05	FEW	1.57167E-05
QAP	0.000154821	EPS	5.8882E-05	FKF	1.57167E-05
RAI	0.000154821	GLH	5.8882E-05	FMV	1.57167E-05
RGH	0.000154821	HAV	5.8882E-05	GPP	1.57167E-05
RGQ	0.000154821	IIA	5.8882E-05	GPT	1.57167E-05
RYV	0.000154821	KDR	5.8882E-05	GYI	1.57167E-05
STY	0.000154821	KSG	5.8882E-05	IPR	1.57167E-05
SVF	0.000154821	MPR	5.8882E-05	KLP	1.57167E-05
THP	0.000154821	PYG	5.8882E-05	KVT	1.57167E-05
HVV	0.000154388	QPS	5.8882E-05	LRE	1.57167E-05
KAP	0.000154388	SNL	5.8882E-05	LWD	1.57167E-05
PHT	0.000154388	GTD	5.8525E-05	NCW	1.57167E-05
QPG	0.000154388	LRD	5.8525E-05	PAS	1.57167E-05
TTC	0.000154388	PPN	5.8525E-05	PLQ	1.57167E-05
CPP	0.000153954	RKH	5.8525E-05	PRG	1.57167E-05
EVT	0.000153954	VGL	5.8525E-05	REA	1.57167E-05
IRG	0.000153954	VLP	5.8525E-05	RLV	1.57167E-05
PKA	0.000153954	WKS	5.8525E-05	SER	1.57167E-05
PMT	0.000153954	AGQ	5.8168E-05	VRN	1.57167E-05
PTQ	0.000153954	ALH	5.8168E-05	VWG	1.57167E-05
RTF	0.000153954	ANT	5.8168E-05	WCQ	1.57167E-05

VWP	0.000153954	ATP	5.8168E-05	WIA	1.57167E-05
DTG	0.00015352	CVY	5.8168E-05	WSV	1.57167E-05
ITP	0.00015352	DHA	5.8168E-05	WWK	1.57167E-05
MRG	0.00015352	MGC	5.8168E-05	YDL	1.57167E-05
PPK	0.00015352	RRV	5.8168E-05	YGW	1.57167E-05
PTC	0.00015352	RYW	5.8168E-05	YWS	1.57167E-05
RAY	0.00015352	TGH	5.8168E-05	AEP	1.54672E-05
RGK	0.00015352	WRR	5.8168E-05	AGL	1.54672E-05
VCP	0.00015352	YLC	5.8168E-05	APN	1.54672E-05
WPP	0.00015352	LPV	5.7812E-05	ELL	1.54672E-05
WSV	0.00015352	RDH	5.7812E-05	GAD	1.54672E-05
EVV	0.000153087	SPP	5.7812E-05	GEC	1.54672E-05
HAV	0.000153087	WCG	5.7812E-05	GEG	1.54672E-05
IPP	0.000153087	WMS	5.7812E-05	GVR	1.54672E-05
ISG	0.000153087	YDR	5.7812E-05	GWS	1.54672E-05
PDP	0.000153087	ARG	5.7455E-05	KSN	1.54672E-05
SGM	0.000153087	FLW	5.7455E-05	LAY	1.54672E-05
MAR	0.000152653	GAW	5.7455E-05	LFT	1.54672E-05
MLG	0.000152653	GLT	5.7455E-05	NFS	1.54672E-05
PEP	0.000152653	MSH	5.7455E-05	NWH	1.54672E-05
VIT	0.000152653	NME	5.7455E-05	PHP	1.54672E-05
ACA	0.000152219	PWE	5.7455E-05	PIL	1.54672E-05
AHP	0.000152219	WST	5.7455E-05	PVS	1.54672E-05
GSI	0.000152219	YGS	5.7455E-05	RER	1.54672E-05
HHS	0.000152219	ILH	5.7098E-05	RGW	1.54672E-05
LCW	0.000152219	MLH	5.7098E-05	RYW	1.54672E-05
RGN	0.000152219	RFS	5.7098E-05	SQG	1.54672E-05
RTI	0.000152219	SHS	5.7098E-05	SRA	1.54672E-05
RVI	0.000152219	SLV	5.7098E-05	VKQ	1.54672E-05
TSE	0.000152219	AKL	5.6741E-05	VTW	1.54672E-05
ANA	0.000151786	FCY	5.6741E-05	ALC	1.52177E-05
FCR	0.000151786	GEA	5.6741E-05	AQA	1.52177E-05
GYT	0.000151786	GQS	5.6741E-05	CKS	1.52177E-05
LEG	0.000151786	LGW	5.6741E-05	FQM	1.52177E-05
SAI	0.000151786	LIG	5.6741E-05	GDC	1.52177E-05
SFV	0.000151786	MPS	5.6741E-05	GIC	1.52177E-05
SKG	0.000151786	QNS	5.6741E-05	GNN	1.52177E-05
TSI	0.000151786	SEY	5.6741E-05	HNG	1.52177E-05
TTM	0.000151786	STR	5.6741E-05	HSG	1.52177E-05
VFP	0.000151786	SYS	5.6741E-05	ISV	1.52177E-05
VTQ	0.000151786	ADL	5.6384E-05	KIR	1.52177E-05
ACT	0.000151352	LWP	5.6384E-05	KWL	1.52177E-05
GPW	0.000151352	NTA	5.6384E-05	LGA	1.52177E-05
SFG	0.000151352	PTD	5.6384E-05	LLF	1.52177E-05

YNR	0.000151352	RES	5.6384E-05	LYK	1.52177E-05
AHT	0.000150918	FRI	5.6027E-05	NNS	1.52177E-05
NNR	0.000150918	FWP	5.6027E-05	PFL	1.52177E-05
PQT	0.000150918	GLW	5.6027E-05	PLK	1.52177E-05
TTK	0.000150918	HRV	5.6027E-05	PPM	1.52177E-05
VNP	0.000150918	LDA	5.6027E-05	PSP	1.52177E-05
ARY	0.000150485	MFL	5.6027E-05	QMR	1.52177E-05
KAA	0.000150485	NPF	5.6027E-05	QNF	1.52177E-05
NNL	0.000150485	QSL	5.6027E-05	RPK	1.52177E-05
YNL	0.000150485	TFG	5.6027E-05	SDS	1.52177E-05
APM	0.000150051	TGD	5.6027E-05	TNR	1.52177E-05
FLM	0.000150051	FKR	5.567E-05	VGH	1.52177E-05
GPM	0.000150051	FLQ	5.567E-05	WAS	1.52177E-05
NGA	0.000150051	GFL	5.567E-05	WAY	1.52177E-05
NSC	0.000150051	GGE	5.567E-05	WEA	1.52177E-05
NVV	0.000150051	KEH	5.567E-05	YLW	1.52177E-05
TDT	0.000150051	KYP	5.567E-05	AAL	1.49683E-05
AYT	0.000149617	VRS	5.567E-05	ACS	1.49683E-05
LGD	0.000149617	WTN	5.567E-05	APV	1.49683E-05
RCG	0.000149617	GAG	5.5313E-05	CNC	1.49683E-05
RIV	0.000149617	LRE	5.5313E-05	CYP	1.49683E-05
TTH	0.000149617	NRT	5.5313E-05	DFP	1.49683E-05
DAV	0.000149184	PQP	5.5313E-05	EAQ	1.49683E-05
HDL	0.000149184	QDL	5.5313E-05	FFW	1.49683E-05
QVA	0.000149184	RWR	5.5313E-05	FRE	1.49683E-05
RFG	0.000149184	TRN	5.5313E-05	FSE	1.49683E-05
AMT	0.00014875	WSA	5.5313E-05	GCA	1.49683E-05
ATQ	0.00014875	ASI	5.4957E-05	GCF	1.49683E-05
EPT	0.00014875	DLT	5.4957E-05	GLY	1.49683E-05
HNL	0.00014875	GGV	5.4957E-05	GWG	1.49683E-05
PFT	0.00014875	LCG	5.4957E-05	MKT	1.49683E-05
WVA	0.00014875	PWI	5.4957E-05	NDR	1.49683E-05
DML	0.000148316	TLF	5.4957E-05	NES	1.49683E-05
DPG	0.000148316	VLA	5.4957E-05	NLQ	1.49683E-05
HTV	0.000148316	VTI	5.4957E-05	NRL	1.49683E-05
NDR	0.000148316	KPP	5.46E-05	PPQ	1.49683E-05
NDS	0.000148316	LVP	5.46E-05	QKP	1.49683E-05
NLK	0.000148316	PCN	5.46E-05	SAL	1.49683E-05
TDP	0.000148316	PNT	5.46E-05	SEQ	1.49683E-05
THA	0.000148316	PTG	5.46E-05	SLC	1.49683E-05
AHA	0.000147882	RPG	5.46E-05	SLF	1.49683E-05
DAA	0.000147882	SRH	5.46E-05	SQS	1.49683E-05
NIL	0.000147882	TPE	5.46E-05	VCT	1.49683E-05
PAW	0.000147882	VHA	5.46E-05	WEG	1.49683E-05

PFA	0.000147882	FYH	5.4243E-05	WPY	1.49683E-05
SGN	0.000147882	KCG	5.4243E-05	WRT	1.49683E-05
SPF	0.000147882	PID	5.4243E-05	YDH	1.49683E-05
TNP	0.000147882	QRS	5.4243E-05	AIS	1.47188E-05
DDL	0.000147449	QTL	5.4243E-05	AKA	1.47188E-05
NNS	0.000147449	VRF	5.4243E-05	AYL	1.47188E-05
PTW	0.000147449	AGE	5.3886E-05	DLR	1.47188E-05
TIP	0.000147449	CST	5.3886E-05	DSG	1.47188E-05
TSN	0.000147449	FHQ	5.3886E-05	GIS	1.47188E-05
TTQ	0.000147449	FYL	5.3886E-05	GRP	1.47188E-05
VTW	0.000147449	GNM	5.3886E-05	ISF	1.47188E-05
VVM	0.000147449	INA	5.3886E-05	KNC	1.47188E-05
IAT	0.000147015	PWL	5.3886E-05	KRG	1.47188E-05
PPN	0.000147015	SAR	5.3886E-05	KYP	1.47188E-05
PPY	0.000147015	SLI	5.3886E-05	LCE	1.47188E-05
SGK	0.000147015	WES	5.3886E-05	LHF	1.47188E-05
WVT	0.000147015	EPC	5.3529E-05	PNF	1.47188E-05
YGT	0.000147015	FDA	5.3529E-05	RAT	1.47188E-05
DVG	0.000146581	FYR	5.3529E-05	RCL	1.47188E-05
NAG	0.000146581	GTY	5.3529E-05	RCS	1.47188E-05
PIP	0.000146581	HYT	5.3529E-05	RHD	1.47188E-05
PMP	0.000146581	LAT	5.3529E-05	RQC	1.47188E-05
RVF	0.000146581	LCA	5.3529E-05	RSW	1.47188E-05
TRF	0.000146581	LMR	5.3529E-05	RWI	1.47188E-05
YLM	0.000146581	LTQ	5.3529E-05	SNG	1.47188E-05
FAP	0.000146148	MLV	5.3529E-05	TMT	1.47188E-05
HGP	0.000146148	NAT	5.3529E-05	TPA	1.47188E-05
MPT	0.000146148	PSH	5.3529E-05	VSN	1.47188E-05
PCT	0.000146148	RFD	5.3529E-05	VSV	1.47188E-05
PMA	0.000146148	WLA	5.3529E-05	WVD	1.47188E-05
ADT	0.000145714	WWL	5.3529E-05	ARI	1.44693E-05
MGS	0.000145714	CLR	5.3172E-05	ATL	1.44693E-05
PAQ	0.000145714	FGV	5.3172E-05	ATM	1.44693E-05
PCA	0.000145714	GYA	5.3172E-05	CYV	1.44693E-05
PPI	0.000145714	HLG	5.3172E-05	EDC	1.44693E-05
PYA	0.000145714	LIL	5.3172E-05	EDL	1.44693E-05
SEV	0.000145714	MSP	5.3172E-05	FGF	1.44693E-05
VMP	0.000145714	NTT	5.3172E-05	FQH	1.44693E-05
VYP	0.000145714	PGW	5.3172E-05	GLK	1.44693E-05
AKA	0.00014528	RHA	5.3172E-05	GLQ	1.44693E-05
FNL	0.00014528	VTC	5.3172E-05	GMF	1.44693E-05
GSF	0.00014528	ASV	5.2815E-05	GTI	1.44693E-05
IPT	0.00014528	DEQ	5.2815E-05	HYS	1.44693E-05
QVT	0.00014528	IRA	5.2815E-05	IHS	1.44693E-05

GKA	0.000144847	ITK	5.2815E-05	IQR	1.44693E-05
HYL	0.000144847	KYT	5.2815E-05	ISM	1.44693E-05
KDS	0.000144847	NLE	5.2815E-05	IWV	1.44693E-05
RGE	0.000144847	NSG	5.2815E-05	KYT	1.44693E-05
SGD	0.000144847	PSN	5.2815E-05	LDQ	1.44693E-05
VPW	0.000144847	TND	5.2815E-05	LKP	1.44693E-05
YLC	0.000144847	VSV	5.2815E-05	LWE	1.44693E-05
ASI	0.000144413	WMF	5.2815E-05	MRS	1.44693E-05
CTP	0.000144413	YKR	5.2815E-05	MSS	1.44693E-05
EVA	0.000144413	GEY	5.2459E-05	PAN	1.44693E-05
GFA	0.000144413	KCH	5.2459E-05	PSG	1.44693E-05
GMT	0.000144413	KRL	5.2459E-05	QPD	1.44693E-05
KGP	0.000144413	KWV	5.2459E-05	QYG	1.44693E-05
RYG	0.000144413	NHA	5.2459E-05	RDL	1.44693E-05
VKP	0.000144413	PGT	5.2459E-05	REP	1.44693E-05
ATC	0.000143979	PHV	5.2459E-05	RMQ	1.44693E-05
DNS	0.000143979	SNS	5.2459E-05	RNN	1.44693E-05
FVG	0.000143979	ELF	5.2102E-05	RRT	1.44693E-05
GDA	0.000143979	FES	5.2102E-05	RVH	1.44693E-05
GTC	0.000143979	FLF	5.2102E-05	VPP	1.44693E-05
PSF	0.000143979	GRY	5.2102E-05	WNT	1.44693E-05
RPF	0.000143979	IAR	5.2102E-05	WRD	1.44693E-05
RVY	0.000143979	IGL	5.2102E-05	WSK	1.44693E-05
TPD	0.000143979	ITL	5.2102E-05	WWG	1.44693E-05
WGP	0.000143979	IWL	5.2102E-05	YNL	1.44693E-05
YLW	0.000143979	KCD	5.2102E-05	YVR	1.44693E-05
EAT	0.000143546	RAG	5.2102E-05	AGQ	1.42199E-05
KGA	0.000143546	RMS	5.2102E-05	AHM	1.42199E-05
NHL	0.000143546	TIV	5.2102E-05	AHR	1.42199E-05
SQG	0.000143546	TPP	5.2102E-05	APG	1.42199E-05
TQT	0.000143546	YTR	5.2102E-05	ASM	1.42199E-05
VAC	0.000143546	ATR	5.1745E-05	ASN	1.42199E-05
WPT	0.000143546	EES	5.1745E-05	ATR	1.42199E-05
FVP	0.000143112	ISG	5.1745E-05	CLM	1.42199E-05
NDL	0.000143112	KRR	5.1745E-05	CLS	1.42199E-05
PAH	0.000143112	LHA	5.1745E-05	EDH	1.42199E-05
PSY	0.000143112	PPF	5.1745E-05	EGQ	1.42199E-05
RIG	0.000143112	QYR	5.1745E-05	ESR	1.42199E-05
TIT	0.000143112	RVR	5.1745E-05	EYT	1.42199E-05
TPE	0.000143112	TAS	5.1745E-05	FPC	1.42199E-05
VTM	0.000143112	TEL	5.1745E-05	GPG	1.42199E-05
YDS	0.000143112	TES	5.1745E-05	KYA	1.42199E-05
ATW	0.000142678	WTR	5.1745E-05	LMT	1.42199E-05
EAA	0.000142678	ADA	5.1388E-05	LQW	1.42199E-05

FPG	0.000142678	FSM	5.1388E-05	LTQ	1.42199E-05
HDR	0.000142678	GIA	5.1388E-05	MTR	1.42199E-05
KNS	0.000142678	GYL	5.1388E-05	NSW	1.42199E-05
KVP	0.000142678	PNS	5.1388E-05	NVN	1.42199E-05
NTG	0.000142678	TSV	5.1388E-05	PRC	1.42199E-05
TQP	0.000142678	WAH	5.1388E-05	RSM	1.42199E-05
YDL	0.000142678	YSF	5.1388E-05	SMM	1.42199E-05
YVV	0.000142678	GAV	5.1031E-05	SSH	1.42199E-05
HIL	0.000142245	GMS	5.1031E-05	TTY	1.42199E-05
KNR	0.000142245	HIE	5.1031E-05	VHG	1.42199E-05
NHS	0.000142245	IAH	5.1031E-05	VKN	1.42199E-05
NMS	0.000142245	IRH	5.1031E-05	WFD	1.42199E-05
PEA	0.000142245	RGG	5.1031E-05	WIV	1.42199E-05
TNA	0.000142245	RGH	5.1031E-05	WRE	1.42199E-05
VHA	0.000142245	TTC	5.1031E-05	WTQ	1.42199E-05
VTE	0.000142245	WNI	5.1031E-05	WYD	1.42199E-05
WTA	0.000142245	YFS	5.1031E-05	WYT	1.42199E-05
YPG	0.000142245	AQL	5.0674E-05	YHT	1.42199E-05
ATM	0.000141811	FNS	5.0674E-05	YTT	1.42199E-05
EGT	0.000141811	GLV	5.0674E-05	CWV	1.39704E-05
FGV	0.000141811	PFH	5.0674E-05	DLA	1.39704E-05
KHS	0.000141811	RGE	5.0674E-05	FMT	1.39704E-05
KMR	0.000141811	VSR	5.0674E-05	FPT	1.39704E-05
KVG	0.000141811	EHS	5.0317E-05	GCW	1.39704E-05
MSG	0.000141811	FRN	5.0317E-05	GDP	1.39704E-05
RGY	0.000141811	GHC	5.0317E-05	GRD	1.39704E-05
RPI	0.000141811	HSS	5.0317E-05	IER	1.39704E-05
SWV	0.000141811	LDF	5.0317E-05	LNN	1.39704E-05
TVM	0.000141811	LQL	5.0317E-05	NAL	1.39704E-05
TYT	0.000141811	PDT	5.0317E-05	NSH	1.39704E-05
VDA	0.000141811	PRI	5.0317E-05	NSP	1.39704E-05
DDS	0.000141377	RAE	5.0317E-05	NTC	1.39704E-05
GTQ	0.000141377	TNA	5.0317E-05	PFH	1.39704E-05
KPG	0.000141377	ELV	4.9961E-05	PFS	1.39704E-05
PVC	0.000141377	FPF	4.9961E-05	RFN	1.39704E-05
SGY	0.000141377	GCV	4.9961E-05	RGE	1.39704E-05
TKP	0.000141377	GSY	4.9961E-05	RWK	1.39704E-05
VDV	0.000141377	GYS	4.9961E-05	SGE	1.39704E-05
YAV	0.000141377	IHL	4.9961E-05	SGG	1.39704E-05
ATH	0.000140944	ISE	4.9961E-05	SQR	1.39704E-05
FVA	0.000140944	RDG	4.9961E-05	VSA	1.39704E-05
NLW	0.000140944	TLY	4.9961E-05	WKP	1.39704E-05
RWV	0.000140944	VEL	4.9961E-05	WQA	1.39704E-05
VAM	0.000140944	WAD	4.9961E-05	YWC	1.39704E-05

YAA	0.000140944	WTK	4.9961E-05	CRG	1.37209E-05
YDR	0.000140944	YDC	4.9961E-05	CRQ	1.37209E-05
YHS	0.000140944	ANS	4.9604E-05	CYG	1.37209E-05
DCL	0.00014051	AWY	4.9604E-05	DLI	1.37209E-05
DMS	0.00014051	NGH	4.9604E-05	EKQ	1.37209E-05
FSM	0.00014051	PAD	4.9604E-05	FKG	1.37209E-05
GDT	0.00014051	PFS	4.9604E-05	FKV	1.37209E-05
GKT	0.00014051	PQS	4.9604E-05	FLC	1.37209E-05
HGG	0.00014051	PYS	4.9604E-05	GEE	1.37209E-05
HHR	0.00014051	RFT	4.9604E-05	GHR	1.37209E-05
HLC	0.00014051	RPI	4.9604E-05	HAH	1.37209E-05
HNR	0.00014051	SAE	4.9604E-05	IEQ	1.37209E-05
HNS	0.00014051	SDD	4.9604E-05	IRP	1.37209E-05
KNL	0.00014051	SDL	4.9604E-05	KHR	1.37209E-05
PYT	0.00014051	TAP	4.9604E-05	KRR	1.37209E-05
VIP	0.00014051	WDH	4.9604E-05	LYF	1.37209E-05
VQT	0.00014051	AWL	4.9247E-05	LYQ	1.37209E-05
DSM	0.000140076	EPH	4.9247E-05	NHR	1.37209E-05
GTF	0.000140076	ERS	4.9247E-05	NRR	1.37209E-05
GTM	0.000140076	HPA	4.9247E-05	NTH	1.37209E-05
PDA	0.000140076	ISH	4.9247E-05	PCT	1.37209E-05
VPN	0.000140076	LAW	4.9247E-05	PHL	1.37209E-05
YAG	0.000140076	LQT	4.9247E-05	QDG	1.37209E-05
YTV	0.000140076	MSA	4.9247E-05	RAR	1.37209E-05
CTT	0.000139643	NLL	4.9247E-05	RHY	1.37209E-05
DGP	0.000139643	PIT	4.9247E-05	RYC	1.37209E-05
DMR	0.000139643	PQL	4.9247E-05	SLD	1.37209E-05
DNR	0.000139643	QPL	4.9247E-05	SVL	1.37209E-05
DYR	0.000139643	QSA	4.9247E-05	TSL	1.37209E-05
GSY	0.000139643	RKA	4.9247E-05	VSW	1.37209E-05
HRC	0.000139643	SRE	4.9247E-05	WAL	1.37209E-05
PTE	0.000139643	AAS	4.889E-05	WPP	1.37209E-05
TSF	0.000139643	KKT	4.889E-05	YLC	1.37209E-05
VDP	0.000139643	NGA	4.889E-05	ASD	1.34714E-05
WVP	0.000139643	PAI	4.889E-05	ELS	1.34714E-05
IGT	0.000139209	PLD	4.889E-05	FRD	1.34714E-05
KYS	0.000139209	QFL	4.889E-05	GMY	1.34714E-05
TIA	0.000139209	RPV	4.889E-05	IAR	1.34714E-05
AKP	0.000138775	RQR	4.889E-05	KMV	1.34714E-05
ENR	0.000138775	TCG	4.889E-05	LDP	1.34714E-05
HAG	0.000138775	ALG	4.8533E-05	LTV	1.34714E-05
HCL	0.000138775	EGH	4.8533E-05	MGL	1.34714E-05
IVA	0.000138775	GMH	4.8533E-05	MST	1.34714E-05
NLN	0.000138775	HTP	4.8533E-05	NWP	1.34714E-05

PAC	0.000138775	MFS	4.8533E-05	NYH	1.34714E-05
TMP	0.000138775	MLI	4.8533E-05	PDW	1.34714E-05
DKS	0.000138342	PVR	4.8533E-05	PMC	1.34714E-05
FAA	0.000138342	QVA	4.8533E-05	PML	1.34714E-05
GET	0.000138342	WIL	4.8533E-05	PRT	1.34714E-05
GNA	0.000138342	ILS	4.8176E-05	QNQ	1.34714E-05
HVG	0.000138342	LRK	4.8176E-05	QRL	1.34714E-05
LGE	0.000138342	MFT	4.8176E-05	RIY	1.34714E-05
LGY	0.000138342	MYK	4.8176E-05	RPC	1.34714E-05
NKL	0.000138342	NNG	4.8176E-05	SLK	1.34714E-05
NSM	0.000138342	NRH	4.8176E-05	SSN	1.34714E-05
NSW	0.000138342	PAW	4.8176E-05	STN	1.34714E-05
PFP	0.000138342	PDL	4.8176E-05	SYP	1.34714E-05
PGQ	0.000138342	QGA	4.8176E-05	TKR	1.34714E-05
PIT	0.000138342	SRY	4.8176E-05	TSS	1.34714E-05
SGH	0.000138342	TFV	4.8176E-05	VMG	1.34714E-05
TSY	0.000138342	VLR	4.8176E-05	VNY	1.34714E-05
APN	0.000137908	YNA	4.8176E-05	VWF	1.34714E-05
ASF	0.000137908	YSV	4.8176E-05	WWN	1.34714E-05
FML	0.000137908	AAA	4.7819E-05	YKH	1.34714E-05
GMA	0.000137908	CRL	4.7819E-05	YRS	1.34714E-05
KKR	0.000137908	DSR	4.7819E-05	YWW	1.34714E-05
PCP	0.000137908	FWV	4.7819E-05	ANT	1.3222E-05
TFT	0.000137908	GAF	4.7819E-05	ARF	1.3222E-05
VVC	0.000137908	PEW	4.7819E-05	DLP	1.3222E-05
VVW	0.000137908	PMQ	4.7819E-05	ETR	1.3222E-05
ACP	0.000137474	RDT	4.7819E-05	FEV	1.3222E-05
DCS	0.000137474	RNW	4.7819E-05	FFC	1.3222E-05
FLE	0.000137474	STN	4.7819E-05	FFT	1.3222E-05
FNS	0.000137474	CLV	4.7463E-05	FHQ	1.3222E-05
FPV	0.000137474	DGA	4.7463E-05	FLK	1.3222E-05
HIR	0.000137474	FAL	4.7463E-05	GGA	1.3222E-05
NEL	0.000137474	FGG	4.7463E-05	GQD	1.3222E-05
PWT	0.000137474	GRE	4.7463E-05	GRM	1.3222E-05
VHT	0.000137474	HSG	4.7463E-05	HYG	1.3222E-05
DFL	0.000137041	IIG	4.7463E-05	ILQ	1.3222E-05
DHR	0.000137041	IPQ	4.7463E-05	KTP	1.3222E-05
FAV	0.000137041	LHR	4.7463E-05	LLI	1.3222E-05
GMP	0.000137041	LPY	4.7463E-05	NGT	1.3222E-05
GNP	0.000137041	PET	4.7463E-05	NSG	1.3222E-05
HCR	0.000137041	PSC	4.7463E-05	PFC	1.3222E-05
KLM	0.000137041	PTK	4.7463E-05	PFD	1.3222E-05
NYR	0.000137041	QRD	4.7463E-05	PPK	1.3222E-05
PKP	0.000137041	RPE	4.7463E-05	PSF	1.3222E-05

PPF	0.000137041	RSV	4.7463E-05	PTL	1.3222E-05
REV	0.000137041	EGQ	4.7106E-05	PYT	1.3222E-05
TQA	0.000137041	ESM	4.7106E-05	QTG	1.3222E-05
VET	0.000137041	FLI	4.7106E-05	QWN	1.3222E-05
VTH	0.000137041	FPQ	4.7106E-05	RLK	1.3222E-05
VWT	0.000137041	GAH	4.7106E-05	RLW	1.3222E-05
AQT	0.000136607	GAT	4.7106E-05	RRD	1.3222E-05
AVM	0.000136607	GLF	4.7106E-05	SDH	1.3222E-05
DKL	0.000136607	HTL	4.7106E-05	SES	1.3222E-05
DLC	0.000136607	KPH	4.7106E-05	SHT	1.3222E-05
EDL	0.000136607	KTT	4.7106E-05	SNA	1.3222E-05
GNV	0.000136607	LNY	4.7106E-05	SNH	1.3222E-05
HEL	0.000136607	LRF	4.7106E-05	SRI	1.3222E-05
MSV	0.000136607	MGL	4.7106E-05	STH	1.3222E-05
SAY	0.000136607	MRA	4.7106E-05	TGL	1.3222E-05
SEG	0.000136607	MRS	4.7106E-05	VCG	1.3222E-05
TDA	0.000136607	NLN	4.7106E-05	VDS	1.3222E-05
VNV	0.000136607	AGD	4.6749E-05	VKC	1.3222E-05
WAT	0.000136607	DRF	4.6749E-05	VRC	1.3222E-05
YLK	0.000136607	GNR	4.6749E-05	VSH	1.3222E-05
DLW	0.000136173	ISP	4.6749E-05	WNV	1.3222E-05
FGG	0.000136173	KLD	4.6749E-05	YYV	1.3222E-05
GTD	0.000136173	KWF	4.6749E-05	AHQ	1.29725E-05
HLW	0.000136173	LTW	4.6749E-05	AMT	1.29725E-05
HMS	0.000136173	PFA	4.6749E-05	CSG	1.29725E-05
HSW	0.000136173	QAR	4.6749E-05	ENG	1.29725E-05
NCS	0.000136173	QFH	4.6749E-05	EPA	1.29725E-05
PGW	0.000136173	TPV	4.6749E-05	FKI	1.29725E-05
SWG	0.000136173	TTL	4.6749E-05	FNW	1.29725E-05
TAQ	0.000136173	ATL	4.6392E-05	GEV	1.29725E-05
VKV	0.000136173	DRP	4.6392E-05	GFT	1.29725E-05
VPD	0.000136173	FAC	4.6392E-05	GGK	1.29725E-05
VPQ	0.000136173	FAT	4.6392E-05	GHT	1.29725E-05
YNS	0.000136173	FCS	4.6392E-05	GRI	1.29725E-05
GTW	0.00013574	FPS	4.6392E-05	GWH	1.29725E-05
KTG	0.00013574	NSW	4.6392E-05	GWQ	1.29725E-05
MVT	0.00013574	PCA	4.6392E-05	ILP	1.29725E-05
NHR	0.00013574	PFP	4.6392E-05	INL	1.29725E-05
RQG	0.00013574	RLD	4.6392E-05	KEY	1.29725E-05
VCV	0.00013574	RNN	4.6392E-05	KPA	1.29725E-05
AAH	0.000135306	RPF	4.6392E-05	LDA	1.29725E-05
ATE	0.000135306	SDR	4.6392E-05	LNY	1.29725E-05
DIR	0.000135306	TGV	4.6392E-05	MMT	1.29725E-05
EGA	0.000135306	TYA	4.6392E-05	NHA	1.29725E-05

GWP	0.000135306	VEH	4.6392E-05	NSY	1.29725E-05
HYR	0.000135306	CSY	4.6035E-05	PCG	1.29725E-05
IVT	0.000135306	GAE	4.6035E-05	PHD	1.29725E-05
KIL	0.000135306	GKH	4.6035E-05	RCT	1.29725E-05
NYS	0.000135306	GKI	4.6035E-05	REN	1.29725E-05
TCT	0.000135306	KRG	4.6035E-05	RLY	1.29725E-05
WLC	0.000135306	LVS	4.6035E-05	SML	1.29725E-05
WPA	0.000135306	NFW	4.6035E-05	SRC	1.29725E-05
ADG	0.000134872	RFH	4.6035E-05	SYT	1.29725E-05
DHS	0.000134872	VGV	4.6035E-05	VER	1.29725E-05
DIL	0.000134872	EEH	4.5678E-05	VPC	1.29725E-05
FKL	0.000134872	HNA	4.5678E-05	WHF	1.29725E-05
GFP	0.000134872	HSP	4.5678E-05	WQW	1.29725E-05
HCS	0.000134872	KHS	4.5678E-05	YRA	1.29725E-05
HKS	0.000134872	LLF	4.5678E-05	YSQ	1.29725E-05
HLE	0.000134872	NTS	4.5678E-05	AHY	1.2723E-05
KAV	0.000134872	RVG	4.5678E-05	ASR	1.2723E-05
KGG	0.000134872	TLC	4.5678E-05	DFT	1.2723E-05
KPV	0.000134872	VGT	4.5678E-05	DHA	1.2723E-05
TWT	0.000134872	VLM	4.5678E-05	EPD	1.2723E-05
YHL	0.000134872	YPL	4.5678E-05	FLH	1.2723E-05
YIL	0.000134872	EAR	4.5321E-05	FNA	1.2723E-05
ADP	0.000134439	FMD	4.5321E-05	FNC	1.2723E-05
CSG	0.000134439	HSD	4.5321E-05	FQW	1.2723E-05
CVT	0.000134439	LEP	4.5321E-05	GGE	1.2723E-05
DNL	0.000134439	LGH	4.5321E-05	GHN	1.2723E-05
EGP	0.000134439	MGR	4.5321E-05	GQQ	1.2723E-05
GDP	0.000134439	NLR	4.5321E-05	HRL	1.2723E-05
GQA	0.000134439	QDW	4.5321E-05	LDC	1.2723E-05
GTH	0.000134439	QPP	4.5321E-05	LEC	1.2723E-05
HMR	0.000134439	RHL	4.5321E-05	LGD	1.2723E-05
NLQ	0.000134439	RTM	4.5321E-05	LMC	1.2723E-05
QGP	0.000134439	TRQ	4.5321E-05	LYD	1.2723E-05
VYA	0.000134439	WIK	4.5321E-05	MWR	1.2723E-05
AWT	0.000134005	WRH	4.5321E-05	MWT	1.2723E-05
DAG	0.000134005	YHS	4.5321E-05	NMK	1.2723E-05
NKS	0.000134005	CLL	4.4965E-05	NNG	1.2723E-05
VGM	0.000134005	EGA	4.4965E-05	PPF	1.2723E-05
YHR	0.000134005	ETS	4.4965E-05	PQR	1.2723E-05
FYL	0.000133571	GIP	4.4965E-05	PYR	1.2723E-05
ITA	0.000133571	IRP	4.4965E-05	QCW	1.2723E-05
PIA	0.000133571	KGS	4.4965E-05	QTN	1.2723E-05
QKS	0.000133571	LEL	4.4965E-05	RHL	1.2723E-05
RWG	0.000133571	LNC	4.4965E-05	SGT	1.2723E-05

TPK	0.000133571	LWV	4.4965E-05	SIR	1.2723E-05
VIV	0.000133571	NPT	4.4965E-05	STD	1.2723E-05
VMA	0.000133571	PHS	4.4965E-05	VEP	1.2723E-05
AFT	0.000133138	SWK	4.4965E-05	VLV	1.2723E-05
AIA	0.000133138	TCE	4.4965E-05	VPH	1.2723E-05
ATK	0.000133138	TKT	4.4965E-05	WFT	1.2723E-05
DHL	0.000133138	WSR	4.4965E-05	WNA	1.2723E-05
DRM	0.000133138	ENT	4.4608E-05	WRA	1.2723E-05
FHS	0.000133138	EPA	4.4608E-05	YLI	1.2723E-05
HIS	0.000133138	ETL	4.4608E-05	AAP	1.24736E-05
HLK	0.000133138	GSC	4.4608E-05	AHL	1.24736E-05
KKL	0.000133138	GVA	4.4608E-05	ALW	1.24736E-05
KLW	0.000133138	KGD	4.4608E-05	ARD	1.24736E-05
LNC	0.000133138	MTA	4.4608E-05	ARV	1.24736E-05
NCL	0.000133138	PIQ	4.4608E-05	ATA	1.24736E-05
NFL	0.000133138	QRI	4.4608E-05	CLV	1.24736E-05
TDV	0.000133138	SYL	4.4608E-05	EHR	1.24736E-05
TMA	0.000133138	TSM	4.4608E-05	FIG	1.24736E-05
WLM	0.000133138	VWR	4.4608E-05	FIS	1.24736E-05
YCL	0.000133138	AMK	4.4251E-05	FPH	1.24736E-05
YSM	0.000133138	EDS	4.4251E-05	GFF	1.24736E-05
AEP	0.000132704	GNN	4.4251E-05	GQC	1.24736E-05
CVA	0.000132704	TCY	4.4251E-05	GSM	1.24736E-05
FDL	0.000132704	TFA	4.4251E-05	HNP	1.24736E-05
FKR	0.000132704	TSD	4.4251E-05	HWR	1.24736E-05
FRQ	0.000132704	VVI	4.4251E-05	ISC	1.24736E-05
GVQ	0.000132704	YDA	4.4251E-05	IVS	1.24736E-05
HFR	0.000132704	YNC	4.4251E-05	LEP	1.24736E-05
HLH	0.000132704	FFR	4.3894E-05	LPN	1.24736E-05
KLC	0.000132704	GDR	4.3894E-05	LQG	1.24736E-05
NAV	0.000132704	LMQ	4.3894E-05	PIT	1.24736E-05
PDT	0.000132704	LSI	4.3894E-05	PKT	1.24736E-05
PTK	0.000132704	MTL	4.3894E-05	PMM	1.24736E-05
QMR	0.000132704	NLG	4.3894E-05	PNL	1.24736E-05
VGQ	0.000132704	PHH	4.3894E-05	PPW	1.24736E-05
VMV	0.000132704	PLM	4.3894E-05	PWM	1.24736E-05
VVE	0.000132704	QRG	4.3894E-05	RNY	1.24736E-05
CVV	0.00013227	RDM	4.3894E-05	RSE	1.24736E-05
DLN	0.00013227	RSW	4.3894E-05	SFG	1.24736E-05
FDR	0.00013227	SYG	4.3894E-05	TTP	1.24736E-05
HSQ	0.00013227	TDA	4.3894E-05	VNP	1.24736E-05
TAC	0.00013227	WLH	4.3894E-05	WVT	1.24736E-05
TET	0.00013227	ACA	4.3537E-05	WVW	1.24736E-05
TFA	0.00013227	GMP	4.3537E-05	WYF	1.24736E-05

VAW	0.00013227	LKH	4.3537E-05	WYN	1.24736E-05
YGA	0.00013227	LWW	4.3537E-05	YRW	1.24736E-05
APD	0.000131837	NRV	4.3537E-05	AAH	1.22241E-05
CAT	0.000131837	PVA	4.3537E-05	ACP	1.22241E-05
DDR	0.000131837	PVT	4.3537E-05	ANC	1.22241E-05
EAG	0.000131837	QHS	4.3537E-05	DKR	1.22241E-05
FTV	0.000131837	RTY	4.3537E-05	EAH	1.22241E-05
GHT	0.000131837	SHD	4.3537E-05	ESP	1.22241E-05
IAP	0.000131837	SRF	4.3537E-05	FMC	1.22241E-05
IVV	0.000131837	TCL	4.3537E-05	FQQ	1.22241E-05
NYL	0.000131837	TPW	4.3537E-05	FTN	1.22241E-05
PTD	0.000131837	AAP	4.318E-05	GDR	1.22241E-05
PVW	0.000131837	AMP	4.318E-05	GDY	1.22241E-05
PWA	0.000131837	ANL	4.318E-05	GNQ	1.22241E-05
VEP	0.000131837	AQA	4.318E-05	GQI	1.22241E-05
WGT	0.000131837	DGR	4.318E-05	HWW	1.22241E-05
ATD	0.000131403	DRT	4.318E-05	HYC	1.22241E-05
AYP	0.000131403	GMQ	4.318E-05	IRQ	1.22241E-05
FLC	0.000131403	IPR	4.318E-05	KKG	1.22241E-05
FLW	0.000131403	KMV	4.318E-05	KVR	1.22241E-05
HGV	0.000131403	KSH	4.318E-05	NVQ	1.22241E-05
HHL	0.000131403	LLC	4.318E-05	PFR	1.22241E-05
KDL	0.000131403	LPW	4.318E-05	RPQ	1.22241E-05
NGV	0.000131403	MPL	4.318E-05	RQR	1.22241E-05
VAQ	0.000131403	SKT	4.318E-05	RTK	1.22241E-05
VWV	0.000131403	TKI	4.318E-05	RYQ	1.22241E-05
YGG	0.000131403	WHH	4.318E-05	SHQ	1.22241E-05
AAC	0.000130969	APH	4.2823E-05	SKY	1.22241E-05
AGM	0.000130969	DLR	4.2823E-05	TFT	1.22241E-05
ANV	0.000130969	DSS	4.2823E-05	TKS	1.22241E-05
DLQ	0.000130969	GKV	4.2823E-05	TKT	1.22241E-05
ETV	0.000130969	LWH	4.2823E-05	TLA	1.22241E-05
FFS	0.000130969	MAI	4.2823E-05	TRL	1.22241E-05
GDG	0.000130969	NAC	4.2823E-05	TRS	1.22241E-05
GTN	0.000130969	PEA	4.2823E-05	TVR	1.22241E-05
IAA	0.000130969	RFG	4.2823E-05	VGM	1.22241E-05
KIR	0.000130969	RIH	4.2823E-05	VHA	1.22241E-05
KSM	0.000130969	RRD	4.2823E-05	VIR	1.22241E-05
NRM	0.000130969	SGK	4.2823E-05	VMK	1.22241E-05
PHG	0.000130969	SPQ	4.2823E-05	VMN	1.22241E-05
SQV	0.000130969	VRN	4.2823E-05	WAK	1.22241E-05
TKA	0.000130969	ALI	4.2466E-05	WHW	1.22241E-05
VHP	0.000130969	GVH	4.2466E-05	ANA	1.19746E-05
VNA	0.000130969	GWR	4.2466E-05	AYH	1.19746E-05

VPC	0.000130969	IGR	4.2466E-05	DMR	1.19746E-05
YSW	0.000130969	LGN	4.2466E-05	FYF	1.19746E-05
AIP	0.000130536	LPD	4.2466E-05	GCD	1.19746E-05
CTA	0.000130536	NFR	4.2466E-05	GFS	1.19746E-05
EAP	0.000130536	NKA	4.2466E-05	GGS	1.19746E-05
FDS	0.000130536	PDP	4.2466E-05	GHD	1.19746E-05
GIA	0.000130536	PGD	4.2466E-05	GMC	1.19746E-05
GQT	0.000130536	PHD	4.2466E-05	GMT	1.19746E-05
GWT	0.000130536	PMS	4.2466E-05	GNM	1.19746E-05
HDS	0.000130536	RNH	4.2466E-05	GQR	1.19746E-05
KML	0.000130536	SIA	4.2466E-05	KSH	1.19746E-05
NKR	0.000130536	ANH	4.211E-05	KTG	1.19746E-05
TYA	0.000130536	DLP	4.211E-05	LTG	1.19746E-05
VGW	0.000130536	FSQ	4.211E-05	NKS	1.19746E-05
FMS	0.000130102	HRA	4.211E-05	NNP	1.19746E-05
GCT	0.000130102	IIE	4.211E-05	NYR	1.19746E-05
KAG	0.000130102	IKP	4.211E-05	PAH	1.19746E-05
KHL	0.000130102	KFS	4.211E-05	PFF	1.19746E-05
KVV	0.000130102	KLY	4.211E-05	PGR	1.19746E-05
NLE	0.000130102	KPD	4.211E-05	PQH	1.19746E-05
SGF	0.000130102	LLW	4.211E-05	QGQ	1.19746E-05
TAW	0.000130102	RHS	4.211E-05	QHD	1.19746E-05
TFP	0.000130102	RKM	4.211E-05	QHL	1.19746E-05
TMG	0.000130102	RYL	4.211E-05	QKG	1.19746E-05
TYP	0.000130102	RYP	4.211E-05	QSY	1.19746E-05
VAK	0.000130102	SSQ	4.211E-05	RGS	1.19746E-05
VFA	0.000130102	TRI	4.211E-05	RRY	1.19746E-05
VTY	0.000130102	YLG	4.211E-05	SDG	1.19746E-05
WGQ	0.000130102	AER	4.1753E-05	SEP	1.19746E-05
DLK	0.000129668	ATT	4.1753E-05	SGW	1.19746E-05
ETG	0.000129668	GGG	4.1753E-05	SNF	1.19746E-05
GIT	0.000129668	LLN	4.1753E-05	SRD	1.19746E-05
HKL	0.000129668	MHD	4.1753E-05	TLW	1.19746E-05
HKR	0.000129668	PAH	4.1753E-05	VAQ	1.19746E-05
KDR	0.000129668	PER	4.1753E-05	VGV	1.19746E-05
KHR	0.000129668	PNF	4.1753E-05	VGW	1.19746E-05
NRW	0.000129668	RCN	4.1753E-05	VHL	1.19746E-05
QNL	0.000129668	RLF	4.1753E-05	VKA	1.19746E-05
TTN	0.000129668	TYL	4.1753E-05	VWC	1.19746E-05
YPV	0.000129668	FPT	4.1396E-05	WFH	1.19746E-05
YVG	0.000129668	GGF	4.1396E-05	YEP	1.19746E-05
DSC	0.000129235	HLS	4.1396E-05	YYH	1.19746E-05
FAG	0.000129235	QSC	4.1396E-05	CLT	1.17251E-05
FLQ	0.000129235	SDG	4.1396E-05	CPL	1.17251E-05

FMR	0.000129235	TPD	4.1396E-05	DES	1.17251E-05
FSQ	0.000129235	ADQ	4.1039E-05	DYC	1.17251E-05
GTK	0.000129235	ASD	4.1039E-05	EKA	1.17251E-05
HLQ	0.000129235	ERR	4.1039E-05	GKM	1.17251E-05
IGP	0.000129235	GDP	4.1039E-05	GQV	1.17251E-05
MTT	0.000129235	GTQ	4.1039E-05	HDR	1.17251E-05
NWL	0.000129235	INN	4.1039E-05	ICG	1.17251E-05
PMG	0.000129235	LCP	4.1039E-05	ICT	1.17251E-05
QNS	0.000129235	LDQ	4.1039E-05	ITW	1.17251E-05
VGC	0.000129235	LRM	4.1039E-05	KQH	1.17251E-05
VKA	0.000129235	LVH	4.1039E-05	KYS	1.17251E-05
WNL	0.000129235	NAH	4.1039E-05	LHN	1.17251E-05
YML	0.000129235	PLK	4.1039E-05	LLY	1.17251E-05
AEA	0.000128801	PMP	4.1039E-05	NKL	1.17251E-05
AMA	0.000128801	QTT	4.1039E-05	PMD	1.17251E-05
CPT	0.000128801	TGG	4.1039E-05	PPI	1.17251E-05
DEL	0.000128801	TLM	4.1039E-05	PSE	1.17251E-05
DGV	0.000128801	VDA	4.1039E-05	QQR	1.17251E-05
FEL	0.000128801	WVR	4.1039E-05	RAP	1.17251E-05
GAD	0.000128801	DAS	4.0682E-05	RES	1.17251E-05
GPH	0.000128801	GEH	4.0682E-05	SCW	1.17251E-05
LDW	0.000128801	GRI	4.0682E-05	SKP	1.17251E-05
NRH	0.000128801	KAS	4.0682E-05	SYG	1.17251E-05
PYP	0.000128801	KCE	4.0682E-05	VPD	1.17251E-05
QGG	0.000128801	KES	4.0682E-05	VPR	1.17251E-05
QGT	0.000128801	PIS	4.0682E-05	VPT	1.17251E-05
QTV	0.000128801	PWT	4.0682E-05	YCV	1.17251E-05
TCA	0.000128801	SGF	4.0682E-05	YRP	1.17251E-05
AAE	0.000128367	SGH	4.0682E-05	YRQ	1.17251E-05
AVQ	0.000128367	YGW	4.0682E-05	AAQ	1.14757E-05
EPG	0.000128367	AYA	4.0325E-05	AMK	1.14757E-05
FHR	0.000128367	CMN	4.0325E-05	ARC	1.14757E-05
FRM	0.000128367	EHF	4.0325E-05	ATF	1.14757E-05
GFT	0.000128367	FDT	4.0325E-05	CEL	1.14757E-05
HLI	0.000128367	FEL	4.0325E-05	DEY	1.14757E-05
HRM	0.000128367	FPA	4.0325E-05	DHR	1.14757E-05
KQR	0.000128367	FSG	4.0325E-05	DMM	1.14757E-05
PAD	0.000128367	GHA	4.0325E-05	FWD	1.14757E-05
PDG	0.000128367	GIL	4.0325E-05	GGT	1.14757E-05
PMV	0.000128367	IKL	4.0325E-05	GII	1.14757E-05
TCP	0.000128367	KNP	4.0325E-05	GWD	1.14757E-05
TEA	0.000128367	PCS	4.0325E-05	HIR	1.14757E-05
VPH	0.000128367	PML	4.0325E-05	KAR	1.14757E-05
YMR	0.000128367	PNQ	4.0325E-05	KSG	1.14757E-05

YTG	0.000128367	PPE	4.0325E-05	LIF	1.14757E-05
AAK	0.000127934	PSQ	4.0325E-05	LQV	1.14757E-05
AAW	0.000127934	RVI	4.0325E-05	LTA	1.14757E-05
CPA	0.000127934	SVA	4.0325E-05	MNR	1.14757E-05
DGG	0.000127934	TYS	4.0325E-05	NKP	1.14757E-05
PDV	0.000127934	VPR	4.0325E-05	PRQ	1.14757E-05
RGI	0.000127934	WGP	4.0325E-05	PSV	1.14757E-05
RQV	0.000127934	ANP	3.9968E-05	RTI	1.14757E-05
TDG	0.000127934	EGP	3.9968E-05	SGV	1.14757E-05
VGE	0.000127934	FHL	3.9968E-05	SLW	1.14757E-05
VPK	0.000127934	GGW	3.9968E-05	SVG	1.14757E-05
ADA	0.0001275	GLY	3.9968E-05	VMW	1.14757E-05
AMP	0.0001275	IRG	3.9968E-05	VNR	1.14757E-05
APE	0.0001275	KHQ	3.9968E-05	VQT	1.14757E-05
DER	0.0001275	KPT	3.9968E-05	VRA	1.14757E-05
DSW	0.0001275	LKW	3.9968E-05	VWN	1.14757E-05
FRW	0.0001275	MGA	3.9968E-05	WAC	1.14757E-05
IMS	0.0001275	MRN	3.9968E-05	WAN	1.14757E-05
KFL	0.0001275	MWR	3.9968E-05	WMW	1.14757E-05
KYL	0.0001275	NSH	3.9968E-05	WNN	1.14757E-05
NPG	0.0001275	PHG	3.9968E-05	WVC	1.14757E-05
NSK	0.0001275	PKQ	3.9968E-05	YDS	1.14757E-05
REG	0.0001275	QRW	3.9968E-05	YLV	1.14757E-05
TAM	0.0001275	SIK	3.9968E-05	YPH	1.14757E-05
VPY	0.0001275	WVG	3.9968E-05	AGS	1.12262E-05
WGA	0.0001275	YGV	3.9968E-05	ARK	1.12262E-05
YEL	0.0001275	ACS	3.9612E-05	CCR	1.12262E-05
FIL	0.000127066	AFT	3.9612E-05	CRS	1.12262E-05
FSC	0.000127066	CLH	3.9612E-05	DGH	1.12262E-05
GEA	0.000127066	CPY	3.9612E-05	DQG	1.12262E-05
NIS	0.000127066	DEL	3.9612E-05	DSA	1.12262E-05
TVW	0.000127066	FIS	3.9612E-05	EQA	1.12262E-05
VCG	0.000127066	GCE	3.9612E-05	FGC	1.12262E-05
VIA	0.000127066	GRW	3.9612E-05	FVV	1.12262E-05
WAA	0.000127066	HGT	3.9612E-05	GMI	1.12262E-05
YLE	0.000127066	INL	3.9612E-05	GSK	1.12262E-05
FSW	0.000126633	KKS	3.9612E-05	HNA	1.12262E-05
HFS	0.000126633	PTY	3.9612E-05	ILK	1.12262E-05
QAA	0.000126633	QLV	3.9612E-05	KPD	1.12262E-05
QPV	0.000126633	SPH	3.9612E-05	LDG	1.12262E-05
TNG	0.000126633	TWL	3.9612E-05	LIP	1.12262E-05
TNV	0.000126633	WHL	3.9612E-05	LPM	1.12262E-05
VPE	0.000126633	WVQ	3.9612E-05	LTK	1.12262E-05
VQA	0.000126633	ERY	3.9255E-05	LVG	1.12262E-05

VQP	0.000126633	FRF	3.9255E-05	LVQ	1.12262E-05
WLQ	0.000126633	GPH	3.9255E-05	LYE	1.12262E-05
AMV	0.000126199	GPV	3.9255E-05	LYY	1.12262E-05
DIS	0.000126199	HCV	3.9255E-05	MTP	1.12262E-05
KFS	0.000126199	HKL	3.9255E-05	NPS	1.12262E-05
MPA	0.000126199	IPS	3.9255E-05	PDR	1.12262E-05
MTA	0.000126199	KAD	3.9255E-05	PHT	1.12262E-05
NES	0.000126199	KLK	3.9255E-05	PHY	1.12262E-05
PKV	0.000126199	KNL	3.9255E-05	PKY	1.12262E-05
VVD	0.000126199	KTA	3.9255E-05	PLC	1.12262E-05
VVH	0.000126199	LQQ	3.9255E-05	PSI	1.12262E-05
YCS	0.000126199	MRG	3.9255E-05	PVL	1.12262E-05
CVP	0.000125765	NNS	3.9255E-05	PWT	1.12262E-05
GAN	0.000125765	PDQ	3.9255E-05	QDS	1.12262E-05
GIP	0.000125765	PNN	3.9255E-05	QHC	1.12262E-05
GQP	0.000125765	QAS	3.9255E-05	REC	1.12262E-05
GVM	0.000125765	QCD	3.9255E-05	RKQ	1.12262E-05
IVP	0.000125765	TLW	3.9255E-05	RVL	1.12262E-05
KLK	0.000125765	TSN	3.9255E-05	SFW	1.12262E-05
KNT	0.000125765	VRD	3.9255E-05	SIA	1.12262E-05
TMV	0.000125765	AMS	3.8898E-05	SLQ	1.12262E-05
VAH	0.000125765	ASE	3.8898E-05	SWG	1.12262E-05
VDG	0.000125765	DKM	3.8898E-05	TAH	1.12262E-05
VTI	0.000125765	DRS	3.8898E-05	TPP	1.12262E-05
VTN	0.000125765	ELW	3.8898E-05	WDF	1.12262E-05
WVV	0.000125765	FRH	3.8898E-05	WIH	1.12262E-05
YKL	0.000125765	GVG	3.8898E-05	WNQ	1.12262E-05
ATN	0.000125331	IKR	3.8898E-05	WVS	1.12262E-05
DYL	0.000125331	ISR	3.8898E-05	WVV	1.12262E-05
HWL	0.000125331	KPM	3.8898E-05	YEG	1.12262E-05
HYS	0.000125331	MLP	3.8898E-05	YFW	1.12262E-05
KLQ	0.000125331	RFP	3.8898E-05	YNS	1.12262E-05
KMS	0.000125331	SAF	3.8898E-05	ADS	1.09767E-05
KRD	0.000125331	SCA	3.8898E-05	AEM	1.09767E-05
NFR	0.000125331	TGY	3.8898E-05	AHC	1.09767E-05
TAN	0.000125331	TSK	3.8898E-05	APW	1.09767E-05
TGM	0.000125331	VGH	3.8898E-05	AQD	1.09767E-05
TTD	0.000125331	VIT	3.8898E-05	AWS	1.09767E-05
YLQ	0.000125331	VNL	3.8898E-05	EDS	1.09767E-05
YRM	0.000125331	WVS	3.8898E-05	EEY	1.09767E-05
AYA	0.000124898	YAL	3.8898E-05	ENY	1.09767E-05
CAV	0.000124898	YDG	3.8898E-05	ERS	1.09767E-05
ELH	0.000124898	DEP	3.8541E-05	FQA	1.09767E-05
ILW	0.000124898	ESS	3.8541E-05	GFD	1.09767E-05

NQL	0.000124898	GVD	3.8541E-05	GGY	1.09767E-05
PAK	0.000124898	HGL	3.8541E-05	GND	1.09767E-05
YWL	0.000124898	IRK	3.8541E-05	GNW	1.09767E-05
YYS	0.000124898	KFM	3.8541E-05	GTV	1.09767E-05
ANG	0.000124464	KRS	3.8541E-05	HER	1.09767E-05
AVK	0.000124464	LHD	3.8541E-05	HFT	1.09767E-05
DFR	0.000124464	LQR	3.8541E-05	HKH	1.09767E-05
EVP	0.000124464	LWQ	3.8541E-05	HNL	1.09767E-05
GTY	0.000124464	MKT	3.8541E-05	HNS	1.09767E-05
GVH	0.000124464	PTV	3.8541E-05	ISP	1.09767E-05
GWA	0.000124464	RGT	3.8541E-05	KFS	1.09767E-05
GYP	0.000124464	TCS	3.8541E-05	LGY	1.09767E-05
KCR	0.000124464	THG	3.8541E-05	LKA	1.09767E-05
KFR	0.000124464	TYT	3.8541E-05	LPE	1.09767E-05
NSN	0.000124464	WRS	3.8541E-05	MIS	1.09767E-05
PTN	0.000124464	YRG	3.8541E-05	MTC	1.09767E-05
QKL	0.000124464	APV	3.8184E-05	NGN	1.09767E-05
WLW	0.000124464	ARW	3.8184E-05	NSD	1.09767E-05
YER	0.000124464	GCG	3.8184E-05	PIG	1.09767E-05
YKR	0.000124464	GLC	3.8184E-05	PMT	1.09767E-05
GAC	0.00012403	KAP	3.8184E-05	QSA	1.09767E-05
HLN	0.00012403	KCL	3.8184E-05	QYH	1.09767E-05
HSC	0.00012403	KGF	3.8184E-05	RFW	1.09767E-05
KIS	0.00012403	KWQ	3.8184E-05	RGD	1.09767E-05
KSC	0.00012403	LFH	3.8184E-05	RPM	1.09767E-05
LNW	0.00012403	MHA	3.8184E-05	RRW	1.09767E-05
NMT	0.00012403	NND	3.8184E-05	SCS	1.09767E-05
VEA	0.00012403	NNE	3.8184E-05	SDC	1.09767E-05
VTF	0.00012403	PFQ	3.8184E-05	SFC	1.09767E-05
YKS	0.00012403	RVQ	3.8184E-05	SHL	1.09767E-05
AVH	0.000123597	SAP	3.8184E-05	SQH	1.09767E-05
DKR	0.000123597	ADT	3.7827E-05	SQP	1.09767E-05
DWL	0.000123597	CTP	3.7827E-05	THS	1.09767E-05
FYR	0.000123597	EHL	3.7827E-05	TLK	1.09767E-05
GCA	0.000123597	ERG	3.7827E-05	TSC	1.09767E-05
KLD	0.000123597	ETP	3.7827E-05	VGP	1.09767E-05
LYW	0.000123597	FKF	3.7827E-05	VIS	1.09767E-05
VVK	0.000123597	GEW	3.7827E-05	VLP	1.09767E-05
VVN	0.000123597	GHS	3.7827E-05	WGM	1.09767E-05
YYL	0.000123597	GWS	3.7827E-05	WGV	1.09767E-05
CDS	0.000123163	IFW	3.7827E-05	WID	1.09767E-05
DFS	0.000123163	KGA	3.7827E-05	WLY	1.09767E-05
ELC	0.000123163	LIA	3.7827E-05	WSE	1.09767E-05
EVG	0.000123163	LSK	3.7827E-05	WWI	1.09767E-05

GAM	0.000123163	MNF	3.7827E-05	YAQ	1.09767E-05
GKV	0.000123163	MRY	3.7827E-05	YLK	1.09767E-05
GVD	0.000123163	QHA	3.7827E-05	CAQ	1.07273E-05
LCN	0.000123163	QSW	3.7827E-05	DGR	1.07273E-05
LKQ	0.000123163	TVA	3.7827E-05	DWR	1.07273E-05
TPI	0.000123163	WFL	3.7827E-05	EST	1.07273E-05
TVK	0.000123163	YCR	3.7827E-05	EYL	1.07273E-05
VHV	0.000123163	AVP	3.747E-05	FAM	1.07273E-05
VPI	0.000123163	CPL	3.747E-05	FFF	1.07273E-05
YMS	0.000123163	EPT	3.747E-05	FIT	1.07273E-05
AVW	0.000122729	FWG	3.747E-05	FTG	1.07273E-05
CGA	0.000122729	GPI	3.747E-05	IFW	1.07273E-05
FER	0.000122729	ITH	3.747E-05	IRV	1.07273E-05
FLK	0.000122729	IWR	3.747E-05	KKT	1.07273E-05
GCP	0.000122729	KMG	3.747E-05	KRS	1.07273E-05
GTI	0.000122729	MRP	3.747E-05	LAF	1.07273E-05
KRM	0.000122729	NRA	3.747E-05	LDT	1.07273E-05
NRC	0.000122729	PAC	3.747E-05	MCL	1.07273E-05
VAD	0.000122729	PFN	3.747E-05	MVR	1.07273E-05
VAE	0.000122729	PHL	3.747E-05	NAQ	1.07273E-05
VVF	0.000122729	PLN	3.747E-05	NLI	1.07273E-05
AGW	0.000122296	PRN	3.747E-05	PCY	1.07273E-05
APY	0.000122296	PSK	3.747E-05	PKG	1.07273E-05
AWP	0.000122296	QTQ	3.747E-05	PVM	1.07273E-05
DLD	0.000122296	RVV	3.747E-05	QAR	1.07273E-05
DQL	0.000122296	WLC	3.747E-05	QIS	1.07273E-05
DRC	0.000122296	WPL	3.747E-05	QKA	1.07273E-05
DYS	0.000122296	AAR	3.7114E-05	QYC	1.07273E-05
FIS	0.000122296	ATM	3.7114E-05	QYL	1.07273E-05
FWS	0.000122296	DQT	3.7114E-05	RFD	1.07273E-05
KKS	0.000122296	DSP	3.7114E-05	RFR	1.07273E-05
LMM	0.000122296	ENR	3.7114E-05	RNI	1.07273E-05
LWM	0.000122296	GLD	3.7114E-05	RPI	1.07273E-05
NER	0.000122296	GTG	3.7114E-05	RYF	1.07273E-05
VKG	0.000122296	HLL	3.7114E-05	SDA	1.07273E-05
VWA	0.000122296	IAL	3.7114E-05	SGK	1.07273E-05
WVG	0.000122296	ISK	3.7114E-05	SIL	1.07273E-05
YFS	0.000122296	KAE	3.7114E-05	SIT	1.07273E-05
AET	0.000121862	KAH	3.7114E-05	SLN	1.07273E-05
AKV	0.000121862	NLP	3.7114E-05	VAS	1.07273E-05
DCR	0.000121862	PAN	3.7114E-05	VAW	1.07273E-05
DLE	0.000121862	PVF	3.7114E-05	VKP	1.07273E-05
DLH	0.000121862	RLE	3.7114E-05	VLA	1.07273E-05
ENS	0.000121862	RPD	3.7114E-05	WGC	1.07273E-05

FFR	0.000121862	STK	3.7114E-05	WQY	1.07273E-05
GKP	0.000121862	TNL	3.7114E-05	WWM	1.07273E-05
HFL	0.000121862	TWT	3.7114E-05	AFM	1.04778E-05
HRQ	0.000121862	VPS	3.7114E-05	AGM	1.04778E-05
ITV	0.000121862	WGM	3.7114E-05	ASH	1.04778E-05
NSD	0.000121862	AGV	3.6757E-05	AVI	1.04778E-05
PET	0.000121862	APW	3.6757E-05	CKG	1.04778E-05
PWG	0.000121862	ASN	3.6757E-05	DSF	1.04778E-05
QVP	0.000121862	CGL	3.6757E-05	DVR	1.04778E-05
VEV	0.000121862	EAS	3.6757E-05	EDP	1.04778E-05
WHR	0.000121862	ERD	3.6757E-05	EEW	1.04778E-05
YCR	0.000121862	FQT	3.6757E-05	ELG	1.04778E-05
YIS	0.000121862	FRM	3.6757E-05	EQH	1.04778E-05
AMG	0.000121428	GFW	3.6757E-05	FAC	1.04778E-05
EMR	0.000121428	KGR	3.6757E-05	FGI	1.04778E-05
GMV	0.000121428	KNR	3.6757E-05	FGN	1.04778E-05
GVC	0.000121428	KSY	3.6757E-05	FPY	1.04778E-05
KER	0.000121428	LPM	3.6757E-05	FVW	1.04778E-05
KLE	0.000121428	LVR	3.6757E-05	GAF	1.04778E-05
MAT	0.000121428	MAT	3.6757E-05	GEK	1.04778E-05
NFS	0.000121428	RVN	3.6757E-05	GGD	1.04778E-05
QMS	0.000121428	TGC	3.6757E-05	GQF	1.04778E-05
RGF	0.000121428	TRE	3.6757E-05	HYT	1.04778E-05
TTI	0.000121428	TSE	3.6757E-05	ICS	1.04778E-05
VTC	0.000121428	VLT	3.6757E-05	IMI	1.04778E-05
AAM	0.000120995	WRP	3.6757E-05	IWG	1.04778E-05
EGG	0.000120995	YGR	3.6757E-05	KDS	1.04778E-05
EML	0.000120995	YNL	3.6757E-05	LFP	1.04778E-05
GAE	0.000120995	YYL	3.6757E-05	LFY	1.04778E-05
GHA	0.000120995	AGH	3.64E-05	NFM	1.04778E-05
KRC	0.000120995	CML	3.64E-05	NKN	1.04778E-05
MTP	0.000120995	CWL	3.64E-05	NSA	1.04778E-05
NCR	0.000120995	DDL	3.64E-05	NVK	1.04778E-05
NGG	0.000120995	DRD	3.64E-05	PDA	1.04778E-05
QAV	0.000120995	EAD	3.64E-05	PKP	1.04778E-05
QKR	0.000120995	LGI	3.64E-05	PQG	1.04778E-05
TCV	0.000120995	LYF	3.64E-05	PSQ	1.04778E-05
YFL	0.000120995	NPC	3.64E-05	PTG	1.04778E-05
AHG	0.000120561	NPH	3.64E-05	QFR	1.04778E-05
ATY	0.000120561	PSE	3.64E-05	QFT	1.04778E-05
EAV	0.000120561	QNG	3.64E-05	QPH	1.04778E-05
EFR	0.000120561	QQR	3.64E-05	QPN	1.04778E-05
ELW	0.000120561	QSD	3.64E-05	RQP	1.04778E-05
FRC	0.000120561	RRY	3.64E-05	RRV	1.04778E-05

FTG	0.000120561	RSM	3.64E-05	SGS	1.04778E-05
GGH	0.000120561	TPH	3.64E-05	SHP	1.04778E-05
GMG	0.000120561	VDL	3.64E-05	SVM	1.04778E-05
GPQ	0.000120561	WYS	3.64E-05	SYC	1.04778E-05
KWL	0.000120561	DRR	3.6043E-05	SYR	1.04778E-05
LCC	0.000120561	LCH	3.6043E-05	TRM	1.04778E-05
LCM	0.000120561	LWG	3.6043E-05	VPM	1.04778E-05
MAP	0.000120561	MKL	3.6043E-05	VRG	1.04778E-05
MPG	0.000120561	PCR	3.6043E-05	VWK	1.04778E-05
QAG	0.000120561	PSF	3.6043E-05	WTN	1.04778E-05
QSC	0.000120561	QKS	3.6043E-05	YTI	1.04778E-05
TWP	0.000120561	QPG	3.6043E-05	YTW	1.04778E-05
VFG	0.000120561	RHT	3.6043E-05	AFS	1.02283E-05
VMG	0.000120561	RYI	3.6043E-05	AFT	1.02283E-05
AWA	0.000120127	SPE	3.6043E-05	AMS	1.02283E-05
CGP	0.000120127	TQD	3.6043E-05	CLQ	1.02283E-05
FCS	0.000120127	TSG	3.6043E-05	DAD	1.02283E-05
FLD	0.000120127	WPS	3.6043E-05	DAR	1.02283E-05
GVW	0.000120127	YQA	3.6043E-05	DET	1.02283E-05
IGA	0.000120127	GDA	3.5686E-05	DSQ	1.02283E-05
LFM	0.000120127	GVE	3.5686E-05	EAS	1.02283E-05
NLF	0.000120127	GWF	3.5686E-05	EHP	1.02283E-05
NSQ	0.000120127	LGM	3.5686E-05	ELA	1.02283E-05
PAE	0.000120127	LPK	3.5686E-05	ESL	1.02283E-05
QTG	0.000120127	LTN	3.5686E-05	FFP	1.02283E-05
SGI	0.000120127	MGP	3.5686E-05	FFQ	1.02283E-05
TVC	0.000120127	PMR	3.5686E-05	FGE	1.02283E-05
VAN	0.000120127	RRC	3.5686E-05	GAA	1.02283E-05
VVQ	0.000120127	SFG	3.5686E-05	GHC	1.02283E-05
AFA	0.000119694	SIV	3.5686E-05	HNC	1.02283E-05
CAP	0.000119694	VAS	3.5686E-05	HPG	1.02283E-05
ELM	0.000119694	VQS	3.5686E-05	HYW	1.02283E-05
FLI	0.000119694	YSW	3.5686E-05	IIW	1.02283E-05
FYS	0.000119694	AWQ	3.5329E-05	IKP	1.02283E-05
GCV	0.000119694	EVL	3.5329E-05	INH	1.02283E-05
KSN	0.000119694	FKI	3.5329E-05	IRA	1.02283E-05
LKM	0.000119694	FYS	3.5329E-05	IRW	1.02283E-05
NNT	0.000119694	FYT	3.5329E-05	KKP	1.02283E-05
NWR	0.000119694	GHF	3.5329E-05	LHV	1.02283E-05
VHG	0.000119694	IAY	3.5329E-05	LQC	1.02283E-05
VVI	0.000119694	IPL	3.5329E-05	MMW	1.02283E-05
YGP	0.000119694	NAD	3.5329E-05	MRT	1.02283E-05
YYR	0.000119694	NLI	3.5329E-05	MTL	1.02283E-05
FNR	0.00011926	QPA	3.5329E-05	NDQ	1.02283E-05

GGQ	0.00011926	RAK	3.5329E-05	NEK	1.02283E-05
GGW	0.00011926	RCR	3.5329E-05	NMM	1.02283E-05
HQR	0.00011926	SPN	3.5329E-05	NRG	1.02283E-05
HRD	0.00011926	SWP	3.5329E-05	NYS	1.02283E-05
INL	0.00011926	TIT	3.5329E-05	PQL	1.02283E-05
NML	0.00011926	TRW	3.5329E-05	PQS	1.02283E-05
NQR	0.00011926	YRR	3.5329E-05	PYY	1.02283E-05
NRK	0.00011926	AGK	3.4972E-05	QTD	1.02283E-05
NRQ	0.00011926	AHD	3.4972E-05	RAC	1.02283E-05
PHV	0.00011926	ARF	3.4972E-05	RDQ	1.02283E-05
PVN	0.00011926	EDM	3.4972E-05	REL	1.02283E-05
QDL	0.00011926	FKS	3.4972E-05	RLF	1.02283E-05
TTW	0.00011926	GAC	3.4972E-05	SDL	1.02283E-05
TVH	0.00011926	GYT	3.4972E-05	SFR	1.02283E-05
VGK	0.00011926	HSC	3.4972E-05	SMG	1.02283E-05
VTD	0.00011926	KKY	3.4972E-05	SSQ	1.02283E-05
CVG	0.000118826	KSE	3.4972E-05	SVQ	1.02283E-05
FHL	0.000118826	MRQ	3.4972E-05	VHC	1.02283E-05
FRK	0.000118826	NTV	3.4972E-05	VTP	1.02283E-05
GPI	0.000118826	NWL	3.4972E-05	WKN	1.02283E-05
MVA	0.000118826	PGC	3.4972E-05	WMT	1.02283E-05
NLH	0.000118826	PLY	3.4972E-05	WWC	1.02283E-05
VNG	0.000118826	PND	3.4972E-05	YKS	1.02283E-05
WAP	0.000118826	RET	3.4972E-05	YNA	1.02283E-05
YQR	0.000118826	RQP	3.4972E-05	YPA	1.02283E-05
ACV	0.000118393	SAG	3.4972E-05	YTQ	1.02283E-05
AGC	0.000118393	SFA	3.4972E-05	YYT	1.02283E-05
AQA	0.000118393	TIA	3.4972E-05	AGR	9.97885E-06
GPC	0.000118393	VKR	3.4972E-05	AHG	9.97885E-06
GPE	0.000118393	VLK	3.4972E-05	AND	9.97885E-06
HQS	0.000118393	WVA	3.4972E-05	CLA	9.97885E-06
HSE	0.000118393	APQ	3.4616E-05	CSN	9.97885E-06
HSM	0.000118393	AQS	3.4616E-05	CSV	9.97885E-06
KCL	0.000118393	FEP	3.4616E-05	DCL	9.97885E-06
KRW	0.000118393	GCS	3.4616E-05	DNL	9.97885E-06
QDS	0.000118393	GIE	3.4616E-05	DQM	9.97885E-06
WKR	0.000118393	HGQ	3.4616E-05	DYQ	9.97885E-06
YRW	0.000118393	HSL	3.4616E-05	EEE	9.97885E-06
AHV	0.000117959	KDQ	3.4616E-05	ETF	9.97885E-06
DWR	0.000117959	KHA	3.4616E-05	FAP	9.97885E-06
EKL	0.000117959	KSF	3.4616E-05	FDG	9.97885E-06
FKS	0.000117959	LCD	3.4616E-05	FMQ	9.97885E-06
FLH	0.000117959	MLS	3.4616E-05	FNV	9.97885E-06
FQL	0.000117959	NGS	3.4616E-05	GGF	9.97885E-06

HES	0.000117959	NMR	3.4616E-05	GMH	9.97885E-06
KSH	0.000117959	PLW	3.4616E-05	GPI	9.97885E-06
KSW	0.000117959	PPM	3.4616E-05	HPC	9.97885E-06
LNK	0.000117959	RMK	3.4616E-05	HSN	9.97885E-06
NIR	0.000117959	SDH	3.4616E-05	HWT	9.97885E-06
NQS	0.000117959	TAQ	3.4616E-05	IKG	9.97885E-06
QVV	0.000117959	TLH	3.4616E-05	KDG	9.97885E-06
TPF	0.000117959	VAL	3.4616E-05	KES	9.97885E-06
YGV	0.000117959	VSH	3.4616E-05	KEW	9.97885E-06
EGV	0.000117525	WHS	3.4616E-05	KFR	9.97885E-06
EKS	0.000117525	YQR	3.4616E-05	KLV	9.97885E-06
FSN	0.000117525	AAT	3.4259E-05	KQA	9.97885E-06
GGM	0.000117525	AFL	3.4259E-05	KRA	9.97885E-06
HRN	0.000117525	AKT	3.4259E-05	KYR	9.97885E-06
KLH	0.000117525	ARY	3.4259E-05	LHD	9.97885E-06
NLD	0.000117525	ERP	3.4259E-05	LIT	9.97885E-06
PGC	0.000117525	ERV	3.4259E-05	NDP	9.97885E-06
PIG	0.000117525	FFT	3.4259E-05	NLV	9.97885E-06
PNG	0.000117525	FGQ	3.4259E-05	NWA	9.97885E-06
VAF	0.000117525	GLN	3.4259E-05	PAR	9.97885E-06
WPG	0.000117525	GMF	3.4259E-05	PEL	9.97885E-06
WTG	0.000117525	GVQ	3.4259E-05	PGN	9.97885E-06
YIR	0.000117525	KAL	3.4259E-05	PGW	9.97885E-06
APF	0.000117092	KSQ	3.4259E-05	PKQ	9.97885E-06
API	0.000117092	LDW	3.4259E-05	PVI	9.97885E-06
AWG	0.000117092	NER	3.4259E-05	QYT	9.97885E-06
DQR	0.000117092	NFH	3.4259E-05	RNW	9.97885E-06
IDS	0.000117092	NSF	3.4259E-05	SGD	9.97885E-06
LWW	0.000117092	NVL	3.4259E-05	SKW	9.97885E-06
NRN	0.000117092	QIP	3.4259E-05	SMC	9.97885E-06
PQG	0.000117092	QNH	3.4259E-05	SQM	9.97885E-06
TGC	0.000117092	QSQ	3.4259E-05	SVS	9.97885E-06
TGW	0.000117092	RNF	3.4259E-05	TAL	9.97885E-06
TKV	0.000117092	RTG	3.4259E-05	THA	9.97885E-06
TPN	0.000117092	TIS	3.4259E-05	TVT	9.97885E-06
AQG	0.000116658	TLI	3.4259E-05	TYA	9.97885E-06
DNT	0.000116658	WNM	3.4259E-05	VSD	9.97885E-06
EIL	0.000116658	YDI	3.4259E-05	VTK	9.97885E-06
ENL	0.000116658	AKH	3.3902E-05	WYV	9.97885E-06
HSK	0.000116658	ARM	3.3902E-05	YNP	9.97885E-06
IHS	0.000116658	DFL	3.3902E-05	YNW	9.97885E-06
IKS	0.000116658	DKL	3.3902E-05	YWH	9.97885E-06
KLN	0.000116658	EPG	3.3902E-05	YYD	9.97885E-06
LDC	0.000116658	GDF	3.3902E-05	AAC	9.72938E-06

LHM	0.000116658	KGL	3.3902E-05	AHF	9.72938E-06
NNA	0.000116658	KNH	3.3902E-05	AKF	9.72938E-06
PAM	0.000116658	LRI	3.3902E-05	AQV	9.72938E-06
PTY	0.000116658	NCV	3.3902E-05	AVQ	9.72938E-06
TAE	0.000116658	NDH	3.3902E-05	AWP	9.72938E-06
TAK	0.000116658	NPS	3.3902E-05	CIG	9.72938E-06
WNS	0.000116658	PHT	3.3902E-05	CLW	9.72938E-06
YWS	0.000116658	QNQ	3.3902E-05	CNF	9.72938E-06
APK	0.000116224	RTW	3.3902E-05	DKL	9.72938E-06
AVC	0.000116224	RWG	3.3902E-05	DSD	9.72938E-06
CLM	0.000116224	SEQ	3.3902E-05	ESH	9.72938E-06
EKR	0.000116224	SFF	3.3902E-05	FAT	9.72938E-06
EMS	0.000116224	TNS	3.3902E-05	FKA	9.72938E-06
EYR	0.000116224	WRQ	3.3902E-05	FPA	9.72938E-06
PVM	0.000116224	AFS	3.3545E-05	GEW	9.72938E-06
QYR	0.000116224	DMK	3.3545E-05	GLW	9.72938E-06
WLH	0.000116224	ECC	3.3545E-05	GMG	9.72938E-06
EYL	0.000115791	FIT	3.3545E-05	GPR	9.72938E-06
EYS	0.000115791	FWQ	3.3545E-05	HLM	9.72938E-06
FLN	0.000115791	GNY	3.3545E-05	IYP	9.72938E-06
GGE	0.000115791	HTC	3.3545E-05	KLN	9.72938E-06
GHV	0.000115791	HTH	3.3545E-05	KMA	9.72938E-06
GVN	0.000115791	ILA	3.3545E-05	KNP	9.72938E-06
HRW	0.000115791	LCQ	3.3545E-05	KRH	9.72938E-06
WPV	0.000115791	LDS	3.3545E-05	KTH	9.72938E-06
ACG	0.000115357	LMT	3.3545E-05	LFM	9.72938E-06
DLI	0.000115357	LWM	3.3545E-05	LHE	9.72938E-06
EHS	0.000115357	MSY	3.3545E-05	LMV	9.72938E-06
EIS	0.000115357	NAW	3.3545E-05	LVW	9.72938E-06
GAW	0.000115357	NRQ	3.3545E-05	MKS	9.72938E-06
GGY	0.000115357	PAV	3.3545E-05	MWP	9.72938E-06
HLD	0.000115357	PHM	3.3545E-05	MWS	9.72938E-06
HQL	0.000115357	PHP	3.3545E-05	NGD	9.72938E-06
KES	0.000115357	PLC	3.3545E-05	PWS	9.72938E-06
KLI	0.000115357	PNG	3.3545E-05	QAH	9.72938E-06
TPY	0.000115357	PSY	3.3545E-05	QDL	9.72938E-06
TTF	0.000115357	RKY	3.3545E-05	QQP	9.72938E-06
WML	0.000115357	VMN	3.3545E-05	QRS	9.72938E-06
WTV	0.000115357	YDT	3.3545E-05	RCQ	9.72938E-06
AGQ	0.000114923	APN	3.3188E-05	SEW	9.72938E-06
DSN	0.000114923	AWT	3.3188E-05	SKA	9.72938E-06
EHL	0.000114923	DPS	3.3188E-05	SLY	9.72938E-06
ELQ	0.000114923	DRH	3.3188E-05	TPC	9.72938E-06
GAF	0.000114923	EDW	3.3188E-05	TRT	9.72938E-06

GHG	0.000114923	ESN	3.3188E-05	VMC	9.72938E-06
GPK	0.000114923	FDL	3.3188E-05	VRD	9.72938E-06
HWR	0.000114923	GED	3.3188E-05	WCV	9.72938E-06
IWR	0.000114923	HTG	3.3188E-05	YNT	9.72938E-06
KRH	0.000114923	LNN	3.3188E-05	YVL	9.72938E-06
QVG	0.000114923	MRH	3.3188E-05	AAR	9.47991E-06
TEP	0.000114923	NNP	3.3188E-05	AGH	9.47991E-06
WKL	0.000114923	NSD	3.3188E-05	AIM	9.47991E-06
YLH	0.000114923	PWF	3.3188E-05	ARN	9.47991E-06
AFP	0.00011449	RAY	3.3188E-05	CPG	9.47991E-06
CLQ	0.00011449	RHR	3.3188E-05	CRW	9.47991E-06
CPV	0.00011449	SDN	3.3188E-05	CYC	9.47991E-06
GAK	0.00011449	SHL	3.3188E-05	DAC	9.47991E-06
IAV	0.00011449	TLK	3.3188E-05	DDR	9.47991E-06
IDL	0.00011449	VFA	3.3188E-05	DNF	9.47991E-06
KCS	0.00011449	VHT	3.3188E-05	DNS	9.47991E-06
LMH	0.00011449	YIS	3.3188E-05	DPM	9.47991E-06
LWQ	0.00011449	YYA	3.3188E-05	ESG	9.47991E-06
PGE	0.00011449	CSV	3.2831E-05	ETL	9.47991E-06
QDR	0.00011449	CVA	3.2831E-05	FTK	9.47991E-06
WAV	0.00011449	DNG	3.2831E-05	GHQ	9.47991E-06
ATF	0.000114056	EKY	3.2831E-05	GIR	9.47991E-06
CPG	0.000114056	FAA	3.2831E-05	GTG	9.47991E-06
DSK	0.000114056	FQS	3.2831E-05	GTK	9.47991E-06
EPV	0.000114056	FSI	3.2831E-05	GVN	9.47991E-06
EQS	0.000114056	GFD	3.2831E-05	HDH	9.47991E-06
ESC	0.000114056	GNV	3.2831E-05	IFT	9.47991E-06
FQR	0.000114056	HPT	3.2831E-05	IMA	9.47991E-06
GVI	0.000114056	INP	3.2831E-05	INC	9.47991E-06
IWL	0.000114056	KKL	3.2831E-05	IPT	9.47991E-06
SMM	0.000114056	LAI	3.2831E-05	KFG	9.47991E-06
THV	0.000114056	LDR	3.2831E-05	KSL	9.47991E-06
YLD	0.000114056	PAE	3.2831E-05	LAT	9.47991E-06
AVF	0.000113622	QGR	3.2831E-05	LDH	9.47991E-06
AVI	0.000113622	QQS	3.2831E-05	LMQ	9.47991E-06
DRW	0.000113622	QTH	3.2831E-05	NFV	9.47991E-06
GAH	0.000113622	QWL	3.2831E-05	NTN	9.47991E-06
GAQ	0.000113622	RIR	3.2831E-05	NWQ	9.47991E-06
KLY	0.000113622	SYF	3.2831E-05	PKD	9.47991E-06
PNV	0.000113622	TGF	3.2831E-05	PRE	9.47991E-06
PVE	0.000113622	TSF	3.2831E-05	PSM	9.47991E-06
QHL	0.000113622	TSI	3.2831E-05	QRC	9.47991E-06
QRC	0.000113622	VCL	3.2831E-05	QRT	9.47991E-06
QSQ	0.000113622	VSF	3.2831E-05	RDC	9.47991E-06

RNC	0.000113622	VWA	3.2831E-05	RMN	9.47991E-06
TAH	0.000113622	WNP	3.2831E-05	SPE	9.47991E-06
TTE	0.000113622	YKS	3.2831E-05	SSK	9.47991E-06
VFV	0.000113622	YWR	3.2831E-05	SYS	9.47991E-06
VGD	0.000113622	AIA	3.2474E-05	THD	9.47991E-06
VGN	0.000113622	CVD	3.2474E-05	TLD	9.47991E-06
AGH	0.000113189	FNA	3.2474E-05	TMG	9.47991E-06
AKG	0.000113189	HAH	3.2474E-05	TMM	9.47991E-06
DSE	0.000113189	IRS	3.2474E-05	VAH	9.47991E-06
EES	0.000113189	KGG	3.2474E-05	VCF	9.47991E-06
KKT	0.000113189	LSE	3.2474E-05	WDA	9.47991E-06
LYM	0.000113189	LTE	3.2474E-05	WDP	9.47991E-06
MMR	0.000113189	NAM	3.2474E-05	WQT	9.47991E-06
PGM	0.000113189	NFP	3.2474E-05	WWW	9.47991E-06
PVQ	0.000113189	NPA	3.2474E-05	YAG	9.47991E-06
RNW	0.000113189	PFK	3.2474E-05	YCP	9.47991E-06
TGN	0.000113189	PVG	3.2474E-05	YEY	9.47991E-06
VQV	0.000113189	RFK	3.2474E-05	YHP	9.47991E-06
YQL	0.000113189	RLC	3.2474E-05	YTK	9.47991E-06
AVD	0.000112755	TLE	3.2474E-05	ACT	9.23044E-06
CLW	0.000112755	WDR	3.2474E-05	AQK	9.23044E-06
DRN	0.000112755	WPP	3.2474E-05	AYT	9.23044E-06
DSQ	0.000112755	YPT	3.2474E-05	CAL	9.23044E-06
HSN	0.000112755	ADS	3.2118E-05	CMR	9.23044E-06
KRQ	0.000112755	AKA	3.2118E-05	CSY	9.23044E-06
LDM	0.000112755	AKS	3.2118E-05	CYR	9.23044E-06
NRD	0.000112755	DTA	3.2118E-05	DDL	9.23044E-06
PCG	0.000112755	GEQ	3.2118E-05	DLK	9.23044E-06
PVD	0.000112755	GFR	3.2118E-05	DLV	9.23044E-06
QIR	0.000112755	GIR	3.2118E-05	EDG	9.23044E-06
QRD	0.000112755	GMG	3.2118E-05	EEA	9.23044E-06
WKS	0.000112755	HFT	3.2118E-05	EPH	9.23044E-06
YFR	0.000112755	HLR	3.2118E-05	EPT	9.23044E-06
YHG	0.000112755	HRS	3.2118E-05	FFM	9.23044E-06
YRQ	0.000112755	IPT	3.2118E-05	FTI	9.23044E-06
DRK	0.000112321	KYL	3.2118E-05	GAR	9.23044E-06
GVF	0.000112321	LIV	3.2118E-05	HEL	9.23044E-06
KYR	0.000112321	MSG	3.2118E-05	HLL	9.23044E-06
LMN	0.000112321	NAP	3.2118E-05	HTC	9.23044E-06
NIT	0.000112321	PGF	3.2118E-05	IPS	9.23044E-06
SKM	0.000112321	PVV	3.2118E-05	ITN	9.23044E-06
TAD	0.000112321	RFA	3.2118E-05	IWH	9.23044E-06
TGQ	0.000112321	RPW	3.2118E-05	KMC	9.23044E-06
WDS	0.000112321	SPC	3.2118E-05	KSY	9.23044E-06

YLN	0.000112321	YPH	3.2118E-05	LGI	9.23044E-06
CDL	0.000111888	YRV	3.2118E-05	LLK	9.23044E-06
DQS	0.000111888	ALC	3.1761E-05	LMK	9.23044E-06
EQL	0.000111888	ASG	3.1761E-05	LNQ	9.23044E-06
FLF	0.000111888	ASQ	3.1761E-05	LYM	9.23044E-06
GHP	0.000111888	ATN	3.1761E-05	NHH	9.23044E-06
HER	0.000111888	EGR	3.1761E-05	NHS	9.23044E-06
LHW	0.000111888	ESF	3.1761E-05	NSN	9.23044E-06
NSE	0.000111888	FCQ	3.1761E-05	NVS	9.23044E-06
PTI	0.000111888	FPP	3.1761E-05	PMN	9.23044E-06
QHR	0.000111888	FTY	3.1761E-05	QQS	9.23044E-06
QLE	0.000111888	GSW	3.1761E-05	QRN	9.23044E-06
TVI	0.000111888	HRF	3.1761E-05	RAL	9.23044E-06
TWA	0.000111888	ILT	3.1761E-05	RGA	9.23044E-06
VGH	0.000111888	KFC	3.1761E-05	SAD	9.23044E-06
WFL	0.000111888	MNY	3.1761E-05	SAW	9.23044E-06
WNR	0.000111888	NSM	3.1761E-05	SNL	9.23044E-06
YQS	0.000111888	PCE	3.1761E-05	SPQ	9.23044E-06
YRC	0.000111888	PKH	3.1761E-05	SQT	9.23044E-06
ADV	0.000111454	PYR	3.1761E-05	SSE	9.23044E-06
AQV	0.000111454	RAC	3.1761E-05	TLN	9.23044E-06
DSD	0.000111454	RCL	3.1761E-05	TLP	9.23044E-06
DSH	0.000111454	RDV	3.1761E-05	VAF	9.23044E-06
HHT	0.000111454	RGW	3.1761E-05	VHP	9.23044E-06
HWS	0.000111454	SRC	3.1761E-05	VPW	9.23044E-06
KEL	0.000111454	YGA	3.1761E-05	VQP	9.23044E-06
NLI	0.000111454	AGG	3.1404E-05	WPT	9.23044E-06
PGD	0.000111454	AWR	3.1404E-05	YAD	9.23044E-06
TGK	0.000111454	ELH	3.1404E-05	YML	9.23044E-06
VAI	0.000111454	GVV	3.1404E-05	YNV	9.23044E-06
WIR	0.000111454	IPD	3.1404E-05	YRH	9.23044E-06
WWL	0.000111454	ISC	3.1404E-05	AVR	8.98096E-06
CAA	0.00011102	KIT	3.1404E-05	CFL	8.98096E-06
DCT	0.00011102	KVM	3.1404E-05	CST	8.98096E-06
EDR	0.00011102	LIH	3.1404E-05	CVL	8.98096E-06
HRH	0.00011102	MFA	3.1404E-05	DEN	8.98096E-06
IPV	0.00011102	MLM	3.1404E-05	DQL	8.98096E-06
IVG	0.00011102	NLW	3.1404E-05	DRL	8.98096E-06
KRE	0.00011102	NVR	3.1404E-05	DTH	8.98096E-06
KRN	0.00011102	PRY	3.1404E-05	DTS	8.98096E-06
LNQ	0.00011102	QFT	3.1404E-05	ENA	8.98096E-06
PGH	0.00011102	QNA	3.1404E-05	ERD	8.98096E-06
SNC	0.00011102	QSY	3.1404E-05	FAY	8.98096E-06
AAN	0.000110587	RAV	3.1404E-05	FLE	8.98096E-06

AGK	0.000110587	RHH	3.1404E-05	GDV	8.98096E-06
AGN	0.000110587	TAI	3.1404E-05	GFV	8.98096E-06
CNL	0.000110587	TEE	3.1404E-05	GHI	8.98096E-06
FRH	0.000110587	THT	3.1404E-05	GSW	8.98096E-06
GPN	0.000110587	VPH	3.1404E-05	HCS	8.98096E-06
HIP	0.000110587	WTF	3.1404E-05	HGL	8.98096E-06
HSI	0.000110587	YTA	3.1404E-05	HLP	8.98096E-06
ILC	0.000110587	ALD	3.1047E-05	HPL	8.98096E-06
IML	0.000110587	APC	3.1047E-05	IYL	8.98096E-06
LKW	0.000110587	AYP	3.1047E-05	IYS	8.98096E-06
TGE	0.000110587	AYT	3.1047E-05	KEC	8.98096E-06
VYV	0.000110587	DCA	3.1047E-05	KTD	8.98096E-06
WIL	0.000110587	DMA	3.1047E-05	LFH	8.98096E-06
EEL	0.000110153	DTY	3.1047E-05	MGR	8.98096E-06
EFS	0.000110153	FHS	3.1047E-05	PQM	8.98096E-06
EIR	0.000110153	FNQ	3.1047E-05	PYD	8.98096E-06
GDV	0.000110153	FSW	3.1047E-05	QQT	8.98096E-06
GPY	0.000110153	GWA	3.1047E-05	QTP	8.98096E-06
GVK	0.000110153	HFR	3.1047E-05	RIA	8.98096E-06
GYV	0.000110153	HLE	3.1047E-05	RLE	8.98096E-06
LYQ	0.000110153	ISN	3.1047E-05	RML	8.98096E-06
MVG	0.000110153	LCV	3.1047E-05	RQL	8.98096E-06
NSH	0.000110153	LKD	3.1047E-05	RSV	8.98096E-06
PVI	0.000110153	LVT	3.1047E-05	RWF	8.98096E-06
QIL	0.000110153	NRR	3.1047E-05	SAV	8.98096E-06
QLQ	0.000110153	PQT	3.1047E-05	SFY	8.98096E-06
QQR	0.000110153	PVH	3.1047E-05	SIS	8.98096E-06
QRM	0.000110153	PWR	3.1047E-05	SRF	8.98096E-06
TGF	0.000110153	QCA	3.1047E-05	TCW	8.98096E-06
TVQ	0.000110153	QRT	3.1047E-05	TIR	8.98096E-06
VPF	0.000110153	RWQ	3.1047E-05	TQH	8.98096E-06
AAD	0.000109719	SGI	3.1047E-05	VCV	8.98096E-06
ECR	0.000109719	SWT	3.1047E-05	VIT	8.98096E-06
EDS	0.000109719	TWA	3.1047E-05	VNH	8.98096E-06
ERC	0.000109719	TYV	3.1047E-05	VTA	8.98096E-06
ERE	0.000109719	YRP	3.1047E-05	WCW	8.98096E-06
ERM	0.000109719	YSY	3.1047E-05	WFW	8.98096E-06
ERQ	0.000109719	YTT	3.1047E-05	YIG	8.98096E-06
ERW	0.000109719	ASM	3.069E-05	ACL	8.73149E-06
ESM	0.000109719	CVG	3.069E-05	AGP	8.73149E-06
FSE	0.000109719	DWC	3.069E-05	AWH	8.73149E-06
GGD	0.000109719	ENQ	3.069E-05	CRH	8.73149E-06
GIV	0.000109719	FDW	3.069E-05	CSA	8.73149E-06
GTE	0.000109719	GET	3.069E-05	CTF	8.73149E-06

KNA	0.000109719	GND	3.069E-05	CWG	8.73149E-06
KQS	0.000109719	HPQ	3.069E-05	CYS	8.73149E-06
MTV	0.000109719	IGS	3.069E-05	DAY	8.73149E-06
RMC	0.000109719	KCS	3.069E-05	DCC	8.73149E-06
TAF	0.000109719	KNW	3.069E-05	DLQ	8.73149E-06
YSQ	0.000109719	KYA	3.069E-05	DPR	8.73149E-06
AGE	0.000109286	LAQ	3.069E-05	DSM	8.73149E-06
CLC	0.000109286	NIA	3.069E-05	EEK	8.73149E-06
CLN	0.000109286	NLH	3.069E-05	EKL	8.73149E-06
CNS	0.000109286	NRG	3.069E-05	EKP	8.73149E-06
DLY	0.000109286	NTQ	3.069E-05	EKS	8.73149E-06
DSI	0.000109286	PFM	3.069E-05	FAH	8.73149E-06
ESQ	0.000109286	QNP	3.069E-05	FFH	8.73149E-06
FIR	0.000109286	QRE	3.069E-05	FKY	8.73149E-06
GPD	0.000109286	REQ	3.069E-05	FPE	8.73149E-06
HSH	0.000109286	RRK	3.069E-05	GCC	8.73149E-06
HYA	0.000109286	SDE	3.069E-05	GDG	8.73149E-06
IDR	0.000109286	SLC	3.069E-05	GGG	8.73149E-06
IKL	0.000109286	SLH	3.069E-05	GPV	8.73149E-06
KQL	0.000109286	SNN	3.069E-05	HNT	8.73149E-06
LMW	0.000109286	SPG	3.069E-05	HTS	8.73149E-06
NAC	0.000109286	THV	3.069E-05	IGW	8.73149E-06
PTF	0.000109286	VRK	3.069E-05	IKH	8.73149E-06
RDW	0.000109286	WGT	3.069E-05	IKW	8.73149E-06
SDM	0.000109286	WSC	3.069E-05	ISN	8.73149E-06
SNW	0.000109286	APD	3.0333E-05	IVL	8.73149E-06
WHS	0.000109286	CTY	3.0333E-05	KHT	8.73149E-06
WRQ	0.000109286	EKM	3.0333E-05	KIS	8.73149E-06
FQS	0.000108852	FDS	3.0333E-05	KLQ	8.73149E-06
GEP	0.000108852	GPY	3.0333E-05	KRL	8.73149E-06
ILQ	0.000108852	GYM	3.0333E-05	LAP	8.73149E-06
ITG	0.000108852	HKQ	3.0333E-05	LGF	8.73149E-06
LFW	0.000108852	KFW	3.0333E-05	LQM	8.73149E-06
LIC	0.000108852	LFA	3.0333E-05	MHT	8.73149E-06
PAN	0.000108852	LMH	3.0333E-05	NKG	8.73149E-06
QLW	0.000108852	NKP	3.0333E-05	NMH	8.73149E-06
QQL	0.000108852	PEH	3.0333E-05	NMW	8.73149E-06
RKC	0.000108852	PRF	3.0333E-05	NQG	8.73149E-06
CNR	0.000108418	QCV	3.0333E-05	NVL	8.73149E-06
CRM	0.000108418	QFR	3.0333E-05	PCA	8.73149E-06
ECS	0.000108418	QSF	3.0333E-05	PKA	8.73149E-06
ELF	0.000108418	RKV	3.0333E-05	PLW	8.73149E-06
FSH	0.000108418	RWY	3.0333E-05	QDQ	8.73149E-06
GEV	0.000108418	RYC	3.0333E-05	QNY	8.73149E-06

GVE	0.000108418	SLM	3.0333E-05	QYD	8.73149E-06
HRE	0.000108418	SVV	3.0333E-05	RHV	8.73149E-06
IIS	0.000108418	SWI	3.0333E-05	RMY	8.73149E-06
ILK	0.000108418	SYQ	3.0333E-05	RTW	8.73149E-06
NFT	0.000108418	TWS	3.0333E-05	RWD	8.73149E-06
PYG	0.000108418	VML	3.0333E-05	RWM	8.73149E-06
QHS	0.000108418	WGK	3.0333E-05	SAC	8.73149E-06
QLC	0.000108418	YRI	3.0333E-05	SDT	8.73149E-06
QML	0.000108418	AKF	2.9976E-05	SVT	8.73149E-06
RMQ	0.000108418	CFL	2.9976E-05	SWA	8.73149E-06
TVD	0.000108418	CNL	2.9976E-05	VGS	8.73149E-06
TWV	0.000108418	DLC	2.9976E-05	VLY	8.73149E-06
WRW	0.000108418	DRQ	2.9976E-05	VTE	8.73149E-06
WYS	0.000108418	EDR	2.9976E-05	WQN	8.73149E-06
CGT	0.000107985	FKK	2.9976E-05	WVK	8.73149E-06
ELI	0.000107985	FMP	2.9976E-05	YDA	8.73149E-06
FSF	0.000107985	FSD	2.9976E-05	YDE	8.73149E-06
HNT	0.000107985	GPD	2.9976E-05	YQW	8.73149E-06
IIR	0.000107985	HPS	2.9976E-05	YRC	8.73149E-06
ILM	0.000107985	IEP	2.9976E-05	AIT	8.48202E-06
INR	0.000107985	IIS	2.9976E-05	AWT	8.48202E-06
LHH	0.000107985	IPG	2.9976E-05	CFC	8.48202E-06
NTM	0.000107985	KAT	2.9976E-05	CHR	8.48202E-06
NVE	0.000107985	KHL	2.9976E-05	CRC	8.48202E-06
PFV	0.000107985	MAS	2.9976E-05	EED	8.48202E-06
TFV	0.000107985	MSV	2.9976E-05	EPP	8.48202E-06
TKG	0.000107985	NFV	2.9976E-05	ERL	8.48202E-06
WFS	0.000107985	NML	2.9976E-05	ERY	8.48202E-06
YSE	0.000107985	PSI	2.9976E-05	EYC	8.48202E-06
AFV	0.000107551	QKR	2.9976E-05	FNN	8.48202E-06
CTV	0.000107551	QPC	2.9976E-05	FYH	8.48202E-06
DES	0.000107551	QRH	2.9976E-05	GAC	8.48202E-06
EHR	0.000107551	QWF	2.9976E-05	GCP	8.48202E-06
GCG	0.000107551	QYT	2.9976E-05	GGV	8.48202E-06
KTW	0.000107551	RPC	2.9976E-05	GIT	8.48202E-06
NDT	0.000107551	SEF	2.9976E-05	GVS	8.48202E-06
NMP	0.000107551	SNV	2.9976E-05	GWN	8.48202E-06
NRE	0.000107551	VES	2.9976E-05	GYN	8.48202E-06
QRQ	0.000107551	VGQ	2.9976E-05	HPR	8.48202E-06
WGG	0.000107551	WKR	2.9976E-05	HRR	8.48202E-06
YLI	0.000107551	YAT	2.9976E-05	HSA	8.48202E-06
YSN	0.000107551	APY	2.9619E-05	HSP	8.48202E-06
AVE	0.000107117	DLQ	2.9619E-05	IKA	8.48202E-06
ECL	0.000107117	EAT	2.9619E-05	IRN	8.48202E-06

ELE	0.000107117	ELY	2.9619E-05	KKL	8.48202E-06
FRI	0.000107117	ENI	2.9619E-05	KMD	8.48202E-06
GGF	0.000107117	EPY	2.9619E-05	LAN	8.48202E-06
GPF	0.000107117	ETT	2.9619E-05	MGQ	8.48202E-06
IGV	0.000107117	FHA	2.9619E-05	MKC	8.48202E-06
LEW	0.000107117	GEN	2.9619E-05	NAS	8.48202E-06
LMC	0.000107117	GRC	2.9619E-05	NKQ	8.48202E-06
LNH	0.000107117	HRP	2.9619E-05	NLW	8.48202E-06
LYI	0.000107117	HWW	2.9619E-05	NPL	8.48202E-06
MGT	0.000107117	ICL	2.9619E-05	NQQ	8.48202E-06
MNS	0.000107117	ILQ	2.9619E-05	NRT	8.48202E-06
NCT	0.000107117	KCP	2.9619E-05	PAG	8.48202E-06
NWS	0.000107117	KPW	2.9619E-05	PAP	8.48202E-06
PIV	0.000107117	LKE	2.9619E-05	PAT	8.48202E-06
QRW	0.000107117	LVV	2.9619E-05	PCH	8.48202E-06
SWM	0.000107117	NAA	2.9619E-05	PVR	8.48202E-06
TFG	0.000107117	NPY	2.9619E-05	QCD	8.48202E-06
TVY	0.000107117	NVT	2.9619E-05	QWR	8.48202E-06
VGF	0.000107117	PCL	2.9619E-05	QYN	8.48202E-06
VIG	0.000107117	PGQ	2.9619E-05	RIT	8.48202E-06
WDR	0.000107117	PIL	2.9619E-05	RTV	8.48202E-06
WSC	0.000107117	QRL	2.9619E-05	SFA	8.48202E-06
YNT	0.000107117	RCW	2.9619E-05	SFV	8.48202E-06
AGF	0.000106684	RTC	2.9619E-05	SGA	8.48202E-06
AIV	0.000106684	SGN	2.9619E-05	SHM	8.48202E-06
AVN	0.000106684	TYG	2.9619E-05	SWH	8.48202E-06
DIT	0.000106684	VAR	2.9619E-05	SWW	8.48202E-06
DLF	0.000106684	WPT	2.9619E-05	TGQ	8.48202E-06
ERN	0.000106684	YAD	2.9619E-05	TSA	8.48202E-06
FLY	0.000106684	YPP	2.9619E-05	VDF	8.48202E-06
FRD	0.000106684	EAY	2.9263E-05	VNI	8.48202E-06
GQV	0.000106684	EKS	2.9263E-05	VYH	8.48202E-06
IKR	0.000106684	GDH	2.9263E-05	WAE	8.48202E-06
MKR	0.000106684	GKC	2.9263E-05	WAT	8.48202E-06
MVV	0.000106684	GLI	2.9263E-05	WDD	8.48202E-06
SIW	0.000106684	GTM	2.9263E-05	WVA	8.48202E-06
VYG	0.000106684	GWH	2.9263E-05	YES	8.48202E-06
WSQ	0.000106684	HLK	2.9263E-05	YMS	8.48202E-06
CMR	0.00010625	HNR	2.9263E-05	YSI	8.48202E-06
EER	0.00010625	IRE	2.9263E-05	APQ	8.23255E-06
FRN	0.00010625	KER	2.9263E-05	CLC	8.23255E-06
LCK	0.00010625	LAV	2.9263E-05	CNG	8.23255E-06
LDQ	0.00010625	LCY	2.9263E-05	CNS	8.23255E-06
LHE	0.00010625	MKP	2.9263E-05	EPR	8.23255E-06

LNM	0.00010625	MYG	2.9263E-05	EQL	8.23255E-06
MLC	0.00010625	PEC	2.9263E-05	EVQ	8.23255E-06
NDP	0.00010625	PRK	2.9263E-05	EWL	8.23255E-06
NHT	0.00010625	PYL	2.9263E-05	FCD	8.23255E-06
NKT	0.00010625	QCG	2.9263E-05	FDA	8.23255E-06
QNA	0.00010625	RCF	2.9263E-05	FMD	8.23255E-06
ELD	0.000105816	SAN	2.9263E-05	FMP	8.23255E-06
INT	0.000105816	SEC	2.9263E-05	GCG	8.23255E-06
LFC	0.000105816	SKA	2.9263E-05	GFL	8.23255E-06
LKC	0.000105816	SMR	2.9263E-05	GPM	8.23255E-06
MAA	0.000105816	STD	2.9263E-05	HCL	8.23255E-06
PEV	0.000105816	SYR	2.9263E-05	HSD	8.23255E-06
PGN	0.000105816	TAN	2.9263E-05	HYL	8.23255E-06
RKW	0.000105816	TCQ	2.9263E-05	IDS	8.23255E-06
WCR	0.000105816	TGQ	2.9263E-05	IKM	8.23255E-06
WGV	0.000105816	VMT	2.9263E-05	IKN	8.23255E-06
YRN	0.000105816	VPP	2.9263E-05	ILV	8.23255E-06
HRK	0.000105383	VPT	2.9263E-05	KGR	8.23255E-06
IAG	0.000105383	VRE	2.9263E-05	KKY	8.23255E-06
ICS	0.000105383	WNH	2.9263E-05	KLD	8.23255E-06
IFL	0.000105383	YCL	2.9263E-05	KTN	8.23255E-06
IRM	0.000105383	AVL	2.8906E-05	LAM	8.23255E-06
KRF	0.000105383	EFL	2.8906E-05	LMH	8.23255E-06
LDK	0.000105383	EGN	2.8906E-05	LNV	8.23255E-06
LYC	0.000105383	ENY	2.8906E-05	MWN	8.23255E-06
PCV	0.000105383	FIV	2.8906E-05	NAD	8.23255E-06
QLN	0.000105383	GDY	2.8906E-05	NAN	8.23255E-06
TQG	0.000105383	GER	2.8906E-05	NAP	8.23255E-06
TVF	0.000105383	GFG	2.8906E-05	NPT	8.23255E-06
VVY	0.000105383	GQP	2.8906E-05	NQR	8.23255E-06
WQL	0.000105383	GQR	2.8906E-05	NTL	8.23255E-06
AAQ	0.000104949	INR	2.8906E-05	NTP	8.23255E-06
AWV	0.000104949	IWQ	2.8906E-05	PFW	8.23255E-06
CIS	0.000104949	IYT	2.8906E-05	PGH	8.23255E-06
EQR	0.000104949	KGQ	2.8906E-05	PIA	8.23255E-06
GNG	0.000104949	KTH	2.8906E-05	PIQ	8.23255E-06
ILN	0.000104949	KTK	2.8906E-05	PLP	8.23255E-06
LHC	0.000104949	NGV	2.8906E-05	PQP	8.23255E-06
NSF	0.000104949	NNH	2.8906E-05	PWV	8.23255E-06
NVM	0.000104949	PVW	2.8906E-05	PYL	8.23255E-06
PVH	0.000104949	QDI	2.8906E-05	QGH	8.23255E-06
QFR	0.000104949	QNT	2.8906E-05	QMG	8.23255E-06
RMM	0.000104949	QPQ	2.8906E-05	QRG	8.23255E-06
SHM	0.000104949	RDD	2.8906E-05	QTL	8.23255E-06

SNM	0.000104949	REC	2.8906E-05	RCY	8.23255E-06
THG	0.000104949	SFP	2.8906E-05	RHF	8.23255E-06
TVE	0.000104949	TTE	2.8906E-05	RMH	8.23255E-06
VEG	0.000104949	VKQ	2.8906E-05	SEA	8.23255E-06
WAG	0.000104949	VQL	2.8906E-05	SPW	8.23255E-06
WHL	0.000104949	VTY	2.8906E-05	SRY	8.23255E-06
YES	0.000104949	WVP	2.8906E-05	STF	8.23255E-06
YRK	0.000104949	DHL	2.8549E-05	SVH	8.23255E-06
AAF	0.000104515	DVA	2.8549E-05	TLE	8.23255E-06
AYV	0.000104515	EAQ	2.8549E-05	TPR	8.23255E-06
DNA	0.000104515	EGK	2.8549E-05	TRR	8.23255E-06
DRQ	0.000104515	ETY	2.8549E-05	TSN	8.23255E-06
DRY	0.000104515	FMK	2.8549E-05	VAR	8.23255E-06
DSF	0.000104515	HLI	2.8549E-05	VEY	8.23255E-06
ELN	0.000104515	KKH	2.8549E-05	VKW	8.23255E-06
ESW	0.000104515	MCN	2.8549E-05	VNC	8.23255E-06
FES	0.000104515	PFV	2.8549E-05	VQY	8.23255E-06
FSK	0.000104515	PIY	2.8549E-05	VSQ	8.23255E-06
FWP	0.000104515	PLE	2.8549E-05	WIF	8.23255E-06
IHR	0.000104515	PTC	2.8549E-05	YLH	8.23255E-06
ISM	0.000104515	PTF	2.8549E-05	YPV	8.23255E-06
KGV	0.000104515	QLH	2.8549E-05	YQH	8.23255E-06
LMQ	0.000104515	RNV	2.8549E-05	YQT	8.23255E-06
MVP	0.000104515	SCP	2.8549E-05	ACR	7.98308E-06
SDW	0.000104515	SCW	2.8549E-05	ADH	7.98308E-06
SFW	0.000104515	SHR	2.8549E-05	AES	7.98308E-06
SNQ	0.000104515	SPF	2.8549E-05	AMN	7.98308E-06
TIG	0.000104515	STG	2.8549E-05	ANN	7.98308E-06
TVN	0.000104515	SVH	2.8549E-05	AQN	7.98308E-06
TYV	0.000104515	TFM	2.8549E-05	ATI	7.98308E-06
AAI	0.000104082	TVG	2.8549E-05	CFR	7.98308E-06
AGD	0.000104082	VAD	2.8549E-05	CQG	7.98308E-06
ATI	0.000104082	VNH	2.8549E-05	CWT	7.98308E-06
CAG	0.000104082	WGN	2.8549E-05	CWW	7.98308E-06
DVM	0.000104082	WSP	2.8549E-05	DSH	7.98308E-06
ESK	0.000104082	CGP	2.8192E-05	DVK	7.98308E-06
HRY	0.000104082	CLC	2.8192E-05	DYG	7.98308E-06
IIL	0.000104082	DLD	2.8192E-05	EKF	7.98308E-06
ISW	0.000104082	EMT	2.8192E-05	EQQ	7.98308E-06
LDN	0.000104082	ETM	2.8192E-05	ESV	7.98308E-06
LEQ	0.000104082	FGD	2.8192E-05	GCN	7.98308E-06
LKK	0.000104082	FMR	2.8192E-05	GGN	7.98308E-06
MPV	0.000104082	FPD	2.8192E-05	GMA	7.98308E-06
QLK	0.000104082	GSQ	2.8192E-05	GQY	7.98308E-06

QSM	0.000104082	HKA	2.8192E-05	GSQ	7.98308E-06
QSW	0.000104082	HRW	2.8192E-05	GTQ	7.98308E-06
SCC	0.000104082	IKQ	2.8192E-05	ILN	7.98308E-06
SDC	0.000104082	MIP	2.8192E-05	ITM	7.98308E-06
SDN	0.000104082	NWD	2.8192E-05	KAC	7.98308E-06
TYG	0.000104082	QKW	2.8192E-05	KEL	7.98308E-06
WLD	0.000104082	SIL	2.8192E-05	KFP	7.98308E-06
WSE	0.000104082	SMT	2.8192E-05	KPY	7.98308E-06
YLF	0.000104082	SQL	2.8192E-05	LAG	7.98308E-06
AIG	0.000103648	SSY	2.8192E-05	LVI	7.98308E-06
CHL	0.000103648	TCR	2.8192E-05	MMC	7.98308E-06
CHR	0.000103648	TDS	2.8192E-05	MSN	7.98308E-06
CSW	0.000103648	VFL	2.8192E-05	MSR	7.98308E-06
GQG	0.000103648	WKP	2.8192E-05	NCC	7.98308E-06
HSD	0.000103648	YEW	2.8192E-05	NDG	7.98308E-06
ILH	0.000103648	YYR	2.8192E-05	NEC	7.98308E-06
KMT	0.000103648	ALK	2.7835E-05	NKY	7.98308E-06
KNP	0.000103648	APM	2.7835E-05	NQT	7.98308E-06
NVW	0.000103648	EDY	2.7835E-05	NTG	7.98308E-06
NYT	0.000103648	ETH	2.7835E-05	PAW	7.98308E-06
PAF	0.000103648	FER	2.7835E-05	PER	7.98308E-06
PVF	0.000103648	ILP	2.7835E-05	PIV	7.98308E-06
QCR	0.000103648	KRE	2.7835E-05	PLE	7.98308E-06
QEL	0.000103648	LAE	2.7835E-05	PQE	7.98308E-06
QFL	0.000103648	LHE	2.7835E-05	PRK	7.98308E-06
QSH	0.000103648	MCT	2.7835E-05	PWP	7.98308E-06
SMQ	0.000103648	MWL	2.7835E-05	PYH	7.98308E-06
VGI	0.000103648	NGP	2.7835E-05	QAN	7.98308E-06
WMR	0.000103648	NPK	2.7835E-05	QDP	7.98308E-06
WRH	0.000103648	NYS	2.7835E-05	QHQ	7.98308E-06
CFL	0.000103214	PAQ	2.7835E-05	QSP	7.98308E-06
DRI	0.000103214	PCG	2.7835E-05	RAA	7.98308E-06
GGC	0.000103214	QHR	2.7835E-05	RCD	7.98308E-06
GKG	0.000103214	SAC	2.7835E-05	RVD	7.98308E-06
IEL	0.000103214	SCT	2.7835E-05	SHV	7.98308E-06
KSD	0.000103214	SIG	2.7835E-05	SPN	7.98308E-06
LHF	0.000103214	SLD	2.7835E-05	SRE	7.98308E-06
LQC	0.000103214	SMQ	2.7835E-05	SYV	7.98308E-06
QLM	0.000103214	SND	2.7835E-05	TEP	7.98308E-06
QSN	0.000103214	STF	2.7835E-05	TNS	7.98308E-06
RHH	0.000103214	TEP	2.7835E-05	TSP	7.98308E-06
RMN	0.000103214	VWS	2.7835E-05	TVL	7.98308E-06
SDK	0.000103214	WAN	2.7835E-05	VLC	7.98308E-06
SHW	0.000103214	YGM	2.7835E-05	VPN	7.98308E-06

WDL	0.000103214	YVL	2.7835E-05	VYF	7.98308E-06
CGG	0.00010278	ANF	2.7478E-05	WAG	7.98308E-06
CKR	0.00010278	AQR	2.7478E-05	WAW	7.98308E-06
DNP	0.00010278	AVA	2.7478E-05	WMQ	7.98308E-06
ESH	0.00010278	DTT	2.7478E-05	WPF	7.98308E-06
HMT	0.00010278	EKH	2.7478E-05	WWF	7.98308E-06
HPQ	0.00010278	ENN	2.7478E-05	YEC	7.98308E-06
HYP	0.00010278	ENP	2.7478E-05	YPT	7.98308E-06
HYT	0.00010278	EYS	2.7478E-05	YWG	7.98308E-06
IHL	0.00010278	FGW	2.7478E-05	AAF	7.73361E-06
LCD	0.00010278	FIA	2.7478E-05	AMD	7.73361E-06
MGP	0.00010278	FKY	2.7478E-05	CWA	7.73361E-06
MSM	0.00010278	FSN	2.7478E-05	DHQ	7.73361E-06
MWL	0.00010278	GTW	2.7478E-05	DNG	7.73361E-06
PFG	0.00010278	GVT	2.7478E-05	DNT	7.73361E-06
PKG	0.00010278	GYG	2.7478E-05	DQR	7.73361E-06
QCL	0.00010278	HPP	2.7478E-05	DWT	7.73361E-06
QLH	0.00010278	KKR	2.7478E-05	EKC	7.73361E-06
QYS	0.00010278	KRP	2.7478E-05	ERH	7.73361E-06
RNN	0.00010278	MKR	2.7478E-05	ETT	7.73361E-06
TAY	0.00010278	PHW	2.7478E-05	FEM	7.73361E-06
WYR	0.00010278	PQA	2.7478E-05	FNH	7.73361E-06
YSH	0.00010278	QCR	2.7478E-05	FVG	7.73361E-06
CDR	0.000102347	QSE	2.7478E-05	FVM	7.73361E-06
CFS	0.000102347	RGN	2.7478E-05	GCS	7.73361E-06
CTG	0.000102347	RHE	2.7478E-05	GIY	7.73361E-06
GAY	0.000102347	RNC	2.7478E-05	GQN	7.73361E-06
GVY	0.000102347	RRE	2.7478E-05	HGR	7.73361E-06
HKT	0.000102347	RTF	2.7478E-05	HLK	7.73361E-06
KKP	0.000102347	SID	2.7478E-05	HPA	7.73361E-06
LMK	0.000102347	SMH	2.7478E-05	IAQ	7.73361E-06
LNE	0.000102347	THR	2.7478E-05	IFS	7.73361E-06
NLY	0.000102347	TIG	2.7478E-05	IQS	7.73361E-06
RQM	0.000102347	TSW	2.7478E-05	ITC	7.73361E-06
SIM	0.000102347	VAA	2.7478E-05	IWC	7.73361E-06
SIQ	0.000102347	VNM	2.7478E-05	KAY	7.73361E-06
SYW	0.000102347	VNP	2.7478E-05	KLI	7.73361E-06
VAY	0.000102347	VSM	2.7478E-05	KVS	7.73361E-06
VQG	0.000102347	VTA	2.7478E-05	KWD	7.73361E-06
WRE	0.000102347	YMS	2.7478E-05	LAH	7.73361E-06
CFR	0.000101913	YTC	2.7478E-05	LEM	7.73361E-06
EWL	0.000101913	YTI	2.7478E-05	LKM	7.73361E-06
GAI	0.000101913	AIP	2.7121E-05	LMY	7.73361E-06
HIT	0.000101913	CTR	2.7121E-05	LTI	7.73361E-06

LIK	0.000101913	DAT	2.7121E-05	MFR	7.73361E-06
MAV	0.000101913	DWF	2.7121E-05	NDD	7.73361E-06
NKA	0.000101913	FNP	2.7121E-05	NDY	7.73361E-06
NMA	0.000101913	FRK	2.7121E-05	NFA	7.73361E-06
NSI	0.000101913	GRK	2.7121E-05	NFC	7.73361E-06
PYV	0.000101913	IAQ	2.7121E-05	NHT	7.73361E-06
QIS	0.000101913	INI	2.7121E-05	NMN	7.73361E-06
RQH	0.000101913	IPK	2.7121E-05	NMV	7.73361E-06
WLN	0.000101913	KLQ	2.7121E-05	NSK	7.73361E-06
YRE	0.000101913	PRM	2.7121E-05	PAL	7.73361E-06
AGI	0.000101479	QLP	2.7121E-05	PAY	7.73361E-06
CSM	0.000101479	RCV	2.7121E-05	PGP	7.73361E-06
DRD	0.000101479	RTE	2.7121E-05	PHV	7.73361E-06
DVC	0.000101479	RYV	2.7121E-05	PQT	7.73361E-06
EFL	0.000101479	SDV	2.7121E-05	PVQ	7.73361E-06
GGN	0.000101479	SQT	2.7121E-05	QCA	7.73361E-06
HKP	0.000101479	TGI	2.7121E-05	QGN	7.73361E-06
HTM	0.000101479	TVI	2.7121E-05	RFC	7.73361E-06
IGG	0.000101479	VNA	2.7121E-05	RIC	7.73361E-06
ISC	0.000101479	WKT	2.7121E-05	RKD	7.73361E-06
IYR	0.000101479	WLI	2.7121E-05	RQD	7.73361E-06
LDF	0.000101479	YYC	2.7121E-05	RTQ	7.73361E-06
NTQ	0.000101479	AEA	2.6765E-05	SET	7.73361E-06
NTW	0.000101479	APE	2.6765E-05	TAS	7.73361E-06
QCS	0.000101479	AVS	2.6765E-05	TTH	7.73361E-06
QRE	0.000101479	CGH	2.6765E-05	VQM	7.73361E-06
TTY	0.000101479	ELM	2.6765E-05	VRK	7.73361E-06
WFR	0.000101479	FWF	2.6765E-05	VVM	7.73361E-06
WIS	0.000101479	GHP	2.6765E-05	WCC	7.73361E-06
YLY	0.000101479	GPQ	2.6765E-05	WEE	7.73361E-06
CLD	0.000101046	GTC	2.6765E-05	WGF	7.73361E-06
DRH	0.000101046	HCC	2.6765E-05	WHE	7.73361E-06
ERD	0.000101046	IHG	2.6765E-05	WIL	7.73361E-06
FNT	0.000101046	IRD	2.6765E-05	WLK	7.73361E-06
FSI	0.000101046	KEP	2.6765E-05	WPV	7.73361E-06
GFG	0.000101046	KLW	2.6765E-05	WQD	7.73361E-06
HSY	0.000101046	KTD	2.6765E-05	WRK	7.73361E-06
IYS	0.000101046	KTY	2.6765E-05	YTA	7.73361E-06
LFH	0.000101046	NLQ	2.6765E-05	AHI	7.48414E-06
MCL	0.000101046	NTN	2.6765E-05	ALD	7.48414E-06
NDA	0.000101046	PHQ	2.6765E-05	ARW	7.48414E-06
PAI	0.000101046	QPT	2.6765E-05	CRP	7.48414E-06
PGY	0.000101046	QRV	2.6765E-05	CSP	7.48414E-06
PVK	0.000101046	QTC	2.6765E-05	CYT	7.48414E-06

PWV	0.000101046	RQM	2.6765E-05	DTN	7.48414E-06
SKW	0.000101046	SWA	2.6765E-05	DVH	7.48414E-06
SMW	0.000101046	THS	2.6765E-05	DVT	7.48414E-06
WCL	0.000101046	TTD	2.6765E-05	DWQ	7.48414E-06
WEL	0.000101046	TVT	2.6765E-05	ENV	7.48414E-06
WRM	0.000101046	VGG	2.6765E-05	ETP	7.48414E-06
YDT	0.000101046	WSY	2.6765E-05	EYD	7.48414E-06
YRF	0.000101046	WTH	2.6765E-05	FEF	7.48414E-06
AEG	0.000100612	YLN	2.6765E-05	FFA	7.48414E-06
CCS	0.000100612	YPF	2.6765E-05	FVS	7.48414E-06
CLK	0.000100612	YQL	2.6765E-05	GCR	7.48414E-06
CML	0.000100612	AHA	2.6408E-05	GDI	7.48414E-06
DMT	0.000100612	AHQ	2.6408E-05	GID	7.48414E-06
ELK	0.000100612	CPS	2.6408E-05	GLE	7.48414E-06
FSY	0.000100612	DKT	2.6408E-05	GRE	7.48414E-06
GYG	0.000100612	ENV	2.6408E-05	GVT	7.48414E-06
HNP	0.000100612	FNV	2.6408E-05	GVV	7.48414E-06
INS	0.000100612	FRY	2.6408E-05	GWM	7.48414E-06
KSE	0.000100612	GRM	2.6408E-05	GYQ	7.48414E-06
KSQ	0.000100612	IIT	2.6408E-05	HFH	7.48414E-06
LEC	0.000100612	KDP	2.6408E-05	HKL	7.48414E-06
LIH	0.000100612	KID	2.6408E-05	HNH	7.48414E-06
LME	0.000100612	KNG	2.6408E-05	HTD	7.48414E-06
LYK	0.000100612	KRN	2.6408E-05	HTL	7.48414E-06
MGA	0.000100612	KYN	2.6408E-05	IAS	7.48414E-06
MKL	0.000100612	LNI	2.6408E-05	IKI	7.48414E-06
PGK	0.000100612	MFF	2.6408E-05	ITG	7.48414E-06
CIL	0.000100178	MYS	2.6408E-05	KDL	7.48414E-06
CMS	0.000100178	NNM	2.6408E-05	KKA	7.48414E-06
HLF	0.000100178	RNY	2.6408E-05	KPH	7.48414E-06
IRW	0.000100178	RWV	2.6408E-05	LAD	7.48414E-06
KWS	0.000100178	SER	2.6408E-05	LFF	7.48414E-06
LCQ	0.000100178	SHP	2.6408E-05	LKN	7.48414E-06
MHR	0.000100178	SLK	2.6408E-05	LMN	7.48414E-06
MML	0.000100178	TFY	2.6408E-05	MPK	7.48414E-06
QLD	0.000100178	VMM	2.6408E-05	NCT	7.48414E-06
QYL	0.000100178	VMQ	2.6408E-05	NIT	7.48414E-06
RIQ	0.000100178	WAL	2.6408E-05	PFG	7.48414E-06
TGD	0.000100178	WSV	2.6408E-05	PGS	7.48414E-06
TGH	0.000100178	YFH	2.6408E-05	PWG	7.48414E-06
WER	0.000100178	ANV	2.6051E-05	QDH	7.48414E-06
YFT	0.000100178	CGV	2.6051E-05	QIG	7.48414E-06
CYL	9.97448E-05	DTS	2.6051E-05	QPG	7.48414E-06
DTW	9.97448E-05	EFT	2.6051E-05	QSW	7.48414E-06

DYP	9.97448E-05	GGK	2.6051E-05	RED	7.48414E-06
IYL	9.97448E-05	GPC	2.6051E-05	RFH	7.48414E-06
KKA	9.97448E-05	GPG	2.6051E-05	RGF	7.48414E-06
LDE	9.97448E-05	GYH	2.6051E-05	RQG	7.48414E-06
LWC	9.97448E-05	HRL	2.6051E-05	SCT	7.48414E-06
MIR	9.97448E-05	HSN	2.6051E-05	SHW	7.48414E-06
NAW	9.97448E-05	KSN	2.6051E-05	SNY	7.48414E-06
NNP	9.97448E-05	KTM	2.6051E-05	THT	7.48414E-06
NTH	9.97448E-05	LDT	2.6051E-05	TIT	7.48414E-06
QGV	9.97448E-05	LKM	2.6051E-05	TPL	7.48414E-06
RHN	9.97448E-05	LWI	2.6051E-05	VKD	7.48414E-06
RIN	9.97448E-05	NAQ	2.6051E-05	VKM	7.48414E-06
RKM	9.97448E-05	NIV	2.6051E-05	WDY	7.48414E-06
SDQ	9.97448E-05	NMT	2.6051E-05	WSM	7.48414E-06
WSM	9.97448E-05	PKA	2.6051E-05	YGP	7.48414E-06
WSW	9.97448E-05	PTE	2.6051E-05	YLF	7.48414E-06
CLH	9.93111E-05	QER	2.6051E-05	YLQ	7.48414E-06
ESD	9.93111E-05	RIT	2.6051E-05	YMG	7.48414E-06
ESE	9.93111E-05	RVD	2.6051E-05	YQS	7.48414E-06
IPG	9.93111E-05	THE	2.6051E-05	YRD	7.48414E-06
KRK	9.93111E-05	TNH	2.6051E-05	YRM	7.48414E-06
LYN	9.93111E-05	WAQ	2.6051E-05	YTP	7.48414E-06
MLM	9.93111E-05	WRV	2.6051E-05	YYF	7.48414E-06
MNL	9.93111E-05	WVY	2.6051E-05	AMV	7.23467E-06
QFS	9.93111E-05	AEQ	2.5694E-05	ANQ	7.23467E-06
RCW	9.93111E-05	CLF	2.5694E-05	APK	7.23467E-06
SCM	9.93111E-05	DCL	2.5694E-05	ASW	7.23467E-06
TCG	9.93111E-05	DGS	2.5694E-05	AYR	7.23467E-06
TGY	9.93111E-05	FAD	2.5694E-05	CDH	7.23467E-06
TQV	9.93111E-05	FFS	2.5694E-05	CHL	7.23467E-06
YRH	9.93111E-05	FIR	2.5694E-05	CRV	7.23467E-06
YSK	9.93111E-05	FNH	2.5694E-05	DED	7.23467E-06
DDT	9.88774E-05	FVV	2.5694E-05	DEF	7.23467E-06
FRF	9.88774E-05	GAQ	2.5694E-05	DLW	7.23467E-06
FWT	9.88774E-05	GDM	2.5694E-05	DPL	7.23467E-06
HSF	9.88774E-05	GIN	2.5694E-05	DPT	7.23467E-06
ICL	9.88774E-05	GMT	2.5694E-05	DQT	7.23467E-06
KPM	9.88774E-05	HKT	2.5694E-05	DRA	7.23467E-06
KRY	9.88774E-05	IGE	2.5694E-05	DRR	7.23467E-06
KSK	9.88774E-05	ILV	2.5694E-05	ESN	7.23467E-06
KWR	9.88774E-05	KAA	2.5694E-05	FCE	7.23467E-06
MDR	9.88774E-05	KFA	2.5694E-05	FDT	7.23467E-06
MFS	9.88774E-05	KRQ	2.5694E-05	FIV	7.23467E-06
MNR	9.88774E-05	LKC	2.5694E-05	FIY	7.23467E-06

MTG	9.88774E-05	LWY	2.5694E-05	FMA	7.23467E-06
QWS	9.88774E-05	MDR	2.5694E-05	FTD	7.23467E-06
RDC	9.88774E-05	MSN	2.5694E-05	FVQ	7.23467E-06
SEQ	9.88774E-05	NRY	2.5694E-05	FYI	7.23467E-06
WMS	9.88774E-05	PGN	2.5694E-05	GAT	7.23467E-06
YMT	9.88774E-05	PNC	2.5694E-05	GCH	7.23467E-06
YSI	9.88774E-05	QCW	2.5694E-05	GFC	7.23467E-06
CES	9.84438E-05	QFP	2.5694E-05	GVA	7.23467E-06
CIR	9.84438E-05	RFF	2.5694E-05	GVD	7.23467E-06
CSC	9.84438E-05	SAY	2.5694E-05	GVK	7.23467E-06
CWL	9.84438E-05	SDI	2.5694E-05	HDQ	7.23467E-06
HHP	9.84438E-05	SVP	2.5694E-05	HEP	7.23467E-06
HRF	9.84438E-05	TIH	2.5694E-05	HTG	7.23467E-06
KLF	9.84438E-05	TMA	2.5694E-05	IES	7.23467E-06
LHQ	9.84438E-05	VEQ	2.5694E-05	IKC	7.23467E-06
MIL	9.84438E-05	VET	2.5694E-05	IMP	7.23467E-06
NHP	9.84438E-05	VQA	2.5694E-05	IQT	7.23467E-06
NPQ	9.84438E-05	WRA	2.5694E-05	IVW	7.23467E-06
RNM	9.84438E-05	YPA	2.5694E-05	KFA	7.23467E-06
SDE	9.84438E-05	ASH	2.5337E-05	KRC	7.23467E-06
SMC	9.84438E-05	AWN	2.5337E-05	LET	7.23467E-06
AAY	9.80101E-05	DEH	2.5337E-05	LFA	7.23467E-06
CLE	9.80101E-05	DSD	2.5337E-05	LMF	7.23467E-06
DDA	9.80101E-05	DSI	2.5337E-05	LMM	7.23467E-06
DSY	9.80101E-05	ERC	2.5337E-05	LVV	7.23467E-06
HDP	9.80101E-05	FNY	2.5337E-05	MQS	7.23467E-06
HHA	9.80101E-05	FRE	2.5337E-05	NDS	7.23467E-06
HTC	9.80101E-05	FYA	2.5337E-05	NFL	7.23467E-06
KSY	9.80101E-05	IMT	2.5337E-05	NRH	7.23467E-06
LFQ	9.80101E-05	IMV	2.5337E-05	NWV	7.23467E-06
MCS	9.80101E-05	LPI	2.5337E-05	PCD	7.23467E-06
PGF	9.80101E-05	MGG	2.5337E-05	PDS	7.23467E-06
SYM	9.80101E-05	MGN	2.5337E-05	PGD	7.23467E-06
VWG	9.80101E-05	MLT	2.5337E-05	PNN	7.23467E-06
YAM	9.80101E-05	NHG	2.5337E-05	PVD	7.23467E-06
IWS	9.75764E-05	NRD	2.5337E-05	QCL	7.23467E-06
LCH	9.75764E-05	PDM	2.5337E-05	QNN	7.23467E-06
LCI	9.75764E-05	QAN	2.5337E-05	QQG	7.23467E-06
LDI	9.75764E-05	QDT	2.5337E-05	QTA	7.23467E-06
LNI	9.75764E-05	RWA	2.5337E-05	RDD	7.23467E-06
NRF	9.75764E-05	TPM	2.5337E-05	RDS	7.23467E-06
NVC	9.75764E-05	WAE	2.5337E-05	RGG	7.23467E-06
PEG	9.75764E-05	WEQ	2.5337E-05	RGY	7.23467E-06
QQS	9.75764E-05	WNA	2.5337E-05	RMW	7.23467E-06

RHC	9.75764E-05	WNQ	2.5337E-05	RPE	7.23467E-06
RYQ	9.75764E-05	YAC	2.5337E-05	RRF	7.23467E-06
SFM	9.75764E-05	YPG	2.5337E-05	RWG	7.23467E-06
TAI	9.75764E-05	CRR	2.498E-05	RYY	7.23467E-06
WRC	9.75764E-05	CSC	2.498E-05	SAH	7.23467E-06
WRD	9.75764E-05	DVL	2.498E-05	SCC	7.23467E-06
FGM	9.71427E-05	EGD	2.498E-05	SDQ	7.23467E-06
FRE	9.71427E-05	FCT	2.498E-05	SPK	7.23467E-06
HLY	9.71427E-05	FNM	2.498E-05	SQQ	7.23467E-06
KRI	9.71427E-05	FTQ	2.498E-05	STG	7.23467E-06
LIW	9.71427E-05	GYF	2.498E-05	STW	7.23467E-06
LQQ	9.71427E-05	HCR	2.498E-05	SVW	7.23467E-06
NCP	9.71427E-05	HMQ	2.498E-05	TAT	7.23467E-06
NET	9.71427E-05	KHT	2.498E-05	TKC	7.23467E-06
NPH	9.71427E-05	KIS	2.498E-05	TLF	7.23467E-06
QHA	9.71427E-05	KKA	2.498E-05	TPH	7.23467E-06
RHW	9.71427E-05	KMM	2.498E-05	TSD	7.23467E-06
SNN	9.71427E-05	KMS	2.498E-05	TWT	7.23467E-06
SQH	9.71427E-05	KNF	2.498E-05	VCM	7.23467E-06
VGY	9.71427E-05	KPE	2.498E-05	VGK	7.23467E-06
YVM	9.71427E-05	KSW	2.498E-05	VIL	7.23467E-06
CGV	9.67091E-05	LFM	2.498E-05	VQA	7.23467E-06
ELY	9.67091E-05	LGF	2.498E-05	VTG	7.23467E-06
ERY	9.67091E-05	LYI	2.498E-05	WCN	7.23467E-06
FNP	9.67091E-05	NFS	2.498E-05	YAS	7.23467E-06
HQP	9.67091E-05	NQR	2.498E-05	YGG	7.23467E-06
HTK	9.67091E-05	NWT	2.498E-05	YKQ	7.23467E-06
LEN	9.67091E-05	PCV	2.498E-05	YNY	7.23467E-06
LKI	9.67091E-05	PCW	2.498E-05	YQQ	7.23467E-06
LWE	9.67091E-05	PDD	2.498E-05	AEK	6.98519E-06
MAG	9.67091E-05	PGH	2.498E-05	CHA	6.98519E-06
MDS	9.67091E-05	PPI	2.498E-05	CLH	6.98519E-06
NKP	9.67091E-05	RCE	2.498E-05	CMS	6.98519E-06
NYP	9.67091E-05	RGY	2.498E-05	CQH	6.98519E-06
PGI	9.67091E-05	RNE	2.498E-05	CSH	6.98519E-06
RIH	9.67091E-05	RNG	2.498E-05	CWM	6.98519E-06
RND	9.67091E-05	RVC	2.498E-05	DEW	6.98519E-06
SCW	9.67091E-05	SIP	2.498E-05	DKY	6.98519E-06
SFC	9.67091E-05	TDG	2.498E-05	DPC	6.98519E-06
WSH	9.67091E-05	TPQ	2.498E-05	DQA	6.98519E-06
WYL	9.67091E-05	WRG	2.498E-05	DTQ	6.98519E-06
CCR	9.62754E-05	YRW	2.498E-05	DVM	6.98519E-06
CYR	9.62754E-05	DLF	2.4623E-05	EKD	6.98519E-06
EHT	9.62754E-05	EGV	2.4623E-05	FMK	6.98519E-06

IQL	9.62754E-05	FLH	2.4623E-05	FQF	6.98519E-06
KHT	9.62754E-05	FVL	2.4623E-05	GDW	6.98519E-06
LDD	9.62754E-05	GFA	2.4623E-05	GGM	6.98519E-06
LEM	9.62754E-05	HGS	2.4623E-05	GPK	6.98519E-06
LKF	9.62754E-05	IQT	2.4623E-05	GRK	6.98519E-06
LWK	9.62754E-05	KLC	2.4623E-05	GRN	6.98519E-06
NFP	9.62754E-05	KLF	2.4623E-05	GYE	6.98519E-06
YIT	9.62754E-05	KWR	2.4623E-05	HCG	6.98519E-06
CSQ	9.58417E-05	LCK	2.4623E-05	HSH	6.98519E-06
HTW	9.58417E-05	LDG	2.4623E-05	IAT	6.98519E-06
ILY	9.58417E-05	NCQ	2.4623E-05	IFH	6.98519E-06
LMI	9.58417E-05	NGF	2.4623E-05	IGS	6.98519E-06
LQE	9.58417E-05	QEM	2.4623E-05	ILA	6.98519E-06
MFR	9.58417E-05	QLA	2.4623E-05	ILH	6.98519E-06
NAM	9.58417E-05	QPW	2.4623E-05	IRK	6.98519E-06
NIP	9.58417E-05	QRP	2.4623E-05	ISG	6.98519E-06
PAY	9.58417E-05	QRY	2.4623E-05	ITY	6.98519E-06
RDM	9.58417E-05	RAF	2.4623E-05	KNL	6.98519E-06
SEW	9.58417E-05	SCV	2.4623E-05	KPL	6.98519E-06
TIV	9.58417E-05	SGM	2.4623E-05	KPR	6.98519E-06
WRF	9.58417E-05	SNI	2.4623E-05	LND	6.98519E-06
CKS	9.54081E-05	SPW	2.4623E-05	MPL	6.98519E-06
CRW	9.54081E-05	SWF	2.4623E-05	MSF	6.98519E-06
DKT	9.54081E-05	TAK	2.4623E-05	MSP	6.98519E-06
DWS	9.54081E-05	VAT	2.4623E-05	MSV	6.98519E-06
ENP	9.54081E-05	VNF	2.4623E-05	MWV	6.98519E-06
HRI	9.54081E-05	VYL	2.4623E-05	NAT	6.98519E-06
LEE	9.54081E-05	VYP	2.4623E-05	NFP	6.98519E-06
LIQ	9.54081E-05	WLF	2.4623E-05	NHE	6.98519E-06
LQW	9.54081E-05	YPC	2.4623E-05	NSV	6.98519E-06
MFL	9.54081E-05	ACT	2.4267E-05	NTW	6.98519E-06
MLW	9.54081E-05	AIV	2.4267E-05	NYG	6.98519E-06
MMS	9.54081E-05	AMT	2.4267E-05	PAE	6.98519E-06
MYL	9.54081E-05	CSW	2.4267E-05	PHE	6.98519E-06
NPM	9.54081E-05	EED	2.4267E-05	PRI	6.98519E-06
NSY	9.54081E-05	FKG	2.4267E-05	QAW	6.98519E-06
NVN	9.54081E-05	GDT	2.4267E-05	QCC	6.98519E-06
NYA	9.54081E-05	GMN	2.4267E-05	QCH	6.98519E-06
QSK	9.54081E-05	GNQ	2.4267E-05	QLN	6.98519E-06
SEM	9.54081E-05	HAA	2.4267E-05	QPL	6.98519E-06
SIH	9.54081E-05	HNW	2.4267E-05	QSK	6.98519E-06
TEG	9.54081E-05	IGV	2.4267E-05	QTT	6.98519E-06
WQR	9.54081E-05	IKS	2.4267E-05	RDF	6.98519E-06
YCP	9.54081E-05	IMP	2.4267E-05	RHQ	6.98519E-06

YSD	9.54081E-05	KDS	2.4267E-05	RIH	6.98519E-06
YVC	9.54081E-05	KVL	2.4267E-05	RPF	6.98519E-06
YYT	9.54081E-05	LWK	2.4267E-05	SCP	6.98519E-06
CLI	9.49744E-05	MGS	2.4267E-05	SFD	6.98519E-06
CRC	9.49744E-05	MNA	2.4267E-05	SQV	6.98519E-06
DTC	9.49744E-05	NTC	2.4267E-05	SYW	6.98519E-06
ERF	9.49744E-05	NYA	2.4267E-05	TSG	6.98519E-06
FVM	9.49744E-05	PIN	2.4267E-05	TTC	6.98519E-06
GGK	9.49744E-05	PMV	2.4267E-05	VGE	6.98519E-06
LKY	9.49744E-05	PNV	2.4267E-05	VKG	6.98519E-06
RIM	9.49744E-05	QLM	2.4267E-05	VLK	6.98519E-06
FKT	9.45407E-05	RGI	2.4267E-05	VMQ	6.98519E-06
FSD	9.45407E-05	SNH	2.4267E-05	VPA	6.98519E-06
HAQ	9.45407E-05	SNY	2.4267E-05	VQQ	6.98519E-06
HIA	9.45407E-05	SVQ	2.4267E-05	VWI	6.98519E-06
HPH	9.45407E-05	TQL	2.4267E-05	WEY	6.98519E-06
MGG	9.45407E-05	TWK	2.4267E-05	WIY	6.98519E-06
MLQ	9.45407E-05	VKF	2.4267E-05	WKD	6.98519E-06
NWT	9.45407E-05	VTF	2.4267E-05	WRN	6.98519E-06
QLY	9.45407E-05	WFR	2.4267E-05	WYE	6.98519E-06
QPC	9.45407E-05	WGF	2.4267E-05	YFA	6.98519E-06
RCQ	9.45407E-05	WGS	2.4267E-05	YKW	6.98519E-06
RHQ	9.45407E-05	WQL	2.4267E-05	YRT	6.98519E-06
SKC	9.45407E-05	WWH	2.4267E-05	YVH	6.98519E-06
CHS	9.4107E-05	WWQ	2.4267E-05	AAT	6.73572E-06
DEP	9.4107E-05	YNF	2.4267E-05	AEF	6.73572E-06
DHT	9.4107E-05	YPW	2.4267E-05	ATE	6.73572E-06
ESN	9.4107E-05	YSD	2.4267E-05	ATQ	6.73572E-06
FPM	9.4107E-05	YTK	2.4267E-05	AVK	6.73572E-06
FVC	9.4107E-05	CPM	2.391E-05	AWK	6.73572E-06
FWV	9.4107E-05	CSQ	2.391E-05	CCC	6.73572E-06
FYT	9.4107E-05	DCS	2.391E-05	CGK	6.73572E-06
HCT	9.4107E-05	EDT	2.391E-05	CLF	6.73572E-06
KTC	9.4107E-05	ENA	2.391E-05	CML	6.73572E-06
LMF	9.4107E-05	ENF	2.391E-05	DRQ	6.73572E-06
MHS	9.4107E-05	ENW	2.391E-05	EAD	6.73572E-06
MRC	9.4107E-05	ETA	2.391E-05	EGT	6.73572E-06
NDG	9.4107E-05	HNH	2.391E-05	EPN	6.73572E-06
NDV	9.4107E-05	HTD	2.391E-05	ERP	6.73572E-06
NVK	9.4107E-05	IGT	2.391E-05	EYG	6.73572E-06
RNQ	9.4107E-05	IKY	2.391E-05	FDP	6.73572E-06
RYH	9.4107E-05	KPC	2.391E-05	FLD	6.73572E-06
TGI	9.4107E-05	KQS	2.391E-05	GDD	6.73572E-06
CCL	9.36734E-05	KVS	2.391E-05	GEI	6.73572E-06

FTM	9.36734E-05	LDM	2.391E-05	GWW	6.73572E-06
GIG	9.36734E-05	LET	2.391E-05	HGW	6.73572E-06
HPC	9.36734E-05	LVF	2.391E-05	HHS	6.73572E-06
HPM	9.36734E-05	MSW	2.391E-05	HLA	6.73572E-06
HVW	9.36734E-05	NGK	2.391E-05	HTW	6.73572E-06
ICR	9.36734E-05	PAF	2.391E-05	HYH	6.73572E-06
ISK	9.36734E-05	QGS	2.391E-05	ICN	6.73572E-06
KTQ	9.36734E-05	RCA	2.391E-05	IVT	6.73572E-06
LIF	9.36734E-05	RED	2.391E-05	KAQ	6.73572E-06
LKH	9.36734E-05	RIV	2.391E-05	KPG	6.73572E-06
LNN	9.36734E-05	RKF	2.391E-05	KRT	6.73572E-06
MRK	9.36734E-05	SAV	2.391E-05	LAV	6.73572E-06
NRY	9.36734E-05	SCE	2.391E-05	LEA	6.73572E-06
NTC	9.36734E-05	SMP	2.391E-05	LFQ	6.73572E-06
PQV	9.36734E-05	TIL	2.391E-05	LPK	6.73572E-06
QDT	9.36734E-05	TQR	2.391E-05	LVC	6.73572E-06
RDK	9.36734E-05	VCD	2.391E-05	MLP	6.73572E-06
RKQ	9.36734E-05	VLV	2.391E-05	MPA	6.73572E-06
WES	9.36734E-05	VRC	2.391E-05	MVI	6.73572E-06
WLI	9.36734E-05	WHQ	2.391E-05	NDW	6.73572E-06
YTM	9.36734E-05	WNW	2.391E-05	NIS	6.73572E-06
AFG	9.32397E-05	AKQ	2.3553E-05	NPD	6.73572E-06
CKL	9.32397E-05	ALE	2.3553E-05	NTD	6.73572E-06
CSH	9.32397E-05	DFR	2.3553E-05	PAQ	6.73572E-06
CSN	9.32397E-05	DIT	2.3553E-05	PIP	6.73572E-06
DHP	9.32397E-05	FPG	2.3553E-05	PMF	6.73572E-06
DPM	9.32397E-05	GDC	2.3553E-05	PQV	6.73572E-06
DRE	9.32397E-05	GTE	2.3553E-05	PVC	6.73572E-06
ENT	9.32397E-05	KHV	2.3553E-05	PVT	6.73572E-06
KQT	9.32397E-05	KKV	2.3553E-05	PYE	6.73572E-06
LEK	9.32397E-05	KNN	2.3553E-05	QRH	6.73572E-06
LFD	9.32397E-05	KTE	2.3553E-05	QRP	6.73572E-06
LHD	9.32397E-05	KWL	2.3553E-05	QYE	6.73572E-06
LIM	9.32397E-05	LCE	2.3553E-05	RCH	6.73572E-06
MGV	9.32397E-05	LNE	2.3553E-05	RCV	6.73572E-06
MIS	9.32397E-05	NHL	2.3553E-05	RDE	6.73572E-06
NPK	9.32397E-05	NPE	2.3553E-05	RET	6.73572E-06
SFD	9.32397E-05	NTD	2.3553E-05	REY	6.73572E-06
SHC	9.32397E-05	QLN	2.3553E-05	RFF	6.73572E-06
TMM	9.32397E-05	QPY	2.3553E-05	RMP	6.73572E-06
YCT	9.32397E-05	RDF	2.3553E-05	RND	6.73572E-06
YHA	9.32397E-05	RDK	2.3553E-05	RVA	6.73572E-06
AEV	9.2806E-05	RVT	2.3553E-05	SAP	6.73572E-06
AGY	9.2806E-05	SWQ	2.3553E-05	SHD	6.73572E-06

CYS	9.2806E-05	TCW	2.3553E-05	SIH	6.73572E-06
DTD	9.2806E-05	TTV	2.3553E-05	SNP	6.73572E-06
DVQ	9.2806E-05	VFS	2.3553E-05	SRK	6.73572E-06
DVW	9.2806E-05	VGD	2.3553E-05	THH	6.73572E-06
DYT	9.2806E-05	VHS	2.3553E-05	TNL	6.73572E-06
FDP	9.2806E-05	WWS	2.3553E-05	TPM	6.73572E-06
IRK	9.2806E-05	YVS	2.3553E-05	TRD	6.73572E-06
KSF	9.2806E-05	AYF	2.3196E-05	VAD	6.73572E-06
MRW	9.2806E-05	CGD	2.3196E-05	VEK	6.73572E-06
QMT	9.2806E-05	DDQ	2.3196E-05	VLH	6.73572E-06
QWL	9.2806E-05	EDV	2.3196E-05	VPQ	6.73572E-06
RKN	9.2806E-05	ELD	2.3196E-05	VQH	6.73572E-06
YQT	9.2806E-05	EMH	2.3196E-05	WMH	6.73572E-06
AVY	9.23723E-05	ETN	2.3196E-05	WYY	6.73572E-06
CWR	9.23723E-05	EVT	2.3196E-05	YCD	6.73572E-06
DCA	9.23723E-05	FWH	2.3196E-05	AKI	6.48625E-06
DMV	9.23723E-05	GYP	2.3196E-05	AKK	6.48625E-06
FMT	9.23723E-05	HHR	2.3196E-05	ATK	6.48625E-06
FPQ	9.23723E-05	KDW	2.3196E-05	CCW	6.48625E-06
KDT	9.23723E-05	KGE	2.3196E-05	CES	6.48625E-06
KET	9.23723E-05	LMA	2.3196E-05	CKF	6.48625E-06
LFK	9.23723E-05	LNM	2.3196E-05	CLP	6.48625E-06
NCA	9.23723E-05	MGD	2.3196E-05	CNL	6.48625E-06
QRK	9.23723E-05	NEP	2.3196E-05	CSQ	6.48625E-06
QSF	9.23723E-05	NSE	2.3196E-05	CWH	6.48625E-06
RCH	9.23723E-05	QGL	2.3196E-05	DML	6.48625E-06
REQ	9.23723E-05	RCT	2.3196E-05	DNQ	6.48625E-06
REW	9.23723E-05	SGC	2.3196E-05	DTD	6.48625E-06
RQK	9.23723E-05	SKY	2.3196E-05	DWP	6.48625E-06
SIC	9.23723E-05	TKP	2.3196E-05	DYI	6.48625E-06
SMN	9.23723E-05	TNF	2.3196E-05	EHH	6.48625E-06
YCA	9.23723E-05	VDQ	2.3196E-05	EMR	6.48625E-06
YRD	9.23723E-05	VPE	2.3196E-05	EMY	6.48625E-06
DIP	9.19387E-05	VWP	2.3196E-05	ESF	6.48625E-06
DTQ	9.19387E-05	WCR	2.3196E-05	FEG	6.48625E-06
ERH	9.19387E-05	YEP	2.3196E-05	GCT	6.48625E-06
FKP	9.19387E-05	YSN	2.3196E-05	GCV	6.48625E-06
HFP	9.19387E-05	AND	2.2839E-05	GPQ	6.48625E-06
HNA	9.19387E-05	DGP	2.2839E-05	HHE	6.48625E-06
ILI	9.19387E-05	DPP	2.2839E-05	HLS	6.48625E-06
ISQ	9.19387E-05	EAK	2.2839E-05	HRG	6.48625E-06
KFT	9.19387E-05	ENS	2.2839E-05	HRW	6.48625E-06
LEY	9.19387E-05	EQT	2.2839E-05	HTH	6.48625E-06
LYH	9.19387E-05	EVS	2.2839E-05	HTN	6.48625E-06

NAQ	9.19387E-05	FLM	2.2839E-05	INM	6.48625E-06
RFM	9.19387E-05	FPV	2.2839E-05	IVG	6.48625E-06
RKI	9.19387E-05	FSK	2.2839E-05	KAD	6.48625E-06
SKN	9.19387E-05	FVR	2.2839E-05	KIT	6.48625E-06
SME	9.19387E-05	IDT	2.2839E-05	KWP	6.48625E-06
TEV	9.19387E-05	IFM	2.2839E-05	KYG	6.48625E-06
YHP	9.19387E-05	IGP	2.2839E-05	LIV	6.48625E-06
YRY	9.19387E-05	KGC	2.2839E-05	LKE	6.48625E-06
DCP	9.1505E-05	LCC	2.2839E-05	LKV	6.48625E-06
DHA	9.1505E-05	LVC	2.2839E-05	LMP	6.48625E-06
DKA	9.1505E-05	MHS	2.2839E-05	LQY	6.48625E-06
EFT	9.1505E-05	NNF	2.2839E-05	MRG	6.48625E-06
IQR	9.1505E-05	NNR	2.2839E-05	MYT	6.48625E-06
ISD	9.1505E-05	NPV	2.2839E-05	NDN	6.48625E-06
ISH	9.1505E-05	PMG	2.2839E-05	NPP	6.48625E-06
ISN	9.1505E-05	PWP	2.2839E-05	NQP	6.48625E-06
KTM	9.1505E-05	PYM	2.2839E-05	NSF	6.48625E-06
LQF	9.1505E-05	QGE	2.2839E-05	PTK	6.48625E-06
LQH	9.1505E-05	QLQ	2.2839E-05	PWC	6.48625E-06
LYE	9.1505E-05	QRF	2.2839E-05	PWQ	6.48625E-06
MCR	9.1505E-05	RID	2.2839E-05	QCE	6.48625E-06
MRM	9.1505E-05	RRM	2.2839E-05	QFS	6.48625E-06
NAK	9.1505E-05	TAF	2.2839E-05	QPP	6.48625E-06
QNP	9.1505E-05	TER	2.2839E-05	QRQ	6.48625E-06
RCM	9.1505E-05	TFN	2.2839E-05	RHC	6.48625E-06
RDQ	9.1505E-05	TMR	2.2839E-05	RRI	6.48625E-06
RMH	9.1505E-05	TQS	2.2839E-05	RVK	6.48625E-06
RNH	9.1505E-05	TVW	2.2839E-05	SAY	6.48625E-06
RWW	9.1505E-05	VPW	2.2839E-05	SFS	6.48625E-06
SIK	9.1505E-05	VWW	2.2839E-05	SMH	6.48625E-06
WLK	9.1505E-05	YCS	2.2839E-05	SMY	6.48625E-06
YAQ	9.1505E-05	YNR	2.2839E-05	TCN	6.48625E-06
YDA	9.1505E-05	AEH	2.2482E-05	TDH	6.48625E-06
YWR	9.1505E-05	AHF	2.2482E-05	TGH	6.48625E-06
CQR	9.10713E-05	ANM	2.2482E-05	TSR	6.48625E-06
DQT	9.10713E-05	CAL	2.2482E-05	VCQ	6.48625E-06
ESI	9.10713E-05	CPA	2.2482E-05	VEA	6.48625E-06
IFR	9.10713E-05	DGV	2.2482E-05	VGN	6.48625E-06
KIT	9.10713E-05	DMS	2.2482E-05	VHH	6.48625E-06
LDH	9.10713E-05	DPT	2.2482E-05	VIY	6.48625E-06
LHN	9.10713E-05	DYP	2.2482E-05	VLF	6.48625E-06
MEL	9.10713E-05	DYS	2.2482E-05	VVQ	6.48625E-06
MLH	9.10713E-05	FCP	2.2482E-05	WFQ	6.48625E-06
NMV	9.10713E-05	GFC	2.2482E-05	WMF	6.48625E-06

QER	9.10713E-05	GGN	2.2482E-05	WND	6.48625E-06
RDF	9.10713E-05	GIH	2.2482E-05	WRM	6.48625E-06
RKH	9.10713E-05	GQV	2.2482E-05	YDV	6.48625E-06
RMW	9.10713E-05	IAK	2.2482E-05	YLN	6.48625E-06
RMY	9.10713E-05	KAG	2.2482E-05	YNF	6.48625E-06
SDD	9.10713E-05	KWA	2.2482E-05	YQE	6.48625E-06
SNH	9.10713E-05	KYH	2.2482E-05	ADP	6.23678E-06
SQM	9.10713E-05	MAP	2.2482E-05	AEA	6.23678E-06
DIA	9.06377E-05	MEL	2.2482E-05	AFL	6.23678E-06
EHP	9.06377E-05	NTH	2.2482E-05	AMC	6.23678E-06
EPH	9.06377E-05	PWA	2.2482E-05	ANI	6.23678E-06
FGW	9.06377E-05	SFH	2.2482E-05	ANV	6.23678E-06
GEG	9.06377E-05	SPV	2.2482E-05	APE	6.23678E-06
GFV	9.06377E-05	SQA	2.2482E-05	AVS	6.23678E-06
GGI	9.06377E-05	SVD	2.2482E-05	CIS	6.23678E-06
GWV	9.06377E-05	THL	2.2482E-05	CVQ	6.23678E-06
HMA	9.06377E-05	TMG	2.2482E-05	DDW	6.23678E-06
ISE	9.06377E-05	TNR	2.2482E-05	DKS	6.23678E-06
KYT	9.06377E-05	VIR	2.2482E-05	DRP	6.23678E-06
LHK	9.06377E-05	VRM	2.2482E-05	DSY	6.23678E-06
LNF	9.06377E-05	WAY	2.2482E-05	EEN	6.23678E-06
LWN	9.06377E-05	YIF	2.2482E-05	EHV	6.23678E-06
MYS	9.06377E-05	YSM	2.2482E-05	EKN	6.23678E-06
QIT	9.06377E-05	YTD	2.2482E-05	EML	6.23678E-06
RFC	9.06377E-05	YWH	2.2482E-05	EQS	6.23678E-06
RMK	9.06377E-05	AEY	2.2125E-05	EQV	6.23678E-06
SMK	9.06377E-05	AGM	2.2125E-05	ESE	6.23678E-06
YKT	9.06377E-05	APF	2.2125E-05	FEA	6.23678E-06
YRI	9.06377E-05	CGQ	2.2125E-05	FLN	6.23678E-06
EMT	9.0204E-05	CNS	2.2125E-05	GTW	6.23678E-06
ETN	9.0204E-05	CQA	2.2125E-05	HDA	6.23678E-06
FRY	9.0204E-05	CSR	2.2125E-05	HGQ	6.23678E-06
HVD	9.0204E-05	ESC	2.2125E-05	HLN	6.23678E-06
KHA	9.0204E-05	FGH	2.2125E-05	HTA	6.23678E-06
LFF	9.0204E-05	FGM	2.2125E-05	HTI	6.23678E-06
LIN	9.0204E-05	FMA	2.2125E-05	IKK	6.23678E-06
NFA	9.0204E-05	GDG	2.2125E-05	IRI	6.23678E-06
NHV	9.0204E-05	GWT	2.2125E-05	KET	6.23678E-06
NNV	9.0204E-05	ITN	2.2125E-05	KGD	6.23678E-06
NPN	9.0204E-05	KVR	2.2125E-05	KYH	6.23678E-06
NVH	9.0204E-05	LFW	2.2125E-05	LIC	6.23678E-06
PVY	9.0204E-05	MER	2.2125E-05	LIW	6.23678E-06
QRN	9.0204E-05	MES	2.2125E-05	LQF	6.23678E-06
RFW	9.0204E-05	MNP	2.2125E-05	MKL	6.23678E-06

RMF	9.0204E-05	MRE	2.2125E-05	MLM	6.23678E-06
SCD	9.0204E-05	NEM	2.2125E-05	MLV	6.23678E-06
SMD	9.0204E-05	NGN	2.2125E-05	MMK	6.23678E-06
SND	9.0204E-05	NSY	2.2125E-05	MRV	6.23678E-06
SQQ	9.0204E-05	NWS	2.2125E-05	MTF	6.23678E-06
VDW	9.0204E-05	NWY	2.2125E-05	MWM	6.23678E-06
WWR	9.0204E-05	PAK	2.2125E-05	NCR	6.23678E-06
YKP	9.0204E-05	PFW	2.2125E-05	NEA	6.23678E-06
YNA	9.0204E-05	QDQ	2.2125E-05	NKC	6.23678E-06
EHA	8.97703E-05	QPD	2.2125E-05	NLC	6.23678E-06
FCT	8.97703E-05	QYG	2.2125E-05	NPR	6.23678E-06
HAW	8.97703E-05	RND	2.2125E-05	NRK	6.23678E-06
HCP	8.97703E-05	RRI	2.2125E-05	NVE	6.23678E-06
HTQ	8.97703E-05	RTI	2.2125E-05	NVM	6.23678E-06
IES	8.97703E-05	RYY	2.2125E-05	PAC	6.23678E-06
IRI	8.97703E-05	SQG	2.2125E-05	PKN	6.23678E-06
IRQ	8.97703E-05	SYE	2.2125E-05	PNQ	6.23678E-06
KVM	8.97703E-05	THN	2.2125E-05	PTE	6.23678E-06
KYP	8.97703E-05	TNV	2.2125E-05	PTW	6.23678E-06
MLD	8.97703E-05	TPY	2.2125E-05	PWH	6.23678E-06
MSW	8.97703E-05	TVD	2.2125E-05	PYW	6.23678E-06
NTK	8.97703E-05	TVY	2.2125E-05	QHW	6.23678E-06
NTN	8.97703E-05	VLW	2.2125E-05	QIC	6.23678E-06
QWR	8.97703E-05	VQP	2.2125E-05	QND	6.23678E-06
RIC	8.97703E-05	VTK	2.2125E-05	QNV	6.23678E-06
RKF	8.97703E-05	YHP	2.2125E-05	RDP	6.23678E-06
SHQ	8.97703E-05	AGN	2.1769E-05	RGC	6.23678E-06
SKE	8.97703E-05	ANY	2.1769E-05	RPV	6.23678E-06
SWC	8.97703E-05	ARC	2.1769E-05	RQY	6.23678E-06
TWG	8.97703E-05	ATH	2.1769E-05	SCA	6.23678E-06
YPW	8.97703E-05	CRV	2.1769E-05	SCV	6.23678E-06
DAQ	8.93366E-05	DAD	2.1769E-05	SIC	6.23678E-06
DKP	8.93366E-05	DEY	2.1769E-05	SKN	6.23678E-06
DRF	8.93366E-05	DPW	2.1769E-05	SMW	6.23678E-06
DTE	8.93366E-05	EAH	2.1769E-05	SPI	6.23678E-06
ESY	8.93366E-05	EIF	2.1769E-05	SQA	6.23678E-06
FHT	8.93366E-05	EPK	2.1769E-05	SRN	6.23678E-06
HDA	8.93366E-05	ETK	2.1769E-05	STQ	6.23678E-06
IPW	8.93366E-05	FGC	2.1769E-05	SYH	6.23678E-06
IQS	8.93366E-05	GIY	2.1769E-05	TFY	6.23678E-06
KIP	8.93366E-05	HAT	2.1769E-05	TLH	6.23678E-06
LND	8.93366E-05	HDR	2.1769E-05	TPY	6.23678E-06
LYF	8.93366E-05	HRG	2.1769E-05	TVH	6.23678E-06
MLE	8.93366E-05	HYP	2.1769E-05	TVW	6.23678E-06

MLN	8.93366E-05	IQL	2.1769E-05	VDW	6.23678E-06
NIA	8.93366E-05	IYP	2.1769E-05	VFR	6.23678E-06
RCN	8.93366E-05	KFQ	2.1769E-05	VLW	6.23678E-06
REM	8.93366E-05	KWY	2.1769E-05	VRI	6.23678E-06
RFN	8.93366E-05	LEA	2.1769E-05	VSG	6.23678E-06
RHD	8.93366E-05	LVN	2.1769E-05	VSI	6.23678E-06
RNK	8.93366E-05	MAA	2.1769E-05	VWY	6.23678E-06
SCE	8.93366E-05	MAL	2.1769E-05	WFP	6.23678E-06
SDF	8.93366E-05	MGQ	2.1769E-05	WHV	6.23678E-06
SQW	8.93366E-05	NAY	2.1769E-05	WKV	6.23678E-06
YTC	8.93366E-05	NDT	2.1769E-05	WWD	6.23678E-06
CER	8.8903E-05	NTW	2.1769E-05	WWE	6.23678E-06
DTM	8.8903E-05	NWF	2.1769E-05	YGV	6.23678E-06
EIA	8.8903E-05	PKY	2.1769E-05	YHM	6.23678E-06
HNG	8.8903E-05	QFS	2.1769E-05	YIR	6.23678E-06
HPD	8.8903E-05	RDN	2.1769E-05	YKP	6.23678E-06
HVN	8.8903E-05	RYQ	2.1769E-05	YLD	6.23678E-06
IRC	8.8903E-05	SEH	2.1769E-05	YMT	6.23678E-06
KVC	8.8903E-05	SHH	2.1769E-05	YNH	6.23678E-06
LKE	8.8903E-05	SPY	2.1769E-05	YQD	6.23678E-06
MQS	8.8903E-05	THH	2.1769E-05	YYE	6.23678E-06
NTI	8.8903E-05	TNY	2.1769E-05	AER	5.98731E-06
QLI	8.8903E-05	TTQ	2.1769E-05	AQY	5.98731E-06
YET	8.8903E-05	TVH	2.1769E-05	CGE	5.98731E-06
YHT	8.8903E-05	TWG	2.1769E-05	CHQ	5.98731E-06
YPM	8.8903E-05	VIK	2.1769E-05	CRT	5.98731E-06
DPC	8.84693E-05	VLF	2.1769E-05	CTR	5.98731E-06
DTK	8.84693E-05	VYS	2.1769E-05	CVV	5.98731E-06
DWT	8.84693E-05	WIT	2.1769E-05	CYH	5.98731E-06
ERI	8.84693E-05	WTQ	2.1769E-05	DDG	5.98731E-06
GDQ	8.84693E-05	WVT	2.1769E-05	DGT	5.98731E-06
GNF	8.84693E-05	YAW	2.1769E-05	DHH	5.98731E-06
HPE	8.84693E-05	YEY	2.1769E-05	DHV	5.98731E-06
ILE	8.84693E-05	ATE	2.1412E-05	DKT	5.98731E-06
KAC	8.84693E-05	CLG	2.1412E-05	DLG	5.98731E-06
LCF	8.84693E-05	CSI	2.1412E-05	DQH	5.98731E-06
LEF	8.84693E-05	CYF	2.1412E-05	EEG	5.98731E-06
LQM	8.84693E-05	ECV	2.1412E-05	EES	5.98731E-06
LWI	8.84693E-05	EMG	2.1412E-05	EFS	5.98731E-06
MLI	8.84693E-05	FAP	2.1412E-05	EGH	5.98731E-06
QES	8.84693E-05	FCA	2.1412E-05	EHL	5.98731E-06
QPW	8.84693E-05	FSH	2.1412E-05	EIR	5.98731E-06
QSD	8.84693E-05	GCP	2.1412E-05	ELR	5.98731E-06
RHK	8.84693E-05	GGM	2.1412E-05	EPF	5.98731E-06

RNI	8.84693E-05	GNK	2.1412E-05	EWT	5.98731E-06
SEC	8.84693E-05	GQG	2.1412E-05	FHC	5.98731E-06
SIE	8.84693E-05	HKS	2.1412E-05	FNM	5.98731E-06
SWQ	8.84693E-05	HLW	2.1412E-05	FQI	5.98731E-06
YIP	8.84693E-05	IHR	2.1412E-05	FTA	5.98731E-06
YSY	8.84693E-05	KEL	2.1412E-05	GMD	5.98731E-06
CQL	8.80356E-05	KWI	2.1412E-05	GTM	5.98731E-06
DAM	8.80356E-05	NPD	2.1412E-05	GWF	5.98731E-06
DDP	8.80356E-05	NVS	2.1412E-05	HKQ	5.98731E-06
DPW	8.80356E-05	RMT	2.1412E-05	HPS	5.98731E-06
EKT	8.80356E-05	SCD	2.1412E-05	HQA	5.98731E-06
ESF	8.80356E-05	SGY	2.1412E-05	IGT	5.98731E-06
ETH	8.80356E-05	SKC	2.1412E-05	IQE	5.98731E-06
EYT	8.80356E-05	SNQ	2.1412E-05	ISA	5.98731E-06
FVW	8.80356E-05	SPD	2.1412E-05	ISI	5.98731E-06
IRN	8.80356E-05	SPI	2.1412E-05	IWA	5.98731E-06
KAW	8.80356E-05	TDW	2.1412E-05	KAP	5.98731E-06
KMA	8.80356E-05	TEA	2.1412E-05	KDT	5.98731E-06
NHA	8.80356E-05	VDP	2.1412E-05	KDY	5.98731E-06
NWP	8.80356E-05	VGS	2.1412E-05	KGT	5.98731E-06
RCC	8.80356E-05	VPQ	2.1412E-05	KPC	5.98731E-06
RDE	8.80356E-05	VSD	2.1412E-05	KQT	5.98731E-06
RQC	8.80356E-05	WEL	2.1412E-05	KYL	5.98731E-06
SFQ	8.80356E-05	WMW	2.1412E-05	LFI	5.98731E-06
SWW	8.80356E-05	WNC	2.1412E-05	LHM	5.98731E-06
WLF	8.80356E-05	WYQ	2.1412E-05	LKI	5.98731E-06
WNT	8.80356E-05	YFW	2.1412E-05	LKK	5.98731E-06
CRK	8.76019E-05	YGH	2.1412E-05	LLE	5.98731E-06
FEP	8.76019E-05	AMA	2.1055E-05	LNI	5.98731E-06
FHP	8.76019E-05	CVW	2.1055E-05	LVM	5.98731E-06
HKA	8.76019E-05	DNH	2.1055E-05	MNL	5.98731E-06
IKT	8.76019E-05	EDK	2.1055E-05	MNN	5.98731E-06
KQA	8.76019E-05	EPM	2.1055E-05	NAW	5.98731E-06
LID	8.76019E-05	EVY	2.1055E-05	NFW	5.98731E-06
NGM	8.76019E-05	EYF	2.1055E-05	NNN	5.98731E-06
NVD	8.76019E-05	FTH	2.1055E-05	NVD	5.98731E-06
RHI	8.76019E-05	GHH	2.1055E-05	NYC	5.98731E-06
SCQ	8.76019E-05	GKK	2.1055E-05	PDH	5.98731E-06
SKK	8.76019E-05	HAL	2.1055E-05	PGY	5.98731E-06
SWE	8.76019E-05	IQP	2.1055E-05	PYC	5.98731E-06
TNM	8.76019E-05	IVL	2.1055E-05	QCS	5.98731E-06
WKT	8.76019E-05	KAV	2.1055E-05	QRV	5.98731E-06
WLE	8.76019E-05	KLH	2.1055E-05	QSI	5.98731E-06
YSF	8.76019E-05	KQL	2.1055E-05	RAI	5.98731E-06

CLF	8.71683E-05	KRM	2.1055E-05	RCP	5.98731E-06
DMP	8.71683E-05	KRW	2.1055E-05	RMA	5.98731E-06
DTY	8.71683E-05	KWS	2.1055E-05	RRE	5.98731E-06
FYP	8.71683E-05	LEF	2.1055E-05	RWV	5.98731E-06
HET	8.71683E-05	LYC	2.1055E-05	RWY	5.98731E-06
ILF	8.71683E-05	MQL	2.1055E-05	RYI	5.98731E-06
IRH	8.71683E-05	MRV	2.1055E-05	SCG	5.98731E-06
LKD	8.71683E-05	NDL	2.1055E-05	SGC	5.98731E-06
LWH	8.71683E-05	NKQ	2.1055E-05	SHC	5.98731E-06
MLK	8.71683E-05	NNY	2.1055E-05	SIF	5.98731E-06
NRI	8.71683E-05	NWR	2.1055E-05	SKD	5.98731E-06
QSE	8.71683E-05	PSM	2.1055E-05	SMK	5.98731E-06
QSY	8.71683E-05	PWV	2.1055E-05	SNW	5.98731E-06
RHM	8.71683E-05	QDH	2.1055E-05	SSI	5.98731E-06
RWM	8.71683E-05	QPN	2.1055E-05	SWE	5.98731E-06
RYC	8.71683E-05	RHG	2.1055E-05	SWI	5.98731E-06
SMH	8.71683E-05	RHQ	2.1055E-05	TAR	5.98731E-06
WSK	8.71683E-05	RME	2.1055E-05	TCL	5.98731E-06
AYG	8.67346E-05	RWN	2.1055E-05	TFL	5.98731E-06
CRQ	8.67346E-05	SFI	2.1055E-05	TIS	5.98731E-06
DFP	8.67346E-05	TET	2.1055E-05	TLQ	5.98731E-06
DMG	8.67346E-05	TKA	2.1055E-05	TTD	5.98731E-06
ENA	8.67346E-05	TTN	2.1055E-05	TTN	5.98731E-06
EWS	8.67346E-05	TTY	2.1055E-05	VAC	5.98731E-06
FPC	8.67346E-05	VAE	2.1055E-05	VDR	5.98731E-06
HTY	8.67346E-05	VSQ	2.1055E-05	VGY	5.98731E-06
HVM	8.67346E-05	VSW	2.1055E-05	VHW	5.98731E-06
IER	8.67346E-05	WAR	2.1055E-05	VLN	5.98731E-06
KDP	8.67346E-05	WFH	2.1055E-05	VNA	5.98731E-06
KWT	8.67346E-05	WSN	2.1055E-05	VRE	5.98731E-06
LIE	8.67346E-05	WSQ	2.1055E-05	WED	5.98731E-06
LNY	8.67346E-05	YEL	2.1055E-05	YAF	5.98731E-06
NAH	8.67346E-05	YFP	2.1055E-05	YPF	5.98731E-06
NPC	8.67346E-05	YHD	2.1055E-05	YWF	5.98731E-06
NPW	8.67346E-05	YNG	2.1055E-05	AAA	5.73784E-06
RHE	8.67346E-05	YPQ	2.1055E-05	AHD	5.73784E-06
RKK	8.67346E-05	YWF	2.1055E-05	ASV	5.73784E-06
SFN	8.67346E-05	AAE	2.0698E-05	AVV	5.73784E-06
SIY	8.67346E-05	AHP	2.0698E-05	AWQ	5.73784E-06
SYC	8.67346E-05	ANE	2.0698E-05	CCA	5.73784E-06
YPK	8.67346E-05	ANQ	2.0698E-05	DCA	5.73784E-06
YTW	8.67346E-05	ATF	2.0698E-05	DCR	5.73784E-06
YVW	8.67346E-05	CKR	2.0698E-05	DCW	5.73784E-06
DNV	8.63009E-05	CMQ	2.0698E-05	DDE	5.73784E-06

DTN	8.63009E-05	CSP	2.0698E-05	DKP	5.73784E-06
FIT	8.63009E-05	EPP	2.0698E-05	DNH	5.73784E-06
FPW	8.63009E-05	ESW	2.0698E-05	DQS	5.73784E-06
HFA	8.63009E-05	EWT	2.0698E-05	EHT	5.73784E-06
HPW	8.63009E-05	FFV	2.0698E-05	ELP	5.73784E-06
HVC	8.63009E-05	FIP	2.0698E-05	EMG	5.73784E-06
IFS	8.63009E-05	GGY	2.0698E-05	FDQ	5.73784E-06
KCT	8.63009E-05	GHD	2.0698E-05	FHE	5.73784E-06
KIA	8.63009E-05	HRD	2.0698E-05	FHY	5.73784E-06
KVW	8.63009E-05	IML	2.0698E-05	FKN	5.73784E-06
LFN	8.63009E-05	IQY	2.0698E-05	FNQ	5.73784E-06
LKN	8.63009E-05	KAC	2.0698E-05	FVP	5.73784E-06
MER	8.63009E-05	KTG	2.0698E-05	GFR	5.73784E-06
QLF	8.63009E-05	LEQ	2.0698E-05	GVE	5.73784E-06
QRF	8.63009E-05	LQA	2.0698E-05	GVW	5.73784E-06
QRH	8.63009E-05	MCL	2.0698E-05	HEN	5.73784E-06
QRI	8.63009E-05	MNH	2.0698E-05	HHW	5.73784E-06
RDD	8.63009E-05	MQV	2.0698E-05	HPH	5.73784E-06
RKD	8.63009E-05	NFC	2.0698E-05	IHG	5.73784E-06
RWK	8.63009E-05	NFI	2.0698E-05	IIN	5.73784E-06
SHD	8.63009E-05	PMY	2.0698E-05	ILC	5.73784E-06
SYH	8.63009E-05	QKP	2.0698E-05	ILI	5.73784E-06
VHM	8.63009E-05	RGK	2.0698E-05	IPM	5.73784E-06
VNM	8.63009E-05	RHD	2.0698E-05	ITK	5.73784E-06
WFT	8.63009E-05	RMD	2.0698E-05	KQS	5.73784E-06
WQS	8.63009E-05	RYH	2.0698E-05	LAA	5.73784E-06
WRK	8.63009E-05	SNW	2.0698E-05	LDM	5.73784E-06
WYT	8.63009E-05	SWG	2.0698E-05	LEV	5.73784E-06
CDT	8.58672E-05	TIP	2.0698E-05	LMA	5.73784E-06
CLY	8.58672E-05	TWR	2.0698E-05	LVP	5.73784E-06
CRD	8.58672E-05	VFR	2.0698E-05	MIT	5.73784E-06
EWR	8.58672E-05	VYT	2.0698E-05	MMM	5.73784E-06
FHA	8.58672E-05	WDN	2.0698E-05	MRP	5.73784E-06
HAM	8.58672E-05	YFG	2.0698E-05	MYS	5.73784E-06
HGW	8.58672E-05	YNT	2.0698E-05	NKK	5.73784E-06
HMP	8.58672E-05	AMQ	2.0341E-05	NLD	5.73784E-06
KDA	8.58672E-05	CAN	2.0341E-05	NPC	5.73784E-06
MDL	8.58672E-05	CFW	2.0341E-05	NPG	5.73784E-06
NQP	8.58672E-05	CLI	2.0341E-05	NRS	5.73784E-06
NVQ	8.58672E-05	CVS	2.0341E-05	NWG	5.73784E-06
QNT	8.58672E-05	DHS	2.0341E-05	PDG	5.73784E-06
TDC	8.58672E-05	DSH	2.0341E-05	PFA	5.73784E-06
WCS	8.58672E-05	EGY	2.0341E-05	PMG	5.73784E-06
YDP	8.58672E-05	EKP	2.0341E-05	PMQ	5.73784E-06

YMP	8.58672E-05	EKT	2.0341E-05	PNW	5.73784E-06
YVK	8.58672E-05	ESI	2.0341E-05	PWY	5.73784E-06
CWS	8.54336E-05	FCV	2.0341E-05	PYN	5.73784E-06
DAC	8.54336E-05	FWA	2.0341E-05	QDE	5.73784E-06
DAW	8.54336E-05	FWK	2.0341E-05	QEL	5.73784E-06
DMA	8.54336E-05	GCC	2.0341E-05	QES	5.73784E-06
ERK	8.54336E-05	GKM	2.0341E-05	QGL	5.73784E-06
GWG	8.54336E-05	GMV	2.0341E-05	QKW	5.73784E-06
HCA	8.54336E-05	IAE	2.0341E-05	QYV	5.73784E-06
HWT	8.54336E-05	IGN	2.0341E-05	RFP	5.73784E-06
ICP	8.54336E-05	ISY	2.0341E-05	RKI	5.73784E-06
ILD	8.54336E-05	IWS	2.0341E-05	RKK	5.73784E-06
KAD	8.54336E-05	KSI	2.0341E-05	RRK	5.73784E-06
LCE	8.54336E-05	LDP	2.0341E-05	RVS	5.73784E-06
LMD	8.54336E-05	LFK	2.0341E-05	SDP	5.73784E-06
QKP	8.54336E-05	MIS	2.0341E-05	SEC	5.73784E-06
SCH	8.54336E-05	MMR	2.0341E-05	SNN	5.73784E-06
SDH	8.54336E-05	NET	2.0341E-05	SWK	5.73784E-06
SEN	8.54336E-05	NKH	2.0341E-05	TER	5.73784E-06
SHN	8.54336E-05	NVG	2.0341E-05	TFR	5.73784E-06
SQE	8.54336E-05	PHF	2.0341E-05	TGR	5.73784E-06
WRI	8.54336E-05	PVM	2.0341E-05	TGS	5.73784E-06
YYP	8.54336E-05	QGM	2.0341E-05	TGT	5.73784E-06
AKH	8.49999E-05	QKL	2.0341E-05	TIN	5.73784E-06
CRE	8.49999E-05	RHP	2.0341E-05	TPV	5.73784E-06
CSI	8.49999E-05	RIK	2.0341E-05	TRH	5.73784E-06
DAD	8.49999E-05	RWT	2.0341E-05	TTL	5.73784E-06
EFA	8.49999E-05	SHV	2.0341E-05	VFS	5.73784E-06
HDT	8.49999E-05	STH	2.0341E-05	VGT	5.73784E-06
HGQ	8.49999E-05	SVC	2.0341E-05	VLQ	5.73784E-06
HNV	8.49999E-05	TKV	2.0341E-05	VNN	5.73784E-06
HTH	8.49999E-05	TNM	2.0341E-05	VPV	5.73784E-06
HWP	8.49999E-05	TNP	2.0341E-05	WDE	5.73784E-06
LDY	8.49999E-05	TYR	2.0341E-05	WNI	5.73784E-06
MHL	8.49999E-05	VCT	2.0341E-05	WPW	5.73784E-06
MKT	8.49999E-05	WDD	2.0341E-05	WYW	5.73784E-06
MRH	8.49999E-05	WLY	2.0341E-05	YCF	5.73784E-06
MSC	8.49999E-05	YHR	2.0341E-05	YIL	5.73784E-06
MSK	8.49999E-05	YMI	2.0341E-05	YVW	5.73784E-06
QPH	8.49999E-05	YNQ	2.0341E-05	ACC	5.48837E-06
SID	8.49999E-05	AKR	1.9984E-05	ADF	5.48837E-06
VKW	8.49999E-05	ATK	1.9984E-05	AGA	5.48837E-06
WWS	8.49999E-05	DFS	1.9984E-05	ALE	5.48837E-06
YMV	8.49999E-05	DNS	1.9984E-05	CCG	5.48837E-06

YTQ	8.49999E-05	DNV	1.9984E-05	CDG	5.48837E-06
YVH	8.49999E-05	DQS	1.9984E-05	CNH	5.48837E-06
CEL	8.45662E-05	DRC	1.9984E-05	CQL	5.48837E-06
CRN	8.45662E-05	DWV	1.9984E-05	CVG	5.48837E-06
DTH	8.45662E-05	EIY	1.9984E-05	CVR	5.48837E-06
EAC	8.45662E-05	EMA	1.9984E-05	DAP	5.48837E-06
EPM	8.45662E-05	FAV	1.9984E-05	DHT	5.48837E-06
EYP	8.45662E-05	FMF	1.9984E-05	DQP	5.48837E-06
FDA	8.45662E-05	GEF	1.9984E-05	DTC	5.48837E-06
FTC	8.45662E-05	GNC	1.9984E-05	DYD	5.48837E-06
IMT	8.45662E-05	GNE	1.9984E-05	EPL	5.48837E-06
KPH	8.45662E-05	HDV	1.9984E-05	ESW	5.48837E-06
KSI	8.45662E-05	HHA	1.9984E-05	FAG	5.48837E-06
KTH	8.45662E-05	HIV	1.9984E-05	FMF	5.48837E-06
LHI	8.45662E-05	HLD	1.9984E-05	FVT	5.48837E-06
NKV	8.45662E-05	HPD	1.9984E-05	GHM	5.48837E-06
QPQ	8.45662E-05	ICH	1.9984E-05	GMW	5.48837E-06
REC	8.45662E-05	IVA	1.9984E-05	GYW	5.48837E-06
RIK	8.45662E-05	KEN	1.9984E-05	HCN	5.48837E-06
RIW	8.45662E-05	KFV	1.9984E-05	HFL	5.48837E-06
RKE	8.45662E-05	KHD	1.9984E-05	HHP	5.48837E-06
RYM	8.45662E-05	KPQ	1.9984E-05	HHT	5.48837E-06
SDY	8.45662E-05	KTC	1.9984E-05	HLG	5.48837E-06
SKQ	8.45662E-05	LFD	1.9984E-05	HMS	5.48837E-06
SYD	8.45662E-05	LGY	1.9984E-05	HQL	5.48837E-06
VMW	8.45662E-05	LHN	1.9984E-05	ICP	5.48837E-06
WYA	8.45662E-05	LVD	1.9984E-05	IHA	5.48837E-06
DPH	8.41326E-05	MIR	1.9984E-05	IPG	5.48837E-06
DQP	8.41326E-05	MNM	1.9984E-05	IQL	5.48837E-06
DYA	8.41326E-05	NDV	1.9984E-05	IYA	5.48837E-06
EVM	8.41326E-05	NHS	1.9984E-05	KAW	5.48837E-06
FAC	8.41326E-05	NIS	1.9984E-05	KDP	5.48837E-06
FVD	8.41326E-05	NTI	1.9984E-05	KFD	5.48837E-06
GKC	8.41326E-05	NWP	1.9984E-05	KHP	5.48837E-06
HFT	8.41326E-05	PFF	1.9984E-05	KMW	5.48837E-06
KNV	8.41326E-05	PWK	1.9984E-05	KNW	5.48837E-06
LEH	8.41326E-05	QDS	1.9984E-05	KPT	5.48837E-06
LQI	8.41326E-05	QYH	1.9984E-05	KRV	5.48837E-06
LQN	8.41326E-05	RWI	1.9984E-05	KTF	5.48837E-06
MRN	8.41326E-05	RYM	1.9984E-05	LAK	5.48837E-06
NQT	8.41326E-05	STY	1.9984E-05	LFD	5.48837E-06
QKT	8.41326E-05	SVE	1.9984E-05	LIH	5.48837E-06
RHY	8.41326E-05	SWH	1.9984E-05	LNM	5.48837E-06
RNE	8.41326E-05	TPN	1.9984E-05	LQN	5.48837E-06

RYE	8.41326E-05	WNF	1.9984E-05	LTE	5.48837E-06
SHH	8.41326E-05	WSH	1.9984E-05	LVY	5.48837E-06
SMF	8.41326E-05	WYA	1.9984E-05	MNP	5.48837E-06
SNK	8.41326E-05	YDH	1.9984E-05	MPR	5.48837E-06
WTW	8.41326E-05	YGP	1.9984E-05	NDC	5.48837E-06
YNP	8.41326E-05	YRY	1.9984E-05	NDT	5.48837E-06
YPQ	8.41326E-05	APK	1.9627E-05	NFG	5.48837E-06
CSF	8.36989E-05	AQF	1.9627E-05	NGP	5.48837E-06
DFT	8.36989E-05	ATG	1.9627E-05	NHP	5.48837E-06
DPK	8.36989E-05	ATQ	1.9627E-05	NHV	5.48837E-06
EKP	8.36989E-05	CNA	1.9627E-05	NIG	5.48837E-06
EQA	8.36989E-05	CTI	1.9627E-05	NNL	5.48837E-06
FTH	8.36989E-05	DQA	1.9627E-05	NRD	5.48837E-06
FTK	8.36989E-05	DSW	1.9627E-05	NRQ	5.48837E-06
LFE	8.36989E-05	DTF	1.9627E-05	NSM	5.48837E-06
MQR	8.36989E-05	FET	1.9627E-05	NTA	5.48837E-06
MSQ	8.36989E-05	FPY	1.9627E-05	NVY	5.48837E-06
NEA	8.36989E-05	GEM	1.9627E-05	NWW	5.48837E-06
PQQ	8.36989E-05	GFY	1.9627E-05	PCQ	5.48837E-06
RME	8.36989E-05	GIF	1.9627E-05	PFP	5.48837E-06
SYK	8.36989E-05	GIQ	1.9627E-05	PHC	5.48837E-06
SYN	8.36989E-05	GVP	1.9627E-05	PKM	5.48837E-06
YVQ	8.36989E-05	HPN	1.9627E-05	PRF	5.48837E-06
DVE	8.32652E-05	IHT	1.9627E-05	PTF	5.48837E-06
FMV	8.32652E-05	KDA	1.9627E-05	QCY	5.48837E-06
HPF	8.32652E-05	KVH	1.9627E-05	QEW	5.48837E-06
KMG	8.32652E-05	KYF	1.9627E-05	QLM	5.48837E-06
KPC	8.32652E-05	LCM	1.9627E-05	QLP	5.48837E-06
LIY	8.32652E-05	LFC	1.9627E-05	QPY	5.48837E-06
PCW	8.32652E-05	LKN	1.9627E-05	RAF	5.48837E-06
REF	8.32652E-05	LQI	1.9627E-05	RDT	5.48837E-06
RMD	8.32652E-05	LWE	1.9627E-05	RFV	5.48837E-06
RQN	8.32652E-05	MNW	1.9627E-05	RFY	5.48837E-06
SNE	8.32652E-05	NEL	1.9627E-05	RIF	5.48837E-06
SYE	8.32652E-05	NQS	1.9627E-05	RNQ	5.48837E-06
YGW	8.32652E-05	NSI	1.9627E-05	RPW	5.48837E-06
YKA	8.32652E-05	NVD	1.9627E-05	SGM	5.48837E-06
DAN	8.28315E-05	QAD	1.9627E-05	SKF	5.48837E-06
DDG	8.28315E-05	QTK	1.9627E-05	SKM	5.48837E-06
DET	8.28315E-05	QTV	1.9627E-05	SNV	5.48837E-06
FPK	8.28315E-05	QTY	1.9627E-05	STV	5.48837E-06
FTW	8.28315E-05	SCG	1.9627E-05	SVV	5.48837E-06
FWG	8.28315E-05	TAC	1.9627E-05	SWF	5.48837E-06
HPI	8.28315E-05	TEH	1.9627E-05	TEQ	5.48837E-06

HPY	8.28315E-05	TKD	1.9627E-05	TNH	5.48837E-06
PQC	8.28315E-05	TPF	1.9627E-05	TPG	5.48837E-06
QIA	8.28315E-05	TSH	1.9627E-05	TRA	5.48837E-06
RMI	8.28315E-05	VCP	1.9627E-05	TSF	5.48837E-06
SIN	8.28315E-05	VHQ	1.9627E-05	TSV	5.48837E-06
YAC	8.28315E-05	VSN	1.9627E-05	TYT	5.48837E-06
YAK	8.28315E-05	WYF	1.9627E-05	VET	5.48837E-06
YEP	8.28315E-05	YIT	1.9627E-05	WKM	5.48837E-06
AKM	8.23979E-05	YNS	1.9627E-05	WVP	5.48837E-06
DVY	8.23979E-05	YTH	1.9627E-05	WVY	5.48837E-06
EDT	8.23979E-05	YTQ	1.9627E-05	YFF	5.48837E-06
EET	8.23979E-05	AKI	1.9271E-05	YRN	5.48837E-06
EPQ	8.23979E-05	DPQ	1.9271E-05	YSK	5.48837E-06
EQT	8.23979E-05	DRW	1.9271E-05	YVG	5.48837E-06
FAN	8.23979E-05	EAP	1.9271E-05	YVQ	5.48837E-06
HGC	8.23979E-05	EFP	1.9271E-05	ACN	5.2389E-06
HGM	8.23979E-05	EIR	1.9271E-05	ADR	5.2389E-06
HTE	8.23979E-05	EVR	1.9271E-05	AIL	5.2389E-06
KEP	8.23979E-05	FAH	1.9271E-05	ASG	5.2389E-06
KTD	8.23979E-05	FLY	1.9271E-05	AWW	5.2389E-06
KTE	8.23979E-05	FMY	1.9271E-05	CER	5.2389E-06
MQL	8.23979E-05	FTV	1.9271E-05	CLI	5.2389E-06
NAF	8.23979E-05	FVH	1.9271E-05	CSI	5.2389E-06
NVF	8.23979E-05	GEK	1.9271E-05	DCS	5.2389E-06
QTC	8.23979E-05	GEV	1.9271E-05	DEM	5.2389E-06
RDH	8.23979E-05	GGC	1.9271E-05	DFC	5.2389E-06
RDN	8.23979E-05	GPE	1.9271E-05	DIS	5.2389E-06
REN	8.23979E-05	HCG	1.9271E-05	DKC	5.2389E-06
RQW	8.23979E-05	HRH	1.9271E-05	DPH	5.2389E-06
SIF	8.23979E-05	HSQ	1.9271E-05	EAC	5.2389E-06
SNI	8.23979E-05	IAA	1.9271E-05	EAN	5.2389E-06
TNC	8.23979E-05	IAD	1.9271E-05	EDA	5.2389E-06
VFW	8.23979E-05	IFV	1.9271E-05	EFP	5.2389E-06
WIT	8.23979E-05	KNQ	1.9271E-05	EGL	5.2389E-06
WLY	8.23979E-05	KSK	1.9271E-05	EHD	5.2389E-06
YFA	8.23979E-05	KYG	1.9271E-05	EKG	5.2389E-06
YPH	8.23979E-05	LYW	1.9271E-05	ELI	5.2389E-06
YVN	8.23979E-05	NDS	1.9271E-05	ELV	5.2389E-06
YYA	8.23979E-05	NRI	1.9271E-05	EMS	5.2389E-06
CRI	8.19642E-05	PMN	1.9271E-05	EMT	5.2389E-06
DAK	8.19642E-05	QDN	1.9271E-05	END	5.2389E-06
DCV	8.19642E-05	QGT	1.9271E-05	ENT	5.2389E-06
ECT	8.19642E-05	QGV	1.9271E-05	ERC	5.2389E-06
EQP	8.19642E-05	RKE	1.9271E-05	ERN	5.2389E-06

FFT	8.19642E-05	RQN	1.9271E-05	ERT	5.2389E-06
FTN	8.19642E-05	RVE	1.9271E-05	ETA	5.2389E-06
HVQ	8.19642E-05	SKQ	1.9271E-05	FAD	5.2389E-06
ISY	8.19642E-05	THP	1.9271E-05	FFI	5.2389E-06
KAE	8.19642E-05	THY	1.9271E-05	FHF	5.2389E-06
KHP	8.19642E-05	TVN	1.9271E-05	FLY	5.2389E-06
KVN	8.19642E-05	VCN	1.9271E-05	FMY	5.2389E-06
LII	8.19642E-05	VLY	1.9271E-05	FNY	5.2389E-06
LWD	8.19642E-05	WAP	1.9271E-05	FVH	5.2389E-06
MNT	8.19642E-05	WCL	1.9271E-05	GAV	5.2389E-06
MWR	8.19642E-05	WDW	1.9271E-05	GDT	5.2389E-06
MYR	8.19642E-05	WSD	1.9271E-05	GEM	5.2389E-06
NAD	8.19642E-05	AAG	1.8914E-05	GIK	5.2389E-06
NEH	8.19642E-05	AAN	1.8914E-05	GMV	5.2389E-06
NVI	8.19642E-05	ACK	1.8914E-05	GWC	5.2389E-06
QCA	8.19642E-05	AFQ	1.8914E-05	GWI	5.2389E-06
QMP	8.19642E-05	AVM	1.8914E-05	GYY	5.2389E-06
RED	8.19642E-05	AWS	1.8914E-05	HIS	5.2389E-06
REH	8.19642E-05	CAD	1.8914E-05	HNQ	5.2389E-06
RFQ	8.19642E-05	DGN	1.8914E-05	HWC	5.2389E-06
SHK	8.19642E-05	DGY	1.8914E-05	IGV	5.2389E-06
WSF	8.19642E-05	DQL	1.8914E-05	IIL	5.2389E-06
WVH	8.19642E-05	DRN	1.8914E-05	IIM	5.2389E-06
YAW	8.19642E-05	EKR	1.8914E-05	IMV	5.2389E-06
YDG	8.19642E-05	ESQ	1.8914E-05	IVM	5.2389E-06
YPC	8.19642E-05	FQQ	1.8914E-05	IVQ	5.2389E-06
CSE	8.15305E-05	GCF	1.8914E-05	IWN	5.2389E-06
CSK	8.15305E-05	GCH	1.8914E-05	KDA	5.2389E-06
DGW	8.15305E-05	GDW	1.8914E-05	KDQ	5.2389E-06
DVN	8.15305E-05	GEC	1.8914E-05	KEG	5.2389E-06
FAW	8.15305E-05	GVM	1.8914E-05	LFK	5.2389E-06
FCV	8.15305E-05	HES	1.8914E-05	LKD	5.2389E-06
FGQ	8.15305E-05	HFF	1.8914E-05	LNK	5.2389E-06
FPH	8.15305E-05	HGA	1.8914E-05	MCT	5.2389E-06
GCM	8.15305E-05	HMA	1.8914E-05	MGH	5.2389E-06
HAH	8.15305E-05	HNG	1.8914E-05	MLA	5.2389E-06
HVE	8.15305E-05	HSW	1.8914E-05	MWW	5.2389E-06
ICT	8.15305E-05	ICG	1.8914E-05	NNC	5.2389E-06
IDA	8.15305E-05	IDA	1.8914E-05	NWS	5.2389E-06
ISI	8.15305E-05	KCR	1.8914E-05	PDL	5.2389E-06
IVM	8.15305E-05	KDM	1.8914E-05	PHW	5.2389E-06
IVW	8.15305E-05	LDC	1.8914E-05	PTQ	5.2389E-06
KPN	8.15305E-05	LFV	1.8914E-05	PWA	5.2389E-06
MKS	8.15305E-05	MRC	1.8914E-05	PWI	5.2389E-06

NAE	8.15305E-05	MYP	1.8914E-05	QAC	5.2389E-06
NTY	8.15305E-05	NCA	1.8914E-05	QCT	5.2389E-06
RFE	8.15305E-05	NFA	1.8914E-05	QGW	5.2389E-06
RYN	8.15305E-05	NPG	1.8914E-05	QNE	5.2389E-06
SII	8.15305E-05	NTF	1.8914E-05	QTW	5.2389E-06
SQK	8.15305E-05	PFY	1.8914E-05	RDG	5.2389E-06
SWH	8.15305E-05	PMC	1.8914E-05	REG	5.2389E-06
VNQ	8.15305E-05	PNM	1.8914E-05	RIP	5.2389E-06
WNP	8.15305E-05	QIR	1.8914E-05	RVP	5.2389E-06
YTH	8.15305E-05	RNM	1.8914E-05	SYD	5.2389E-06
CTM	8.10968E-05	SKF	1.8914E-05	TDL	5.2389E-06
ETC	8.10968E-05	SMA	1.8914E-05	THR	5.2389E-06
HFV	8.10968E-05	STI	1.8914E-05	TKL	5.2389E-06
INP	8.10968E-05	TML	1.8914E-05	TKP	5.2389E-06
KFP	8.10968E-05	TSY	1.8914E-05	TLY	5.2389E-06
KGM	8.10968E-05	TWE	1.8914E-05	VDQ	5.2389E-06
MLY	8.10968E-05	VCG	1.8914E-05	VDT	5.2389E-06
MPW	8.10968E-05	VIA	1.8914E-05	VMH	5.2389E-06
MRQ	8.10968E-05	VPV	1.8914E-05	VYW	5.2389E-06
NHG	8.10968E-05	VTH	1.8914E-05	WHK	5.2389E-06
QSI	8.10968E-05	VYA	1.8914E-05	WTK	5.2389E-06
QTN	8.10968E-05	WGY	1.8914E-05	YDP	5.2389E-06
QVW	8.10968E-05	WIC	1.8914E-05	YGT	5.2389E-06
SEY	8.10968E-05	WNV	1.8914E-05	YKT	5.2389E-06
SHE	8.10968E-05	YER	1.8914E-05	YPM	5.2389E-06
SMY	8.10968E-05	YFM	1.8914E-05	YVI	5.2389E-06
SQD	8.10968E-05	YHF	1.8914E-05	YWA	5.2389E-06
SWK	8.10968E-05	YHV	1.8914E-05	ADL	4.98942E-06
SYQ	8.10968E-05	YPY	1.8914E-05	ADQ	4.98942E-06
VNW	8.10968E-05	YYF	1.8914E-05	AFI	4.98942E-06
WVD	8.10968E-05	AHL	1.8557E-05	AGT	4.98942E-06
YEV	8.10968E-05	AKN	1.8557E-05	ASI	4.98942E-06
CDV	8.06632E-05	AQH	1.8557E-05	AWA	4.98942E-06
CSY	8.06632E-05	AVI	1.8557E-05	CAH	4.98942E-06
FVQ	8.06632E-05	CAR	1.8557E-05	CCS	4.98942E-06
HAN	8.06632E-05	CCL	1.8557E-05	CHS	4.98942E-06
HMG	8.06632E-05	CPP	1.8557E-05	CIR	4.98942E-06
KCG	8.06632E-05	DER	1.8557E-05	DAK	4.98942E-06
KIV	8.06632E-05	EET	1.8557E-05	DDA	4.98942E-06
QQP	8.06632E-05	EFY	1.8557E-05	DEA	4.98942E-06
QRY	8.06632E-05	ERI	1.8557E-05	DPW	4.98942E-06
RQE	8.06632E-05	EYH	1.8557E-05	DRM	4.98942E-06
RWN	8.06632E-05	FDP	1.8557E-05	EAL	4.98942E-06
SCN	8.06632E-05	FHF	1.8557E-05	EEV	4.98942E-06

SFI	8.06632E-05	FLK	1.8557E-05	EGR	4.98942E-06
SHI	8.06632E-05	FMT	1.8557E-05	EGW	4.98942E-06
SKH	8.06632E-05	FNN	1.8557E-05	ENI	4.98942E-06
VMM	8.06632E-05	FTK	1.8557E-05	ENQ	4.98942E-06
WNA	8.06632E-05	FTM	1.8557E-05	EQT	4.98942E-06
WTC	8.06632E-05	FWI	1.8557E-05	FAA	4.98942E-06
CMP	8.02295E-05	GKQ	1.8557E-05	FCY	4.98942E-06
DKG	8.02295E-05	GNG	1.8557E-05	FDC	4.98942E-06
ETD	8.02295E-05	HLM	1.8557E-05	FGD	4.98942E-06
FNA	8.02295E-05	HWI	1.8557E-05	FMI	4.98942E-06
FVE	8.02295E-05	IIP	1.8557E-05	GAG	4.98942E-06
GDH	8.02295E-05	ISF	1.8557E-05	GFP	4.98942E-06
IRE	8.02295E-05	KAY	1.8557E-05	GIA	4.98942E-06
KCP	8.02295E-05	KFG	1.8557E-05	GMN	4.98942E-06
KDG	8.02295E-05	KWD	1.8557E-05	GRW	4.98942E-06
KFA	8.02295E-05	LVE	1.8557E-05	GVY	4.98942E-06
KNG	8.02295E-05	NIY	1.8557E-05	HAQ	4.98942E-06
MMT	8.02295E-05	NTG	1.8557E-05	HGH	4.98942E-06
MSH	8.02295E-05	PMA	1.8557E-05	HQG	4.98942E-06
QIP	8.02295E-05	QLF	1.8557E-05	HTT	4.98942E-06
REE	8.02295E-05	QNY	1.8557E-05	IFC	4.98942E-06
RYW	8.02295E-05	QTG	1.8557E-05	IGM	4.98942E-06
SCK	8.02295E-05	RFY	1.8557E-05	ING	4.98942E-06
SCY	8.02295E-05	SLE	1.8557E-05	IPP	4.98942E-06
SFK	8.02295E-05	TVP	1.8557E-05	KDD	4.98942E-06
SKD	8.02295E-05	VRI	1.8557E-05	KFL	4.98942E-06
SMI	8.02295E-05	WDT	1.8557E-05	KML	4.98942E-06
SNF	8.02295E-05	WKW	1.8557E-05	KVD	4.98942E-06
WVC	8.02295E-05	WPH	1.8557E-05	KVN	4.98942E-06
YAD	8.02295E-05	YFI	1.8557E-05	LDY	4.98942E-06
CNP	7.97958E-05	AWE	1.82E-05	LMD	4.98942E-06
CQS	7.97958E-05	CSE	1.82E-05	MFL	4.98942E-06
CVE	7.97958E-05	DAF	1.82E-05	MKW	4.98942E-06
DHG	7.97958E-05	DAM	1.82E-05	MMN	4.98942E-06
DYV	7.97958E-05	DNP	1.82E-05	MRC	4.98942E-06
EDA	7.97958E-05	DPN	1.82E-05	MRH	4.98942E-06
EKV	7.97958E-05	EQN	1.82E-05	NEE	4.98942E-06
ETW	7.97958E-05	FAN	1.82E-05	NEF	4.98942E-06
FDT	7.97958E-05	FCW	1.82E-05	NHD	4.98942E-06
HDG	7.97958E-05	FEA	1.82E-05	NML	4.98942E-06
HEP	7.97958E-05	FFP	1.82E-05	NMQ	4.98942E-06
IRD	7.97958E-05	FHD	1.82E-05	NQS	4.98942E-06
IRF	7.97958E-05	FHT	1.82E-05	NTK	4.98942E-06
IRY	7.97958E-05	FIG	1.82E-05	NVG	4.98942E-06

IYT	7.97958E-05	GMI	1.82E-05	NVP	4.98942E-06
KHV	7.97958E-05	GMM	1.82E-05	PIW	4.98942E-06
KMP	7.97958E-05	GWN	1.82E-05	PKW	4.98942E-06
KQP	7.97958E-05	HFP	1.82E-05	PVN	4.98942E-06
KVE	7.97958E-05	HGG	1.82E-05	QIH	4.98942E-06
LHY	7.97958E-05	IGQ	1.82E-05	QKT	4.98942E-06
LQD	7.97958E-05	IKH	1.82E-05	QLL	4.98942E-06
LYD	7.97958E-05	IQE	1.82E-05	QSF	4.98942E-06
MRD	7.97958E-05	KCT	1.82E-05	QYW	4.98942E-06
QDA	7.97958E-05	KDC	1.82E-05	RAW	4.98942E-06
QPM	7.97958E-05	KMP	1.82E-05	RID	4.98942E-06
RHF	7.97958E-05	KTI	1.82E-05	RIN	4.98942E-06
VDC	7.97958E-05	LCI	1.82E-05	RYK	4.98942E-06
WDT	7.97958E-05	LMD	1.82E-05	SLE	4.98942E-06
WKC	7.97958E-05	MPF	1.82E-05	TEN	4.98942E-06
YMA	7.97958E-05	NCR	1.82E-05	TML	4.98942E-06
YTK	7.97958E-05	NGE	1.82E-05	TMN	4.98942E-06
ANQ	7.93622E-05	NIR	1.82E-05	TRG	4.98942E-06
CNT	7.93622E-05	PFC	1.82E-05	TTA	4.98942E-06
DTF	7.93622E-05	PMW	1.82E-05	TWR	4.98942E-06
FFV	7.93622E-05	RKG	1.82E-05	VCH	4.98942E-06
FNV	7.93622E-05	SFV	1.82E-05	VFT	4.98942E-06
FQP	7.93622E-05	TAY	1.82E-05	VMI	4.98942E-06
FTD	7.93622E-05	TFD	1.82E-05	WCD	4.98942E-06
FVN	7.93622E-05	VAG	1.82E-05	WCS	4.98942E-06
HPK	7.93622E-05	VCH	1.82E-05	WEK	4.98942E-06
HTI	7.93622E-05	VKA	1.82E-05	YGS	4.98942E-06
HTN	7.93622E-05	VKH	1.82E-05	YIC	4.98942E-06
LFY	7.93622E-05	VSG	1.82E-05	YIS	4.98942E-06
LQK	7.93622E-05	VTD	1.82E-05	YNE	4.98942E-06
NNG	7.93622E-05	WDA	1.82E-05	YPN	4.98942E-06
RYD	7.93622E-05	WFG	1.82E-05	YQC	4.98942E-06
VCW	7.93622E-05	WRW	1.82E-05	ACA	4.73995E-06
VDQ	7.93622E-05	WTV	1.82E-05	AEN	4.73995E-06
VKM	7.93622E-05	YGG	1.82E-05	AET	4.73995E-06
VMN	7.93622E-05	YKA	1.82E-05	AFP	4.73995E-06
VNK	7.93622E-05	YLH	1.82E-05	AHV	4.73995E-06
WSN	7.93622E-05	YRH	1.82E-05	AVH	4.73995E-06
YKV	7.93622E-05	AAF	1.7843E-05	CAC	4.73995E-06
YPD	7.93622E-05	AAQ	1.7843E-05	CCP	4.73995E-06
YVF	7.93622E-05	AVE	1.7843E-05	CFS	4.73995E-06
AHQ	7.89285E-05	AWF	1.7843E-05	CFW	4.73995E-06
AIC	7.89285E-05	CIQ	1.7843E-05	CKH	4.73995E-06
CFT	7.89285E-05	CPW	1.7843E-05	CNA	4.73995E-06

DVK	7.89285E-05	DKP	1.7843E-05	CNR	4.73995E-06
EAW	7.89285E-05	EEA	1.7843E-05	CPC	4.73995E-06
ECA	7.89285E-05	EGE	1.7843E-05	CSK	4.73995E-06
FAM	7.89285E-05	EVN	1.7843E-05	CTW	4.73995E-06
FIA	7.89285E-05	FGK	1.7843E-05	DEV	4.73995E-06
FQT	7.89285E-05	FHH	1.7843E-05	DFS	4.73995E-06
HCG	7.89285E-05	FKV	1.7843E-05	DHL	4.73995E-06
HEA	7.89285E-05	GHT	1.7843E-05	DNC	4.73995E-06
HTD	7.89285E-05	GKD	1.7843E-05	DPD	4.73995E-06
HVF	7.89285E-05	GLE	1.7843E-05	DSE	4.73995E-06
KAF	7.89285E-05	HDQ	1.7843E-05	DSN	4.73995E-06
KKG	7.89285E-05	IRF	1.7843E-05	EHE	4.73995E-06
LWF	7.89285E-05	IRM	1.7843E-05	ENM	4.73995E-06
MRF	7.89285E-05	ITM	1.7843E-05	ENW	4.73995E-06
NGQ	7.89285E-05	KDN	1.7843E-05	ETE	4.73995E-06
NQA	7.89285E-05	KGY	1.7843E-05	FET	4.73995E-06
NVY	7.89285E-05	KMA	1.7843E-05	FKK	4.73995E-06
QYP	7.89285E-05	KNM	1.7843E-05	FQE	4.73995E-06
RQQ	7.89285E-05	KVP	1.7843E-05	GAK	4.73995E-06
VWW	7.89285E-05	KYR	1.7843E-05	GDN	4.73995E-06
YGM	7.89285E-05	LMF	1.7843E-05	GQE	4.73995E-06
YPN	7.89285E-05	MAG	1.7843E-05	GSE	4.73995E-06
AMM	7.84948E-05	MAH	1.7843E-05	GVF	4.73995E-06
CMT	7.84948E-05	MPQ	1.7843E-05	HAC	4.73995E-06
CRH	7.84948E-05	NEQ	1.7843E-05	HFR	4.73995E-06
DVH	7.84948E-05	NMK	1.7843E-05	HPY	4.73995E-06
EIT	7.84948E-05	NMS	1.7843E-05	HSF	4.73995E-06
FKV	7.84948E-05	NRC	1.7843E-05	HSY	4.73995E-06
GMM	7.84948E-05	NTY	1.7843E-05	IAH	4.73995E-06
GNH	7.84948E-05	QGI	1.7843E-05	ICQ	4.73995E-06
GYM	7.84948E-05	QKT	1.7843E-05	IGP	4.73995E-06
HGH	7.84948E-05	QVL	1.7843E-05	IHH	4.73995E-06
HVH	7.84948E-05	QWH	1.7843E-05	IIK	4.73995E-06
IDP	7.84948E-05	SCQ	1.7843E-05	INF	4.73995E-06
KAH	7.84948E-05	SNC	1.7843E-05	IPV	4.73995E-06
MES	7.84948E-05	TCT	1.7843E-05	ITI	4.73995E-06
MRE	7.84948E-05	TGM	1.7843E-05	IWF	4.73995E-06
QEP	7.84948E-05	TKH	1.7843E-05	KAL	4.73995E-06
QHP	7.84948E-05	TWH	1.7843E-05	KAN	4.73995E-06
QMA	7.84948E-05	VEA	1.7843E-05	KHE	4.73995E-06
RIF	7.84948E-05	VER	1.7843E-05	KKW	4.73995E-06
RWC	7.84948E-05	VIG	1.7843E-05	KLC	4.73995E-06
RYI	7.84948E-05	VKM	1.7843E-05	KPP	4.73995E-06
SHY	7.84948E-05	VPM	1.7843E-05	KQR	4.73995E-06

THM	7.84948E-05	VVL	1.7843E-05	KRF	4.73995E-06
VKQ	7.84948E-05	WIA	1.7843E-05	KRY	4.73995E-06
WPW	7.84948E-05	WMT	1.7843E-05	KVQ	4.73995E-06
WSY	7.84948E-05	YNW	1.7843E-05	KWA	4.73995E-06
WTQ	7.84948E-05	YPM	1.7843E-05	KYD	4.73995E-06
YQP	7.84948E-05	YWS	1.7843E-05	LEG	4.73995E-06
CKT	7.80611E-05	CGR	1.7486E-05	LIN	4.73995E-06
CRY	7.80611E-05	CVP	1.7486E-05	LVA	4.73995E-06
CSD	7.80611E-05	DAV	1.7486E-05	LVH	4.73995E-06
DAH	7.80611E-05	DFT	1.7486E-05	MCS	4.73995E-06
DFA	7.80611E-05	DKA	1.7486E-05	MCW	4.73995E-06
DGE	7.80611E-05	DWH	1.7486E-05	MEH	4.73995E-06
DGM	7.80611E-05	DWS	1.7486E-05	MRW	4.73995E-06
DPQ	7.80611E-05	DYL	1.7486E-05	NCL	4.73995E-06
EDP	7.80611E-05	EIL	1.7486E-05	NFK	4.73995E-06
EPK	7.80611E-05	ELI	1.7486E-05	NKA	4.73995E-06
FGI	7.80611E-05	EQG	1.7486E-05	NKD	4.73995E-06
FIP	7.80611E-05	EVQ	1.7486E-05	NNF	4.73995E-06
FPE	7.80611E-05	FAQ	1.7486E-05	NPH	4.73995E-06
HIV	7.80611E-05	FCM	1.7486E-05	NQD	4.73995E-06
HYV	7.80611E-05	FHG	1.7486E-05	NRP	4.73995E-06
IDT	7.80611E-05	GDI	1.7486E-05	NYD	4.73995E-06
IMP	7.80611E-05	GEE	1.7486E-05	PCP	4.73995E-06
IPQ	7.80611E-05	GLK	1.7486E-05	PES	4.73995E-06
KGD	7.80611E-05	HAG	1.7486E-05	PIE	4.73995E-06
KHG	7.80611E-05	HDW	1.7486E-05	PIK	4.73995E-06
NEP	7.80611E-05	HNS	1.7486E-05	PMW	4.73995E-06
NWA	7.80611E-05	HRT	1.7486E-05	QAS	4.73995E-06
PHC	7.80611E-05	IDD	1.7486E-05	QCP	4.73995E-06
QDP	7.80611E-05	IMK	1.7486E-05	QEA	4.73995E-06
RCK	7.80611E-05	KAW	1.7486E-05	QKY	4.73995E-06
REI	7.80611E-05	KVV	1.7486E-05	QLV	4.73995E-06
SEE	7.80611E-05	LDI	1.7486E-05	QQV	4.73995E-06
SYF	7.80611E-05	LEW	1.7486E-05	QSE	4.73995E-06
VME	7.80611E-05	MCA	1.7486E-05	QWT	4.73995E-06
VYM	7.80611E-05	MQR	1.7486E-05	RAE	4.73995E-06
DTI	7.76275E-05	MTV	1.7486E-05	RFI	4.73995E-06
EKA	7.76275E-05	MYA	1.7486E-05	RHE	4.73995E-06
EPW	7.76275E-05	NLF	1.7486E-05	RVN	4.73995E-06
FET	7.76275E-05	NLY	1.7486E-05	RWE	4.73995E-06
FFP	7.76275E-05	NPQ	1.7486E-05	SAA	4.73995E-06
HAK	7.76275E-05	NVA	1.7486E-05	SAG	4.73995E-06
HPN	7.76275E-05	NWV	1.7486E-05	SAM	4.73995E-06
HQT	7.76275E-05	NYT	1.7486E-05	SEN	4.73995E-06

HVI	7.76275E-05	PAY	1.7486E-05	SHY	4.73995E-06
KAN	7.76275E-05	PKG	1.7486E-05	SKI	4.73995E-06
KPD	7.76275E-05	PKN	1.7486E-05	SMF	4.73995E-06
KPW	7.76275E-05	PNE	1.7486E-05	SMP	4.73995E-06
KTK	7.76275E-05	QCQ	1.7486E-05	SMV	4.73995E-06
KVH	7.76275E-05	QDG	1.7486E-05	SQC	4.73995E-06
KVK	7.76275E-05	QNM	1.7486E-05	SQD	4.73995E-06
LED	7.76275E-05	QRN	1.7486E-05	SVC	4.73995E-06
LFI	7.76275E-05	QTN	1.7486E-05	SYM	4.73995E-06
MLF	7.76275E-05	RFV	1.7486E-05	TFS	4.73995E-06
MSE	7.76275E-05	RQK	1.7486E-05	TGN	4.73995E-06
RFK	7.76275E-05	RVY	1.7486E-05	TGP	4.73995E-06
RIE	7.76275E-05	SFM	1.7486E-05	TLC	4.73995E-06
RNF	7.76275E-05	SIF	1.7486E-05	TMW	4.73995E-06
RWD	7.76275E-05	TCD	1.7486E-05	TMY	4.73995E-06
VCM	7.76275E-05	TSQ	1.7486E-05	TNP	4.73995E-06
WCT	7.76275E-05	VTN	1.7486E-05	TRV	4.73995E-06
WPC	7.76275E-05	VVS	1.7486E-05	TWM	4.73995E-06
WQP	7.76275E-05	VYQ	1.7486E-05	TYS	4.73995E-06
YNV	7.76275E-05	WAT	1.7486E-05	VGG	4.73995E-06
YTD	7.76275E-05	WRC	1.7486E-05	VHF	4.73995E-06
YTY	7.76275E-05	WRF	1.7486E-05	VHM	4.73995E-06
ACC	7.71938E-05	WTI	1.7486E-05	VHQ	4.73995E-06
DWA	7.71938E-05	YCC	1.7486E-05	VPE	4.73995E-06
EVK	7.71938E-05	AFG	1.7129E-05	VSK	4.73995E-06
FAQ	7.71938E-05	CGA	1.7129E-05	VVV	4.73995E-06
GKW	7.71938E-05	CYT	1.7129E-05	VYC	4.73995E-06
GQH	7.71938E-05	DCG	1.7129E-05	WCF	4.73995E-06
HTF	7.71938E-05	DLN	1.7129E-05	WMV	4.73995E-06
ITC	7.71938E-05	DQR	1.7129E-05	WMY	4.73995E-06
KAQ	7.71938E-05	DSQ	1.7129E-05	WTV	4.73995E-06
NAN	7.71938E-05	DWK	1.7129E-05	YCQ	4.73995E-06
NGE	7.71938E-05	EPQ	1.7129E-05	YDD	4.73995E-06
PNW	7.71938E-05	ETI	1.7129E-05	YFD	4.73995E-06
QFT	7.71938E-05	EYP	1.7129E-05	YFH	4.73995E-06
QVK	7.71938E-05	FFF	1.7129E-05	YFP	4.73995E-06
QYT	7.71938E-05	FKA	1.7129E-05	YRI	4.73995E-06
SDI	7.71938E-05	FSE	1.7129E-05	YTE	4.73995E-06
SED	7.71938E-05	FTI	1.7129E-05	YTV	4.73995E-06
SEH	7.71938E-05	FTN	1.7129E-05	YVC	4.73995E-06
SKY	7.71938E-05	FVN	1.7129E-05	YVT	4.73995E-06
TDM	7.71938E-05	FYF	1.7129E-05	ACW	4.49048E-06
VNE	7.71938E-05	GCN	1.7129E-05	AED	4.49048E-06
YYG	7.71938E-05	GDV	1.7129E-05	AGV	4.49048E-06

CTN	7.67601E-05	GFP	1.7129E-05	AIR	4.49048E-06
CYT	7.67601E-05	GFV	1.7129E-05	AKW	4.49048E-06
DAE	7.67601E-05	GQH	1.7129E-05	AMQ	4.49048E-06
DAF	7.67601E-05	GWK	1.7129E-05	AQF	4.49048E-06
DDV	7.67601E-05	HGF	1.7129E-05	ARE	4.49048E-06
DPE	7.67601E-05	HRC	1.7129E-05	ASK	4.49048E-06
EAH	7.67601E-05	HWR	1.7129E-05	ATG	4.49048E-06
EPC	7.67601E-05	ISD	1.7129E-05	ATV	4.49048E-06
EWT	7.67601E-05	IVE	1.7129E-05	CKY	4.49048E-06
FEV	7.67601E-05	KCC	1.7129E-05	CLD	4.49048E-06
GCC	7.67601E-05	KTQ	1.7129E-05	CQR	4.49048E-06
GIC	7.67601E-05	LCF	1.7129E-05	CRM	4.49048E-06
GKQ	7.67601E-05	LDH	1.7129E-05	CWF	4.49048E-06
GNC	7.67601E-05	LDV	1.7129E-05	DAS	4.49048E-06
IIT	7.67601E-05	LFY	1.7129E-05	DDS	4.49048E-06
KEG	7.67601E-05	LHY	1.7129E-05	DFL	4.49048E-06
KKV	7.67601E-05	LIW	1.7129E-05	DLF	4.49048E-06
KPK	7.67601E-05	LQG	1.7129E-05	DLN	4.49048E-06
KYA	7.67601E-05	MFC	1.7129E-05	DMQ	4.49048E-06
LWY	7.67601E-05	MIV	1.7129E-05	DSV	4.49048E-06
LYY	7.67601E-05	MMS	1.7129E-05	EAP	4.49048E-06
NTE	7.67601E-05	MRI	1.7129E-05	EAR	4.49048E-06
QNG	7.67601E-05	MRW	1.7129E-05	ECP	4.49048E-06
QTH	7.67601E-05	NSK	1.7129E-05	EDY	4.49048E-06
QTW	7.67601E-05	PDH	1.7129E-05	EEC	4.49048E-06
QVM	7.67601E-05	PEY	1.7129E-05	EIA	4.49048E-06
REK	7.67601E-05	PTM	1.7129E-05	EPG	4.49048E-06
RKY	7.67601E-05	PYH	1.7129E-05	EQD	4.49048E-06
SQC	7.67601E-05	QLI	1.7129E-05	EQR	4.49048E-06
TNW	7.67601E-05	RAI	1.7129E-05	ESI	4.49048E-06
VYQ	7.67601E-05	REG	1.7129E-05	ETQ	4.49048E-06
WFP	7.67601E-05	RVF	1.7129E-05	EVR	4.49048E-06
WRY	7.67601E-05	RWD	1.7129E-05	FAK	4.49048E-06
WSD	7.67601E-05	SFQ	1.7129E-05	FDV	4.49048E-06
YHV	7.67601E-05	TDQ	1.7129E-05	FTE	4.49048E-06
YTF	7.67601E-05	TKR	1.7129E-05	FVA	4.49048E-06
ANH	7.63264E-05	TMH	1.7129E-05	FYN	4.49048E-06
CTW	7.63264E-05	VTG	1.7129E-05	GAE	4.49048E-06
FCA	7.63264E-05	WAC	1.7129E-05	GFY	4.49048E-06
FGH	7.63264E-05	WDQ	1.7129E-05	HAR	4.49048E-06
FYA	7.63264E-05	WKA	1.7129E-05	HDG	4.49048E-06
GNQ	7.63264E-05	WLK	1.7129E-05	HDW	4.49048E-06
HAC	7.63264E-05	WSK	1.7129E-05	HPT	4.49048E-06
KCA	7.63264E-05	WTW	1.7129E-05	HQT	4.49048E-06

KDV	7.63264E-05	YAG	1.7129E-05	HRH	4.49048E-06
KGN	7.63264E-05	YAH	1.7129E-05	HSV	4.49048E-06
KPF	7.63264E-05	YAN	1.7129E-05	HYQ	4.49048E-06
KTN	7.63264E-05	YSE	1.7129E-05	IFG	4.49048E-06
KVD	7.63264E-05	YYW	1.7129E-05	IGC	4.49048E-06
MRY	7.63264E-05	ATV	1.6772E-05	IHP	4.49048E-06
NGK	7.63264E-05	CFQ	1.6772E-05	ILY	4.49048E-06
NPF	7.63264E-05	CRP	1.6772E-05	IMH	4.49048E-06
QAW	7.63264E-05	CTA	1.6772E-05	IPW	4.49048E-06
RID	7.63264E-05	EKI	1.6772E-05	IRH	4.49048E-06
RWF	7.63264E-05	ESY	1.6772E-05	KCN	4.49048E-06
RYK	7.63264E-05	ETC	1.6772E-05	KGL	4.49048E-06
SQN	7.63264E-05	FAW	1.6772E-05	KHD	4.49048E-06
SQY	7.63264E-05	FDQ	1.6772E-05	KSQ	4.49048E-06
TWM	7.63264E-05	FDY	1.6772E-05	KSV	4.49048E-06
VKH	7.63264E-05	GDN	1.6772E-05	KWR	4.49048E-06
WDA	7.63264E-05	HEQ	1.6772E-05	LDF	4.49048E-06
YWT	7.63264E-05	HMV	1.6772E-05	LEY	4.49048E-06
AMW	7.58928E-05	ICR	1.6772E-05	LIQ	4.49048E-06
ANK	7.58928E-05	IFS	1.6772E-05	LVT	4.49048E-06
ANW	7.58928E-05	IRW	1.6772E-05	MCR	4.49048E-06
DHV	7.58928E-05	KKK	1.6772E-05	MIW	4.49048E-06
DPI	7.58928E-05	KQA	1.6772E-05	MKH	4.49048E-06
EPI	7.58928E-05	KQR	1.6772E-05	MLI	4.49048E-06
EVC	7.58928E-05	LID	1.6772E-05	MML	4.49048E-06
EVH	7.58928E-05	MHL	1.6772E-05	MNA	4.49048E-06
FAK	7.58928E-05	NCP	1.6772E-05	MRA	4.49048E-06
FEA	7.58928E-05	NKG	1.6772E-05	MSD	4.49048E-06
FFA	7.58928E-05	PHC	1.6772E-05	MTH	4.49048E-06
FHG	7.58928E-05	PQH	1.6772E-05	NYL	4.49048E-06
FPF	7.58928E-05	QCL	1.6772E-05	PDC	4.49048E-06
FPN	7.58928E-05	QDA	1.6772E-05	PDY	4.49048E-06
FTQ	7.58928E-05	QDP	1.6772E-05	PEC	4.49048E-06
GMC	7.58928E-05	QGD	1.6772E-05	PGT	4.49048E-06
HIG	7.58928E-05	QHL	1.6772E-05	PHM	4.49048E-06
KAK	7.58928E-05	RFI	1.6772E-05	PHQ	4.49048E-06
KAM	7.58928E-05	RMG	1.6772E-05	PMA	4.49048E-06
LEI	7.58928E-05	RQG	1.6772E-05	PQQ	4.49048E-06
MSN	7.58928E-05	RYD	1.6772E-05	PTV	4.49048E-06
NFV	7.58928E-05	SEV	1.6772E-05	QAQ	4.49048E-06
NPE	7.58928E-05	SYW	1.6772E-05	QEP	4.49048E-06
NYV	7.58928E-05	TCP	1.6772E-05	QIP	4.49048E-06
RQF	7.58928E-05	TFK	1.6772E-05	QKQ	4.49048E-06
VDH	7.58928E-05	TQV	1.6772E-05	QLD	4.49048E-06

VFM	7.58928E-05	TTK	1.6772E-05	QLY	4.49048E-06
VIM	7.58928E-05	TVE	1.6772E-05	QTF	4.49048E-06
WSI	7.58928E-05	TWD	1.6772E-05	RDN	4.49048E-06
YDV	7.58928E-05	VDS	1.6772E-05	REK	4.49048E-06
YTE	7.58928E-05	VWK	1.6772E-05	RIL	4.49048E-06
YVE	7.58928E-05	WKQ	1.6772E-05	RIQ	4.49048E-06
AMQ	7.54591E-05	WND	1.6772E-05	RNE	4.49048E-06
ENV	7.54591E-05	WPG	1.6772E-05	RQK	4.49048E-06
FMA	7.54591E-05	WRD	1.6772E-05	SFF	4.49048E-06
FVK	7.54591E-05	WVE	1.6772E-05	SND	4.49048E-06
GEQ	7.54591E-05	YKT	1.6772E-05	SYE	4.49048E-06
GIM	7.54591E-05	YRD	1.6772E-05	TAP	4.49048E-06
HKV	7.54591E-05	YSI	1.6772E-05	TDS	4.49048E-06
IHP	7.54591E-05	YTV	1.6772E-05	THG	4.49048E-06
ITM	7.54591E-05	YTW	1.6772E-05	TMH	4.49048E-06
KCV	7.54591E-05	AIL	1.6416E-05	TQP	4.49048E-06
KGC	7.54591E-05	CQL	1.6416E-05	TQT	4.49048E-06
KVY	7.54591E-05	CRQ	1.6416E-05	TRQ	4.49048E-06
LCY	7.54591E-05	CYA	1.6416E-05	TVM	4.49048E-06
NGH	7.54591E-05	DDW	1.6416E-05	TWP	4.49048E-06
PDC	7.54591E-05	DGF	1.6416E-05	VKK	4.49048E-06
PHW	7.54591E-05	DGK	1.6416E-05	VSE	4.49048E-06
PQW	7.54591E-05	DMP	1.6416E-05	VTI	4.49048E-06
QHT	7.54591E-05	DTV	1.6416E-05	VVS	4.49048E-06
QPN	7.54591E-05	EQA	1.6416E-05	VVT	4.49048E-06
RFH	7.54591E-05	ESE	1.6416E-05	WCE	4.49048E-06
RQD	7.54591E-05	ETF	1.6416E-05	WCP	4.49048E-06
RQI	7.54591E-05	FKM	1.6416E-05	WET	4.49048E-06
SEF	7.54591E-05	FPH	1.6416E-05	WKI	4.49048E-06
SWD	7.54591E-05	FYM	1.6416E-05	WMA	4.49048E-06
SYI	7.54591E-05	FYN	1.6416E-05	WQV	4.49048E-06
TIW	7.54591E-05	GFH	1.6416E-05	WVN	4.49048E-06
WAK	7.54591E-05	GHQ	1.6416E-05	YKA	4.49048E-06
WGE	7.54591E-05	HYS	1.6416E-05	YKD	4.49048E-06
WVM	7.54591E-05	ICV	1.6416E-05	YKV	4.49048E-06
YAI	7.54591E-05	IFG	1.6416E-05	YLY	4.49048E-06
AHM	7.50254E-05	IIL	1.6416E-05	YMC	4.49048E-06
CVD	7.50254E-05	KAQ	1.6416E-05	YPE	4.49048E-06
DFG	7.50254E-05	KHG	1.6416E-05	AAD	4.24101E-06
EYA	7.50254E-05	KHY	1.6416E-05	ADW	4.24101E-06
FAF	7.50254E-05	KIR	1.6416E-05	AEI	4.24101E-06
FMP	7.50254E-05	KNC	1.6416E-05	AIV	4.24101E-06
GNM	7.50254E-05	KPI	1.6416E-05	ARG	4.24101E-06
HGD	7.50254E-05	LMK	1.6416E-05	AVT	4.24101E-06

HHV	7.50254E-05	LMV	1.6416E-05	CFV	4.24101E-06
IGM	7.50254E-05	MND	1.6416E-05	CGG	4.24101E-06
INA	7.50254E-05	MPH	1.6416E-05	CHD	4.24101E-06
PNC	7.50254E-05	MPK	1.6416E-05	CRF	4.24101E-06
QAM	7.50254E-05	MRD	1.6416E-05	CSM	4.24101E-06
RCI	7.50254E-05	MWV	1.6416E-05	CTP	4.24101E-06
RWH	7.50254E-05	NDR	1.6416E-05	CYW	4.24101E-06
SFE	7.50254E-05	NDW	1.6416E-05	DEC	4.24101E-06
SKF	7.50254E-05	NEA	1.6416E-05	DGP	4.24101E-06
YAN	7.50254E-05	NWN	1.6416E-05	DKM	4.24101E-06
YFP	7.50254E-05	PGM	1.6416E-05	DLC	4.24101E-06
YIV	7.50254E-05	PKC	1.6416E-05	DMS	4.24101E-06
YMG	7.50254E-05	PVY	1.6416E-05	DMT	4.24101E-06
DGK	7.45918E-05	PWN	1.6416E-05	DNV	4.24101E-06
EEA	7.45918E-05	QWN	1.6416E-05	DPQ	4.24101E-06
ENG	7.45918E-05	QYP	1.6416E-05	DRG	4.24101E-06
FDV	7.45918E-05	RFM	1.6416E-05	DRT	4.24101E-06
KGW	7.45918E-05	RYF	1.6416E-05	DTF	4.24101E-06
KVQ	7.45918E-05	SCM	1.6416E-05	DTL	4.24101E-06
PMW	7.45918E-05	SFY	1.6416E-05	DTR	4.24101E-06
PNH	7.45918E-05	SHE	1.6416E-05	EDT	4.24101E-06
QKA	7.45918E-05	TWF	1.6416E-05	EFT	4.24101E-06
QTE	7.45918E-05	VFT	1.6416E-05	ELH	4.24101E-06
QVC	7.45918E-05	VME	1.6416E-05	ELY	4.24101E-06
SEK	7.45918E-05	VVR	1.6416E-05	ESQ	4.24101E-06
SHF	7.45918E-05	VVV	1.6416E-05	FEY	4.24101E-06
SNY	7.45918E-05	VWQ	1.6416E-05	FPI	4.24101E-06
VMH	7.45918E-05	WIN	1.6416E-05	GFI	4.24101E-06
WFA	7.45918E-05	WPM	1.6416E-05	GKK	4.24101E-06
WHA	7.45918E-05	YAF	1.6416E-05	GMP	4.24101E-06
WHP	7.45918E-05	ACL	1.6059E-05	GVI	4.24101E-06
WPH	7.45918E-05	AID	1.6059E-05	HFS	4.24101E-06
ANM	7.41581E-05	ATY	1.6059E-05	HLV	4.24101E-06
CTH	7.41581E-05	CAH	1.6059E-05	HMT	4.24101E-06
DGC	7.41581E-05	CRT	1.6059E-05	HQR	4.24101E-06
DKV	7.41581E-05	DNL	1.6059E-05	HTP	4.24101E-06
DVI	7.41581E-05	DNM	1.6059E-05	HVH	4.24101E-06
EMP	7.41581E-05	EGI	1.6059E-05	HWS	4.24101E-06
ETE	7.41581E-05	EHA	1.6059E-05	ICM	4.24101E-06
ETI	7.41581E-05	EPF	1.6059E-05	IHR	4.24101E-06
FNG	7.41581E-05	FYE	1.6059E-05	IKQ	4.24101E-06
GDM	7.41581E-05	GQN	1.6059E-05	INY	4.24101E-06
GEH	7.41581E-05	HAD	1.6059E-05	IPK	4.24101E-06
IHT	7.41581E-05	HGH	1.6059E-05	ISK	4.24101E-06

ISF	7.41581E-05	HHL	1.6059E-05	ITA	4.24101E-06
ITW	7.41581E-05	HKR	1.6059E-05	IVK	4.24101E-06
KMV	7.41581E-05	HQL	1.6059E-05	IYR	4.24101E-06
KWP	7.41581E-05	HWT	1.6059E-05	KCD	4.24101E-06
LMY	7.41581E-05	IKN	1.6059E-05	KIC	4.24101E-06
MWS	7.41581E-05	ILD	1.6059E-05	KLH	4.24101E-06
NAY	7.41581E-05	IMM	1.6059E-05	KMP	4.24101E-06
NGC	7.41581E-05	ISQ	1.6059E-05	KNF	4.24101E-06
PKQ	7.41581E-05	ISW	1.6059E-05	KSE	4.24101E-06
PMC	7.41581E-05	ITD	1.6059E-05	KWN	4.24101E-06
QAD	7.41581E-05	ITQ	1.6059E-05	KWQ	4.24101E-06
QET	7.41581E-05	ITY	1.6059E-05	LHK	4.24101E-06
RDY	7.41581E-05	IVR	1.6059E-05	LNE	4.24101E-06
RYY	7.41581E-05	KNY	1.6059E-05	MAR	4.24101E-06
THW	7.41581E-05	LHC	1.6059E-05	MAS	4.24101E-06
TYM	7.41581E-05	LIM	1.6059E-05	MHA	4.24101E-06
VCH	7.41581E-05	LWC	1.6059E-05	MHH	4.24101E-06
VDE	7.41581E-05	MCQ	1.6059E-05	MHP	4.24101E-06
VHW	7.41581E-05	MET	1.6059E-05	MIN	4.24101E-06
VIK	7.41581E-05	MFM	1.6059E-05	MPG	4.24101E-06
WDP	7.41581E-05	MNN	1.6059E-05	MPV	4.24101E-06
WTN	7.41581E-05	MNR	1.6059E-05	MPY	4.24101E-06
CCT	7.37244E-05	MSF	1.6059E-05	MRN	4.24101E-06
CIA	7.37244E-05	NEN	1.6059E-05	MRQ	4.24101E-06
CNA	7.37244E-05	NGI	1.6059E-05	MRY	4.24101E-06
CPM	7.37244E-05	NGQ	1.6059E-05	MTG	4.24101E-06
CTC	7.37244E-05	NGW	1.6059E-05	MTN	4.24101E-06
CVN	7.37244E-05	NYP	1.6059E-05	MWG	4.24101E-06
DWP	7.37244E-05	PTI	1.6059E-05	NGW	4.24101E-06
EDV	7.37244E-05	QAH	1.6059E-05	NLE	4.24101E-06
EVW	7.37244E-05	QHN	1.6059E-05	NPY	4.24101E-06
GYC	7.37244E-05	QLC	1.6059E-05	PAA	4.24101E-06
HMV	7.37244E-05	QMS	1.6059E-05	PAV	4.24101E-06
IHA	7.37244E-05	QSM	1.6059E-05	PEA	4.24101E-06
ITQ	7.37244E-05	QVI	1.6059E-05	PTI	4.24101E-06
NCG	7.37244E-05	REI	1.6059E-05	PTM	4.24101E-06
NKG	7.37244E-05	REV	1.6059E-05	QGS	4.24101E-06
PHQ	7.37244E-05	SII	1.6059E-05	QIA	4.24101E-06
QFA	7.37244E-05	SIQ	1.6059E-05	QLK	4.24101E-06
QPD	7.37244E-05	TCH	1.6059E-05	QMS	4.24101E-06
QTD	7.37244E-05	TDP	1.6059E-05	QQE	4.24101E-06
RCE	7.37244E-05	TIE	1.6059E-05	QQN	4.24101E-06
SFY	7.37244E-05	TNG	1.6059E-05	QRE	4.24101E-06
SKI	7.37244E-05	TVF	1.6059E-05	QRW	4.24101E-06

SQF	7.37244E-05	TWM	1.6059E-05	RAG	4.24101E-06
TIM	7.37244E-05	TWP	1.6059E-05	RCF	4.24101E-06
VDM	7.37244E-05	VCR	1.6059E-05	REW	4.24101E-06
VNC	7.37244E-05	VCV	1.6059E-05	RKV	4.24101E-06
WMP	7.37244E-05	VNN	1.6059E-05	RQQ	4.24101E-06
WYP	7.37244E-05	VTQ	1.6059E-05	RQW	4.24101E-06
ADM	7.32907E-05	VWV	1.6059E-05	SCH	4.24101E-06
DEA	7.32907E-05	WCD	1.6059E-05	SEM	4.24101E-06
EFV	7.32907E-05	WMH	1.6059E-05	SEY	4.24101E-06
EIP	7.32907E-05	WTG	1.6059E-05	SFP	4.24101E-06
EIV	7.32907E-05	YEC	1.6059E-05	SKK	4.24101E-06
EKG	7.32907E-05	YKH	1.6059E-05	SKV	4.24101E-06
HCV	7.32907E-05	YVA	1.6059E-05	SMQ	4.24101E-06
HHG	7.32907E-05	AIH	1.5702E-05	TCC	4.24101E-06
IYA	7.32907E-05	AVQ	1.5702E-05	TCR	4.24101E-06
MNP	7.32907E-05	AWM	1.5702E-05	TEL	4.24101E-06
NFG	7.32907E-05	AYH	1.5702E-05	TFG	4.24101E-06
PMQ	7.32907E-05	CAV	1.5702E-05	TIC	4.24101E-06
QTM	7.32907E-05	CGS	1.5702E-05	TKN	4.24101E-06
SEI	7.32907E-05	CGT	1.5702E-05	TMC	4.24101E-06
TDW	7.32907E-05	CMH	1.5702E-05	TND	4.24101E-06
TIN	7.32907E-05	CWS	1.5702E-05	TQA	4.24101E-06
TMC	7.32907E-05	DCW	1.5702E-05	TQR	4.24101E-06
TMQ	7.32907E-05	DGE	1.5702E-05	TSW	4.24101E-06
TNK	7.32907E-05	DKS	1.5702E-05	TTQ	4.24101E-06
VHQ	7.32907E-05	DVH	1.5702E-05	TVY	4.24101E-06
VKK	7.32907E-05	EAA	1.5702E-05	TWV	4.24101E-06
WHV	7.32907E-05	EDG	1.5702E-05	TYL	4.24101E-06
WRN	7.32907E-05	EEP	1.5702E-05	VAN	4.24101E-06
YCG	7.32907E-05	EHW	1.5702E-05	VKI	4.24101E-06
YEA	7.32907E-05	ERQ	1.5702E-05	VKV	4.24101E-06
YVD	7.32907E-05	ETD	1.5702E-05	VVG	4.24101E-06
AHC	7.28571E-05	FCF	1.5702E-05	VVW	4.24101E-06
AMC	7.28571E-05	FCG	1.5702E-05	VWD	4.24101E-06
CVH	7.28571E-05	FWC	1.5702E-05	VYQ	4.24101E-06
DNG	7.28571E-05	GDD	1.5702E-05	WCA	4.24101E-06
DQA	7.28571E-05	GHM	1.5702E-05	WCT	4.24101E-06
EAM	7.28571E-05	GHN	1.5702E-05	WDT	4.24101E-06
ECP	7.28571E-05	GYR	1.5702E-05	WFY	4.24101E-06
ETK	7.28571E-05	HFS	1.5702E-05	WIQ	4.24101E-06
ETM	7.28571E-05	HHG	1.5702E-05	WMP	4.24101E-06
FTF	7.28571E-05	HRQ	1.5702E-05	WPM	4.24101E-06
FTI	7.28571E-05	HTY	1.5702E-05	WQF	4.24101E-06
FVY	7.28571E-05	HWS	1.5702E-05	YCH	4.24101E-06

GNN	7.28571E-05	IDG	1.5702E-05	YGC	4.24101E-06
KGH	7.28571E-05	IDL	1.5702E-05	YIY	4.24101E-06
KPQ	7.28571E-05	IGH	1.5702E-05	YLE	4.24101E-06
NIG	7.28571E-05	IPC	1.5702E-05	YMW	4.24101E-06
NTF	7.28571E-05	IWT	1.5702E-05	YND	4.24101E-06
QGD	7.28571E-05	KDD	1.5702E-05	YQF	4.24101E-06
QQA	7.28571E-05	KPK	1.5702E-05	AAM	3.99154E-06
RFD	7.28571E-05	KQM	1.5702E-05	AIF	3.99154E-06
RNY	7.28571E-05	LAK	1.5702E-05	AMP	3.99154E-06
SFH	7.28571E-05	LEE	1.5702E-05	ATW	3.99154E-06
THH	7.28571E-05	LFQ	1.5702E-05	AYY	3.99154E-06
VCN	7.28571E-05	LVW	1.5702E-05	CMQ	3.99154E-06
WEA	7.28571E-05	MCW	1.5702E-05	CNP	3.99154E-06
YTN	7.28571E-05	MDP	1.5702E-05	CNW	3.99154E-06
YWP	7.28571E-05	NMD	1.5702E-05	CPS	3.99154E-06
ADC	7.24234E-05	NQV	1.5702E-05	CQA	3.99154E-06
CDP	7.24234E-05	NRK	1.5702E-05	CWN	3.99154E-06
CIT	7.24234E-05	PKW	1.5702E-05	DAG	3.99154E-06
CPW	7.24234E-05	QAA	1.5702E-05	DPI	3.99154E-06
CWP	7.24234E-05	QDC	1.5702E-05	DPY	3.99154E-06
DGQ	7.24234E-05	QES	1.5702E-05	DRV	3.99154E-06
EMA	7.24234E-05	QFN	1.5702E-05	DTM	3.99154E-06
GFN	7.24234E-05	QGF	1.5702E-05	DTT	3.99154E-06
GKM	7.24234E-05	QYA	1.5702E-05	EDD	3.99154E-06
GKY	7.24234E-05	RHC	1.5702E-05	EDF	3.99154E-06
KFV	7.24234E-05	RKW	1.5702E-05	EHC	3.99154E-06
NPD	7.24234E-05	SFN	1.5702E-05	EHF	3.99154E-06
NTD	7.24234E-05	SHK	1.5702E-05	ELN	3.99154E-06
PDW	7.24234E-05	SKH	1.5702E-05	EPW	3.99154E-06
PKC	7.24234E-05	SMC	1.5702E-05	EQG	3.99154E-06
PKM	7.24234E-05	SWE	1.5702E-05	EQP	3.99154E-06
PQH	7.24234E-05	TDV	1.5702E-05	ERR	3.99154E-06
QHG	7.24234E-05	TGW	1.5702E-05	ETV	3.99154E-06
RFI	7.24234E-05	TMT	1.5702E-05	EYF	3.99154E-06
SFF	7.24234E-05	TTH	1.5702E-05	FDM	3.99154E-06
TKW	7.24234E-05	VDT	1.5702E-05	FEN	3.99154E-06
VNI	7.24234E-05	VHH	1.5702E-05	FFD	3.99154E-06
VWM	7.24234E-05	VPF	1.5702E-05	FIW	3.99154E-06
WMA	7.24234E-05	VVG	1.5702E-05	GFM	3.99154E-06
WVK	7.24234E-05	WIV	1.5702E-05	GMQ	3.99154E-06
YIA	7.24234E-05	WMY	1.5702E-05	GVC	3.99154E-06
AEQ	7.19897E-05	YEA	1.5702E-05	HCT	3.99154E-06
CQP	7.19897E-05	YYQ	1.5702E-05	HEY	3.99154E-06
EAN	7.19897E-05	AAV	1.5345E-05	HKM	3.99154E-06

FCG	7.19897E-05	ACE	1.5345E-05	HKP	3.99154E-06
FGC	7.19897E-05	ACP	1.5345E-05	HQS	3.99154E-06
FIV	7.19897E-05	AIR	1.5345E-05	HRA	3.99154E-06
FTE	7.19897E-05	AKY	1.5345E-05	HRP	3.99154E-06
GDN	7.19897E-05	AQI	1.5345E-05	HVL	3.99154E-06
GFW	7.19897E-05	ASY	1.5345E-05	IEP	3.99154E-06
GKE	7.19897E-05	DAG	1.5345E-05	IIV	3.99154E-06
HAF	7.19897E-05	DAN	1.5345E-05	ILE	3.99154E-06
IAQ	7.19897E-05	DML	1.5345E-05	IPE	3.99154E-06
MHP	7.19897E-05	DPF	1.5345E-05	ISH	3.99154E-06
NGN	7.19897E-05	DPG	1.5345E-05	ITH	3.99154E-06
PHH	7.19897E-05	DSY	1.5345E-05	ITQ	3.99154E-06
QCP	7.19897E-05	ECT	1.5345E-05	KAG	3.99154E-06
QTK	7.19897E-05	EFG	1.5345E-05	KEA	3.99154E-06
THE	7.19897E-05	EGF	1.5345E-05	KGN	3.99154E-06
TMN	7.19897E-05	EIM	1.5345E-05	KGP	3.99154E-06
TNQ	7.19897E-05	EMR	1.5345E-05	KID	3.99154E-06
VCD	7.19897E-05	EQV	1.5345E-05	KKD	3.99154E-06
WMT	7.19897E-05	EYN	1.5345E-05	KNY	3.99154E-06
WWV	7.19897E-05	FDR	1.5345E-05	KQL	3.99154E-06
YAF	7.19897E-05	FYG	1.5345E-05	KWV	3.99154E-06
YGK	7.19897E-05	GCR	1.5345E-05	LEF	3.99154E-06
YGQ	7.19897E-05	HPM	1.5345E-05	LME	3.99154E-06
YVY	7.19897E-05	HTI	1.5345E-05	MCC	3.99154E-06
DGY	7.1556E-05	HYA	1.5345E-05	MDS	3.99154E-06
DIV	7.1556E-05	IEL	1.5345E-05	MEL	3.99154E-06
DPD	7.1556E-05	IFH	1.5345E-05	MFT	3.99154E-06
DPN	7.1556E-05	IYS	1.5345E-05	MIC	3.99154E-06
EGD	7.1556E-05	KDG	1.5345E-05	MKA	3.99154E-06
EMV	7.1556E-05	KEQ	1.5345E-05	MLW	3.99154E-06
EPE	7.1556E-05	KHF	1.5345E-05	MRD	3.99154E-06
EVD	7.1556E-05	KIL	1.5345E-05	MRF	3.99154E-06
EVQ	7.1556E-05	LMY	1.5345E-05	NMA	3.99154E-06
HEV	7.1556E-05	MSD	1.5345E-05	NPA	3.99154E-06
IGK	7.1556E-05	MTY	1.5345E-05	NQA	3.99154E-06
KEA	7.1556E-05	MVR	1.5345E-05	NRA	3.99154E-06
KPY	7.1556E-05	MYL	1.5345E-05	NRN	3.99154E-06
MCT	7.1556E-05	NHT	1.5345E-05	PCF	3.99154E-06
MYP	7.1556E-05	NQA	1.5345E-05	PDP	3.99154E-06
NMG	7.1556E-05	NYL	1.5345E-05	PMI	3.99154E-06
NPY	7.1556E-05	PGK	1.5345E-05	PMK	3.99154E-06
NWV	7.1556E-05	QCF	1.5345E-05	QFA	3.99154E-06
PCM	7.1556E-05	QNI	1.5345E-05	QPT	3.99154E-06
PHK	7.1556E-05	QTD	1.5345E-05	QVR	3.99154E-06

VEM	7.1556E-05	QTF	1.5345E-05	RAV	3.99154E-06
VHN	7.1556E-05	RCH	1.5345E-05	RCK	3.99154E-06
VKY	7.1556E-05	SAI	1.5345E-05	RDV	3.99154E-06
WWT	7.1556E-05	SAW	1.5345E-05	RDW	3.99154E-06
YAE	7.1556E-05	SHN	1.5345E-05	RFQ	3.99154E-06
YNG	7.1556E-05	STE	1.5345E-05	RMD	3.99154E-06
YPY	7.1556E-05	SWM	1.5345E-05	RVV	3.99154E-06
AMD	7.11224E-05	TMY	1.5345E-05	SAK	3.99154E-06
CVQ	7.11224E-05	TYQ	1.5345E-05	SFH	3.99154E-06
DCG	7.11224E-05	VKK	1.5345E-05	SFM	3.99154E-06
DEQ	7.11224E-05	VNR	1.5345E-05	SHE	3.99154E-06
DPY	7.11224E-05	VQQ	1.5345E-05	SMA	3.99154E-06
DVD	7.11224E-05	WAG	1.5345E-05	STI	3.99154E-06
EAF	7.11224E-05	WQA	1.5345E-05	SVD	3.99154E-06
EGQ	7.11224E-05	WQP	1.5345E-05	TAD	3.99154E-06
EPN	7.11224E-05	YKP	1.5345E-05	TDT	3.99154E-06
ETY	7.11224E-05	YLK	1.5345E-05	TKY	3.99154E-06
FCP	7.11224E-05	YNV	1.5345E-05	TQS	3.99154E-06
FGD	7.11224E-05	YPE	1.5345E-05	VGA	3.99154E-06
FGN	7.11224E-05	YVY	1.5345E-05	VHY	3.99154E-06
FIG	7.11224E-05	AFC	1.4988E-05	VLD	3.99154E-06
FKA	7.11224E-05	AKD	1.4988E-05	VMA	3.99154E-06
FQG	7.11224E-05	AMF	1.4988E-05	VMD	3.99154E-06
FVH	7.11224E-05	ANK	1.4988E-05	VMF	3.99154E-06
GHH	7.11224E-05	AYG	1.4988E-05	VPG	3.99154E-06
HAD	7.11224E-05	DKY	1.4988E-05	VQF	3.99154E-06
HQV	7.11224E-05	EFC	1.4988E-05	VQV	3.99154E-06
IIV	7.11224E-05	EQP	1.4988E-05	VTM	3.99154E-06
MSF	7.11224E-05	FFG	1.4988E-05	VYM	3.99154E-06
QNV	7.11224E-05	FLD	1.4988E-05	WAA	3.99154E-06
RWQ	7.11224E-05	FNC	1.4988E-05	WFF	3.99154E-06
SWN	7.11224E-05	FNG	1.4988E-05	WFN	3.99154E-06
TKM	7.11224E-05	FQG	1.4988E-05	WIT	3.99154E-06
VCK	7.11224E-05	FYV	1.4988E-05	WRI	3.99154E-06
VYW	7.11224E-05	GWG	1.4988E-05	WVM	3.99154E-06
WCA	7.11224E-05	GYI	1.4988E-05	YDY	3.99154E-06
WGK	7.11224E-05	HDG	1.4988E-05	YGM	3.99154E-06
YCV	7.11224E-05	HLQ	1.4988E-05	YNQ	3.99154E-06
YPE	7.11224E-05	HWH	1.4988E-05	YPQ	3.99154E-06
ADW	7.06887E-05	IKA	1.4988E-05	YQP	3.99154E-06
AKQ	7.06887E-05	ILW	1.4988E-05	YRE	3.99154E-06
CAE	7.06887E-05	IMN	1.4988E-05	YRF	3.99154E-06
CAQ	7.06887E-05	IQQ	1.4988E-05	ADA	3.74207E-06
CYP	7.06887E-05	KIA	1.4988E-05	AFA	3.74207E-06

DFV	7.06887E-05	KKN	1.4988E-05	AID	3.74207E-06
EVF	7.06887E-05	KVA	1.4988E-05	AIH	3.74207E-06
EWP	7.06887E-05	LDN	1.4988E-05	AKE	3.74207E-06
FYV	7.06887E-05	LDY	1.4988E-05	ANM	3.74207E-06
GQM	7.06887E-05	MIT	1.4988E-05	AQE	3.74207E-06
HQA	7.06887E-05	MTW	1.4988E-05	AQG	3.74207E-06
HYG	7.06887E-05	NFG	1.4988E-05	AWV	3.74207E-06
IAN	7.06887E-05	NKI	1.4988E-05	AYF	3.74207E-06
IMG	7.06887E-05	NRW	1.4988E-05	CAR	3.74207E-06
LQY	7.06887E-05	NVI	1.4988E-05	CCQ	3.74207E-06
NIV	7.06887E-05	PAM	1.4988E-05	CGM	3.74207E-06
NQG	7.06887E-05	PCP	1.4988E-05	CGW	3.74207E-06
PCQ	7.06887E-05	PGY	1.4988E-05	CHE	3.74207E-06
QPE	7.06887E-05	PVE	1.4988E-05	CHV	3.74207E-06
REY	7.06887E-05	QGG	1.4988E-05	CLK	3.74207E-06
RYF	7.06887E-05	QNV	1.4988E-05	CMC	3.74207E-06
SCI	7.06887E-05	RAM	1.4988E-05	CMW	3.74207E-06
TEM	7.06887E-05	RCC	1.4988E-05	CNT	3.74207E-06
TFM	7.06887E-05	RIN	1.4988E-05	CPH	3.74207E-06
TKH	7.06887E-05	RWM	1.4988E-05	CPR	3.74207E-06
VIQ	7.06887E-05	SKI	1.4988E-05	CPY	3.74207E-06
VKE	7.06887E-05	SMY	1.4988E-05	CRA	3.74207E-06
VMQ	7.06887E-05	SNM	1.4988E-05	CTH	3.74207E-06
WIA	7.06887E-05	TFH	1.4988E-05	CWI	3.74207E-06
YEG	7.06887E-05	TWN	1.4988E-05	DAW	3.74207E-06
AEH	7.0255E-05	VFP	1.4988E-05	DCT	3.74207E-06
CAW	7.0255E-05	VGK	1.4988E-05	DDT	3.74207E-06
CCP	7.0255E-05	VGN	1.4988E-05	DFG	3.74207E-06
CIP	7.0255E-05	VGW	1.4988E-05	DHC	3.74207E-06
CVW	7.0255E-05	VNG	1.4988E-05	DHM	3.74207E-06
DGF	7.0255E-05	VPY	1.4988E-05	DKI	3.74207E-06
ETF	7.0255E-05	VQI	1.4988E-05	DPG	3.74207E-06
GFM	7.0255E-05	WAV	1.4988E-05	DQE	3.74207E-06
HAE	7.0255E-05	WDC	1.4988E-05	DQV	3.74207E-06
IQT	7.0255E-05	WEA	1.4988E-05	DSI	3.74207E-06
IVK	7.0255E-05	WGW	1.4988E-05	EIT	3.74207E-06
KEV	7.0255E-05	WIP	1.4988E-05	EKT	3.74207E-06
MFP	7.0255E-05	WKY	1.4988E-05	EKW	3.74207E-06
MNA	7.0255E-05	WMV	1.4988E-05	EPI	3.74207E-06
NEG	7.0255E-05	WVC	1.4988E-05	EQY	3.74207E-06
QAC	7.0255E-05	YMV	1.4988E-05	ERF	3.74207E-06
QAQ	7.0255E-05	YRC	1.4988E-05	EVL	3.74207E-06
QMG	7.0255E-05	AFP	1.4631E-05	FEE	3.74207E-06
THC	7.0255E-05	AIN	1.4631E-05	FEK	3.74207E-06

VHH	7.0255E-05	AIY	1.4631E-05	FGY	3.74207E-06
WGH	7.0255E-05	AMY	1.4631E-05	FHN	3.74207E-06
WVW	7.0255E-05	AVG	1.4631E-05	FIA	3.74207E-06
YGN	7.0255E-05	CDA	1.4631E-05	FIC	3.74207E-06
YPF	7.0255E-05	CMS	1.4631E-05	FME	3.74207E-06
YYV	7.0255E-05	CWF	1.4631E-05	FMH	3.74207E-06
AKD	6.98213E-05	DAH	1.4631E-05	FVY	3.74207E-06
AQM	6.98213E-05	DAQ	1.4631E-05	GGC	3.74207E-06
CET	6.98213E-05	DCD	1.4631E-05	HAS	3.74207E-06
CRF	6.98213E-05	EDC	1.4631E-05	HCA	3.74207E-06
DGI	6.98213E-05	END	1.4631E-05	HDP	3.74207E-06
DGN	6.98213E-05	ERW	1.4631E-05	HEA	3.74207E-06
DPF	6.98213E-05	FAM	1.4631E-05	HFC	3.74207E-06
FQA	6.98213E-05	FIL	1.4631E-05	HHD	3.74207E-06
FTY	6.98213E-05	FPK	1.4631E-05	HHV	3.74207E-06
FWA	6.98213E-05	GKW	1.4631E-05	HKT	3.74207E-06
GDC	6.98213E-05	HAP	1.4631E-05	HNW	3.74207E-06
GKH	6.98213E-05	HCF	1.4631E-05	HQQ	3.74207E-06
GMN	6.98213E-05	HHH	1.4631E-05	IIC	3.74207E-06
IPM	6.98213E-05	HMH	1.4631E-05	IKV	3.74207E-06
KIG	6.98213E-05	HPF	1.4631E-05	IMY	3.74207E-06
MHA	6.98213E-05	HPY	1.4631E-05	IPC	3.74207E-06
MPM	6.98213E-05	HSM	1.4631E-05	ISY	3.74207E-06
MTM	6.98213E-05	HTF	1.4631E-05	ITF	3.74207E-06
MVW	6.98213E-05	HVA	1.4631E-05	IWK	3.74207E-06
PNQ	6.98213E-05	IFP	1.4631E-05	KAA	3.74207E-06
QPK	6.98213E-05	IQS	1.4631E-05	KCC	3.74207E-06
QTQ	6.98213E-05	ITI	1.4631E-05	KCF	3.74207E-06
VMI	6.98213E-05	IWA	1.4631E-05	KCH	3.74207E-06
VND	6.98213E-05	IWV	1.4631E-05	KGW	3.74207E-06
WKA	6.98213E-05	KEC	1.4631E-05	KIP	3.74207E-06
WNG	6.98213E-05	KFH	1.4631E-05	KPV	3.74207E-06
WVN	6.98213E-05	KIV	1.4631E-05	KQG	3.74207E-06
YGF	6.98213E-05	KMR	1.4631E-05	KQP	3.74207E-06
AKC	6.93877E-05	KRK	1.4631E-05	KTW	3.74207E-06
AQH	6.93877E-05	KTF	1.4631E-05	MAL	3.74207E-06
CAD	6.93877E-05	KVT	1.4631E-05	MNI	3.74207E-06
CTD	6.93877E-05	KWT	1.4631E-05	MTA	3.74207E-06
DEV	6.93877E-05	KYQ	1.4631E-05	MTW	3.74207E-06
EAQ	6.93877E-05	LEI	1.4631E-05	NCG	3.74207E-06
EHG	6.93877E-05	LYY	1.4631E-05	NDE	3.74207E-06
FMG	6.93877E-05	MAC	1.4631E-05	NEG	3.74207E-06
GDD	6.93877E-05	MGV	1.4631E-05	NFH	3.74207E-06
HAY	6.93877E-05	MLC	1.4631E-05	NGS	3.74207E-06

HGE	6.93877E-05	MRF	1.4631E-05	NQL	3.74207E-06
HQG	6.93877E-05	NAE	1.4631E-05	NQN	3.74207E-06
HVY	6.93877E-05	NKN	1.4631E-05	NRF	3.74207E-06
HWA	6.93877E-05	PMD	1.4631E-05	NSI	3.74207E-06
IFT	6.93877E-05	PWD	1.4631E-05	NVW	3.74207E-06
KTF	6.93877E-05	QAM	1.4631E-05	NWK	3.74207E-06
KVF	6.93877E-05	QAT	1.4631E-05	PEN	3.74207E-06
MSI	6.93877E-05	QCS	1.4631E-05	PGA	3.74207E-06
PKH	6.93877E-05	QRQ	1.4631E-05	PGG	3.74207E-06
PYC	6.93877E-05	QWS	1.4631E-05	PNV	3.74207E-06
QQT	6.93877E-05	QYL	1.4631E-05	PVF	3.74207E-06
QVQ	6.93877E-05	REY	1.4631E-05	PVW	3.74207E-06
RCD	6.93877E-05	SMV	1.4631E-05	PWD	3.74207E-06
RFF	6.93877E-05	SYI	1.4631E-05	PWK	3.74207E-06
RFY	6.93877E-05	TCF	1.4631E-05	QDM	3.74207E-06
RII	6.93877E-05	TNW	1.4631E-05	QEN	3.74207E-06
TIH	6.93877E-05	TTM	1.4631E-05	QFL	3.74207E-06
VKI	6.93877E-05	VAM	1.4631E-05	QFN	3.74207E-06
WAW	6.93877E-05	VSC	1.4631E-05	QMY	3.74207E-06
WQT	6.93877E-05	WCC	1.4631E-05	QSQ	3.74207E-06
YAY	6.93877E-05	WDS	1.4631E-05	QSV	3.74207E-06
YFG	6.93877E-05	WMM	1.4631E-05	QVY	3.74207E-06
DAY	6.8954E-05	WRM	1.4631E-05	REE	3.74207E-06
EAD	6.8954E-05	WSE	1.4631E-05	REV	3.74207E-06
GMK	6.8954E-05	YKC	1.4631E-05	RFE	3.74207E-06
HDV	6.8954E-05	YKD	1.4631E-05	RKM	3.74207E-06
HVK	6.8954E-05	YMW	1.4631E-05	RTE	3.74207E-06
IPN	6.8954E-05	YSH	1.4631E-05	RVC	3.74207E-06
KVI	6.8954E-05	AAY	1.4274E-05	SAN	3.74207E-06
PFM	6.8954E-05	ADV	1.4274E-05	SCM	3.74207E-06
PIN	6.8954E-05	AEF	1.4274E-05	SGN	3.74207E-06
QAN	6.8954E-05	AYV	1.4274E-05	SIE	3.74207E-06
QGC	6.8954E-05	CAS	1.4274E-05	SMN	3.74207E-06
QWA	6.8954E-05	CSN	1.4274E-05	SVY	3.74207E-06
RDI	6.8954E-05	CVQ	1.4274E-05	TAQ	3.74207E-06
RWE	6.8954E-05	DHP	1.4274E-05	TGA	3.74207E-06
TCM	6.8954E-05	DNQ	1.4274E-05	TGM	3.74207E-06
THD	6.8954E-05	DWM	1.4274E-05	THL	3.74207E-06
TMK	6.8954E-05	DWW	1.4274E-05	TID	3.74207E-06
TMW	6.8954E-05	EER	1.4274E-05	TKQ	3.74207E-06
VMK	6.8954E-05	EIG	1.4274E-05	TNG	3.74207E-06
VQD	6.8954E-05	ERK	1.4274E-05	TNN	3.74207E-06
WIP	6.8954E-05	EWR	1.4274E-05	TPF	3.74207E-06
WTM	6.8954E-05	EYT	1.4274E-05	TSH	3.74207E-06

WWA	6.8954E-05	FNW	1.4274E-05	TSM	3.74207E-06
AFM	6.85203E-05	GAI	1.4274E-05	TSY	3.74207E-06
AQQ	6.85203E-05	GAM	1.4274E-05	TTF	3.74207E-06
CAM	6.85203E-05	GCI	1.4274E-05	TTK	3.74207E-06
CCV	6.85203E-05	GCQ	1.4274E-05	TWG	3.74207E-06
CMV	6.85203E-05	GHV	1.4274E-05	VAG	3.74207E-06
DVF	6.85203E-05	GIK	1.4274E-05	VDP	3.74207E-06
FVF	6.85203E-05	GIW	1.4274E-05	VFG	3.74207E-06
GCW	6.85203E-05	GKG	1.4274E-05	VFP	3.74207E-06
GFC	6.85203E-05	HFA	1.4274E-05	VGC	3.74207E-06
HFG	6.85203E-05	HFN	1.4274E-05	VGF	3.74207E-06
HGI	6.85203E-05	HKP	1.4274E-05	VHN	3.74207E-06
HGN	6.85203E-05	HLY	1.4274E-05	VQI	3.74207E-06
IIA	6.85203E-05	HTN	1.4274E-05	VTV	3.74207E-06
KGQ	6.85203E-05	ICA	1.4274E-05	VVK	3.74207E-06
KPE	6.85203E-05	IER	1.4274E-05	WEN	3.74207E-06
KTY	6.85203E-05	IHE	1.4274E-05	WMD	3.74207E-06
MFT	6.85203E-05	IPM	1.4274E-05	WYK	3.74207E-06
MPH	6.85203E-05	IYA	1.4274E-05	YAP	3.74207E-06
PFW	6.85203E-05	KMY	1.4274E-05	YDT	3.74207E-06
PNK	6.85203E-05	KVC	1.4274E-05	YEE	3.74207E-06
PQE	6.85203E-05	KYC	1.4274E-05	YET	3.74207E-06
QAH	6.85203E-05	LHM	1.4274E-05	YFI	3.74207E-06
QWP	6.85203E-05	LII	1.4274E-05	YFV	3.74207E-06
QWT	6.85203E-05	MAE	1.4274E-05	YHY	3.74207E-06
SYY	6.85203E-05	MQT	1.4274E-05	YKF	3.74207E-06
VDF	6.85203E-05	MYI	1.4274E-05	YMH	3.74207E-06
VYH	6.85203E-05	NWQ	1.4274E-05	YQM	3.74207E-06
WCP	6.85203E-05	PEQ	1.4274E-05	YQY	3.74207E-06
AIM	6.80867E-05	PYD	1.4274E-05	YTM	3.74207E-06
ANN	6.80867E-05	QIS	1.4274E-05	YVS	3.74207E-06
CAC	6.80867E-05	QQH	1.4274E-05	YWK	3.74207E-06
CPC	6.80867E-05	RKD	1.4274E-05	AAV	3.4926E-06
DQG	6.80867E-05	RWW	1.4274E-05	ADT	3.4926E-06
EDG	6.80867E-05	SDF	1.4274E-05	AFF	3.4926E-06
EEP	6.80867E-05	SIM	1.4274E-05	AFR	3.4926E-06
FWM	6.80867E-05	SNK	1.4274E-05	AFY	3.4926E-06
GDW	6.80867E-05	SYD	1.4274E-05	AKV	3.4926E-06
GMF	6.80867E-05	TKF	1.4274E-05	CAY	3.4926E-06
IAH	6.80867E-05	VEY	1.4274E-05	CCV	3.4926E-06
IMA	6.80867E-05	VWH	1.4274E-05	CEC	3.4926E-06
IPH	6.80867E-05	VWT	1.4274E-05	CEW	3.4926E-06
IVN	6.80867E-05	WFW	1.4274E-05	CHC	3.4926E-06
KAY	6.80867E-05	WKF	1.4274E-05	CKQ	3.4926E-06

MPY	6.80867E-05	YGQ	1.4274E-05	CLE	3.4926E-06
MSD	6.80867E-05	YKQ	1.4274E-05	CPV	3.4926E-06
MTW	6.80867E-05	YNY	1.4274E-05	CYF	3.4926E-06
PDN	6.80867E-05	YVV	1.4274E-05	CYQ	3.4926E-06
PEM	6.80867E-05	YWA	1.4274E-05	DEG	3.4926E-06
QCT	6.80867E-05	YWC	1.4274E-05	DFR	3.4926E-06
SCF	6.80867E-05	YYP	1.4274E-05	DHE	3.4926E-06
TKD	6.80867E-05	ACD	1.3918E-05	DLE	3.4926E-06
VFD	6.80867E-05	AEG	1.3918E-05	DNA	3.4926E-06
WAC	6.80867E-05	AFA	1.3918E-05	DPA	3.4926E-06
WDV	6.80867E-05	AHM	1.3918E-05	DPP	3.4926E-06
WTH	6.80867E-05	AHN	1.3918E-05	DTP	3.4926E-06
WWP	6.80867E-05	AHR	1.3918E-05	DVP	3.4926E-06
ACW	6.7653E-05	AMI	1.3918E-05	DWA	3.4926E-06
AEM	6.7653E-05	AVH	1.3918E-05	EAW	3.4926E-06
FAH	6.7653E-05	CNT	1.3918E-05	EHW	3.4926E-06
FHV	6.7653E-05	CTH	1.3918E-05	ELC	3.4926E-06
GHQ	6.7653E-05	DDA	1.3918E-05	ELK	3.4926E-06
GNY	6.7653E-05	DDH	1.3918E-05	ELQ	3.4926E-06
ITH	6.7653E-05	DMM	1.3918E-05	EMF	3.4926E-06
MIT	6.7653E-05	DNN	1.3918E-05	EMI	3.4926E-06
MKP	6.7653E-05	DYA	1.3918E-05	EMM	3.4926E-06
MPQ	6.7653E-05	EAG	1.3918E-05	EMN	3.4926E-06
NGY	6.7653E-05	EDN	1.3918E-05	ERV	3.4926E-06
NYG	6.7653E-05	EEE	1.3918E-05	EYV	3.4926E-06
PEQ	6.7653E-05	EMI	1.3918E-05	FAI	3.4926E-06
PNE	6.7653E-05	EVG	1.3918E-05	FAN	3.4926E-06
QGM	6.7653E-05	EYA	1.3918E-05	FEI	3.4926E-06
QTI	6.7653E-05	FDI	1.3918E-05	FHM	3.4926E-06
RWY	6.7653E-05	FFM	1.3918E-05	FQC	3.4926E-06
SQI	6.7653E-05	FGI	1.3918E-05	GAW	3.4926E-06
SWI	6.7653E-05	FQA	1.3918E-05	GDE	3.4926E-06
TCC	6.7653E-05	GCY	1.3918E-05	GFN	3.4926E-06
TKN	6.7653E-05	GYN	1.3918E-05	GGW	3.4926E-06
TNH	6.7653E-05	HFC	1.3918E-05	GIH	3.4926E-06
TYW	6.7653E-05	HGD	1.3918E-05	GVP	3.4926E-06
VNH	6.7653E-05	HIS	1.3918E-05	GWE	3.4926E-06
WAM	6.7653E-05	HNK	1.3918E-05	HFP	3.4926E-06
WET	6.7653E-05	IHS	1.3918E-05	HIG	3.4926E-06
YQA	6.7653E-05	ITV	1.3918E-05	HPE	3.4926E-06
AKW	6.72193E-05	IWH	1.3918E-05	HPP	3.4926E-06
AQD	6.72193E-05	KDY	1.3918E-05	HQH	3.4926E-06
CKA	6.72193E-05	KEA	1.3918E-05	HTQ	3.4926E-06
CKV	6.72193E-05	KFN	1.3918E-05	HYE	3.4926E-06

CQT	6.72193E-05	KTN	1.3918E-05	IAC	3.4926E-06
DQV	6.72193E-05	KWG	1.3918E-05	IAD	3.4926E-06
DYG	6.72193E-05	LDE	1.3918E-05	ICA	3.4926E-06
EGE	6.72193E-05	LEY	1.3918E-05	ICH	3.4926E-06
EGK	6.72193E-05	MMA	1.3918E-05	IEN	3.4926E-06
FPD	6.72193E-05	MPW	1.3918E-05	IET	3.4926E-06
GIN	6.72193E-05	MYR	1.3918E-05	IGN	3.4926E-06
GNK	6.72193E-05	NCL	1.3918E-05	IIA	3.4926E-06
GNW	6.72193E-05	NCT	1.3918E-05	IWI	3.4926E-06
NEV	6.72193E-05	NKM	1.3918E-05	KAT	3.4926E-06
PHM	6.72193E-05	NVH	1.3918E-05	KCL	3.4926E-06
PKW	6.72193E-05	PMT	1.3918E-05	KCV	3.4926E-06
PQN	6.72193E-05	PYC	1.3918E-05	KFC	3.4926E-06
RQY	6.72193E-05	QQT	1.3918E-05	KFH	3.4926E-06
THQ	6.72193E-05	RCP	1.3918E-05	KGK	3.4926E-06
TKE	6.72193E-05	RDY	1.3918E-05	KGS	3.4926E-06
VFN	6.72193E-05	RIY	1.3918E-05	KKQ	3.4926E-06
YFV	6.72193E-05	RMF	1.3918E-05	KSW	3.4926E-06
ACD	6.67856E-05	RNI	1.3918E-05	KTL	3.4926E-06
AYH	6.67856E-05	SCN	1.3918E-05	KVK	3.4926E-06
CGM	6.67856E-05	SED	1.3918E-05	KVL	3.4926E-06
EGW	6.67856E-05	SHI	1.3918E-05	KWH	3.4926E-06
FDG	6.67856E-05	SVF	1.3918E-05	KWM	3.4926E-06
FVI	6.67856E-05	TEV	1.3918E-05	LDV	3.4926E-06
GHM	6.67856E-05	TVK	1.3918E-05	LIA	3.4926E-06
INV	6.67856E-05	TYM	1.3918E-05	LQE	3.4926E-06
IVF	6.67856E-05	WEM	1.3918E-05	MGT	3.4926E-06
IWP	6.67856E-05	WMQ	1.3918E-05	MIG	3.4926E-06
KGK	6.67856E-05	WYR	1.3918E-05	MKG	3.4926E-06
NPI	6.67856E-05	YDV	1.3918E-05	MMA	3.4926E-06
PDE	6.67856E-05	YKI	1.3918E-05	MNF	3.4926E-06
PND	6.67856E-05	YML	1.3918E-05	MPP	3.4926E-06
TFQ	6.67856E-05	AHG	1.3561E-05	MWH	3.4926E-06
THN	6.67856E-05	CFF	1.3561E-05	MWQ	3.4926E-06
TIK	6.67856E-05	DAY	1.3561E-05	NCD	3.4926E-06
TMY	6.67856E-05	DCR	1.3561E-05	NCH	3.4926E-06
TNE	6.67856E-05	DEA	1.3561E-05	NCN	3.4926E-06
VFQ	6.67856E-05	DKR	1.3561E-05	NGA	3.4926E-06
VNN	6.67856E-05	DTN	1.3561E-05	NHL	3.4926E-06
WDG	6.67856E-05	DYF	1.3561E-05	NMP	3.4926E-06
AKN	6.6352E-05	EHY	1.3561E-05	NPN	3.4926E-06
AND	6.6352E-05	ENG	1.3561E-05	NWE	3.4926E-06
AYC	6.6352E-05	ERM	1.3561E-05	PDD	3.4926E-06
CVM	6.6352E-05	FDV	1.3561E-05	PEW	3.4926E-06

EGM	6.6352E-05	FIF	1.3561E-05	PGC	3.4926E-06
EQV	6.6352E-05	FPM	1.3561E-05	PQC	3.4926E-06
FAY	6.6352E-05	GEG	1.3561E-05	PYV	3.4926E-06
FGK	6.6352E-05	GNW	1.3561E-05	QAL	3.4926E-06
FQV	6.6352E-05	GQF	1.3561E-05	QAY	3.4926E-06
GIW	6.6352E-05	GQI	1.3561E-05	QCG	3.4926E-06
IAM	6.6352E-05	GVI	1.3561E-05	QDN	3.4926E-06
KQV	6.6352E-05	GYQ	1.3561E-05	QIL	3.4926E-06
MPK	6.6352E-05	HAY	1.3561E-05	QML	3.4926E-06
MTN	6.6352E-05	HIP	1.3561E-05	QMT	3.4926E-06
MYA	6.6352E-05	HNF	1.3561E-05	QQL	3.4926E-06
NCV	6.6352E-05	IFT	1.3561E-05	QVS	3.4926E-06
NGD	6.6352E-05	IGD	1.3561E-05	QWD	3.4926E-06
NQV	6.6352E-05	ILM	1.3561E-05	QWH	3.4926E-06
PDM	6.6352E-05	IPN	1.3561E-05	QWP	3.4926E-06
PKD	6.6352E-05	IYQ	1.3561E-05	RGI	3.4926E-06
PQM	6.6352E-05	KCN	1.3561E-05	RMF	3.4926E-06
PYQ	6.6352E-05	KDF	1.3561E-05	RMK	3.4926E-06
QDG	6.6352E-05	KFI	1.3561E-05	RQV	3.4926E-06
QMV	6.6352E-05	KQP	1.3561E-05	SCE	3.4926E-06
QVH	6.6352E-05	KTV	1.3561E-05	SED	3.4926E-06
QVN	6.6352E-05	LEG	1.3561E-05	SEG	3.4926E-06
VCQ	6.6352E-05	LWD	1.3561E-05	SGY	3.4926E-06
VKD	6.6352E-05	MML	1.3561E-05	SNQ	3.4926E-06
VQQ	6.6352E-05	MYN	1.3561E-05	SQE	3.4926E-06
VYD	6.6352E-05	NAI	1.3561E-05	SQW	3.4926E-06
WHG	6.6352E-05	NAK	1.3561E-05	SVP	3.4926E-06
AHW	6.59183E-05	NKR	1.3561E-05	SWC	3.4926E-06
ANE	6.59183E-05	NMP	1.3561E-05	TFC	3.4926E-06
CFA	6.59183E-05	NNN	1.3561E-05	TGG	3.4926E-06
CHT	6.59183E-05	NPW	1.3561E-05	TGK	3.4926E-06
DEG	6.59183E-05	NQL	1.3561E-05	TMI	3.4926E-06
EVN	6.59183E-05	NQP	1.3561E-05	TPK	3.4926E-06
EWV	6.59183E-05	PDI	1.3561E-05	TRP	3.4926E-06
FGF	6.59183E-05	PEF	1.3561E-05	TSE	3.4926E-06
GHN	6.59183E-05	PFD	1.3561E-05	TSQ	3.4926E-06
KGE	6.59183E-05	PIF	1.3561E-05	TVS	3.4926E-06
MMP	6.59183E-05	PWW	1.3561E-05	TWQ	3.4926E-06
MSY	6.59183E-05	PYE	1.3561E-05	TWS	3.4926E-06
NGF	6.59183E-05	QDV	1.3561E-05	VAP	3.4926E-06
PDQ	6.59183E-05	QEL	1.3561E-05	VAV	3.4926E-06
PFQ	6.59183E-05	QEP	1.3561E-05	VCD	3.4926E-06
PIQ	6.59183E-05	QGN	1.3561E-05	VCI	3.4926E-06
PWQ	6.59183E-05	QLD	1.3561E-05	VIW	3.4926E-06

PYH	6.59183E-05	RDE	1.3561E-05	VNV	3.4926E-06
QTY	6.59183E-05	REW	1.3561E-05	VVH	3.4926E-06
RIY	6.59183E-05	RMC	1.3561E-05	VYG	3.4926E-06
TDD	6.59183E-05	RQV	1.3561E-05	WAF	3.4926E-06
TIQ	6.59183E-05	RWC	1.3561E-05	WIE	3.4926E-06
TYQ	6.59183E-05	SDK	1.3561E-05	WPK	3.4926E-06
VDK	6.59183E-05	SIH	1.3561E-05	YCT	3.4926E-06
VEN	6.59183E-05	SIY	1.3561E-05	YED	3.4926E-06
VFC	6.59183E-05	SKG	1.3561E-05	YEN	3.4926E-06
WGW	6.59183E-05	SVN	1.3561E-05	YKM	3.4926E-06
WKP	6.59183E-05	TEN	1.3561E-05	YVY	3.4926E-06
WMG	6.59183E-05	TWQ	1.3561E-05	YYQ	3.4926E-06
WMV	6.59183E-05	VAC	1.3561E-05	YYY	3.4926E-06
YIG	6.59183E-05	VGY	1.3561E-05	AAN	3.24313E-06
YVI	6.59183E-05	VMY	1.3561E-05	ADC	3.24313E-06
ADH	6.54846E-05	WDV	1.3561E-05	AEE	3.24313E-06
ADQ	6.54846E-05	WHT	1.3561E-05	AEV	3.24313E-06
AMK	6.54846E-05	WMD	1.3561E-05	AII	3.24313E-06
AYK	6.54846E-05	WPW	1.3561E-05	AIN	3.24313E-06
CCA	6.54846E-05	WPY	1.3561E-05	AKG	3.24313E-06
EAE	6.54846E-05	WTY	1.3561E-05	AQC	3.24313E-06
GIQ	6.54846E-05	WVW	1.3561E-05	AYI	3.24313E-06
GKN	6.54846E-05	YLD	1.3561E-05	AYM	3.24313E-06
HKG	6.54846E-05	AEI	1.3204E-05	CDW	3.24313E-06
ICV	6.54846E-05	AGF	1.3204E-05	CFA	3.24313E-06
ITE	6.54846E-05	ATI	1.3204E-05	CFT	3.24313E-06
ITN	6.54846E-05	AYM	1.3204E-05	CHP	3.24313E-06
MAK	6.54846E-05	CDT	1.3204E-05	CLN	3.24313E-06
MDG	6.54846E-05	CFS	1.3204E-05	CPF	3.24313E-06
MRI	6.54846E-05	CKA	1.3204E-05	CPP	3.24313E-06
NGW	6.54846E-05	CSH	1.3204E-05	CSD	3.24313E-06
PKE	6.54846E-05	CSK	1.3204E-05	DDK	3.24313E-06
PMH	6.54846E-05	CTK	1.3204E-05	DGM	3.24313E-06
PMM	6.54846E-05	DGC	1.3204E-05	DHF	3.24313E-06
QFP	6.54846E-05	DKH	1.3204E-05	DHW	3.24313E-06
QTF	6.54846E-05	DLK	1.3204E-05	DRH	3.24313E-06
TNN	6.54846E-05	DLY	1.3204E-05	DVE	3.24313E-06
TQQ	6.54846E-05	DQF	1.3204E-05	DWW	3.24313E-06
VDI	6.54846E-05	EAF	1.3204E-05	ECL	3.24313E-06
VWN	6.54846E-05	EAW	1.3204E-05	ECR	3.24313E-06
VYK	6.54846E-05	EMV	1.3204E-05	EET	3.24313E-06
YGE	6.54846E-05	EWH	1.3204E-05	EGN	3.24313E-06
YQG	6.54846E-05	FMV	1.3204E-05	EGS	3.24313E-06
AIK	6.50509E-05	FTW	1.3204E-05	EHQ	3.24313E-06

AIQ	6.50509E-05	GFF	1.3204E-05	EIS	3.24313E-06
CNV	6.50509E-05	HNN	1.3204E-05	ELD	3.24313E-06
FFG	6.50509E-05	HVL	1.3204E-05	EMC	3.24313E-06
GCD	6.50509E-05	ICS	1.3204E-05	ERA	3.24313E-06
GEM	6.50509E-05	IHP	1.3204E-05	ERM	3.24313E-06
GFH	6.50509E-05	ING	1.3204E-05	ESK	3.24313E-06
GIF	6.50509E-05	INQ	1.3204E-05	EVH	3.24313E-06
GIH	6.50509E-05	IRC	1.3204E-05	EVS	3.24313E-06
HGF	6.50509E-05	KET	1.3204E-05	FDI	3.24313E-06
IDG	6.50509E-05	KEY	1.3204E-05	GCY	3.24313E-06
IVC	6.50509E-05	KKG	1.3204E-05	HCP	3.24313E-06
KGI	6.50509E-05	KNE	1.3204E-05	HDL	3.24313E-06
KYV	6.50509E-05	MGE	1.3204E-05	HFY	3.24313E-06
MIP	6.50509E-05	MPC	1.3204E-05	HGN	3.24313E-06
MVC	6.50509E-05	MPY	1.3204E-05	HHL	3.24313E-06
NAI	6.50509E-05	NCS	1.3204E-05	HKC	3.24313E-06
PCE	6.50509E-05	NKD	1.3204E-05	HKS	3.24313E-06
TCD	6.50509E-05	NMQ	1.3204E-05	HRS	3.24313E-06
TCQ	6.50509E-05	NSQ	1.3204E-05	HSK	3.24313E-06
TKQ	6.50509E-05	NVK	1.3204E-05	HWQ	3.24313E-06
VHC	6.50509E-05	PCC	1.3204E-05	HYD	3.24313E-06
VIN	6.50509E-05	PEN	1.3204E-05	IDW	3.24313E-06
VQC	6.50509E-05	PFI	1.3204E-05	IGA	3.24313E-06
WAY	6.50509E-05	PHY	1.3204E-05	INV	3.24313E-06
YAH	6.50509E-05	PKD	1.3204E-05	IQN	3.24313E-06
YPI	6.50509E-05	PKK	1.3204E-05	ISQ	3.24313E-06
ACH	6.46173E-05	PKV	1.3204E-05	ITV	3.24313E-06
AQK	6.46173E-05	PVN	1.3204E-05	IVV	3.24313E-06
AQW	6.46173E-05	PYW	1.3204E-05	KCQ	3.24313E-06
AWC	6.46173E-05	QAF	1.3204E-05	KHL	3.24313E-06
CAK	6.46173E-05	QAG	1.3204E-05	KHW	3.24313E-06
CHA	6.46173E-05	QCC	1.3204E-05	KLE	3.24313E-06
CTQ	6.46173E-05	QHT	1.3204E-05	KND	3.24313E-06
CVC	6.46173E-05	QND	1.3204E-05	KPN	3.24313E-06
CVK	6.46173E-05	QWT	1.3204E-05	KRN	3.24313E-06
EFP	6.46173E-05	SCH	1.3204E-05	KRP	3.24313E-06
EWA	6.46173E-05	SEW	1.3204E-05	KRQ	3.24313E-06
GWC	6.46173E-05	SHC	1.3204E-05	KSF	3.24313E-06
HGK	6.46173E-05	SHF	1.3204E-05	KVW	3.24313E-06
HWV	6.46173E-05	SVM	1.3204E-05	LAE	3.24313E-06
IAC	6.46173E-05	TEG	1.3204E-05	LPI	3.24313E-06
IET	6.46173E-05	TFQ	1.3204E-05	LQD	3.24313E-06
IFP	6.46173E-05	THQ	1.3204E-05	LVD	3.24313E-06
IIP	6.46173E-05	TME	1.3204E-05	LVF	3.24313E-06

IKP	6.46173E-05	VAH	1.3204E-05	MFG	3.24313E-06
ITF	6.46173E-05	VPN	1.3204E-05	MGS	3.24313E-06
ITI	6.46173E-05	VTV	1.3204E-05	MLQ	3.24313E-06
KFG	6.46173E-05	VVP	1.3204E-05	MMQ	3.24313E-06
KGY	6.46173E-05	WAF	1.3204E-05	MNC	3.24313E-06
MHV	6.46173E-05	WEH	1.3204E-05	MPT	3.24313E-06
PED	6.46173E-05	WIF	1.3204E-05	MSC	3.24313E-06
PIW	6.46173E-05	WYH	1.3204E-05	MSG	3.24313E-06
QPY	6.46173E-05	YCF	1.3204E-05	MVG	3.24313E-06
RCY	6.46173E-05	YDP	1.3204E-05	MVQ	3.24313E-06
TKF	6.46173E-05	YEG	1.3204E-05	NAG	3.24313E-06
TWW	6.46173E-05	YIL	1.3204E-05	NCS	3.24313E-06
VHK	6.46173E-05	YIR	1.3204E-05	NDA	3.24313E-06
VMC	6.46173E-05	YVI	1.3204E-05	NGG	3.24313E-06
VMD	6.46173E-05	AAM	1.2847E-05	NKV	3.24313E-06
VQN	6.46173E-05	AEE	1.2847E-05	NLY	3.24313E-06
VWH	6.46173E-05	CIT	1.2847E-05	NRC	3.24313E-06
VWQ	6.46173E-05	CRW	1.2847E-05	NRV	3.24313E-06
WKG	6.46173E-05	DFW	1.2847E-05	PAK	3.24313E-06
WKV	6.46173E-05	DKQ	1.2847E-05	PCI	3.24313E-06
WTF	6.46173E-05	DPD	1.2847E-05	PED	3.24313E-06
YTI	6.46173E-05	DTK	1.2847E-05	PET	3.24313E-06
ACK	6.41836E-05	EDD	1.2847E-05	QAD	3.24313E-06
ADI	6.41836E-05	EEF	1.2847E-05	QCK	3.24313E-06
AKY	6.41836E-05	EID	1.2847E-05	QFC	3.24313E-06
AWM	6.41836E-05	EKQ	1.2847E-05	QFG	3.24313E-06
CFP	6.41836E-05	EPD	1.2847E-05	QGG	3.24313E-06
CMA	6.41836E-05	EPV	1.2847E-05	QKF	3.24313E-06
CTK	6.41836E-05	FGF	1.2847E-05	QPF	3.24313E-06
CYA	6.41836E-05	FHV	1.2847E-05	QVG	3.24313E-06
DGH	6.41836E-05	FMG	1.2847E-05	QWE	3.24313E-06
EFG	6.41836E-05	FMQ	1.2847E-05	QYY	3.24313E-06
GNI	6.41836E-05	FWE	1.2847E-05	RCE	3.24313E-06
IAE	6.41836E-05	FWN	1.2847E-05	RDY	3.24313E-06
IFA	6.41836E-05	FYD	1.2847E-05	REF	3.24313E-06
ITK	6.41836E-05	GHR	1.2847E-05	RFK	3.24313E-06
IWT	6.41836E-05	GIM	1.2847E-05	RIW	3.24313E-06
MAC	6.41836E-05	GME	1.2847E-05	RVE	3.24313E-06
MDT	6.41836E-05	GQE	1.2847E-05	RVM	3.24313E-06
MPC	6.41836E-05	HCS	1.2847E-05	SCN	3.24313E-06
PHD	6.41836E-05	HFM	1.2847E-05	SDD	3.24313E-06
PMN	6.41836E-05	IET	1.2847E-05	SDV	3.24313E-06
PNM	6.41836E-05	IHV	1.2847E-05	SEF	3.24313E-06
PWM	6.41836E-05	ITF	1.2847E-05	SHF	3.24313E-06

QAI	6.41836E-05	IYM	1.2847E-05	STE	3.24313E-06
RCF	6.41836E-05	KHN	1.2847E-05	TET	3.24313E-06
RWI	6.41836E-05	KKF	1.2847E-05	TIG	3.24313E-06
TKI	6.41836E-05	KME	1.2847E-05	TKA	3.24313E-06
VCF	6.41836E-05	KYK	1.2847E-05	TKG	3.24313E-06
VDD	6.41836E-05	MEA	1.2847E-05	TMK	3.24313E-06
VEE	6.41836E-05	MHQ	1.2847E-05	TPE	3.24313E-06
VMF	6.41836E-05	MLN	1.2847E-05	TPN	3.24313E-06
VNF	6.41836E-05	MSC	1.2847E-05	TRF	3.24313E-06
VQW	6.41836E-05	MSM	1.2847E-05	TRN	3.24313E-06
VYN	6.41836E-05	MTH	1.2847E-05	TRW	3.24313E-06
WAH	6.41836E-05	NCW	1.2847E-05	TSK	3.24313E-06
WHT	6.41836E-05	NDD	1.2847E-05	TTG	3.24313E-06
WYV	6.41836E-05	NQT	1.2847E-05	TVC	3.24313E-06
YGC	6.41836E-05	PCH	1.2847E-05	TWW	3.24313E-06
YKG	6.41836E-05	PCQ	1.2847E-05	TYR	3.24313E-06
AHK	6.37499E-05	PDN	1.2847E-05	VAA	3.24313E-06
AKF	6.37499E-05	PEV	1.2847E-05	VEE	3.24313E-06
AKK	6.37499E-05	PHN	1.2847E-05	VEN	3.24313E-06
CPD	6.37499E-05	PIE	1.2847E-05	VFA	3.24313E-06
EIG	6.37499E-05	PIW	1.2847E-05	VIM	3.24313E-06
FAD	6.37499E-05	PNI	1.2847E-05	VMP	3.24313E-06
FEG	6.37499E-05	PVD	1.2847E-05	VNM	3.24313E-06
HGY	6.37499E-05	QCI	1.2847E-05	VQG	3.24313E-06
IHG	6.37499E-05	QEH	1.2847E-05	WFE	3.24313E-06
IVY	6.37499E-05	QHW	1.2847E-05	WIP	3.24313E-06
IYP	6.37499E-05	QQA	1.2847E-05	WKK	3.24313E-06
MET	6.37499E-05	QQL	1.2847E-05	WMN	3.24313E-06
MQT	6.37499E-05	RMV	1.2847E-05	WQE	3.24313E-06
MYT	6.37499E-05	SFE	1.2847E-05	WTI	3.24313E-06
PIH	6.37499E-05	SKD	1.2847E-05	WYI	3.24313E-06
QAE	6.37499E-05	SWW	1.2847E-05	YAN	3.24313E-06
QGE	6.37499E-05	TED	1.2847E-05	YCA	3.24313E-06
QGQ	6.37499E-05	THF	1.2847E-05	YCE	3.24313E-06
TCK	6.37499E-05	TIF	1.2847E-05	YHF	3.24313E-06
TDF	6.37499E-05	TVM	1.2847E-05	YKN	3.24313E-06
TEK	6.37499E-05	VKY	1.2847E-05	YWE	3.24313E-06
TFW	6.37499E-05	VLD	1.2847E-05	ASE	2.99365E-06
TND	6.37499E-05	VSI	1.2847E-05	AYC	2.99365E-06
TQC	6.37499E-05	WDP	1.2847E-05	AYV	2.99365E-06
TQH	6.37499E-05	WIH	1.2847E-05	CAP	2.99365E-06
TWK	6.37499E-05	WKG	1.2847E-05	CAS	2.99365E-06
VFK	6.37499E-05	YCW	1.2847E-05	CDQ	2.99365E-06
WAQ	6.37499E-05	YET	1.2847E-05	CDR	2.99365E-06

WQA	6.37499E-05	YGI	1.2847E-05	CGV	2.99365E-06
ANC	6.33163E-05	YPI	1.2847E-05	CHN	2.99365E-06
CAI	6.33163E-05	YRQ	1.2847E-05	CIL	2.99365E-06
CGC	6.33163E-05	ACC	1.249E-05	CPD	2.99365E-06
CKP	6.33163E-05	ADD	1.249E-05	CPM	2.99365E-06
CPK	6.33163E-05	AKM	1.249E-05	CQP	2.99365E-06
EGN	6.33163E-05	AQM	1.249E-05	CQW	2.99365E-06
EGY	6.33163E-05	AYC	1.249E-05	CVW	2.99365E-06
EPY	6.33163E-05	CLN	1.249E-05	DAF	2.99365E-06
EVI	6.33163E-05	CQR	1.249E-05	DDY	2.99365E-06
GQW	6.33163E-05	DAW	1.249E-05	DGA	2.99365E-06
GYQ	6.33163E-05	DDP	1.249E-05	DGF	2.99365E-06
IQA	6.33163E-05	DMV	1.249E-05	DKK	2.99365E-06
ITD	6.33163E-05	DRI	1.249E-05	DLH	2.99365E-06
IVE	6.33163E-05	DWT	1.249E-05	DQK	2.99365E-06
KTI	6.33163E-05	EHM	1.249E-05	DWS	2.99365E-06
MHT	6.33163E-05	EHT	1.249E-05	DYW	2.99365E-06
MMG	6.33163E-05	EMC	1.249E-05	EAE	2.99365E-06
MTC	6.33163E-05	EQM	1.249E-05	ECA	2.99365E-06
MVN	6.33163E-05	FHI	1.249E-05	EGP	2.99365E-06
PCC	6.33163E-05	FLE	1.249E-05	EVM	2.99365E-06
PCD	6.33163E-05	GII	1.249E-05	EWP	2.99365E-06
PFC	6.33163E-05	HCH	1.249E-05	FAE	2.99365E-06
PHE	6.33163E-05	HRN	1.249E-05	FDH	2.99365E-06
PYE	6.33163E-05	HTM	1.249E-05	FEC	2.99365E-06
QQG	6.33163E-05	ITE	1.249E-05	FHI	2.99365E-06
TFH	6.33163E-05	IVV	1.249E-05	FKE	2.99365E-06
TNI	6.33163E-05	IWP	1.249E-05	FNK	2.99365E-06
VCC	6.33163E-05	KHH	1.249E-05	FQD	2.99365E-06
VQK	6.33163E-05	KHP	1.249E-05	FQK	2.99365E-06
VWK	6.33163E-05	LIN	1.249E-05	FQN	2.99365E-06
WTK	6.33163E-05	LVY	1.249E-05	FQY	2.99365E-06
YWV	6.33163E-05	MCE	1.249E-05	FVC	2.99365E-06
ANI	6.28826E-05	MCP	1.249E-05	FVI	2.99365E-06
CPI	6.28826E-05	MNV	1.249E-05	FYD	2.99365E-06
DWV	6.28826E-05	MTG	1.249E-05	FYE	2.99365E-06
EEV	6.28826E-05	MVI	1.249E-05	GAI	2.99365E-06
EYV	6.28826E-05	QCH	1.249E-05	GCQ	2.99365E-06
GED	6.28826E-05	QLY	1.249E-05	GIM	2.99365E-06
GMQ	6.28826E-05	QPI	1.249E-05	GIP	2.99365E-06
IEP	6.28826E-05	RGM	1.249E-05	GPW	2.99365E-06
IKA	6.28826E-05	TCK	1.249E-05	GQW	2.99365E-06
IPD	6.28826E-05	TCN	1.249E-05	GWK	2.99365E-06
IQP	6.28826E-05	TIC	1.249E-05	HAN	2.99365E-06

KPI	6.28826E-05	TKN	1.249E-05	HEE	2.99365E-06
KYG	6.28826E-05	TPI	1.249E-05	HFA	2.99365E-06
MQP	6.28826E-05	TYN	1.249E-05	HHH	2.99365E-06
MTY	6.28826E-05	VHR	1.249E-05	HKG	2.99365E-06
MVI	6.28826E-05	VKN	1.249E-05	HLI	2.99365E-06
PEW	6.28826E-05	VTW	1.249E-05	HPN	2.99365E-06
PMK	6.28826E-05	WHA	1.249E-05	HPW	2.99365E-06
PWW	6.28826E-05	WPC	1.249E-05	HRT	2.99365E-06
QIG	6.28826E-05	WQR	1.249E-05	HSQ	2.99365E-06
SWY	6.28826E-05	WQT	1.249E-05	HVS	2.99365E-06
TDN	6.28826E-05	WSM	1.249E-05	HWG	2.99365E-06
TDQ	6.28826E-05	YHI	1.249E-05	IAP	2.99365E-06
THY	6.28826E-05	YKV	1.249E-05	ICV	2.99365E-06
TKC	6.28826E-05	YMP	1.249E-05	IDH	2.99365E-06
TQN	6.28826E-05	YRM	1.249E-05	IEY	2.99365E-06
TWC	6.28826E-05	ACQ	1.2133E-05	IFM	2.99365E-06
TYE	6.28826E-05	ADE	1.2133E-05	IGG	2.99365E-06
VHE	6.28826E-05	AEN	1.2133E-05	IGK	2.99365E-06
WEG	6.28826E-05	AEV	1.2133E-05	III	2.99365E-06
WIV	6.28826E-05	AIG	1.2133E-05	IMD	2.99365E-06
WNV	6.28826E-05	AKV	1.2133E-05	IND	2.99365E-06
WVQ	6.28826E-05	AMD	1.2133E-05	INK	2.99365E-06
YGD	6.28826E-05	AVV	1.2133E-05	IPQ	2.99365E-06
YGH	6.28826E-05	AYR	1.2133E-05	IRC	2.99365E-06
YQV	6.28826E-05	CCD	1.2133E-05	IRF	2.99365E-06
AHH	6.24489E-05	CHA	1.2133E-05	IYI	2.99365E-06
AHN	6.24489E-05	CMW	1.2133E-05	KAK	2.99365E-06
AKE	6.24489E-05	CPQ	1.2133E-05	KCA	2.99365E-06
CAH	6.24489E-05	CRF	1.2133E-05	KCR	2.99365E-06
CVF	6.24489E-05	DIL	1.2133E-05	KCT	2.99365E-06
CYV	6.24489E-05	DMF	1.2133E-05	KGG	2.99365E-06
EHV	6.24489E-05	DPY	1.2133E-05	KGY	2.99365E-06
GCH	6.24489E-05	DTG	1.2133E-05	KMH	2.99365E-06
GEN	6.24489E-05	DTI	1.2133E-05	KNV	2.99365E-06
GHC	6.24489E-05	EGG	1.2133E-05	KQQ	2.99365E-06
GKD	6.24489E-05	EHQ	1.2133E-05	KSK	2.99365E-06
IGF	6.24489E-05	EWF	1.2133E-05	KYC	2.99365E-06
ING	6.24489E-05	EWI	1.2133E-05	LIE	2.99365E-06
IVD	6.24489E-05	FFQ	1.2133E-05	LIM	2.99365E-06
IVI	6.24489E-05	FQF	1.2133E-05	LYI	2.99365E-06
KGF	6.24489E-05	FVG	1.2133E-05	MCK	2.99365E-06
MDP	6.24489E-05	GMW	1.2133E-05	MCP	2.99365E-06
MVK	6.24489E-05	GWQ	1.2133E-05	MFS	2.99365E-06
PIF	6.24489E-05	HAQ	1.2133E-05	MGN	2.99365E-06

QDV	6.24489E-05	HEP	1.2133E-05	MKY	2.99365E-06
QVE	6.24489E-05	HGM	1.2133E-05	MPQ	2.99365E-06
QYA	6.24489E-05	HQA	1.2133E-05	MSA	2.99365E-06
TDH	6.24489E-05	HYL	1.2133E-05	MSH	2.99365E-06
TEC	6.24489E-05	IID	1.2133E-05	MSI	2.99365E-06
TFC	6.24489E-05	IKM	1.2133E-05	MVT	2.99365E-06
TKK	6.24489E-05	ILF	1.2133E-05	MWI	2.99365E-06
TQD	6.24489E-05	ITG	1.2133E-05	MYA	2.99365E-06
TQM	6.24489E-05	KEG	1.2133E-05	NCV	2.99365E-06
VEH	6.24489E-05	KGM	1.2133E-05	NFE	2.99365E-06
VHD	6.24489E-05	KRI	1.2133E-05	NGC	2.99365E-06
WTD	6.24489E-05	LED	1.2133E-05	NGE	2.99365E-06
YWA	6.24489E-05	LHW	1.2133E-05	NGK	2.99365E-06
ADE	6.20152E-05	MFN	1.2133E-05	NGY	2.99365E-06
AHD	6.20152E-05	NED	1.2133E-05	NKW	2.99365E-06
AIW	6.20152E-05	NMW	1.2133E-05	NNI	2.99365E-06
AQN	6.20152E-05	NRE	1.2133E-05	NSQ	2.99365E-06
CHV	6.20152E-05	NYH	1.2133E-05	NVA	2.99365E-06
CIV	6.20152E-05	PKI	1.2133E-05	NWN	2.99365E-06
CPE	6.20152E-05	PMK	1.2133E-05	PAF	2.99365E-06
CTF	6.20152E-05	PYQ	1.2133E-05	PCM	2.99365E-06
CTI	6.20152E-05	QGC	1.2133E-05	PFK	2.99365E-06
DWG	6.20152E-05	QKG	1.2133E-05	PGE	2.99365E-06
FPI	6.20152E-05	QKH	1.2133E-05	PHF	2.99365E-06
GCF	6.20152E-05	QKY	1.2133E-05	PHN	2.99365E-06
IWA	6.20152E-05	QMH	1.2133E-05	PKF	2.99365E-06
KWA	6.20152E-05	QVM	1.2133E-05	PKI	2.99365E-06
MDV	6.20152E-05	QVR	1.2133E-05	PQN	2.99365E-06
MPN	6.20152E-05	QWA	1.2133E-05	QDT	2.99365E-06
MTK	6.20152E-05	QYK	1.2133E-05	QFY	2.99365E-06
MWP	6.20152E-05	RHF	1.2133E-05	QGC	2.99365E-06
NFC	6.20152E-05	RQH	1.2133E-05	QHN	2.99365E-06
PDK	6.20152E-05	RQQ	1.2133E-05	QLC	2.99365E-06
PNY	6.20152E-05	SCC	1.2133E-05	QPE	2.99365E-06
PYW	6.20152E-05	SIW	1.2133E-05	QTQ	2.99365E-06
QVD	6.20152E-05	SQQ	1.2133E-05	QVH	2.99365E-06
TEE	6.20152E-05	STW	1.2133E-05	QWA	2.99365E-06
TII	6.20152E-05	TMP	1.2133E-05	RCI	2.99365E-06
TQE	6.20152E-05	TYH	1.2133E-05	RGV	2.99365E-06
VEC	6.20152E-05	VGC	1.2133E-05	RHI	2.99365E-06
VQE	6.20152E-05	VNI	1.2133E-05	RKE	2.99365E-06
VWC	6.20152E-05	WEC	1.2133E-05	RMV	2.99365E-06
WEP	6.20152E-05	WMA	1.2133E-05	RNK	2.99365E-06
WIG	6.20152E-05	WRE	1.2133E-05	RQI	2.99365E-06

ADN	6.15816E-05	YAP	1.2133E-05	RTM	2.99365E-06
AIH	6.15816E-05	YCV	1.2133E-05	SEE	2.99365E-06
AMN	6.15816E-05	YGN	1.2133E-05	SEK	2.99365E-06
AYM	6.15816E-05	YPV	1.2133E-05	SFI	2.99365E-06
CDG	6.15816E-05	AAC	1.1776E-05	SIN	2.99365E-06
CWT	6.15816E-05	ACN	1.1776E-05	SMI	2.99365E-06
EAI	6.15816E-05	ADH	1.1776E-05	SVK	2.99365E-06
EAY	6.15816E-05	ADN	1.1776E-05	SWN	2.99365E-06
FAI	6.15816E-05	ADW	1.1776E-05	TCA	2.99365E-06
HAI	6.15816E-05	AFH	1.1776E-05	TCT	2.99365E-06
IGQ	6.15816E-05	AGY	1.1776E-05	TDR	2.99365E-06
IPF	6.15816E-05	AQV	1.1776E-05	TFV	2.99365E-06
ITY	6.15816E-05	AVC	1.1776E-05	TIH	2.99365E-06
MAE	6.15816E-05	AWI	1.1776E-05	TMV	2.99365E-06
MFA	6.15816E-05	AYN	1.1776E-05	TQD	2.99365E-06
MNV	6.15816E-05	CIR	1.1776E-05	TVN	2.99365E-06
MTQ	6.15816E-05	CMR	1.1776E-05	TVP	2.99365E-06
PIY	6.15816E-05	CNP	1.1776E-05	TWA	2.99365E-06
PMD	6.15816E-05	CPC	1.1776E-05	TWN	2.99365E-06
PNI	6.15816E-05	CRA	1.1776E-05	VCY	2.99365E-06
QKG	6.15816E-05	CVT	1.1776E-05	VDA	2.99365E-06
TFE	6.15816E-05	DDG	1.1776E-05	VFW	2.99365E-06
THK	6.15816E-05	DDR	1.1776E-05	VIC	2.99365E-06
VEQ	6.15816E-05	DFM	1.1776E-05	VNQ	2.99365E-06
VIE	6.15816E-05	DGG	1.1776E-05	VQW	2.99365E-06
VWF	6.15816E-05	DHV	1.1776E-05	WDN	2.99365E-06
WPM	6.15816E-05	DIV	1.1776E-05	WGI	2.99365E-06
WQG	6.15816E-05	DQH	1.1776E-05	WHI	2.99365E-06
WVI	6.15816E-05	DQV	1.1776E-05	WIW	2.99365E-06
ACM	6.11479E-05	DRK	1.1776E-05	WQK	2.99365E-06
AHE	6.11479E-05	EEC	1.1776E-05	YCM	2.99365E-06
ANF	6.11479E-05	EFA	1.1776E-05	YCY	2.99365E-06
FAE	6.11479E-05	ELE	1.1776E-05	YIW	2.99365E-06
GDE	6.11479E-05	EMN	1.1776E-05	YKE	2.99365E-06
GEC	6.11479E-05	ENK	1.1776E-05	YMD	2.99365E-06
GHW	6.11479E-05	ETV	1.1776E-05	YMM	2.99365E-06
GKI	6.11479E-05	EYQ	1.1776E-05	YMN	2.99365E-06
GMI	6.11479E-05	FHR	1.1776E-05	YVP	2.99365E-06
GYD	6.11479E-05	FKN	1.1776E-05	YWN	2.99365E-06
GYH	6.11479E-05	GQM	1.1776E-05	ACF	2.74418E-06
ICG	6.11479E-05	GQQ	1.1776E-05	ADG	2.74418E-06
IHV	6.11479E-05	HER	1.1776E-05	AEC	2.74418E-06
IVH	6.11479E-05	HIT	1.1776E-05	AGY	2.74418E-06
MKV	6.11479E-05	HLC	1.1776E-05	AHN	2.74418E-06

MTI	6.11479E-05	HSE	1.1776E-05	AMA	2.74418E-06
PKK	6.11479E-05	HVR	1.1776E-05	AMF	2.74418E-06
PYN	6.11479E-05	IHH	1.1776E-05	ANG	2.74418E-06
QCV	6.11479E-05	IQA	1.1776E-05	AVD	2.74418E-06
QEV	6.11479E-05	IQD	1.1776E-05	AYG	2.74418E-06
QFV	6.11479E-05	IVS	1.1776E-05	CAD	2.74418E-06
TCE	6.11479E-05	IWW	1.1776E-05	CAG	2.74418E-06
TEH	6.11479E-05	KDV	1.1776E-05	CAT	2.74418E-06
TEW	6.11479E-05	KYW	1.1776E-05	CDL	2.74418E-06
TID	6.11479E-05	LIY	1.1776E-05	CGS	2.74418E-06
TQW	6.11479E-05	MCH	1.1776E-05	CGT	2.74418E-06
TYC	6.11479E-05	MFK	1.1776E-05	CKT	2.74418E-06
VCE	6.11479E-05	MLW	1.1776E-05	CMT	2.74418E-06
VYY	6.11479E-05	MNC	1.1776E-05	CQC	2.74418E-06
WVY	6.11479E-05	MVG	1.1776E-05	CTT	2.74418E-06
AFC	6.07142E-05	MVS	1.1776E-05	CVN	2.74418E-06
AME	6.07142E-05	NCF	1.1776E-05	CWK	2.74418E-06
AQI	6.07142E-05	NEY	1.1776E-05	DAA	2.74418E-06
AYQ	6.07142E-05	PEG	1.1776E-05	DCG	2.74418E-06
CDA	6.07142E-05	PMF	1.1776E-05	DFM	2.74418E-06
CPH	6.07142E-05	QAP	1.1776E-05	DGE	2.74418E-06
CTE	6.07142E-05	QGW	1.1776E-05	DIT	2.74418E-06
FGE	6.07142E-05	QKA	1.1776E-05	DKA	2.74418E-06
FKG	6.07142E-05	QML	1.1776E-05	DLD	2.74418E-06
GKK	6.07142E-05	QWR	1.1776E-05	DLY	2.74418E-06
GMD	6.07142E-05	REM	1.1776E-05	DRD	2.74418E-06
GMH	6.07142E-05	RIW	1.1776E-05	DRS	2.74418E-06
HEG	6.07142E-05	RYE	1.1776E-05	DRW	2.74418E-06
IAY	6.07142E-05	SEE	1.1776E-05	DWM	2.74418E-06
IPK	6.07142E-05	SEG	1.1776E-05	DYE	2.74418E-06
MKA	6.07142E-05	SNE	1.1776E-05	DYF	2.74418E-06
MVM	6.07142E-05	TFI	1.1776E-05	EIL	2.74418E-06
PID	6.07142E-05	TKK	1.1776E-05	EKK	2.74418E-06
PIM	6.07142E-05	VDR	1.1776E-05	ELW	2.74418E-06
QGF	6.07142E-05	VNK	1.1776E-05	EMH	2.74418E-06
QHV	6.07142E-05	VNQ	1.1776E-05	EPQ	2.74418E-06
QPI	6.07142E-05	VWG	1.1776E-05	EQC	2.74418E-06
TDI	6.07142E-05	VYG	1.1776E-05	EQE	2.74418E-06
TEQ	6.07142E-05	WKV	1.1776E-05	EQK	2.74418E-06
VCI	6.07142E-05	WPQ	1.1776E-05	ERQ	2.74418E-06
VDN	6.07142E-05	WVK	1.1776E-05	EVW	2.74418E-06
WFV	6.07142E-05	YGD	1.1776E-05	EWA	2.74418E-06
WPQ	6.07142E-05	YGE	1.1776E-05	EYW	2.74418E-06
WTI	6.07142E-05	YHT	1.1776E-05	EYY	2.74418E-06

ADF	6.02805E-05	YTM	1.1776E-05	FIF	2.74418E-06
AEW	6.02805E-05	YYG	1.1776E-05	FIN	2.74418E-06
AFQ	6.02805E-05	AFM	1.142E-05	FIP	2.74418E-06
AQC	6.02805E-05	AFN	1.142E-05	FPK	2.74418E-06
CAF	6.02805E-05	AMM	1.142E-05	FYK	2.74418E-06
CHP	6.02805E-05	AVN	1.142E-05	FYY	2.74418E-06
DAI	6.02805E-05	AYD	1.142E-05	GCI	2.74418E-06
DGD	6.02805E-05	CDS	1.142E-05	GCM	2.74418E-06
ETQ	6.02805E-05	CPK	1.142E-05	GDK	2.74418E-06
GHK	6.02805E-05	CPV	1.142E-05	GFW	2.74418E-06
GNE	6.02805E-05	DAA	1.142E-05	GWY	2.74418E-06
IDV	6.02805E-05	DCF	1.142E-05	HED	2.74418E-06
PFE	6.02805E-05	DMR	1.142E-05	HLH	2.74418E-06
PQD	6.02805E-05	DNR	1.142E-05	HLQ	2.74418E-06
QCG	6.02805E-05	DTC	1.142E-05	HRN	2.74418E-06
QEA	6.02805E-05	DVK	1.142E-05	HSM	2.74418E-06
WGD	6.02805E-05	ECF	1.142E-05	HTF	2.74418E-06
WGM	6.02805E-05	EHR	1.142E-05	HTK	2.74418E-06
YDM	6.02805E-05	EHV	1.142E-05	HVR	2.74418E-06
ACE	5.98469E-05	EIC	1.142E-05	HWF	2.74418E-06
ADD	5.98469E-05	ELK	1.142E-05	HWH	2.74418E-06
AEN	5.98469E-05	ENC	1.142E-05	HWN	2.74418E-06
AHI	5.98469E-05	FCH	1.142E-05	HWP	2.74418E-06
AIE	5.98469E-05	FEM	1.142E-05	IDL	2.74418E-06
CPQ	5.98469E-05	FVI	1.142E-05	IHN	2.74418E-06
CPY	5.98469E-05	GKE	1.142E-05	IME	2.74418E-06
CWV	5.98469E-05	HAW	1.142E-05	IQP	2.74418E-06
EAK	5.98469E-05	HCE	1.142E-05	ITD	2.74418E-06
ECG	5.98469E-05	HFQ	1.142E-05	KEE	2.74418E-06
EVY	5.98469E-05	HGP	1.142E-05	KGA	2.74418E-06
GMW	5.98469E-05	HIA	1.142E-05	KGE	2.74418E-06
GWQ	5.98469E-05	HMT	1.142E-05	KKF	2.74418E-06
HWG	5.98469E-05	HPC	1.142E-05	KLW	2.74418E-06
IGE	5.98469E-05	HQR	1.142E-05	KMM	2.74418E-06
IVQ	5.98469E-05	HQT	1.142E-05	KSM	2.74418E-06
KAI	5.98469E-05	HWC	1.142E-05	KTK	2.74418E-06
KNW	5.98469E-05	IHY	1.142E-05	KVC	2.74418E-06
MQA	5.98469E-05	IQG	1.142E-05	KYW	2.74418E-06
PCK	5.98469E-05	KMD	1.142E-05	LED	2.74418E-06
PEI	5.98469E-05	KQN	1.142E-05	LMI	2.74418E-06
PFI	5.98469E-05	KSM	1.142E-05	LQK	2.74418E-06
PFK	5.98469E-05	KVD	1.142E-05	MAH	2.74418E-06
QGH	5.98469E-05	MEH	1.142E-05	MDT	2.74418E-06
TFK	5.98469E-05	NAF	1.142E-05	MFN	2.74418E-06

VFH	5.98469E-05	NCN	1.142E-05	MGP	2.74418E-06
VHY	5.98469E-05	NDQ	1.142E-05	MMY	2.74418E-06
VIF	5.98469E-05	NIE	1.142E-05	MPW	2.74418E-06
VKN	5.98469E-05	NKF	1.142E-05	MQA	2.74418E-06
VQM	5.98469E-05	NTE	1.142E-05	MRK	2.74418E-06
WVF	5.98469E-05	PDF	1.142E-05	MRM	2.74418E-06
YYQ	5.98469E-05	PQG	1.142E-05	MSM	2.74418E-06
AFH	5.94132E-05	QCT	1.142E-05	MTK	2.74418E-06
AMH	5.94132E-05	QHF	1.142E-05	MVL	2.74418E-06
CEA	5.94132E-05	QHP	1.142E-05	MWA	2.74418E-06
CQA	5.94132E-05	QMY	1.142E-05	NAK	2.74418E-06
EMG	5.94132E-05	QRM	1.142E-05	NDL	2.74418E-06
GDF	5.94132E-05	QVH	1.142E-05	NFI	2.74418E-06
GFQ	5.94132E-05	SEI	1.142E-05	NIA	2.74418E-06
GQD	5.94132E-05	SYH	1.142E-05	NNQ	2.74418E-06
GQE	5.94132E-05	TKC	1.142E-05	NQE	2.74418E-06
KDC	5.94132E-05	TKG	1.142E-05	NTM	2.74418E-06
KWV	5.94132E-05	TNI	1.142E-05	PEG	2.74418E-06
MWT	5.94132E-05	VEG	1.142E-05	PFM	2.74418E-06
TCW	5.94132E-05	VPD	1.142E-05	PFQ	2.74418E-06
TED	5.94132E-05	VTM	1.142E-05	PFV	2.74418E-06
TME	5.94132E-05	WCT	1.142E-05	PGK	2.74418E-06
TYH	5.94132E-05	WDI	1.142E-05	PII	2.74418E-06
VED	5.94132E-05	WHD	1.142E-05	PIM	2.74418E-06
VEK	5.94132E-05	WLE	1.142E-05	PKE	2.74418E-06
VIH	5.94132E-05	WPN	1.142E-05	PMP	2.74418E-06
VNY	5.94132E-05	WTM	1.142E-05	PNE	2.74418E-06
WAN	5.94132E-05	YDD	1.142E-05	PNI	2.74418E-06
WEV	5.94132E-05	YGY	1.142E-05	PVG	2.74418E-06
WFG	5.94132E-05	YHW	1.142E-05	PWF	2.74418E-06
WHH	5.94132E-05	YMF	1.142E-05	QDK	2.74418E-06
WPE	5.94132E-05	YMR	1.142E-05	QDV	2.74418E-06
CMG	5.89795E-05	YMY	1.142E-05	QFD	2.74418E-06
EGC	5.89795E-05	YPD	1.142E-05	QFH	2.74418E-06
EPF	5.89795E-05	YWT	1.142E-05	QFP	2.74418E-06
EVE	5.89795E-05	YYT	1.142E-05	QGY	2.74418E-06
GQC	5.89795E-05	ADF	1.1063E-05	QIT	2.74418E-06
GYN	5.89795E-05	ADM	1.1063E-05	QKN	2.74418E-06
IAK	5.89795E-05	AIE	1.1063E-05	QLW	2.74418E-06
IGW	5.89795E-05	AIF	1.1063E-05	QPV	2.74418E-06
MAI	5.89795E-05	AQD	1.1063E-05	QPW	2.74418E-06
MGW	5.89795E-05	AVK	1.1063E-05	QTI	2.74418E-06
MKG	5.89795E-05	AWH	1.1063E-05	QTV	2.74418E-06
PEK	5.89795E-05	CDH	1.1063E-05	QWC	2.74418E-06

PFH	5.89795E-05	CFR	1.1063E-05	QWK	2.74418E-06
PHF	5.89795E-05	CLD	1.1063E-05	QWV	2.74418E-06
PKN	5.89795E-05	CMP	1.1063E-05	RIV	2.74418E-06
PME	5.89795E-05	CPH	1.1063E-05	RMI	2.74418E-06
PMI	5.89795E-05	DFH	1.1063E-05	RMM	2.74418E-06
PWK	5.89795E-05	DHT	1.1063E-05	RQE	2.74418E-06
TIC	5.89795E-05	DKF	1.1063E-05	RVI	2.74418E-06
VMY	5.89795E-05	DMG	1.1063E-05	SCQ	2.74418E-06
WPK	5.89795E-05	DPI	1.1063E-05	SDY	2.74418E-06
AFK	5.85459E-05	DRM	1.1063E-05	SEV	2.74418E-06
AMF	5.85459E-05	DVT	1.1063E-05	SFN	2.74418E-06
CAN	5.85459E-05	DVW	1.1063E-05	SII	2.74418E-06
CIG	5.85459E-05	DYH	1.1063E-05	SVI	2.74418E-06
CYG	5.85459E-05	ECS	1.1063E-05	SYQ	2.74418E-06
DIG	5.85459E-05	EEM	1.1063E-05	TAE	2.74418E-06
EWG	5.85459E-05	EIH	1.1063E-05	TEY	2.74418E-06
FYG	5.85459E-05	EMD	1.1063E-05	TGC	2.74418E-06
HDC	5.85459E-05	EVM	1.1063E-05	TKD	2.74418E-06
MCP	5.85459E-05	FAE	1.1063E-05	TNC	2.74418E-06
MGH	5.85459E-05	FEH	1.1063E-05	TNF	2.74418E-06
PFD	5.85459E-05	FKD	1.1063E-05	TPQ	2.74418E-06
PWE	5.85459E-05	FKE	1.1063E-05	TRC	2.74418E-06
QPF	5.85459E-05	FPC	1.1063E-05	TRI	2.74418E-06
TFN	5.85459E-05	FPW	1.1063E-05	TRY	2.74418E-06
VID	5.85459E-05	GFN	1.1063E-05	TTW	2.74418E-06
VKC	5.85459E-05	GHY	1.1063E-05	TWD	2.74418E-06
VKF	5.85459E-05	GMC	1.1063E-05	TYP	2.74418E-06
VWY	5.85459E-05	GYY	1.1063E-05	VCK	2.74418E-06
WGC	5.85459E-05	HEL	1.1063E-05	VDM	2.74418E-06
WTY	5.85459E-05	HMR	1.1063E-05	VHI	2.74418E-06
AEE	5.81122E-05	HNE	1.1063E-05	VIG	2.74418E-06
AEK	5.81122E-05	HQP	1.1063E-05	VIV	2.74418E-06
AMI	5.81122E-05	HTE	1.1063E-05	VKE	2.74418E-06
AYN	5.81122E-05	IAM	1.1063E-05	VPI	2.74418E-06
CPN	5.81122E-05	IEA	1.1063E-05	VPK	2.74418E-06
FYM	5.81122E-05	IPF	1.1063E-05	VQN	2.74418E-06
GIK	5.81122E-05	KCI	1.1063E-05	VVF	2.74418E-06
GYW	5.81122E-05	KCY	1.1063E-05	VVI	2.74418E-06
IGC	5.81122E-05	KGK	1.1063E-05	WTM	2.74418E-06
IGY	5.81122E-05	KKC	1.1063E-05	WYM	2.74418E-06
IKG	5.81122E-05	KMF	1.1063E-05	YAV	2.74418E-06
MGC	5.81122E-05	KVE	1.1063E-05	YEK	2.74418E-06
MGD	5.81122E-05	KYV	1.1063E-05	YFY	2.74418E-06
MGF	5.81122E-05	LEH	1.1063E-05	YGK	2.74418E-06

MTF	5.81122E-05	LEV	1.1063E-05	YHK	2.74418E-06
NMW	5.81122E-05	LQY	1.1063E-05	YVV	2.74418E-06
PDH	5.81122E-05	LVQ	1.1063E-05	YWD	2.74418E-06
PIK	5.81122E-05	MAQ	1.1063E-05	YWY	2.74418E-06
PQI	5.81122E-05	MIA	1.1063E-05	ACD	2.49471E-06
QGY	5.81122E-05	MKD	1.1063E-05	ACY	2.49471E-06
QIV	5.81122E-05	MKN	1.1063E-05	ADV	2.49471E-06
QYG	5.81122E-05	MMG	1.1063E-05	ADY	2.49471E-06
TDE	5.81122E-05	MPN	1.1063E-05	AGD	2.49471E-06
TFD	5.81122E-05	MQP	1.1063E-05	AGN	2.49471E-06
TMF	5.81122E-05	MVL	1.1063E-05	AMG	2.49471E-06
TMH	5.81122E-05	MWS	1.1063E-05	AMW	2.49471E-06
TNF	5.81122E-05	NCD	1.1063E-05	AVN	2.49471E-06
TYD	5.81122E-05	NID	1.1063E-05	AWC	2.49471E-06
VDY	5.81122E-05	NVE	1.1063E-05	AWN	2.49471E-06
VIC	5.81122E-05	NVY	1.1063E-05	CEP	2.49471E-06
VIW	5.81122E-05	NYV	1.1063E-05	CKP	2.49471E-06
VWD	5.81122E-05	PQQ	1.1063E-05	CLY	2.49471E-06
VWI	5.81122E-05	QHG	1.1063E-05	CMP	2.49471E-06
YMC	5.81122E-05	QIF	1.1063E-05	CRE	2.49471E-06
ACQ	5.76785E-05	QIL	1.1063E-05	CTD	2.49471E-06
AIN	5.76785E-05	QMP	1.1063E-05	CWC	2.49471E-06
ANY	5.76785E-05	QPE	1.1063E-05	DAE	2.49471E-06
AQE	5.76785E-05	RIF	1.1063E-05	DCV	2.49471E-06
CGW	5.76785E-05	SCF	1.1063E-05	DDF	2.49471E-06
CNG	5.76785E-05	SHM	1.1063E-05	DGK	2.49471E-06
FPY	5.76785E-05	SKN	1.1063E-05	DIL	2.49471E-06
GFD	5.76785E-05	SMK	1.1063E-05	DKF	2.49471E-06
IAW	5.76785E-05	SYK	1.1063E-05	DKG	2.49471E-06
IGD	5.76785E-05	TAW	1.1063E-05	DKN	2.49471E-06
IWG	5.76785E-05	TDC	1.1063E-05	DMK	2.49471E-06
MAH	5.76785E-05	TEQ	1.1063E-05	DPF	2.49471E-06
MAW	5.76785E-05	TNE	1.1063E-05	DQW	2.49471E-06
MHG	5.76785E-05	TQE	1.1063E-05	DTA	2.49471E-06
MIA	5.76785E-05	VMA	1.1063E-05	DTG	2.49471E-06
NDH	5.76785E-05	VPC	1.1063E-05	DTK	2.49471E-06
PFY	5.76785E-05	VPG	1.1063E-05	DVY	2.49471E-06
PMY	5.76785E-05	WGI	1.1063E-05	DYN	2.49471E-06
QGW	5.76785E-05	WHG	1.1063E-05	DYV	2.49471E-06
TIE	5.76785E-05	WNG	1.1063E-05	EAA	2.49471E-06
TIY	5.76785E-05	WRN	1.1063E-05	EAT	2.49471E-06
TYN	5.76785E-05	WVM	1.1063E-05	EEF	2.49471E-06
VFE	5.76785E-05	WWF	1.1063E-05	EFH	2.49471E-06
YWG	5.76785E-05	YMG	1.1063E-05	EFR	2.49471E-06

AEC	5.72448E-05	YQT	1.1063E-05	EGD	2.49471E-06
AWH	5.72448E-05	YQW	1.1063E-05	EGK	2.49471E-06
AYE	5.72448E-05	AKK	1.0706E-05	EHY	2.49471E-06
CEV	5.72448E-05	AVY	1.0706E-05	EIY	2.49471E-06
CWA	5.72448E-05	CAA	1.0706E-05	EKI	2.49471E-06
EEG	5.72448E-05	CCF	1.0706E-05	EKV	2.49471E-06
EPD	5.72448E-05	CCS	1.0706E-05	EMQ	2.49471E-06
HIC	5.72448E-05	CEL	1.0706E-05	ENN	2.49471E-06
IWV	5.72448E-05	CKQ	1.0706E-05	EPE	2.49471E-06
MAQ	5.72448E-05	CQP	1.0706E-05	EPV	2.49471E-06
MCA	5.72448E-05	CYL	1.0706E-05	ETW	2.49471E-06
MDA	5.72448E-05	DPV	1.0706E-05	EWV	2.49471E-06
MFV	5.72448E-05	DVM	1.0706E-05	EYE	2.49471E-06
MGM	5.72448E-05	EIK	1.0706E-05	EYN	2.49471E-06
MTD	5.72448E-05	EKF	1.0706E-05	EYQ	2.49471E-06
PNN	5.72448E-05	EVC	1.0706E-05	FFY	2.49471E-06
QAK	5.72448E-05	EYV	1.0706E-05	FHD	2.49471E-06
TKY	5.72448E-05	FFC	1.0706E-05	FIM	2.49471E-06
VEF	5.72448E-05	FMI	1.0706E-05	FIQ	2.49471E-06
VEI	5.72448E-05	FTE	1.0706E-05	FMN	2.49471E-06
VQF	5.72448E-05	HMG	1.0706E-05	FND	2.49471E-06
AFD	5.68112E-05	IAV	1.0706E-05	FPN	2.49471E-06
AYW	5.68112E-05	IAW	1.0706E-05	FVE	2.49471E-06
CGN	5.68112E-05	IDS	1.0706E-05	GFH	2.49471E-06
CVY	5.68112E-05	IIY	1.0706E-05	GHK	2.49471E-06
EGF	5.68112E-05	ILI	1.0706E-05	GHW	2.49471E-06
EGI	5.68112E-05	KAK	1.0706E-05	GKE	2.49471E-06
ENC	5.68112E-05	KIH	1.0706E-05	GMK	2.49471E-06
FGY	5.68112E-05	KWP	1.0706E-05	GNK	2.49471E-06
GCI	5.68112E-05	LIE	1.0706E-05	GPE	2.49471E-06
GII	5.68112E-05	LQV	1.0706E-05	GTE	2.49471E-06
IMV	5.68112E-05	MFH	1.0706E-05	HAW	2.49471E-06
IPE	5.68112E-05	MFV	1.0706E-05	HAY	2.49471E-06
KKH	5.68112E-05	MGW	1.0706E-05	HDK	2.49471E-06
MPD	5.68112E-05	MKF	1.0706E-05	HEW	2.49471E-06
NWG	5.68112E-05	MWQ	1.0706E-05	HFW	2.49471E-06
PCH	5.68112E-05	NCG	1.0706E-05	HGA	2.49471E-06
PCN	5.68112E-05	NGY	1.0706E-05	HGG	2.49471E-06
PIC	5.68112E-05	NIL	1.0706E-05	HLC	2.49471E-06
QQV	5.68112E-05	NVP	1.0706E-05	HLW	2.49471E-06
SWF	5.68112E-05	NWG	1.0706E-05	HNN	2.49471E-06
TDK	5.68112E-05	PWY	1.0706E-05	HPV	2.49471E-06
TEN	5.68112E-05	QGQ	1.0706E-05	HQD	2.49471E-06
TFF	5.68112E-05	QLE	1.0706E-05	HVQ	2.49471E-06

TMI	5.68112E-05	QNK	1.0706E-05	IAF	2.49471E-06
TQF	5.68112E-05	QPK	1.0706E-05	IDN	2.49471E-06
VII	5.68112E-05	QTI	1.0706E-05	IDR	2.49471E-06
VYC	5.68112E-05	QVT	1.0706E-05	IDV	2.49471E-06
WPY	5.68112E-05	QWC	1.0706E-05	IFP	2.49471E-06
AKI	5.63775E-05	QYF	1.0706E-05	IKF	2.49471E-06
AMY	5.63775E-05	REK	1.0706E-05	IPD	2.49471E-06
DCM	5.63775E-05	RFE	1.0706E-05	IQA	2.49471E-06
DNC	5.63775E-05	SCI	1.0706E-05	IQG	2.49471E-06
ECV	5.63775E-05	SCK	1.0706E-05	IQQ	2.49471E-06
FWW	5.63775E-05	SDM	1.0706E-05	IRD	2.49471E-06
GEE	5.63775E-05	SIN	1.0706E-05	IYW	2.49471E-06
GEY	5.63775E-05	SKV	1.0706E-05	KCP	2.49471E-06
IEV	5.63775E-05	SMG	1.0706E-05	KDF	2.49471E-06
IFG	5.63775E-05	TMF	1.0706E-05	KDN	2.49471E-06
IKV	5.63775E-05	TQG	1.0706E-05	KEF	2.49471E-06
MCV	5.63775E-05	TQH	1.0706E-05	KFI	2.49471E-06
MNG	5.63775E-05	VAK	1.0706E-05	KFN	2.49471E-06
NGI	5.63775E-05	VCW	1.0706E-05	KHC	2.49471E-06
PHN	5.63775E-05	VEI	1.0706E-05	KIN	2.49471E-06
PIE	5.63775E-05	VHD	1.0706E-05	KIY	2.49471E-06
QFG	5.63775E-05	VKE	1.0706E-05	KLY	2.49471E-06
QGI	5.63775E-05	VMG	1.0706E-05	KNN	2.49471E-06
QGN	5.63775E-05	VSK	1.0706E-05	KPE	2.49471E-06
QKV	5.63775E-05	VSY	1.0706E-05	KVE	2.49471E-06
QWV	5.63775E-05	VVT	1.0706E-05	KVP	2.49471E-06
TNY	5.63775E-05	VWC	1.0706E-05	LAI	2.49471E-06
TQY	5.63775E-05	WCV	1.0706E-05	LDN	2.49471E-06
TWN	5.63775E-05	WDG	1.0706E-05	LHI	2.49471E-06
VHF	5.63775E-05	WEK	1.0706E-05	MAT	2.49471E-06
VQH	5.63775E-05	WHP	1.0706E-05	MLN	2.49471E-06
VYE	5.63775E-05	YAQ	1.0706E-05	MMH	2.49471E-06
WAF	5.63775E-05	YQC	1.0706E-05	MSW	2.49471E-06
AEI	5.59438E-05	AHV	1.0349E-05	MSY	2.49471E-06
AHY	5.59438E-05	AQQ	1.0349E-05	MWF	2.49471E-06
AID	5.59438E-05	AYY	1.0349E-05	NAF	2.49471E-06
CAY	5.59438E-05	CEN	1.0349E-05	NEI	2.49471E-06
CTY	5.59438E-05	CGG	1.0349E-05	NHQ	2.49471E-06
DMW	5.59438E-05	CIN	1.0349E-05	NKF	2.49471E-06
EYG	5.59438E-05	CSM	1.0349E-05	NMC	2.49471E-06
GEW	5.59438E-05	CTV	1.0349E-05	NNV	2.49471E-06
GWH	5.59438E-05	DIF	1.0349E-05	NPM	2.49471E-06
HFH	5.59438E-05	DSK	1.0349E-05	NPW	2.49471E-06
HNK	5.59438E-05	DSN	1.0349E-05	NTQ	2.49471E-06

IPC	5.59438E-05	DWR	1.0349E-05	PAI	2.49471E-06
KKM	5.59438E-05	EAI	1.0349E-05	PCK	2.49471E-06
MPE	5.59438E-05	EFN	1.0349E-05	PCV	2.49471E-06
NNC	5.59438E-05	EKA	1.0349E-05	PDQ	2.49471E-06
PDD	5.59438E-05	ERE	1.0349E-05	PGV	2.49471E-06
PEC	5.59438E-05	ETQ	1.0349E-05	PNM	2.49471E-06
QAY	5.59438E-05	EVH	1.0349E-05	PQK	2.49471E-06
TCN	5.59438E-05	EWA	1.0349E-05	PQY	2.49471E-06
THF	5.59438E-05	EYR	1.0349E-05	PVA	2.49471E-06
TYK	5.59438E-05	FCN	1.0349E-05	PVP	2.49471E-06
VCY	5.59438E-05	FIK	1.0349E-05	PWE	2.49471E-06
WPN	5.59438E-05	FVC	1.0349E-05	PYQ	2.49471E-06
ACN	5.55101E-05	FVM	1.0349E-05	QAG	2.49471E-06
AEY	5.55101E-05	GCM	1.0349E-05	QCQ	2.49471E-06
AFW	5.55101E-05	GFM	1.0349E-05	QGD	2.49471E-06
EDW	5.55101E-05	GFQ	1.0349E-05	QGP	2.49471E-06
GFK	5.55101E-05	GVF	1.0349E-05	QKM	2.49471E-06
GID	5.55101E-05	HAN	1.0349E-05	QLQ	2.49471E-06
GIY	5.55101E-05	HDT	1.0349E-05	QMC	2.49471E-06
GND	5.55101E-05	HGE	1.0349E-05	QMH	2.49471E-06
GWM	5.55101E-05	HND	1.0349E-05	QPK	2.49471E-06
GWW	5.55101E-05	HYR	1.0349E-05	QQC	2.49471E-06
GYK	5.55101E-05	IAG	1.0349E-05	QRK	2.49471E-06
HNW	5.55101E-05	IKD	1.0349E-05	QRY	2.49471E-06
IEA	5.55101E-05	IMW	1.0349E-05	QWQ	2.49471E-06
IGN	5.55101E-05	IVG	1.0349E-05	QWS	2.49471E-06
MAM	5.55101E-05	KIM	1.0349E-05	RFM	2.49471E-06
MAN	5.55101E-05	KIY	1.0349E-05	RHW	2.49471E-06
MMV	5.55101E-05	KKD	1.0349E-05	RQM	2.49471E-06
MTE	5.55101E-05	KKQ	1.0349E-05	RRM	2.49471E-06
MTH	5.55101E-05	KNI	1.0349E-05	RVF	2.49471E-06
NCW	5.55101E-05	LVK	1.0349E-05	RVY	2.49471E-06
PCY	5.55101E-05	MAF	1.0349E-05	SDE	2.49471E-06
PMF	5.55101E-05	MAV	1.0349E-05	SDF	2.49471E-06
TWE	5.55101E-05	MAY	1.0349E-05	SHN	2.49471E-06
VQY	5.55101E-05	MDS	1.0349E-05	SIK	2.49471E-06
VWE	5.55101E-05	MKK	1.0349E-05	SMD	2.49471E-06
WVE	5.55101E-05	MLE	1.0349E-05	SWY	2.49471E-06
AWQ	5.50765E-05	MQA	1.0349E-05	TDA	2.49471E-06
CEP	5.50765E-05	NCM	1.0349E-05	TDW	2.49471E-06
CFG	5.50765E-05	NQN	1.0349E-05	TFA	2.49471E-06
DDM	5.50765E-05	PEE	1.0349E-05	TFW	2.49471E-06
FNQ	5.50765E-05	PFE	1.0349E-05	TNY	2.49471E-06
GDY	5.50765E-05	PWH	1.0349E-05	TQI	2.49471E-06

GHI	5.50765E-05	PWM	1.0349E-05	TQL	2.49471E-06
GIE	5.50765E-05	QCP	1.0349E-05	TQM	2.49471E-06
HIQ	5.50765E-05	QHC	1.0349E-05	TVG	2.49471E-06
HNC	5.50765E-05	QHD	1.0349E-05	TVI	2.49471E-06
IPY	5.50765E-05	QWD	1.0349E-05	VAM	2.49471E-06
KNM	5.50765E-05	QWM	1.0349E-05	VDN	2.49471E-06
KQG	5.50765E-05	QYD	1.0349E-05	VED	2.49471E-06
MVH	5.50765E-05	QYW	1.0349E-05	VFH	2.49471E-06
QGK	5.50765E-05	SEM	1.0349E-05	VGD	2.49471E-06
TCI	5.50765E-05	SHY	1.0349E-05	VLE	2.49471E-06
AED	5.46428E-05	TDM	1.0349E-05	VME	2.49471E-06
CCG	5.46428E-05	TDN	1.0349E-05	VNG	2.49471E-06
GDK	5.46428E-05	TEY	1.0349E-05	VNW	2.49471E-06
GMY	5.46428E-05	TKE	1.0349E-05	VQE	2.49471E-06
GQN	5.46428E-05	TNC	1.0349E-05	VYD	2.49471E-06
GQQ	5.46428E-05	TWY	1.0349E-05	VYV	2.49471E-06
IAI	5.46428E-05	TYF	1.0349E-05	VYY	2.49471E-06
KND	5.46428E-05	TYY	1.0349E-05	WDV	2.49471E-06
MAD	5.46428E-05	VAQ	1.0349E-05	WWY	2.49471E-06
MWA	5.46428E-05	VGI	1.0349E-05	YAT	2.49471E-06
PYD	5.46428E-05	VKV	1.0349E-05	YEA	2.49471E-06
TMD	5.46428E-05	VLE	1.0349E-05	YGD	2.49471E-06
WAE	5.46428E-05	VMF	1.0349E-05	YGE	2.49471E-06
WGN	5.46428E-05	VMV	1.0349E-05	YHN	2.49471E-06
WPF	5.46428E-05	WCH	1.0349E-05	YIP	2.49471E-06
AIY	5.42091E-05	WFF	1.0349E-05	YMV	2.49471E-06
AQY	5.42091E-05	WGC	1.0349E-05	YNM	2.49471E-06
AWD	5.42091E-05	WKK	1.0349E-05	YQK	2.49471E-06
CFV	5.42091E-05	WPV	1.0349E-05	YVM	2.49471E-06
GCE	5.42091E-05	WTE	1.0349E-05	YWI	2.49471E-06
GKF	5.42091E-05	YEF	1.0349E-05	AAK	2.24524E-06
GQK	5.42091E-05	YNH	1.0349E-05	AAW	2.24524E-06
HHQ	5.42091E-05	ACG	9.9921E-06	ADM	2.24524E-06
MEV	5.42091E-05	AKG	9.9921E-06	AEY	2.24524E-06
MGQ	5.42091E-05	ATW	9.9921E-06	AFC	2.24524E-06
MMA	5.42091E-05	CAF	9.9921E-06	AGF	2.24524E-06
MWV	5.42091E-05	CGE	9.9921E-06	AHE	2.24524E-06
NNK	5.42091E-05	CGF	9.9921E-06	AIC	2.24524E-06
PKI	5.42091E-05	CNF	9.9921E-06	AVC	2.24524E-06
PQK	5.42091E-05	CPT	9.9921E-06	AWG	2.24524E-06
PQY	5.42091E-05	CSD	9.9921E-06	AYD	2.24524E-06
QEG	5.42091E-05	CYR	9.9921E-06	AYN	2.24524E-06
VIY	5.42091E-05	DET	9.9921E-06	AYQ	2.24524E-06
YDW	5.42091E-05	DHG	9.9921E-06	CCM	2.24524E-06

YHM	5.42091E-05	DWI	9.9921E-06	CCT	2.24524E-06
AWF	5.37754E-05	ECE	9.9921E-06	CDA	2.24524E-06
DNN	5.37754E-05	EEV	9.9921E-06	CEN	2.24524E-06
FWQ	5.37754E-05	EHP	9.9921E-06	CHW	2.24524E-06
GCN	5.37754E-05	EKN	9.9921E-06	CHY	2.24524E-06
GCQ	5.37754E-05	EVF	9.9921E-06	CMG	2.24524E-06
HMH	5.37754E-05	FDC	9.9921E-06	CQQ	2.24524E-06
IYG	5.37754E-05	FMW	9.9921E-06	CQS	2.24524E-06
MCG	5.37754E-05	FQV	9.9921E-06	CQV	2.24524E-06
MIV	5.37754E-05	FVY	9.9921E-06	CTG	2.24524E-06
MVQ	5.37754E-05	FWY	9.9921E-06	CTN	2.24524E-06
NNM	5.37754E-05	GFE	9.9921E-06	CWE	2.24524E-06
NNN	5.37754E-05	GWM	9.9921E-06	CYD	2.24524E-06
PHI	5.37754E-05	HDS	9.9921E-06	CYE	2.24524E-06
PHY	5.37754E-05	HHV	9.9921E-06	DEI	2.24524E-06
PYI	5.37754E-05	HKM	9.9921E-06	DFD	2.24524E-06
PYK	5.37754E-05	HPI	9.9921E-06	DFH	2.24524E-06
PYM	5.37754E-05	HRE	9.9921E-06	DGN	2.24524E-06
TQK	5.37754E-05	HVT	9.9921E-06	DGW	2.24524E-06
TWH	5.37754E-05	HYH	9.9921E-06	DHD	2.24524E-06
TWI	5.37754E-05	IFQ	9.9921E-06	DIR	2.24524E-06
WAD	5.37754E-05	IGF	9.9921E-06	DKE	2.24524E-06
WNC	5.37754E-05	IKI	9.9921E-06	DKW	2.24524E-06
WQV	5.37754E-05	IVC	9.9921E-06	DMH	2.24524E-06
YNN	5.37754E-05	KEK	9.9921E-06	DMI	2.24524E-06
AFF	5.33418E-05	KFK	9.9921E-06	DMP	2.24524E-06
CGH	5.33418E-05	KHC	9.9921E-06	DNW	2.24524E-06
CGK	5.33418E-05	KMT	9.9921E-06	DPN	2.24524E-06
CGY	5.33418E-05	KQV	9.9921E-06	DRF	2.24524E-06
DCW	5.33418E-05	KWC	9.9921E-06	DRI	2.24524E-06
EGH	5.33418E-05	MCS	9.9921E-06	DWV	2.24524E-06
IFV	5.33418E-05	MDA	9.9921E-06	DWY	2.24524E-06
KCM	5.33418E-05	MHR	9.9921E-06	EAK	2.24524E-06
KWM	5.33418E-05	MTC	9.9921E-06	ECH	2.24524E-06
MEP	5.33418E-05	NEV	9.9921E-06	ECN	2.24524E-06
MGN	5.33418E-05	NMY	9.9921E-06	ECT	2.24524E-06
NDK	5.33418E-05	NPI	9.9921E-06	EFC	2.24524E-06
NFW	5.33418E-05	NVC	9.9921E-06	EFL	2.24524E-06
NKQ	5.33418E-05	PYK	9.9921E-06	EMD	2.24524E-06
NMM	5.33418E-05	QAC	9.9921E-06	EPK	2.24524E-06
PEE	5.33418E-05	QFA	9.9921E-06	ERG	2.24524E-06
PYF	5.33418E-05	QIH	9.9921E-06	EVK	2.24524E-06
QVF	5.33418E-05	QYM	9.9921E-06	EVT	2.24524E-06
TDY	5.33418E-05	RCY	9.9921E-06	EWR	2.24524E-06

TEY	5.33418E-05	REF	9.9921E-06	FIH	2.24524E-06
TQI	5.33418E-05	RVM	9.9921E-06	FVF	2.24524E-06
TYY	5.33418E-05	SVK	9.9921E-06	FVK	2.24524E-06
VEY	5.33418E-05	THW	9.9921E-06	GHE	2.24524E-06
VFF	5.33418E-05	TYW	9.9921E-06	GIN	2.24524E-06
WCG	5.33418E-05	VIY	9.9921E-06	GQK	2.24524E-06
DNM	5.29081E-05	VWE	9.9921E-06	HCQ	2.24524E-06
EHC	5.29081E-05	WEN	9.9921E-06	HDC	2.24524E-06
GYE	5.29081E-05	WHR	9.9921E-06	HES	2.24524E-06
IAD	5.29081E-05	WID	9.9921E-06	HGC	2.24524E-06
MGE	5.29081E-05	WPE	9.9921E-06	HGS	2.24524E-06
MVD	5.29081E-05	WWG	9.9921E-06	HKA	2.24524E-06
NNQ	5.29081E-05	WWP	9.9921E-06	HNF	2.24524E-06
PFN	5.29081E-05	YGC	9.9921E-06	HNY	2.24524E-06
WCV	5.29081E-05	YHQ	9.9921E-06	HQE	2.24524E-06
YNM	5.29081E-05	YMA	9.9921E-06	HQM	2.24524E-06
AWW	5.24744E-05	YPK	9.9921E-06	HQP	2.24524E-06
CHG	5.24744E-05	YRE	9.9921E-06	HRC	2.24524E-06
DIM	5.24744E-05	YYN	9.9921E-06	HRV	2.24524E-06
ENH	5.24744E-05	ANW	9.6353E-06	HVP	2.24524E-06
GCK	5.24744E-05	AQG	9.6353E-06	HWA	2.24524E-06
GEF	5.24744E-05	AYQ	9.6353E-06	IAA	2.24524E-06
GFF	5.24744E-05	CAG	9.6353E-06	IAK	2.24524E-06
GHF	5.24744E-05	CKT	9.6353E-06	IAW	2.24524E-06
GWK	5.24744E-05	CLE	9.6353E-06	IAY	2.24524E-06
HHH	5.24744E-05	CMT	9.6353E-06	ICF	2.24524E-06
HHN	5.24744E-05	CWA	9.6353E-06	ICI	2.24524E-06
IGH	5.24744E-05	DAC	9.6353E-06	ICK	2.24524E-06
MGK	5.24744E-05	DAK	9.6353E-06	IFN	2.24524E-06
NDQ	5.24744E-05	DPC	9.6353E-06	IKY	2.24524E-06
PEH	5.24744E-05	DTW	9.6353E-06	IQM	2.24524E-06
PWC	5.24744E-05	DVP	9.6353E-06	IRE	2.24524E-06
YCM	5.24744E-05	EGW	9.6353E-06	IRY	2.24524E-06
YDQ	5.24744E-05	EHD	9.6353E-06	IVN	2.24524E-06
AQF	5.20408E-05	EHG	9.6353E-06	IYC	2.24524E-06
AWK	5.20408E-05	EHI	9.6353E-06	KAF	2.24524E-06
AYF	5.20408E-05	EQH	9.6353E-06	KCG	2.24524E-06
GHY	5.20408E-05	EWS	9.6353E-06	KDE	2.24524E-06
GWY	5.20408E-05	FDF	9.6353E-06	KFM	2.24524E-06
HMC	5.20408E-05	FFW	9.6353E-06	KKN	2.24524E-06
IQV	5.20408E-05	FKW	9.6353E-06	KLF	2.24524E-06
IYV	5.20408E-05	FQE	9.6353E-06	KNE	2.24524E-06
KKK	5.20408E-05	HCD	9.6353E-06	KPM	2.24524E-06
NFK	5.20408E-05	HGC	9.6353E-06	KPQ	2.24524E-06

NMC	5.20408E-05	HKW	9.6353E-06	KQV	2.24524E-06
PWI	5.20408E-05	HMP	9.6353E-06	KVA	2.24524E-06
VFI	5.20408E-05	HWA	9.6353E-06	KVY	2.24524E-06
YGY	5.20408E-05	IDR	9.6353E-06	KWF	2.24524E-06
ACI	5.16071E-05	IGK	9.6353E-06	KWG	2.24524E-06
ADY	5.16071E-05	IIM	9.6353E-06	KWS	2.24524E-06
AFI	5.16071E-05	ILE	9.6353E-06	LDE	2.24524E-06
AHF	5.16071E-05	ILK	9.6353E-06	LDI	2.24524E-06
CEG	5.16071E-05	ILY	9.6353E-06	LDK	2.24524E-06
CQG	5.16071E-05	IPW	9.6353E-06	LEE	2.24524E-06
DYM	5.16071E-05	IYD	9.6353E-06	LID	2.24524E-06
GFY	5.16071E-05	KFD	9.6353E-06	LII	2.24524E-06
HKM	5.16071E-05	KTW	9.6353E-06	LVK	2.24524E-06
IEG	5.16071E-05	KVW	9.6353E-06	MCG	2.24524E-06
KCC	5.16071E-05	KWK	9.6353E-06	MDH	2.24524E-06
MFG	5.16071E-05	MCD	9.6353E-06	MDR	2.24524E-06
NIW	5.16071E-05	MGY	9.6353E-06	MER	2.24524E-06
NMQ	5.16071E-05	MHP	9.6353E-06	MHL	2.24524E-06
NNH	5.16071E-05	MIL	9.6353E-06	MKP	2.24524E-06
PDY	5.16071E-05	NEW	9.6353E-06	MMI	2.24524E-06
QVY	5.16071E-05	NFY	9.6353E-06	MNH	2.24524E-06
QYV	5.16071E-05	NHI	9.6353E-06	MNY	2.24524E-06
TEI	5.16071E-05	NIM	9.6353E-06	MPD	2.24524E-06
VEW	5.16071E-05	NYM	9.6353E-06	MPM	2.24524E-06
WTE	5.16071E-05	PHE	9.6353E-06	MPN	2.24524E-06
YGI	5.16071E-05	PHK	9.6353E-06	MQT	2.24524E-06
YHK	5.16071E-05	PKE	9.6353E-06	MSK	2.24524E-06
YHW	5.16071E-05	QAY	9.6353E-06	MTD	2.24524E-06
AYD	5.11734E-05	QDK	9.6353E-06	MWC	2.24524E-06
AYY	5.11734E-05	QKC	9.6353E-06	MYL	2.24524E-06
CGQ	5.11734E-05	QKN	9.6353E-06	MYW	2.24524E-06
CKG	5.11734E-05	QMR	9.6353E-06	NAA	2.24524E-06
CPF	5.11734E-05	QQC	9.6353E-06	NCA	2.24524E-06
DDC	5.11734E-05	SKK	9.6353E-06	NCP	2.24524E-06
FMC	5.11734E-05	SME	9.6353E-06	NEW	2.24524E-06
GCY	5.11734E-05	TAM	9.6353E-06	NFN	2.24524E-06
GME	5.11734E-05	TMK	9.6353E-06	NGM	2.24524E-06
GQY	5.11734E-05	TTF	9.6353E-06	NHF	2.24524E-06
GWD	5.11734E-05	TVQ	9.6353E-06	NIL	2.24524E-06
HEQ	5.11734E-05	VFN	9.6353E-06	NLF	2.24524E-06
HHM	5.11734E-05	VWM	9.6353E-06	NME	2.24524E-06
HIN	5.11734E-05	WET	9.6353E-06	NMY	2.24524E-06
HKC	5.11734E-05	WIQ	9.6353E-06	NND	2.24524E-06
HMM	5.11734E-05	WNN	9.6353E-06	NNK	2.24524E-06

HNQ	5.11734E-05	WPI	9.6353E-06	NNW	2.24524E-06
KDH	5.11734E-05	WRY	9.6353E-06	NNY	2.24524E-06
KEH	5.11734E-05	WTD	9.6353E-06	NPV	2.24524E-06
MQV	5.11734E-05	WVI	9.6353E-06	NRM	2.24524E-06
NHK	5.11734E-05	WYP	9.6353E-06	NRW	2.24524E-06
NYW	5.11734E-05	YEQ	9.6353E-06	NVI	2.24524E-06
PNF	5.11734E-05	YFK	9.6353E-06	NWC	2.24524E-06
QKW	5.11734E-05	YQI	9.6353E-06	NWY	2.24524E-06
WGF	5.11734E-05	YQV	9.6353E-06	NYW	2.24524E-06
WGI	5.11734E-05	YSQ	9.6353E-06	NYY	2.24524E-06
WPI	5.11734E-05	YVM	9.6353E-06	PDI	2.24524E-06
WYG	5.11734E-05	YWV	9.6353E-06	PDT	2.24524E-06
YNQ	5.11734E-05	YYM	9.6353E-06	PKV	2.24524E-06
ADK	5.07397E-05	ACH	9.2784E-06	PVE	2.24524E-06
AFN	5.07397E-05	AFD	9.2784E-06	QAA	2.24524E-06
DMM	5.07397E-05	AFI	9.2784E-06	QEC	2.24524E-06
EQG	5.07397E-05	AGW	9.2784E-06	QET	2.24524E-06
FCW	5.07397E-05	AQE	9.2784E-06	QHM	2.24524E-06
GWE	5.07397E-05	AVW	9.2784E-06	QIN	2.24524E-06
HIW	5.07397E-05	AYI	9.2784E-06	QLE	2.24524E-06
IQG	5.07397E-05	CCR	9.2784E-06	QLF	2.24524E-06
MIG	5.07397E-05	CEP	9.2784E-06	QLI	2.24524E-06
MPI	5.07397E-05	CRI	9.2784E-06	QQD	2.24524E-06
NDC	5.07397E-05	CTC	9.2784E-06	QQQ	2.24524E-06
NIC	5.07397E-05	DCT	9.2784E-06	QSM	2.24524E-06
PKY	5.07397E-05	DDT	9.2784E-06	QTE	2.24524E-06
TFI	5.07397E-05	DHR	9.2784E-06	QVC	2.24524E-06
THI	5.07397E-05	DMT	9.2784E-06	QVL	2.24524E-06
TWQ	5.07397E-05	DTQ	9.2784E-06	QVT	2.24524E-06
AFE	5.03061E-05	DVQ	9.2784E-06	QWG	2.24524E-06
CGE	5.03061E-05	DVV	9.2784E-06	QWI	2.24524E-06
CWG	5.03061E-05	EAC	9.2784E-06	QYF	2.24524E-06
DKK	5.03061E-05	ECH	9.2784E-06	RAM	2.24524E-06
DKQ	5.03061E-05	ECN	9.2784E-06	RIE	2.24524E-06
DQE	5.03061E-05	EMK	9.2784E-06	RII	2.24524E-06
DWW	5.03061E-05	EVI	9.2784E-06	SAE	2.24524E-06
EIC	5.03061E-05	EVP	9.2784E-06	SAF	2.24524E-06
FFC	5.03061E-05	FEF	9.2784E-06	SFE	2.24524E-06
GFE	5.03061E-05	FMC	9.2784E-06	SME	2.24524E-06
HDM	5.03061E-05	FPN	9.2784E-06	SNI	2.24524E-06
HFQ	5.03061E-05	GCW	9.2784E-06	SNK	2.24524E-06
HNE	5.03061E-05	GIC	9.2784E-06	SQK	2.24524E-06
MEA	5.03061E-05	GWY	9.2784E-06	SQY	2.24524E-06
MQG	5.03061E-05	HDM	9.2784E-06	SVE	2.24524E-06

NDE	5.03061E-05	HFY	9.2784E-06	SVF	2.24524E-06
NYM	5.03061E-05	HGW	9.2784E-06	SYN	2.24524E-06
QAF	5.03061E-05	HKH	9.2784E-06	SYY	2.24524E-06
QKC	5.03061E-05	HML	9.2784E-06	TAC	2.24524E-06
QKM	5.03061E-05	HNC	9.2784E-06	TAF	2.24524E-06
WPD	5.03061E-05	HWP	9.2784E-06	TAY	2.24524E-06
YHC	5.03061E-05	ICT	9.2784E-06	TCS	2.24524E-06
YKM	5.03061E-05	IHM	9.2784E-06	TCV	2.24524E-06
AIF	4.98724E-05	IHQ	9.2784E-06	THP	2.24524E-06
AWI	4.98724E-05	IIH	9.2784E-06	THV	2.24524E-06
CGD	4.98724E-05	IYV	9.2784E-06	TIV	2.24524E-06
DFC	4.98724E-05	KHR	9.2784E-06	TKV	2.24524E-06
EKN	4.98724E-05	KKI	9.2784E-06	TKW	2.24524E-06
FQH	4.98724E-05	KNK	9.2784E-06	TMA	2.24524E-06
FYQ	4.98724E-05	KQG	9.2784E-06	TMD	2.24524E-06
GEI	4.98724E-05	KQK	9.2784E-06	TMQ	2.24524E-06
GHD	4.98724E-05	KYE	9.2784E-06	TPW	2.24524E-06
GWN	4.98724E-05	LQD	9.2784E-06	TQK	2.24524E-06
HHW	4.98724E-05	MAD	9.2784E-06	TQV	2.24524E-06
HKW	4.98724E-05	MKC	9.2784E-06	TRE	2.24524E-06
HNH	4.98724E-05	MTF	9.2784E-06	TRK	2.24524E-06
ICA	4.98724E-05	MYD	9.2784E-06	TSI	2.24524E-06
IPI	4.98724E-05	NFN	9.2784E-06	TTM	2.24524E-06
KHW	4.98724E-05	NYR	9.2784E-06	TVD	2.24524E-06
MGI	4.98724E-05	PCF	9.2784E-06	TVQ	2.24524E-06
NDW	4.98724E-05	PII	9.2784E-06	TWK	2.24524E-06
PQF	4.98724E-05	PKF	9.2784E-06	TYV	2.24524E-06
QNC	4.98724E-05	PQN	9.2784E-06	VDC	2.24524E-06
VQI	4.98724E-05	QEW	9.2784E-06	VDG	2.24524E-06
YIC	4.98724E-05	QFC	9.2784E-06	VDH	2.24524E-06
AEF	4.94387E-05	QFW	9.2784E-06	VDV	2.24524E-06
AFY	4.94387E-05	QMT	9.2784E-06	VEC	2.24524E-06
CGI	4.94387E-05	QTE	9.2784E-06	VFC	2.24524E-06
DEM	4.94387E-05	RHV	9.2784E-06	VFV	2.24524E-06
DMC	4.94387E-05	RMI	9.2784E-06	VHD	2.24524E-06
GHE	4.94387E-05	RQW	9.2784E-06	VID	2.24524E-06
GYI	4.94387E-05	SEN	9.2784E-06	VIF	2.24524E-06
HCW	4.94387E-05	SQV	9.2784E-06	VIP	2.24524E-06
HNM	4.94387E-05	SWY	9.2784E-06	VNK	2.24524E-06
HYC	4.94387E-05	SYY	9.2784E-06	WEF	2.24524E-06
IAF	4.94387E-05	TDK	9.2784E-06	WMK	2.24524E-06
PEN	4.94387E-05	VCC	9.2784E-06	WNE	2.24524E-06
PWH	4.94387E-05	VEN	9.2784E-06	YAK	2.24524E-06
QHH	4.94387E-05	VHN	9.2784E-06	YCI	2.24524E-06

QVI	4.94387E-05	VNW	9.2784E-06	YCN	2.24524E-06
VYI	4.94387E-05	VYV	9.2784E-06	YEV	2.24524E-06
WWG	4.94387E-05	WKH	9.2784E-06	YIT	2.24524E-06
CQV	4.9005E-05	YDF	9.2784E-06	YIV	2.24524E-06
DNK	4.9005E-05	YHH	9.2784E-06	YNN	2.24524E-06
DNW	4.9005E-05	YIG	9.2784E-06	YRK	2.24524E-06
EEH	4.9005E-05	YIY	9.2784E-06	ACG	1.99577E-06
GDI	4.9005E-05	YVG	9.2784E-06	AEW	1.99577E-06
GYF	4.9005E-05	AAK	8.9215E-06	AFV	1.99577E-06
HCK	4.9005E-05	ADY	8.9215E-06	AGE	1.99577E-06
HYW	4.9005E-05	AFW	8.9215E-06	AGK	1.99577E-06
KFC	4.9005E-05	AHI	8.9215E-06	AGW	1.99577E-06
KKC	4.9005E-05	AWD	8.9215E-06	AHK	1.99577E-06
KNC	4.9005E-05	CAP	8.9215E-06	AIA	1.99577E-06
NCM	4.9005E-05	CEA	8.9215E-06	AIQ	1.99577E-06
NIQ	4.9005E-05	CET	8.9215E-06	ANK	1.99577E-06
NKD	4.9005E-05	CFP	8.9215E-06	ASQ	1.99577E-06
NYD	4.9005E-05	CGN	8.9215E-06	AVG	1.99577E-06
PDF	4.9005E-05	CHR	8.9215E-06	AVP	1.99577E-06
TEF	4.9005E-05	CTQ	8.9215E-06	AVY	1.99577E-06
TWD	4.9005E-05	DFV	8.9215E-06	CCH	1.99577E-06
YDE	4.9005E-05	DID	8.9215E-06	CDS	1.99577E-06
AWY	4.85714E-05	DIR	8.9215E-06	CDV	1.99577E-06
DNH	4.85714E-05	DIW	8.9215E-06	CEG	1.99577E-06
FKQ	4.85714E-05	DMH	8.9215E-06	CEK	1.99577E-06
HDK	4.85714E-05	DYT	8.9215E-06	CFF	1.99577E-06
HFM	4.85714E-05	EAE	8.9215E-06	CGA	1.99577E-06
KKD	4.85714E-05	ECG	8.9215E-06	CGN	1.99577E-06
KNE	4.85714E-05	EFW	8.9215E-06	CKA	1.99577E-06
KNN	4.85714E-05	EGC	8.9215E-06	CNQ	1.99577E-06
MVF	4.85714E-05	EQF	8.9215E-06	CNY	1.99577E-06
NDN	4.85714E-05	EQQ	8.9215E-06	CRD	1.99577E-06
NHH	4.85714E-05	EVE	8.9215E-06	CTK	1.99577E-06
NHM	4.85714E-05	EWV	8.9215E-06	CVH	1.99577E-06
PWN	4.85714E-05	EYD	8.9215E-06	CVK	1.99577E-06
WCC	4.85714E-05	FCD	8.9215E-06	CVS	1.99577E-06
DCN	4.81377E-05	FDG	8.9215E-06	CYM	1.99577E-06
DDK	4.81377E-05	FKC	8.9215E-06	DAN	1.99577E-06
DIW	4.81377E-05	FVQ	8.9215E-06	DAT	1.99577E-06
DMH	4.81377E-05	FYI	8.9215E-06	DAV	1.99577E-06
DYC	4.81377E-05	GQD	8.9215E-06	DDD	1.99577E-06
ECC	4.81377E-05	GWW	8.9215E-06	DFA	1.99577E-06
EKM	4.81377E-05	HFD	8.9215E-06	DHI	1.99577E-06
EYM	4.81377E-05	HFG	8.9215E-06	DIP	1.99577E-06

HMK	4.81377E-05	HKD	8.9215E-06	DKD	1.99577E-06
KHM	4.81377E-05	HRI	8.9215E-06	DMG	1.99577E-06
KHN	4.81377E-05	HVV	8.9215E-06	DNY	1.99577E-06
KWG	4.81377E-05	ICC	8.9215E-06	DRY	1.99577E-06
KYM	4.81377E-05	ICP	8.9215E-06	DTW	1.99577E-06
MVE	4.81377E-05	ICW	8.9215E-06	DVA	1.99577E-06
NYE	4.81377E-05	IEH	8.9215E-06	DVF	1.99577E-06
PWD	4.81377E-05	ILN	8.9215E-06	DVW	1.99577E-06
QHM	4.81377E-05	IWN	8.9215E-06	DWK	1.99577E-06
TIF	4.81377E-05	IYR	8.9215E-06	DYY	1.99577E-06
VHI	4.81377E-05	KAF	8.9215E-06	EAF	1.99577E-06
YCC	4.81377E-05	KIE	8.9215E-06	EAG	1.99577E-06
YMH	4.81377E-05	KMI	8.9215E-06	ECS	1.99577E-06
ACF	4.7704E-05	KYD	8.9215E-06	EDE	1.99577E-06
AII	4.7704E-05	LFE	8.9215E-06	EFA	1.99577E-06
DIQ	4.7704E-05	LQM	8.9215E-06	EFG	1.99577E-06
EQQ	4.7704E-05	MDH	8.9215E-06	EGE	1.99577E-06
FCM	4.7704E-05	MGI	8.9215E-06	EGG	1.99577E-06
FEQ	4.7704E-05	MID	8.9215E-06	EID	1.99577E-06
FKC	4.7704E-05	MQS	8.9215E-06	EIP	1.99577E-06
FMM	4.7704E-05	MRM	8.9215E-06	ESM	1.99577E-06
FNK	4.7704E-05	MSE	8.9215E-06	ETG	1.99577E-06
GEK	4.7704E-05	MWT	8.9215E-06	ETK	1.99577E-06
HCM	4.7704E-05	NDP	8.9215E-06	EWH	1.99577E-06
HYM	4.7704E-05	NEF	8.9215E-06	EWQ	1.99577E-06
IMN	4.7704E-05	NFF	8.9215E-06	FFK	1.99577E-06
KDW	4.7704E-05	NIK	8.9215E-06	FHK	1.99577E-06
KKQ	4.7704E-05	NIQ	8.9215E-06	FII	1.99577E-06
KKW	4.7704E-05	NNI	8.9215E-06	FKD	1.99577E-06
KNH	4.7704E-05	NWC	8.9215E-06	FVD	1.99577E-06
KYW	4.7704E-05	PQF	8.9215E-06	GCE	1.99577E-06
MAF	4.7704E-05	PYF	8.9215E-06	GFQ	1.99577E-06
NCC	4.7704E-05	QGH	8.9215E-06	GGI	1.99577E-06
NEQ	4.7704E-05	QIG	8.9215E-06	GNE	1.99577E-06
NFM	4.7704E-05	QPM	8.9215E-06	GYK	1.99577E-06
NHD	4.7704E-05	QYC	8.9215E-06	HCV	1.99577E-06
NKN	4.7704E-05	QYY	8.9215E-06	HDS	1.99577E-06
NYC	4.7704E-05	TNK	8.9215E-06	HFQ	1.99577E-06
PEF	4.7704E-05	TWC	8.9215E-06	HGP	1.99577E-06
PKF	4.7704E-05	VEE	8.9215E-06	HHC	1.99577E-06
QNW	4.7704E-05	VID	8.9215E-06	HHF	1.99577E-06
WGY	4.7704E-05	VIE	8.9215E-06	HHK	1.99577E-06
WHC	4.7704E-05	VIH	8.9215E-06	HKK	1.99577E-06
YNC	4.7704E-05	VVD	8.9215E-06	HLD	1.99577E-06

CMM	4.72704E-05	VVQ	8.9215E-06	HMY	1.99577E-06
DEN	4.72704E-05	VYN	8.9215E-06	HRD	1.99577E-06
DHH	4.72704E-05	WAW	8.9215E-06	HRY	1.99577E-06
DKW	4.72704E-05	WFN	8.9215E-06	HSI	1.99577E-06
EIW	4.72704E-05	WQH	8.9215E-06	HTE	1.99577E-06
ENW	4.72704E-05	WYM	8.9215E-06	HTM	1.99577E-06
FDM	4.72704E-05	WYN	8.9215E-06	HTV	1.99577E-06
FKK	4.72704E-05	YLE	8.9215E-06	HVT	1.99577E-06
FWK	4.72704E-05	YPN	8.9215E-06	HWM	1.99577E-06
HHC	4.72704E-05	YQP	8.9215E-06	HWV	1.99577E-06
KMH	4.72704E-05	YRK	8.9215E-06	HYN	1.99577E-06
NIN	4.72704E-05	YYY	8.9215E-06	HYY	1.99577E-06
NYH	4.72704E-05	AWG	8.5647E-06	IDA	1.99577E-06
NYQ	4.72704E-05	AWV	8.5647E-06	IDP	1.99577E-06
PDI	4.72704E-05	CFA	8.5647E-06	IEM	1.99577E-06
PII	4.72704E-05	CIE	8.5647E-06	IFA	1.99577E-06
QDC	4.72704E-05	CRM	8.5647E-06	IFK	1.99577E-06
QHD	4.72704E-05	CVI	8.5647E-06	IGI	1.99577E-06
TYF	4.72704E-05	DAE	8.5647E-06	IHQ	1.99577E-06
YDI	4.72704E-05	DFA	8.5647E-06	IIP	1.99577E-06
YKQ	4.72704E-05	DFF	8.5647E-06	IPH	1.99577E-06
YNK	4.72704E-05	DGW	8.5647E-06	IQH	1.99577E-06
YWM	4.72704E-05	DHD	8.5647E-06	IQV	1.99577E-06
CGF	4.68367E-05	DKG	8.5647E-06	IVH	1.99577E-06
CVI	4.68367E-05	DMN	8.5647E-06	IYM	1.99577E-06
DHM	4.68367E-05	DQG	8.5647E-06	IYV	1.99577E-06
DKE	4.68367E-05	DTE	8.5647E-06	KCS	1.99577E-06
DMY	4.68367E-05	DVN	8.5647E-06	KHF	1.99577E-06
DNF	4.68367E-05	DWP	8.5647E-06	KHQ	1.99577E-06
EMM	4.68367E-05	DYI	8.5647E-06	KHV	1.99577E-06
ENE	4.68367E-05	ECD	8.5647E-06	KIA	1.99577E-06
FIQ	4.68367E-05	EKC	8.5647E-06	KIH	1.99577E-06
FKM	4.68367E-05	EKD	8.5647E-06	KMF	1.99577E-06
FMH	4.68367E-05	EKV	8.5647E-06	KNQ	1.99577E-06
FNW	4.68367E-05	EQI	8.5647E-06	KQN	1.99577E-06
HCN	4.68367E-05	EYM	8.5647E-06	KRW	1.99577E-06
HDN	4.68367E-05	FAK	8.5647E-06	KTI	1.99577E-06
HKQ	4.68367E-05	FMH	8.5647E-06	KTQ	1.99577E-06
HYQ	4.68367E-05	FVW	8.5647E-06	KVM	1.99577E-06
KFQ	4.68367E-05	GWC	8.5647E-06	LDD	1.99577E-06
KHH	4.68367E-05	HDA	8.5647E-06	LEK	1.99577E-06
KQC	4.68367E-05	HDP	8.5647E-06	LFE	1.99577E-06
MYV	4.68367E-05	HHQ	8.5647E-06	LIK	1.99577E-06
NCH	4.68367E-05	HIQ	8.5647E-06	MAQ	1.99577E-06

NEC	4.68367E-05	HKN	8.5647E-06	MCD	1.99577E-06
NWM	4.68367E-05	HMS	8.5647E-06	MCQ	1.99577E-06
TCF	4.68367E-05	HVH	8.5647E-06	MDL	1.99577E-06
WAI	4.68367E-05	IDH	8.5647E-06	MES	1.99577E-06
WEC	4.68367E-05	ITC	8.5647E-06	MGM	1.99577E-06
YHN	4.68367E-05	IWG	8.5647E-06	MGW	1.99577E-06
DDQ	4.6403E-05	IYH	8.5647E-06	MHC	1.99577E-06
DNQ	4.6403E-05	KEE	8.5647E-06	MHG	1.99577E-06
FFM	4.6403E-05	KEM	8.5647E-06	MKK	1.99577E-06
FMQ	4.6403E-05	KMH	8.5647E-06	MKN	1.99577E-06
GQF	4.6403E-05	KWN	8.5647E-06	MLD	1.99577E-06
HFC	4.6403E-05	LQC	8.5647E-06	MMV	1.99577E-06
NDM	4.6403E-05	LQW	8.5647E-06	MNW	1.99577E-06
NWW	4.6403E-05	MAN	8.5647E-06	MPF	1.99577E-06
PWF	4.6403E-05	MHN	8.5647E-06	MQR	1.99577E-06
QHC	4.6403E-05	MHV	8.5647E-06	MSQ	1.99577E-06
QWG	4.6403E-05	MLF	8.5647E-06	MTM	1.99577E-06
TWF	4.6403E-05	MVP	8.5647E-06	MVS	1.99577E-06
YDC	4.6403E-05	MWP	8.5647E-06	MYI	1.99577E-06
YFM	4.6403E-05	NFQ	8.5647E-06	MYP	1.99577E-06
DHC	4.59693E-05	NMF	8.5647E-06	NCY	1.99577E-06
DHN	4.59693E-05	NNC	8.5647E-06	NHC	1.99577E-06
DKC	4.59693E-05	NQD	8.5647E-06	NIH	1.99577E-06
EDH	4.59693E-05	NQH	8.5647E-06	NIQ	1.99577E-06
ENQ	4.59693E-05	NYC	8.5647E-06	NKM	1.99577E-06
FDK	4.59693E-05	PQI	8.5647E-06	NNE	1.99577E-06
HKI	4.59693E-05	QAE	8.5647E-06	NPI	1.99577E-06
HKK	4.59693E-05	QHY	8.5647E-06	NPK	1.99577E-06
HKN	4.59693E-05	RKI	8.5647E-06	NQV	1.99577E-06
HQH	4.59693E-05	SCY	8.5647E-06	NQY	1.99577E-06
HYD	4.59693E-05	TDE	8.5647E-06	NRY	1.99577E-06
HYE	4.59693E-05	TEM	8.5647E-06	NWI	1.99577E-06
KMW	4.59693E-05	TIM	8.5647E-06	NYM	1.99577E-06
KQK	4.59693E-05	TIQ	8.5647E-06	PAM	1.99577E-06
MAY	4.59693E-05	TQI	8.5647E-06	PDE	1.99577E-06
MFM	4.59693E-05	WAM	8.5647E-06	PDV	1.99577E-06
MVY	4.59693E-05	WFC	8.5647E-06	PEI	1.99577E-06
NED	4.59693E-05	WYK	8.5647E-06	PEK	1.99577E-06
NEW	4.59693E-05	YCG	8.5647E-06	PGF	1.99577E-06
NHN	4.59693E-05	YCH	8.5647E-06	PHI	1.99577E-06
NKK	4.59693E-05	YKW	8.5647E-06	PME	1.99577E-06
NMI	4.59693E-05	YVF	8.5647E-06	PVK	1.99577E-06
NMN	4.59693E-05	YWW	8.5647E-06	PVV	1.99577E-06
PCI	4.59693E-05	AAI	8.2078E-06	PYF	1.99577E-06

TCH	4.59693E-05	ACW	8.2078E-06	QDF	1.99577E-06
TCY	4.59693E-05	AFV	8.2078E-06	QDY	1.99577E-06
YMQ	4.59693E-05	CCV	8.2078E-06	QEG	1.99577E-06
ACY	4.55357E-05	CDF	8.2078E-06	QEM	1.99577E-06
DKH	4.55357E-05	CDQ	8.2078E-06	QGA	1.99577E-06
DKN	4.55357E-05	CER	8.2078E-06	QGT	1.99577E-06
EMK	4.55357E-05	CFH	8.2078E-06	QHF	1.99577E-06
EMW	4.55357E-05	CGW	8.2078E-06	QHK	1.99577E-06
FDW	4.55357E-05	CHS	8.2078E-06	QNI	1.99577E-06
FFE	4.55357E-05	DDF	8.2078E-06	QNK	1.99577E-06
HND	4.55357E-05	DEN	8.2078E-06	QNM	1.99577E-06
ICM	4.55357E-05	DFP	8.2078E-06	QPI	1.99577E-06
IIG	4.55357E-05	DNY	8.2078E-06	QPQ	1.99577E-06
KIC	4.55357E-05	EAM	8.2078E-06	QQH	1.99577E-06
KKN	4.55357E-05	ECR	8.2078E-06	QQW	1.99577E-06
KME	4.55357E-05	EFH	8.2078E-06	QVW	1.99577E-06
KMM	4.55357E-05	EHC	8.2078E-06	QWW	1.99577E-06
KQM	4.55357E-05	ENE	8.2078E-06	QYQ	1.99577E-06
KWW	4.55357E-05	FCC	8.2078E-06	RDM	1.99577E-06
MGY	4.55357E-05	FFH	8.2078E-06	REI	1.99577E-06
NCK	4.55357E-05	FMN	8.2078E-06	RHK	1.99577E-06
NHW	4.55357E-05	FQH	8.2078E-06	RQF	1.99577E-06
NIM	4.55357E-05	GDE	8.2078E-06	RVW	1.99577E-06
PFF	4.55357E-05	GWI	8.2078E-06	RYM	1.99577E-06
TYI	4.55357E-05	GYC	8.2078E-06	SCD	1.99577E-06
CDH	4.5102E-05	GYE	8.2078E-06	SCF	1.99577E-06
DCC	4.5102E-05	GYW	8.2078E-06	SCY	1.99577E-06
DHW	4.5102E-05	HGK	8.2078E-06	SDK	1.99577E-06
DKM	4.5102E-05	HIH	8.2078E-06	SFK	1.99577E-06
FCQ	4.5102E-05	HKY	8.2078E-06	SFQ	1.99577E-06
FHQ	4.5102E-05	IAN	8.2078E-06	SHI	1.99577E-06
FIW	4.5102E-05	IHN	8.2078E-06	SIM	1.99577E-06
FNH	4.5102E-05	IKF	8.2078E-06	SQI	1.99577E-06
GQI	4.5102E-05	IMH	8.2078E-06	SYF	1.99577E-06
HDW	4.5102E-05	INW	8.2078E-06	TCG	1.99577E-06
HMI	4.5102E-05	IQH	8.2078E-06	TEC	1.99577E-06
HYK	4.5102E-05	IVT	8.2078E-06	TES	1.99577E-06
IGI	4.5102E-05	KAM	8.2078E-06	TFF	1.99577E-06
KEQ	4.5102E-05	KCW	8.2078E-06	TFM	1.99577E-06
KFW	4.5102E-05	KKM	8.2078E-06	TGW	1.99577E-06
KHC	4.5102E-05	KVI	8.2078E-06	TIF	1.99577E-06
KMN	4.5102E-05	KVN	8.2078E-06	TMP	1.99577E-06
KQH	4.5102E-05	KWE	8.2078E-06	TNV	1.99577E-06
NKM	4.5102E-05	LIK	8.2078E-06	TPI	1.99577E-06

QNN	4.5102E-05	MFQ	8.2078E-06	TQE	1.99577E-06
YCW	4.5102E-05	MHF	8.2078E-06	TTE	1.99577E-06
YMM	4.5102E-05	MNG	8.2078E-06	TTI	1.99577E-06
AWN	4.46683E-05	MTM	8.2078E-06	TVE	1.99577E-06
DDN	4.46683E-05	MTN	8.2078E-06	TYC	1.99577E-06
DKD	4.46683E-05	MWH	8.2078E-06	TYD	1.99577E-06
EKY	4.46683E-05	NEG	8.2078E-06	TYG	1.99577E-06
FCC	4.46683E-05	NHM	8.2078E-06	VAI	1.99577E-06
FWH	4.46683E-05	NIF	8.2078E-06	VEG	1.99577E-06
FYC	4.46683E-05	NMN	8.2078E-06	VGI	1.99577E-06
HKD	4.46683E-05	NNW	8.2078E-06	VII	1.99577E-06
HWK	4.46683E-05	NYD	8.2078E-06	VIK	1.99577E-06
KEC	4.46683E-05	NYG	8.2078E-06	VQC	1.99577E-06
KHQ	4.46683E-05	PCI	8.2078E-06	VQD	1.99577E-06
KMK	4.46683E-05	PCK	8.2078E-06	VVA	1.99577E-06
KNK	4.46683E-05	PQV	8.2078E-06	VYK	1.99577E-06
KNQ	4.46683E-05	QAQ	8.2078E-06	VYN	1.99577E-06
NHY	4.46683E-05	QCK	8.2078E-06	WCY	1.99577E-06
NIE	4.46683E-05	QDD	8.2078E-06	WFK	1.99577E-06
NIH	4.46683E-05	QET	8.2078E-06	WIN	1.99577E-06
NKH	4.46683E-05	QIW	8.2078E-06	WKE	1.99577E-06
NKW	4.46683E-05	QLW	8.2078E-06	WNM	1.99577E-06
NQK	4.46683E-05	QQP	8.2078E-06	WPI	1.99577E-06
VYF	4.46683E-05	RQD	8.2078E-06	YAA	1.99577E-06
WDW	4.46683E-05	SKE	8.2078E-06	YFN	1.99577E-06
WNM	4.46683E-05	THI	8.2078E-06	YFQ	1.99577E-06
YFC	4.46683E-05	TKM	8.2078E-06	YHI	1.99577E-06
YFW	4.46683E-05	TWI	8.2078E-06	YIA	1.99577E-06
YQQ	4.46683E-05	TWW	8.2078E-06	YMF	1.99577E-06
YYW	4.46683E-05	VPK	8.2078E-06	YYM	1.99577E-06
DDF	4.42346E-05	WFV	8.2078E-06	AAE	1.7463E-06
DQN	4.42346E-05	WIM	8.2078E-06	AAG	1.7463E-06
DYW	4.42346E-05	WIW	8.2078E-06	AEG	1.7463E-06
EDC	4.42346E-05	WYC	8.2078E-06	AFH	1.7463E-06
EHI	4.42346E-05	YEH	8.2078E-06	AFN	1.7463E-06
FHM	4.42346E-05	YHC	8.2078E-06	AYE	1.7463E-06
HDQ	4.42346E-05	YKM	8.2078E-06	CAF	1.7463E-06
HWM	4.42346E-05	YND	8.2078E-06	CDC	1.7463E-06
KFE	4.42346E-05	YQF	8.2078E-06	CDE	1.7463E-06
KIM	4.42346E-05	YTE	8.2078E-06	CET	1.7463E-06
KMC	4.42346E-05	ACV	7.8509E-06	CFH	1.7463E-06
KQE	4.42346E-05	ADC	7.8509E-06	CGY	1.7463E-06
MWG	4.42346E-05	AGC	7.8509E-06	CHT	1.7463E-06
NNW	4.42346E-05	AHY	7.8509E-06	CIC	1.7463E-06

NWH	4.42346E-05	AII	7.8509E-06	CIE	1.7463E-06
QDM	4.42346E-05	AME	7.8509E-06	CIQ	1.7463E-06
YCQ	4.42346E-05	CID	7.8509E-06	CNV	1.7463E-06
YDD	4.42346E-05	CIV	7.8509E-06	CPQ	1.7463E-06
YFQ	4.42346E-05	CKH	7.8509E-06	CQE	1.7463E-06
YNI	4.42346E-05	CMA	7.8509E-06	CRN	1.7463E-06
CDW	4.3801E-05	CNK	7.8509E-06	CSE	1.7463E-06
CMC	4.3801E-05	CQT	7.8509E-06	CVA	1.7463E-06
DHQ	4.3801E-05	DCH	7.8509E-06	CYY	1.7463E-06
DMK	4.3801E-05	DHQ	7.8509E-06	DCN	1.7463E-06
EDQ	4.3801E-05	DMD	7.8509E-06	DDC	1.7463E-06
EIM	4.3801E-05	DVG	7.8509E-06	DFI	1.7463E-06
FMD	4.3801E-05	EHN	7.8509E-06	DFN	1.7463E-06
FNM	4.3801E-05	EWP	7.8509E-06	DGD	1.7463E-06
GWF	4.3801E-05	EYG	7.8509E-06	DRE	1.7463E-06
HDE	4.3801E-05	FCI	7.8509E-06	DTE	1.7463E-06
HHK	4.3801E-05	FEI	7.8509E-06	DWC	1.7463E-06
HMW	4.3801E-05	FEV	7.8509E-06	DWH	1.7463E-06
HNF	4.3801E-05	FFN	7.8509E-06	ECV	1.7463E-06
HQK	4.3801E-05	FHK	7.8509E-06	ECW	1.7463E-06
MKM	4.3801E-05	HGY	7.8509E-06	EFD	1.7463E-06
MYG	4.3801E-05	HNM	7.8509E-06	EFF	1.7463E-06
NEN	4.3801E-05	HPE	7.8509E-06	EIC	1.7463E-06
NIF	4.3801E-05	HPW	7.8509E-06	EIM	1.7463E-06
QCW	4.3801E-05	IDY	7.8509E-06	ELE	1.7463E-06
QHQ	4.3801E-05	IGM	7.8509E-06	EPM	1.7463E-06
WNQ	4.3801E-05	IHF	7.8509E-06	EQM	1.7463E-06
YDH	4.3801E-05	IIK	7.8509E-06	EWD	1.7463E-06
YKC	4.3801E-05	IKV	7.8509E-06	FED	1.7463E-06
YYM	4.3801E-05	IMQ	7.8509E-06	FFE	1.7463E-06
AWE	4.33673E-05	IYG	7.8509E-06	FNE	1.7463E-06
DCK	4.33673E-05	KQY	7.8509E-06	HAD	1.7463E-06
DDW	4.33673E-05	KVQ	7.8509E-06	HAE	1.7463E-06
DEY	4.33673E-05	KYY	7.8509E-06	HAG	1.7463E-06
DFE	4.33673E-05	LME	7.8509E-06	HAL	1.7463E-06
DIC	4.33673E-05	MDQ	7.8509E-06	HAP	1.7463E-06
DME	4.33673E-05	MDV	7.8509E-06	HEG	1.7463E-06
EIN	4.33673E-05	NHP	7.8509E-06	HEK	1.7463E-06
EKQ	4.33673E-05	NHR	7.8509E-06	HGD	1.7463E-06
EYD	4.33673E-05	NMH	7.8509E-06	HGV	1.7463E-06
FFN	4.33673E-05	PWC	7.8509E-06	HHQ	1.7463E-06
FNE	4.33673E-05	QAI	7.8509E-06	HKD	1.7463E-06
FWE	4.33673E-05	QHH	7.8509E-06	HKY	1.7463E-06
GFI	4.33673E-05	QMQ	7.8509E-06	HLE	1.7463E-06

HCE	4.33673E-05	QVC	7.8509E-06	HMG	1.7463E-06
HEH	4.33673E-05	QWG	7.8509E-06	HMM	1.7463E-06
INE	4.33673E-05	RCI	7.8509E-06	HQV	1.7463E-06
KFM	4.33673E-05	RIC	7.8509E-06	HQW	1.7463E-06
KHD	4.33673E-05	RMM	7.8509E-06	HRK	1.7463E-06
KMQ	4.33673E-05	RQC	7.8509E-06	HVV	1.7463E-06
MPF	4.33673E-05	RQF	7.8509E-06	HWD	1.7463E-06
NFD	4.33673E-05	SMN	7.8509E-06	HWK	1.7463E-06
NHI	4.33673E-05	SQY	7.8509E-06	IAM	1.7463E-06
NKI	4.33673E-05	TEK	7.8509E-06	IAV	1.7463E-06
NMY	4.33673E-05	TMM	7.8509E-06	IED	1.7463E-06
WFW	4.33673E-05	VAW	7.8509E-06	IEV	1.7463E-06
WNH	4.33673E-05	VDF	7.8509E-06	IEW	1.7463E-06
YKD	4.33673E-05	VEC	7.8509E-06	IFV	1.7463E-06
YMN	4.33673E-05	VFH	7.8509E-06	IHD	1.7463E-06
DCQ	4.29336E-05	VFV	7.8509E-06	IHF	1.7463E-06
DKI	4.29336E-05	VHG	7.8509E-06	IIE	1.7463E-06
DWM	4.29336E-05	VWD	7.8509E-06	IKE	1.7463E-06
DYH	4.29336E-05	VYE	7.8509E-06	IMF	1.7463E-06
EHQ	4.29336E-05	WCS	7.8509E-06	INW	1.7463E-06
END	4.29336E-05	WEE	7.8509E-06	IPA	1.7463E-06
FHC	4.29336E-05	WEG	7.8509E-06	IQD	1.7463E-06
FHH	4.29336E-05	WEV	7.8509E-06	ISE	1.7463E-06
FKN	4.29336E-05	WEW	7.8509E-06	ITE	1.7463E-06
FMW	4.29336E-05	WFQ	7.8509E-06	IVA	1.7463E-06
HDF	4.29336E-05	WMK	7.8509E-06	IVI	1.7463E-06
HEN	4.29336E-05	WNK	7.8509E-06	IWY	1.7463E-06
HMD	4.29336E-05	WQC	7.8509E-06	IYQ	1.7463E-06
HMQ	4.29336E-05	WQQ	7.8509E-06	KCY	1.7463E-06
HNN	4.29336E-05	WWA	7.8509E-06	KFK	1.7463E-06
IHW	4.29336E-05	WYV	7.8509E-06	KKI	1.7463E-06
IMM	4.29336E-05	YAV	7.8509E-06	KKK	1.7463E-06
KDQ	4.29336E-05	YCA	7.8509E-06	KMN	1.7463E-06
NDD	4.29336E-05	YCT	7.8509E-06	KPF	1.7463E-06
NHC	4.29336E-05	YKN	7.8509E-06	KPK	1.7463E-06
NNE	4.29336E-05	YNI	7.8509E-06	KRE	1.7463E-06
NYK	4.29336E-05	YYV	7.8509E-06	KTM	1.7463E-06
PWY	4.29336E-05	ADI	7.4941E-06	KWK	1.7463E-06
QCC	4.29336E-05	AEW	7.4941E-06	KWY	1.7463E-06
QHK	4.29336E-05	AFF	7.4941E-06	LEN	1.7463E-06
QNQ	4.29336E-05	AVD	7.4941E-06	LQI	1.7463E-06
QYC	4.29336E-05	CDV	7.4941E-06	LVE	1.7463E-06
YCE	4.29336E-05	CDW	7.4941E-06	LVN	1.7463E-06
YCH	4.29336E-05	CFC	7.4941E-06	MAG	1.7463E-06

YDY	4.29336E-05	CMV	7.4941E-06	MAN	1.7463E-06
YHE	4.29336E-05	CNQ	7.4941E-06	MAP	1.7463E-06
YHY	4.29336E-05	CRG	7.4941E-06	MAW	1.7463E-06
CNM	4.25E-05	CRN	7.4941E-06	MCV	1.7463E-06
DCH	4.25E-05	DNE	7.4941E-06	MEP	1.7463E-06
DEC	4.25E-05	DNI	7.4941E-06	MEQ	1.7463E-06
DEW	4.25E-05	DQP	7.4941E-06	MFC	1.7463E-06
DFQ	4.25E-05	DWY	7.4941E-06	MFW	1.7463E-06
DFW	4.25E-05	DYC	7.4941E-06	MGF	1.7463E-06
DNE	4.25E-05	DYR	7.4941E-06	MGV	1.7463E-06
DQC	4.25E-05	EEI	7.4941E-06	MHQ	1.7463E-06
DWK	4.25E-05	EQY	7.4941E-06	MHW	1.7463E-06
EMI	4.25E-05	FDH	7.4941E-06	MIL	1.7463E-06
FHN	4.25E-05	FEC	7.4941E-06	MLC	1.7463E-06
FMN	4.25E-05	FWD	7.4941E-06	MLH	1.7463E-06
FYW	4.25E-05	GFI	7.4941E-06	MLK	1.7463E-06
GWI	4.25E-05	HAK	7.4941E-06	MNG	1.7463E-06
GYY	4.25E-05	HEY	7.4941E-06	MNQ	1.7463E-06
HEM	4.25E-05	HQV	7.4941E-06	MPC	1.7463E-06
HFK	4.25E-05	HVI	7.4941E-06	MPH	1.7463E-06
HMN	4.25E-05	HVM	7.4941E-06	MQL	1.7463E-06
KCF	4.25E-05	HWG	7.4941E-06	MQQ	1.7463E-06
KDF	4.25E-05	IDC	7.4941E-06	NFD	1.7463E-06
KKI	4.25E-05	IEW	7.4941E-06	NIY	1.7463E-06
KMD	4.25E-05	IGI	7.4941E-06	NKE	1.7463E-06
KYK	4.25E-05	ILC	7.4941E-06	NKI	1.7463E-06
NFE	4.25E-05	IQW	7.4941E-06	NMD	1.7463E-06
NHE	4.25E-05	IWM	7.4941E-06	NPE	1.7463E-06
NII	4.25E-05	KCK	7.4941E-06	NPQ	1.7463E-06
NKE	4.25E-05	KED	7.4941E-06	NRE	1.7463E-06
NKF	4.25E-05	KFY	7.4941E-06	NSE	1.7463E-06
NME	4.25E-05	KGI	7.4941E-06	NTF	1.7463E-06
NND	4.25E-05	KII	7.4941E-06	NTV	1.7463E-06
QKK	4.25E-05	KKE	7.4941E-06	NYN	1.7463E-06
QKQ	4.25E-05	LEN	7.4941E-06	PEE	1.7463E-06
QNH	4.25E-05	LQK	7.4941E-06	PEY	1.7463E-06
VFY	4.25E-05	MCC	7.4941E-06	PFI	1.7463E-06
WMD	4.25E-05	MDM	7.4941E-06	PGI	1.7463E-06
YFN	4.25E-05	MKI	7.4941E-06	PMV	1.7463E-06
YMW	4.25E-05	MKQ	7.4941E-06	PNK	1.7463E-06
YNW	4.25E-05	MLY	7.4941E-06	PYI	1.7463E-06
YWW	4.25E-05	MNQ	7.4941E-06	PYK	1.7463E-06
AYI	4.20663E-05	MQI	7.4941E-06	QAF	1.7463E-06
DDD	4.20663E-05	MSK	7.4941E-06	QAT	1.7463E-06

DQW	4.20663E-05	MWD	7.4941E-06	QEY	1.7463E-06
ECM	4.20663E-05	NCI	7.4941E-06	QGK	1.7463E-06
EHD	4.20663E-05	NDY	7.4941E-06	QID	1.7463E-06
EKE	4.20663E-05	NFE	7.4941E-06	QIF	1.7463E-06
FCE	4.20663E-05	NFK	7.4941E-06	QIW	1.7463E-06
FDN	4.20663E-05	PCM	7.4941E-06	QMA	1.7463E-06
FEM	4.20663E-05	PEM	7.4941E-06	QRF	1.7463E-06
FHD	4.20663E-05	PKM	7.4941E-06	RGM	1.7463E-06
FHI	4.20663E-05	PYI	7.4941E-06	RHM	1.7463E-06
FMI	4.20663E-05	QCY	7.4941E-06	RIK	1.7463E-06
FNN	4.20663E-05	QEY	7.4941E-06	RME	1.7463E-06
FWF	4.20663E-05	QFF	7.4941E-06	SHK	1.7463E-06
FWI	4.20663E-05	QFG	7.4941E-06	SIW	1.7463E-06
HED	4.20663E-05	QGP	7.4941E-06	SKE	1.7463E-06
HHY	4.20663E-05	QIN	7.4941E-06	SQF	1.7463E-06
HII	4.20663E-05	RQI	7.4941E-06	SQN	1.7463E-06
HME	4.20663E-05	RYK	7.4941E-06	SVA	1.7463E-06
HQM	4.20663E-05	SQH	7.4941E-06	SVN	1.7463E-06
INM	4.20663E-05	TDI	7.4941E-06	SYK	1.7463E-06
KCK	4.20663E-05	VDI	7.4941E-06	TAA	1.7463E-06
KEW	4.20663E-05	VDV	7.4941E-06	TAV	1.7463E-06
KNI	4.20663E-05	VKG	7.4941E-06	TAW	1.7463E-06
KWH	4.20663E-05	VMH	7.4941E-06	TCE	1.7463E-06
KWQ	4.20663E-05	VQD	7.4941E-06	TDC	1.7463E-06
KYD	4.20663E-05	WHY	7.4941E-06	TEA	1.7463E-06
NCE	4.20663E-05	WKD	7.4941E-06	TEE	1.7463E-06
NEF	4.20663E-05	WMG	7.4941E-06	TFP	1.7463E-06
NNI	4.20663E-05	WNY	7.4941E-06	TGD	1.7463E-06
NQH	4.20663E-05	YCI	7.4941E-06	TGF	1.7463E-06
NQW	4.20663E-05	YNN	7.4941E-06	TGI	1.7463E-06
QIC	4.20663E-05	ACF	7.1372E-06	TGV	1.7463E-06
QMQ	4.20663E-05	AHE	7.1372E-06	THY	1.7463E-06
WKQ	4.20663E-05	AHK	7.1372E-06	TIA	1.7463E-06
WMM	4.20663E-05	AKC	7.1372E-06	TKK	1.7463E-06
WYW	4.20663E-05	AWC	7.1372E-06	TKM	1.7463E-06
YDK	4.20663E-05	CAT	7.1372E-06	TNI	1.7463E-06
YEC	4.20663E-05	CDP	7.1372E-06	TQQ	1.7463E-06
YEK	4.20663E-05	CDR	7.1372E-06	TWE	1.7463E-06
YFI	4.20663E-05	CND	7.1372E-06	VAK	1.7463E-06
YKE	4.20663E-05	CYP	7.1372E-06	VAT	1.7463E-06
YNH	4.20663E-05	DEW	7.1372E-06	VDY	1.7463E-06
YYC	4.20663E-05	DHH	7.1372E-06	VFM	1.7463E-06
CDM	4.16326E-05	ETE	7.1372E-06	VHK	1.7463E-06
CDN	4.16326E-05	EWN	7.1372E-06	WCM	1.7463E-06

CHM	4.16326E-05	FIY	7.1372E-06	WDM	1.7463E-06
CNC	4.16326E-05	GDK	7.1372E-06	WFI	1.7463E-06
DCD	4.16326E-05	GQW	7.1372E-06	WII	1.7463E-06
DMQ	4.16326E-05	HCT	7.1372E-06	WMM	1.7463E-06
ECK	4.16326E-05	HET	7.1372E-06	WNK	1.7463E-06
EDM	4.16326E-05	HKC	7.1372E-06	WQI	1.7463E-06
EDN	4.16326E-05	HSK	7.1372E-06	WTE	1.7463E-06
EFM	4.16326E-05	HVG	7.1372E-06	YAE	1.7463E-06
EKI	4.16326E-05	HYG	7.1372E-06	YDF	1.7463E-06
ENM	4.16326E-05	HYW	7.1372E-06	YEF	1.7463E-06
EWM	4.16326E-05	IAF	7.1372E-06	YEM	1.7463E-06
FFW	4.16326E-05	ICN	7.1372E-06	YFM	1.7463E-06
FQQ	4.16326E-05	IDW	7.1372E-06	YGY	1.7463E-06
FYE	4.16326E-05	IKK	7.1372E-06	YID	1.7463E-06
FYH	4.16326E-05	KDK	7.1372E-06	YKI	1.7463E-06
HCC	4.16326E-05	KIQ	7.1372E-06	YMI	1.7463E-06
HEW	4.16326E-05	LMI	7.1372E-06	YPI	1.7463E-06
HNY	4.16326E-05	LQE	7.1372E-06	YVF	1.7463E-06
HQE	4.16326E-05	MDW	7.1372E-06	YVK	1.7463E-06
HQQ	4.16326E-05	MFI	7.1372E-06	YYN	1.7463E-06
IFW	4.16326E-05	MMW	7.1372E-06	ACH	1.49683E-06
IMC	4.16326E-05	MPM	7.1372E-06	ADD	1.49683E-06
IWM	4.16326E-05	MSQ	7.1372E-06	ADE	1.49683E-06
KDE	4.16326E-05	NHN	7.1372E-06	AGC	1.49683E-06
KEN	4.16326E-05	NHQ	7.1372E-06	AGI	1.49683E-06
KIK	4.16326E-05	NKC	7.1372E-06	AHW	1.49683E-06
KQN	4.16326E-05	NQG	7.1372E-06	AIE	1.49683E-06
KQY	4.16326E-05	NQK	7.1372E-06	AIG	1.49683E-06
NFQ	4.16326E-05	NVN	7.1372E-06	AME	1.49683E-06
NID	4.16326E-05	NVQ	7.1372E-06	AWI	1.49683E-06
NMH	4.16326E-05	NYF	7.1372E-06	CAA	1.49683E-06
PEY	4.16326E-05	NYN	7.1372E-06	CAV	1.49683E-06
QDW	4.16326E-05	NYQ	7.1372E-06	CCF	1.49683E-06
QFW	4.16326E-05	PHI	7.1372E-06	CDD	1.49683E-06
QHE	4.16326E-05	PIM	7.1372E-06	CDT	1.49683E-06
TFY	4.16326E-05	QAW	7.1372E-06	CEA	1.49683E-06
WDD	4.16326E-05	QEQ	7.1372E-06	CEF	1.49683E-06
WDF	4.16326E-05	QIY	7.1372E-06	CEY	1.49683E-06
WFC	4.16326E-05	QKF	7.1372E-06	CFM	1.49683E-06
WKW	4.16326E-05	QKV	7.1372E-06	CFP	1.49683E-06
YEH	4.16326E-05	QMD	7.1372E-06	CFQ	1.49683E-06
YNY	4.16326E-05	QWP	7.1372E-06	CGD	1.49683E-06
CDK	4.11989E-05	QWQ	7.1372E-06	CIA	1.49683E-06
CYC	4.11989E-05	QYE	7.1372E-06	CIV	1.49683E-06

DEH	4.11989E-05	QYI	7.1372E-06	CKM	1.49683E-06
DHE	4.11989E-05	RCK	7.1372E-06	CMM	1.49683E-06
DIK	4.11989E-05	RHI	7.1372E-06	CMY	1.49683E-06
DYQ	4.11989E-05	SAM	7.1372E-06	CPA	1.49683E-06
EEW	4.11989E-05	SIC	7.1372E-06	CPN	1.49683E-06
EMC	4.11989E-05	SMD	7.1372E-06	CQD	1.49683E-06
FCD	4.11989E-05	SMI	7.1372E-06	CTA	1.49683E-06
FHK	4.11989E-05	SQF	7.1372E-06	CVC	1.49683E-06
FKH	4.11989E-05	SQI	7.1372E-06	CVD	1.49683E-06
FYK	4.11989E-05	SWD	7.1372E-06	CWY	1.49683E-06
FYN	4.11989E-05	TCI	7.1372E-06	DCH	1.49683E-06
HFW	4.11989E-05	TMC	7.1372E-06	DCQ	1.49683E-06
HIM	4.11989E-05	TMQ	7.1372E-06	DDN	1.49683E-06
HWH	4.11989E-05	TYI	7.1372E-06	DDV	1.49683E-06
HYH	4.11989E-05	VCF	7.1372E-06	DFF	1.49683E-06
IDM	4.11989E-05	VED	7.1372E-06	DFW	1.49683E-06
KCY	4.11989E-05	VFC	7.1372E-06	DGC	1.49683E-06
KDM	4.11989E-05	VGF	7.1372E-06	DGS	1.49683E-06
KDY	4.11989E-05	VIF	7.1372E-06	DGV	1.49683E-06
KIW	4.11989E-05	VNC	7.1372E-06	DMN	1.49683E-06
KKE	4.11989E-05	VVH	7.1372E-06	DMY	1.49683E-06
KQF	4.11989E-05	VYK	7.1372E-06	DND	1.49683E-06
NCQ	4.11989E-05	WCN	7.1372E-06	DNN	1.49683E-06
NMF	4.11989E-05	WDE	7.1372E-06	DPV	1.49683E-06
NQE	4.11989E-05	WDF	7.1372E-06	DQC	1.49683E-06
NQQ	4.11989E-05	WED	7.1372E-06	DVG	1.49683E-06
PYY	4.11989E-05	WKN	7.1372E-06	DVS	1.49683E-06
QDN	4.11989E-05	WMN	7.1372E-06	DWG	1.49683E-06
QKH	4.11989E-05	WPD	7.1372E-06	ECD	1.49683E-06
QQQ	4.11989E-05	WPK	7.1372E-06	EDK	1.49683E-06
TWY	4.11989E-05	WWT	7.1372E-06	EDM	1.49683E-06
WKH	4.11989E-05	WYG	7.1372E-06	EDV	1.49683E-06
YED	4.11989E-05	YAM	7.1372E-06	EEM	1.49683E-06
YND	4.11989E-05	YHE	7.1372E-06	EFI	1.49683E-06
YWK	4.11989E-05	YID	7.1372E-06	EFN	1.49683E-06
YYF	4.11989E-05	AAW	6.7804E-06	EFV	1.49683E-06
DFK	4.07653E-05	AEC	6.7804E-06	EFW	1.49683E-06
DHD	4.07653E-05	AED	6.7804E-06	EFY	1.49683E-06
DHK	4.07653E-05	AEK	6.7804E-06	EGY	1.49683E-06
DMF	4.07653E-05	AFK	6.7804E-06	EIF	1.49683E-06
DYN	4.07653E-05	AQN	6.7804E-06	EIH	1.49683E-06
EKH	4.07653E-05	AQY	6.7804E-06	ELF	1.49683E-06
ENF	4.07653E-05	CCQ	6.7804E-06	EMA	1.49683E-06
FDD	4.07653E-05	CIA	6.7804E-06	EMP	1.49683E-06

FEK	4.07653E-05	CIP	6.7804E-06	EMV	1.49683E-06
FHW	4.07653E-05	CRK	6.7804E-06	ENE	1.49683E-06
FIK	4.07653E-05	CVF	6.7804E-06	EQN	1.49683E-06
FNC	4.07653E-05	CVK	6.7804E-06	EQW	1.49683E-06
FYD	4.07653E-05	CWM	6.7804E-06	ERW	1.49683E-06
HDD	4.07653E-05	DHY	6.7804E-06	ETI	1.49683E-06
HFD	4.07653E-05	DIC	6.7804E-06	ETM	1.49683E-06
HHE	4.07653E-05	DIG	6.7804E-06	EVD	1.49683E-06
HQW	4.07653E-05	DIH	6.7804E-06	EVY	1.49683E-06
HWW	4.07653E-05	DIY	6.7804E-06	EWY	1.49683E-06
IDC	4.07653E-05	DKD	6.7804E-06	FDF	1.49683E-06
IDW	4.07653E-05	DKE	6.7804E-06	FDY	1.49683E-06
INN	4.07653E-05	DPK	6.7804E-06	FFN	1.49683E-06
KFY	4.07653E-05	DVC	6.7804E-06	FIK	1.49683E-06
KIH	4.07653E-05	EEW	6.7804E-06	FVN	1.49683E-06
NFY	4.07653E-05	EIE	6.7804E-06	GCK	1.49683E-06
NKC	4.07653E-05	FCK	6.7804E-06	GDM	1.49683E-06
QIH	4.07653E-05	FFI	6.7804E-06	GIE	1.49683E-06
QMM	4.07653E-05	FHE	6.7804E-06	GIQ	1.49683E-06
QMW	4.07653E-05	FIQ	6.7804E-06	GIW	1.49683E-06
WDM	4.07653E-05	FQD	6.7804E-06	GME	1.49683E-06
WIW	4.07653E-05	FQM	6.7804E-06	HCD	1.49683E-06
YIM	4.07653E-05	FQN	6.7804E-06	HCE	1.49683E-06
YKW	4.07653E-05	FVD	6.7804E-06	HCY	1.49683E-06
CIK	4.03316E-05	GFK	6.7804E-06	HDT	1.49683E-06
CWM	4.03316E-05	HAC	6.7804E-06	HEC	1.49683E-06
DFM	4.03316E-05	HAM	6.7804E-06	HET	1.49683E-06
DHY	4.03316E-05	HDC	6.7804E-06	HFG	1.49683E-06
DMI	4.03316E-05	HFI	6.7804E-06	HFN	1.49683E-06
DQM	4.03316E-05	HGN	6.7804E-06	HGM	1.49683E-06
DQQ	4.03316E-05	HHT	6.7804E-06	HHM	1.49683E-06
DYI	4.03316E-05	HMK	6.7804E-06	HIT	1.49683E-06
EDK	4.03316E-05	HMN	6.7804E-06	HIY	1.49683E-06
EFC	4.03316E-05	HNY	6.7804E-06	HKW	1.49683E-06
EIH	4.03316E-05	HQG	6.7804E-06	HMK	1.49683E-06
ENK	4.03316E-05	HVN	6.7804E-06	HML	1.49683E-06
EQK	4.03316E-05	HVP	6.7804E-06	HMQ	1.49683E-06
FCN	4.03316E-05	HWE	6.7804E-06	HMW	1.49683E-06
FMK	4.03316E-05	IIN	6.7804E-06	HND	1.49683E-06
FQM	4.03316E-05	IMF	6.7804E-06	HPQ	1.49683E-06
HQC	4.03316E-05	IMY	6.7804E-06	HRM	1.49683E-06
HYN	4.03316E-05	INK	6.7804E-06	HRQ	1.49683E-06
IIC	4.03316E-05	IQN	6.7804E-06	HVC	1.49683E-06
IKM	4.03316E-05	ITW	6.7804E-06	HVY	1.49683E-06

INK	4.03316E-05	IYC	6.7804E-06	HWY	1.49683E-06
IQE	4.03316E-05	KQH	6.7804E-06	HYV	1.49683E-06
KCI	4.03316E-05	KVK	6.7804E-06	IAE	1.49683E-06
KDD	4.03316E-05	MHG	6.7804E-06	IAN	1.49683E-06
KDK	4.03316E-05	MIC	6.7804E-06	IDF	1.49683E-06
KKY	4.03316E-05	MMH	6.7804E-06	IDQ	1.49683E-06
KMI	4.03316E-05	MPI	6.7804E-06	IEA	1.49683E-06
MMM	4.03316E-05	MTD	6.7804E-06	IEK	1.49683E-06
NDI	4.03316E-05	MTE	6.7804E-06	IGD	1.49683E-06
NHF	4.03316E-05	NCY	6.7804E-06	IIQ	1.49683E-06
NWF	4.03316E-05	NDN	6.7804E-06	INQ	1.49683E-06
PCF	4.03316E-05	NHY	6.7804E-06	IPY	1.49683E-06
QHN	4.03316E-05	NYE	6.7804E-06	ISD	1.49683E-06
QHW	4.03316E-05	PED	6.7804E-06	IVD	1.49683E-06
QMC	4.03316E-05	PME	6.7804E-06	IVY	1.49683E-06
QMH	4.03316E-05	PQD	6.7804E-06	IYG	1.49683E-06
WNK	4.03316E-05	PQY	6.7804E-06	IYN	1.49683E-06
YEM	4.03316E-05	QAK	6.7804E-06	KAV	1.49683E-06
YHF	4.03316E-05	QDF	6.7804E-06	KFE	1.49683E-06
YIN	4.03316E-05	QFY	6.7804E-06	KFW	1.49683E-06
YMK	4.03316E-05	QGY	6.7804E-06	KFY	1.49683E-06
CIW	3.98979E-05	QHV	6.7804E-06	KGC	1.49683E-06
CMY	3.98979E-05	QVP	6.7804E-06	KHY	1.49683E-06
DDI	3.98979E-05	QYV	6.7804E-06	KMI	1.49683E-06
DID	3.98979E-05	RCM	6.7804E-06	KMY	1.49683E-06
DIF	3.98979E-05	RQE	6.7804E-06	KSI	1.49683E-06
EIF	3.98979E-05	SEK	6.7804E-06	KTV	1.49683E-06
EYE	3.98979E-05	SMM	6.7804E-06	KYY	1.49683E-06
EYW	3.98979E-05	SQC	6.7804E-06	LEI	1.49683E-06
FEW	3.98979E-05	SQD	6.7804E-06	MCF	1.49683E-06
FFH	3.98979E-05	TDF	6.7804E-06	MCI	1.49683E-06
FIM	3.98979E-05	TFE	6.7804E-06	MCY	1.49683E-06
FKD	3.98979E-05	TKW	6.7804E-06	MDA	1.49683E-06
FME	3.98979E-05	TQM	6.7804E-06	MET	1.49683E-06
FNI	3.98979E-05	VAF	6.7804E-06	MEY	1.49683E-06
FWC	3.98979E-05	VEF	6.7804E-06	MFM	1.49683E-06
HEC	3.98979E-05	VFM	6.7804E-06	MFP	1.49683E-06
HIE	3.98979E-05	VHC	6.7804E-06	MGC	1.49683E-06
IEM	3.98979E-05	VIQ	6.7804E-06	MGG	1.49683E-06
KCE	3.98979E-05	VKW	6.7804E-06	MGK	1.49683E-06
KDN	3.98979E-05	VMD	6.7804E-06	MIA	1.49683E-06
KFH	3.98979E-05	VQF	6.7804E-06	MIH	1.49683E-06
KIE	3.98979E-05	VQM	6.7804E-06	MPE	1.49683E-06
MND	3.98979E-05	VVW	6.7804E-06	MQH	1.49683E-06

NEE	3.98979E-05	VYF	6.7804E-06	MQY	1.49683E-06
NFH	3.98979E-05	VYR	6.7804E-06	MTE	1.49683E-06
QEW	3.98979E-05	WCE	6.7804E-06	MYC	1.49683E-06
QIQ	3.98979E-05	WCF	6.7804E-06	NAM	1.49683E-06
QKD	3.98979E-05	WEY	6.7804E-06	NDF	1.49683E-06
WDC	3.98979E-05	WHF	6.7804E-06	NEM	1.49683E-06
WKF	3.98979E-05	WWE	6.7804E-06	NFQ	1.49683E-06
WWM	3.98979E-05	YFY	6.7804E-06	NHK	1.49683E-06
WWW	3.98979E-05	YMT	6.7804E-06	NID	1.49683E-06
WYH	3.98979E-05	YQD	6.7804E-06	NIK	1.49683E-06
YIW	3.98979E-05	YQY	6.7804E-06	NIP	1.49683E-06
YKY	3.98979E-05	YVN	6.7804E-06	NIW	1.49683E-06
YQM	3.98979E-05	YVQ	6.7804E-06	NMI	1.49683E-06
YWC	3.98979E-05	ACI	6.4235E-06	NNM	1.49683E-06
YYH	3.98979E-05	CCW	6.4235E-06	NPF	1.49683E-06
YYK	3.98979E-05	CDM	6.4235E-06	NQM	1.49683E-06
CDE	3.94642E-05	CEE	6.4235E-06	NVC	1.49683E-06
CNK	3.94642E-05	CPG	6.4235E-06	NVV	1.49683E-06
DDY	3.94642E-05	CPI	6.4235E-06	NYI	1.49683E-06
DIH	3.94642E-05	CQG	6.4235E-06	NYV	1.49683E-06
EHM	3.94642E-05	CTW	6.4235E-06	PCE	1.49683E-06
EMN	3.94642E-05	DDM	6.4235E-06	PDF	1.49683E-06
FDC	3.94642E-05	DEF	6.4235E-06	PGM	1.49683E-06
FDH	3.94642E-05	DHC	6.4235E-06	PKK	1.49683E-06
FHE	3.94642E-05	DHI	6.4235E-06	QAI	1.49683E-06
FIE	3.94642E-05	DKK	6.4235E-06	QAV	1.49683E-06
HCQ	3.94642E-05	DLE	6.4235E-06	QCI	1.49683E-06
HDI	3.94642E-05	DNW	6.4235E-06	QFF	1.49683E-06
HFI	3.94642E-05	DPE	6.4235E-06	QIE	1.49683E-06
IFM	3.94642E-05	DWD	6.4235E-06	QIQ	1.49683E-06
KFN	3.94642E-05	ECP	6.4235E-06	QMN	1.49683E-06
KNY	3.94642E-05	ECQ	6.4235E-06	QPM	1.49683E-06
NCD	3.94642E-05	EFI	6.4235E-06	QQY	1.49683E-06
NEK	3.94642E-05	EFV	6.4235E-06	QRI	1.49683E-06
NMD	3.94642E-05	EPE	6.4235E-06	QVA	1.49683E-06
NNY	3.94642E-05	FEE	6.4235E-06	QVD	1.49683E-06
NWQ	3.94642E-05	FMM	6.4235E-06	QYK	1.49683E-06
QEH	3.94642E-05	FYW	6.4235E-06	RDI	1.49683E-06
QII	3.94642E-05	HCY	6.4235E-06	SDM	1.49683E-06
WEK	3.94642E-05	HHP	6.4235E-06	SGF	1.49683E-06
WIQ	3.94642E-05	HYQ	6.4235E-06	SGI	1.49683E-06
YDN	3.94642E-05	IAC	6.4235E-06	SIP	1.49683E-06
YMI	3.94642E-05	IIF	6.4235E-06	SIQ	1.49683E-06
YNE	3.94642E-05	INC	6.4235E-06	SNM	1.49683E-06

CKM	3.90306E-05	INE	6.4235E-06	SWD	1.49683E-06
CNH	3.90306E-05	IQV	6.4235E-06	SYI	1.49683E-06
CNN	3.90306E-05	IWK	6.4235E-06	TAG	1.49683E-06
DDH	3.90306E-05	IYF	6.4235E-06	TAI	1.49683E-06
DEK	3.90306E-05	IYK	6.4235E-06	TAN	1.49683E-06
DND	3.90306E-05	KCM	6.4235E-06	TCM	1.49683E-06
EEC	3.90306E-05	KKW	6.4235E-06	TCP	1.49683E-06
FCH	3.90306E-05	KQI	6.4235E-06	TCQ	1.49683E-06
FEY	3.90306E-05	KVY	6.4235E-06	TDD	1.49683E-06
FIC	3.90306E-05	LEK	6.4235E-06	TDF	1.49683E-06
FKW	3.90306E-05	LMM	6.4235E-06	TDG	1.49683E-06
FNF	3.90306E-05	MCF	6.4235E-06	TDN	1.49683E-06
FNY	3.90306E-05	MHW	6.4235E-06	TDP	1.49683E-06
HDH	3.90306E-05	MKG	6.4235E-06	TEM	1.49683E-06
HFE	3.90306E-05	MLK	6.4235E-06	TFH	1.49683E-06
HMY	3.90306E-05	MMT	6.4235E-06	TGE	1.49683E-06
IKD	3.90306E-05	MQY	6.4235E-06	TGY	1.49683E-06
IWQ	3.90306E-05	MTK	6.4235E-06	THC	1.49683E-06
KHK	3.90306E-05	MVT	6.4235E-06	THE	1.49683E-06
KQQ	3.90306E-05	NDE	6.4235E-06	THN	1.49683E-06
KWC	3.90306E-05	NHH	6.4235E-06	TIK	1.49683E-06
NIK	3.90306E-05	NIN	6.4235E-06	TIW	1.49683E-06
NQC	3.90306E-05	NKK	6.4235E-06	TKE	1.49683E-06
NQF	3.90306E-05	NQF	6.4235E-06	TKF	1.49683E-06
QDF	3.90306E-05	PDK	6.4235E-06	TMF	1.49683E-06
QMD	3.90306E-05	PQE	6.4235E-06	TNA	1.49683E-06
QNM	3.90306E-05	PQW	6.4235E-06	TNK	1.49683E-06
WDQ	3.90306E-05	QGK	6.4235E-06	TQG	1.49683E-06
WMC	3.90306E-05	QHQ	6.4235E-06	TTV	1.49683E-06
WMH	3.90306E-05	QNE	6.4235E-06	TWC	1.49683E-06
YFK	3.90306E-05	QTM	6.4235E-06	TYE	1.49683E-06
YHH	3.90306E-05	QVV	6.4235E-06	TYH	1.49683E-06
YKK	3.90306E-05	SWC	6.4235E-06	TYW	1.49683E-06
CMQ	3.85969E-05	VMI	6.4235E-06	VAE	1.49683E-06
DCY	3.85969E-05	VTE	6.4235E-06	VCE	1.49683E-06
DMD	3.85969E-05	VWY	6.4235E-06	VDD	1.49683E-06
EMD	3.85969E-05	VYD	6.4235E-06	VDE	1.49683E-06
EMF	3.85969E-05	WCQ	6.4235E-06	VFI	1.49683E-06
ENI	3.85969E-05	WEF	6.4235E-06	VFY	1.49683E-06
EYC	3.85969E-05	WFD	6.4235E-06	VIN	1.49683E-06
FDI	3.85969E-05	WHN	6.4235E-06	VIQ	1.49683E-06
FHF	3.85969E-05	WHV	6.4235E-06	VQK	1.49683E-06
FHY	3.85969E-05	YAE	6.4235E-06	VVY	1.49683E-06
FQN	3.85969E-05	YCQ	6.4235E-06	VWE	1.49683E-06

HCD	3.85969E-05	YED	6.4235E-06	WDK	1.49683E-06
HCH	3.85969E-05	YMH	6.4235E-06	WEV	1.49683E-06
IFC	3.85969E-05	YMK	6.4235E-06	WHM	1.49683E-06
IHM	3.85969E-05	YQH	6.4235E-06	WMI	1.49683E-06
IMH	3.85969E-05	YWI	6.4235E-06	WPE	1.49683E-06
KDI	3.85969E-05	ADK	6.0666E-06	WQM	1.49683E-06
KIN	3.85969E-05	ANC	6.0666E-06	YDM	1.49683E-06
MHH	3.85969E-05	AQK	6.0666E-06	YDN	1.49683E-06
QHF	3.85969E-05	AWW	6.0666E-06	YFK	1.49683E-06
WKN	3.85969E-05	CDD	6.0666E-06	YGA	1.49683E-06
WWH	3.85969E-05	CDG	6.0666E-06	YGF	1.49683E-06
YEQ	3.85969E-05	CEH	6.0666E-06	YGN	1.49683E-06
YEY	3.85969E-05	CKY	6.0666E-06	YIH	1.49683E-06
YFE	3.85969E-05	CLM	6.0666E-06	YMP	1.49683E-06
YHD	3.85969E-05	CNH	6.0666E-06	YNI	1.49683E-06
YHI	3.85969E-05	CTD	6.0666E-06	YNK	1.49683E-06
YQC	3.85969E-05	CWH	6.0666E-06	YYI	1.49683E-06
CDC	3.81632E-05	DCE	6.0666E-06	ACI	1.24736E-06
DFI	3.81632E-05	DCY	6.0666E-06	ADI	1.24736E-06
DHI	3.81632E-05	DEI	6.0666E-06	AFG	1.24736E-06
DQK	3.81632E-05	DQQ	6.0666E-06	AIP	1.24736E-06
EFW	3.81632E-05	DWG	6.0666E-06	ANW	1.24736E-06
EHK	3.81632E-05	DYQ	6.0666E-06	AVF	1.24736E-06
EHN	3.81632E-05	EEG	6.0666E-06	AVW	1.24736E-06
EIQ	3.81632E-05	EIQ	6.0666E-06	AWD	1.24736E-06
EMQ	3.81632E-05	EQK	6.0666E-06	AYW	1.24736E-06
EQW	3.81632E-05	EVW	6.0666E-06	CCD	1.24736E-06
FCI	3.81632E-05	FDD	6.0666E-06	CDP	1.24736E-06
FDE	3.81632E-05	FFK	6.0666E-06	CEV	1.24736E-06
FFD	3.81632E-05	FNE	6.0666E-06	CGF	1.24736E-06
FFK	3.81632E-05	FYC	6.0666E-06	CHF	1.24736E-06
FIH	3.81632E-05	GHE	6.0666E-06	CII	1.24736E-06
FKE	3.81632E-05	GQY	6.0666E-06	CKN	1.24736E-06
FQI	3.81632E-05	HCQ	6.0666E-06	CMH	1.24736E-06
FWY	3.81632E-05	HEA	6.0666E-06	CNN	1.24736E-06
FYI	3.81632E-05	HRK	6.0666E-06	CPE	1.24736E-06
HIH	3.81632E-05	HSY	6.0666E-06	CPT	1.24736E-06
HKH	3.81632E-05	HVW	6.0666E-06	CQM	1.24736E-06
IFH	3.81632E-05	HWY	6.0666E-06	CQN	1.24736E-06
IHH	3.81632E-05	HYF	6.0666E-06	CQT	1.24736E-06
IIM	3.81632E-05	HYV	6.0666E-06	CVI	1.24736E-06
IIQ	3.81632E-05	ICM	6.0666E-06	CVM	1.24736E-06
KEM	3.81632E-05	IED	6.0666E-06	CVT	1.24736E-06
KHF	3.81632E-05	III	6.0666E-06	DCD	1.24736E-06

KII	3.81632E-05	IIW	6.0666E-06	DCF	1.24736E-06
MDW	3.81632E-05	KDI	6.0666E-06	DIG	1.24736E-06
MNN	3.81632E-05	KEV	6.0666E-06	DIY	1.24736E-06
NCN	3.81632E-05	KFE	6.0666E-06	DKV	1.24736E-06
NWC	3.81632E-05	KHW	6.0666E-06	DMW	1.24736E-06
QCD	3.81632E-05	KMN	6.0666E-06	DNI	1.24736E-06
QYW	3.81632E-05	KQD	6.0666E-06	DQD	1.24736E-06
WYE	3.81632E-05	KWM	6.0666E-06	DQF	1.24736E-06
YCN	3.81632E-05	MVV	6.0666E-06	DQI	1.24736E-06
YIK	3.81632E-05	MWF	6.0666E-06	DRC	1.24736E-06
YKN	3.81632E-05	NDC	6.0666E-06	DRN	1.24736E-06
CCN	3.77295E-05	NEI	6.0666E-06	DSK	1.24736E-06
CCW	3.77295E-05	NHE	6.0666E-06	DVI	1.24736E-06
CDY	3.77295E-05	NII	6.0666E-06	DVN	1.24736E-06
CFW	3.77295E-05	NVF	6.0666E-06	DYM	1.24736E-06
CIQ	3.77295E-05	NWK	6.0666E-06	ECG	1.24736E-06
CQH	3.77295E-05	PMM	6.0666E-06	EEI	1.24736E-06
CYH	3.77295E-05	PQM	6.0666E-06	EFM	1.24736E-06
DFN	3.77295E-05	PWQ	6.0666E-06	EHI	1.24736E-06
DIN	3.77295E-05	QDY	6.0666E-06	EHM	1.24736E-06
DYD	3.77295E-05	QFQ	6.0666E-06	EIG	1.24736E-06
EDE	3.77295E-05	QMF	6.0666E-06	EII	1.24736E-06
EDF	3.77295E-05	SKW	6.0666E-06	EKM	1.24736E-06
EFY	3.77295E-05	SMW	6.0666E-06	EMK	1.24736E-06
EIY	3.77295E-05	SQN	6.0666E-06	EQF	1.24736E-06
EKK	3.77295E-05	TDY	6.0666E-06	ERE	1.24736E-06
EKW	3.77295E-05	TMW	6.0666E-06	EVE	1.24736E-06
EMY	3.77295E-05	VDD	6.0666E-06	EVP	1.24736E-06
EQC	3.77295E-05	VDH	6.0666E-06	FIE	1.24736E-06
FEH	3.77295E-05	VFW	6.0666E-06	GAM	1.24736E-06
FII	3.77295E-05	VIC	6.0666E-06	HAV	1.24736E-06
FQC	3.77295E-05	WAK	6.0666E-06	HCF	1.24736E-06
FQD	3.77295E-05	WCA	6.0666E-06	HCH	1.24736E-06
HFF	3.77295E-05	WIY	6.0666E-06	HCI	1.24736E-06
HWQ	3.77295E-05	WME	6.0666E-06	HDF	1.24736E-06
HYI	3.77295E-05	WMI	6.0666E-06	HDM	1.24736E-06
INI	3.77295E-05	WQG	6.0666E-06	HFD	1.24736E-06
KCD	3.77295E-05	WVF	6.0666E-06	HGE	1.24736E-06
KCN	3.77295E-05	WWV	6.0666E-06	HGT	1.24736E-06
KCQ	3.77295E-05	YFQ	6.0666E-06	HIA	1.24736E-06
KHE	3.77295E-05	YMC	6.0666E-06	HLF	1.24736E-06
KMF	3.77295E-05	YMD	6.0666E-06	HMH	1.24736E-06
KMY	3.77295E-05	YWK	6.0666E-06	HMP	1.24736E-06
KYQ	3.77295E-05	ACY	5.7098E-06	HNM	1.24736E-06

MKE	3.77295E-05	AIK	5.7098E-06	HNV	1.24736E-06
MNH	3.77295E-05	AIM	5.7098E-06	HPF	1.24736E-06
NDY	3.77295E-05	CAW	5.7098E-06	HQY	1.24736E-06
NEI	3.77295E-05	CEQ	5.7098E-06	HVM	1.24736E-06
NQM	3.77295E-05	CHG	5.7098E-06	HVN	1.24736E-06
NWK	3.77295E-05	CHT	5.7098E-06	HWE	1.24736E-06
QEQ	3.77295E-05	CKW	5.7098E-06	HWI	1.24736E-06
QFC	3.77295E-05	CNM	5.7098E-06	HYI	1.24736E-06
QND	3.77295E-05	CRD	5.7098E-06	ICY	1.24736E-06
QQW	3.77295E-05	CTG	5.7098E-06	IDC	1.24736E-06
WWQ	3.77295E-05	CYN	5.7098E-06	IEG	1.24736E-06
YEW	3.77295E-05	DCQ	5.7098E-06	IHW	1.24736E-06
YID	3.77295E-05	DEE	5.7098E-06	IIF	1.24736E-06
YKH	3.77295E-05	DEV	5.7098E-06	IIY	1.24736E-06
YQN	3.77295E-05	ECM	5.7098E-06	ILF	1.24736E-06
CKI	3.72959E-05	EFF	5.7098E-06	IPF	1.24736E-06
DCE	3.72959E-05	EFK	5.7098E-06	IPN	1.24736E-06
DFF	3.72959E-05	EQC	5.7098E-06	IVE	1.24736E-06
DMN	3.72959E-05	EQW	5.7098E-06	IVP	1.24736E-06
DNI	3.72959E-05	EWC	5.7098E-06	IWD	1.24736E-06
DNY	3.72959E-05	EYC	5.7098E-06	IYF	1.24736E-06
EEM	3.72959E-05	FIC	5.7098E-06	IYK	1.24736E-06
EEQ	3.72959E-05	FII	5.7098E-06	KDV	1.24736E-06
EHE	3.72959E-05	FVE	5.7098E-06	KFF	1.24736E-06
EII	3.72959E-05	GYK	5.7098E-06	KFV	1.24736E-06
EKC	3.72959E-05	HDF	5.7098E-06	KGF	1.24736E-06
EQE	3.72959E-05	HIL	5.7098E-06	KGM	1.24736E-06
EYH	3.72959E-05	IGY	5.7098E-06	KIE	1.24736E-06
FND	3.72959E-05	IKE	5.7098E-06	KIL	1.24736E-06
FQK	3.72959E-05	IPI	5.7098E-06	KKE	1.24736E-06
HKE	3.72959E-05	IVM	5.7098E-06	KME	1.24736E-06
IEK	3.72959E-05	IVP	5.7098E-06	KMQ	1.24736E-06
IKW	3.72959E-05	KDE	5.7098E-06	KRK	1.24736E-06
IME	3.72959E-05	KHM	5.7098E-06	KRM	1.24736E-06
IMW	3.72959E-05	MEM	5.7098E-06	KTE	1.24736E-06
IYQ	3.72959E-05	MEY	5.7098E-06	KVV	1.24736E-06
KFK	3.72959E-05	MFD	5.7098E-06	KWE	1.24736E-06
KIY	3.72959E-05	MKA	5.7098E-06	KWW	1.24736E-06
NMK	3.72959E-05	MMK	5.7098E-06	KYF	1.24736E-06
NYN	3.72959E-05	MQC	5.7098E-06	KYI	1.24736E-06
QMK	3.72959E-05	MQG	5.7098E-06	KYQ	1.24736E-06
QQH	3.72959E-05	MVH	5.7098E-06	MAC	1.24736E-06
QYK	3.72959E-05	MWN	5.7098E-06	MAY	1.24736E-06
WCW	3.72959E-05	MYW	5.7098E-06	MCA	1.24736E-06

WEM	3.72959E-05	NCE	5.7098E-06	MCH	1.24736E-06
WHQ	3.72959E-05	NVW	5.7098E-06	MDC	1.24736E-06
WKK	3.72959E-05	NWM	5.7098E-06	MDG	1.24736E-06
WKM	3.72959E-05	NWW	5.7098E-06	MEV	1.24736E-06
WMF	3.72959E-05	PEK	5.7098E-06	MFD	1.24736E-06
WMQ	3.72959E-05	PMI	5.7098E-06	MFH	1.24736E-06
YME	3.72959E-05	QDE	5.7098E-06	MFQ	1.24736E-06
YQK	3.72959E-05	QFV	5.7098E-06	MHN	1.24736E-06
YYI	3.72959E-05	QIC	5.7098E-06	MHR	1.24736E-06
CCC	3.68622E-05	QKM	5.7098E-06	MHV	1.24736E-06
CFK	3.68622E-05	QLK	5.7098E-06	MIK	1.24736E-06
CFM	3.68622E-05	QRK	5.7098E-06	MMP	1.24736E-06
CHK	3.68622E-05	QVY	5.7098E-06	MRE	1.24736E-06
CIH	3.68622E-05	QWW	5.7098E-06	MSE	1.24736E-06
CNE	3.68622E-05	RIM	5.7098E-06	MTQ	1.24736E-06
CYI	3.68622E-05	RQY	5.7098E-06	MVH	1.24736E-06
DEE	3.68622E-05	SQE	5.7098E-06	MWY	1.24736E-06
DWD	3.68622E-05	TEC	5.7098E-06	MYG	1.24736E-06
ECW	3.68622E-05	THK	5.7098E-06	MYH	1.24736E-06
EEN	3.68622E-05	VEW	5.7098E-06	MYR	1.24736E-06
EHY	3.68622E-05	VIN	5.7098E-06	NAE	1.24736E-06
EIE	3.68622E-05	VPI	5.7098E-06	NCM	1.24736E-06
EYK	3.68622E-05	VQH	5.7098E-06	NDK	1.24736E-06
EYN	3.68622E-05	VYM	5.7098E-06	NFF	1.24736E-06
FFQ	3.68622E-05	WDY	5.7098E-06	NGF	1.24736E-06
HFY	3.68622E-05	WYD	5.7098E-06	NGV	1.24736E-06
HHD	3.68622E-05	YCD	5.7098E-06	NHN	1.24736E-06
HNI	3.68622E-05	YCP	5.7098E-06	NHW	1.24736E-06
IDQ	3.68622E-05	YFN	5.7098E-06	NIN	1.24736E-06
IHN	3.68622E-05	YHM	5.7098E-06	NTE	1.24736E-06
III	3.68622E-05	YWM	5.7098E-06	NVF	1.24736E-06
IKN	3.68622E-05	YWN	5.7098E-06	NYQ	1.24736E-06
IMQ	3.68622E-05	YWQ	5.7098E-06	PDK	1.24736E-06
IYW	3.68622E-05	YYI	5.7098E-06	PQI	1.24736E-06
KFD	3.68622E-05	AHC	5.3529E-06	QAE	1.24736E-06
KNF	3.68622E-05	CAQ	5.3529E-06	QCV	1.24736E-06
KYE	3.68622E-05	CFK	5.3529E-06	QDI	1.24736E-06
MMC	3.68622E-05	CGK	5.3529E-06	QEF	1.24736E-06
NHQ	3.68622E-05	CIH	5.3529E-06	QEK	1.24736E-06
NWN	3.68622E-05	CIL	5.3529E-06	QEV	1.24736E-06
QEC	3.68622E-05	CKF	5.3529E-06	QFV	1.24736E-06
QFH	3.68622E-05	CKG	5.3529E-06	QHI	1.24736E-06
QKE	3.68622E-05	CNC	5.3529E-06	QII	1.24736E-06
QQC	3.68622E-05	CNG	5.3529E-06	QKI	1.24736E-06

WHK	3.68622E-05	CNV	5.3529E-06	QKK	1.24736E-06
WIN	3.68622E-05	CPE	5.3529E-06	QMD	1.24736E-06
WNW	3.68622E-05	CRC	5.3529E-06	QMP	1.24736E-06
WYD	3.68622E-05	CTM	5.3529E-06	QTK	1.24736E-06
YCK	3.68622E-05	CWQ	5.3529E-06	QWF	1.24736E-06
YMY	3.68622E-05	CYH	5.3529E-06	QWM	1.24736E-06
YNF	3.68622E-05	DCP	5.3529E-06	RCM	1.24736E-06
YWH	3.68622E-05	DDC	5.3529E-06	RIM	1.24736E-06
YWQ	3.68622E-05	DED	5.3529E-06	SAI	1.24736E-06
CNQ	3.64285E-05	DME	5.3529E-06	SCI	1.24736E-06
DII	3.64285E-05	DQN	5.3529E-06	SCK	1.24736E-06
DQY	3.64285E-05	DVD	5.3529E-06	SDN	1.24736E-06
DWY	3.64285E-05	DYM	5.3529E-06	SEI	1.24736E-06
ECH	3.64285E-05	DYN	5.3529E-06	TCH	1.24736E-06
ECQ	3.64285E-05	EFM	5.3529E-06	TCI	1.24736E-06
EQH	3.64285E-05	EFQ	5.3529E-06	TEK	1.24736E-06
HYF	3.64285E-05	EHE	5.3529E-06	TFE	1.24736E-06
IDF	3.64285E-05	EHK	5.3529E-06	THQ	1.24736E-06
IEE	3.64285E-05	EWQ	5.3529E-06	TQF	1.24736E-06
IFN	3.64285E-05	FDE	5.3529E-06	TQW	1.24736E-06
IKC	3.64285E-05	FDN	5.3529E-06	TVK	1.24736E-06
IND	3.64285E-05	FME	5.3529E-06	TVV	1.24736E-06
INQ	3.64285E-05	FYY	5.3529E-06	TWF	1.24736E-06
IYH	3.64285E-05	GHK	5.3529E-06	TYN	1.24736E-06
KKF	3.64285E-05	GQK	5.3529E-06	VDI	1.24736E-06
KYC	3.64285E-05	HCN	5.3529E-06	VEI	1.24736E-06
MMQ	3.64285E-05	HCP	5.3529E-06	VFF	1.24736E-06
MNM	3.64285E-05	HGI	5.3529E-06	VFK	1.24736E-06
MYW	3.64285E-05	HHW	5.3529E-06	VFN	1.24736E-06
QQN	3.64285E-05	HIC	5.3529E-06	VIH	1.24736E-06
QWM	3.64285E-05	HKF	5.3529E-06	VMV	1.24736E-06
QYQ	3.64285E-05	HKI	5.3529E-06	VND	1.24736E-06
WCM	3.64285E-05	HKK	5.3529E-06	VVC	1.24736E-06
WCY	3.64285E-05	HPK	5.3529E-06	VVE	1.24736E-06
WHY	3.64285E-05	HQN	5.3529E-06	VVP	1.24736E-06
WYQ	3.64285E-05	HQQ	5.3529E-06	VYI	1.24736E-06
YEI	3.64285E-05	HQW	5.3529E-06	WAM	1.24736E-06
YIH	3.64285E-05	HWK	5.3529E-06	WCK	1.24736E-06
YMF	3.64285E-05	HYC	5.3529E-06	WDI	1.24736E-06
CDF	3.59949E-05	ICK	5.3529E-06	WEM	1.24736E-06
CFN	3.59949E-05	IDM	5.3529E-06	WIM	1.24736E-06
CIC	3.59949E-05	IEV	5.3529E-06	WVI	1.24736E-06
CKC	3.59949E-05	IFK	5.3529E-06	YKK	1.24736E-06
CME	3.59949E-05	IKG	5.3529E-06	YMA	1.24736E-06

DED	3.59949E-05	IMC	5.3529E-06	YMK	1.24736E-06
DHF	3.59949E-05	IQI	5.3529E-06	YVA	1.24736E-06
EEY	3.59949E-05	IWE	5.3529E-06	YVE	1.24736E-06
EME	3.59949E-05	IYE	5.3529E-06	YYK	1.24736E-06
EQM	3.59949E-05	IYI	5.3529E-06	ACM	9.97885E-07
EYF	3.59949E-05	IYN	5.3529E-06	ACQ	9.97885E-07
FDQ	3.59949E-05	KEI	5.3529E-06	ACV	9.97885E-07
FFI	3.59949E-05	KHE	5.3529E-06	AGG	9.97885E-07
FIF	3.59949E-05	KVF	5.3529E-06	AIK	9.97885E-07
FQF	3.59949E-05	KYI	5.3529E-06	AQW	9.97885E-07
HCI	3.59949E-05	LDK	5.3529E-06	AVA	9.97885E-07
HID	3.59949E-05	MCI	5.3529E-06	AWE	9.97885E-07
HQN	3.59949E-05	MDG	5.3529E-06	AWF	9.97885E-07
HWY	3.59949E-05	MIN	5.3529E-06	AWY	9.97885E-07
IMK	3.59949E-05	MKV	5.3529E-06	CAN	9.97885E-07
INH	3.59949E-05	MME	5.3529E-06	CAW	9.97885E-07
IYN	3.59949E-05	MTQ	5.3529E-06	CCE	9.97885E-07
KEI	3.59949E-05	MVA	5.3529E-06	CCI	9.97885E-07
KEY	3.59949E-05	MYH	5.3529E-06	CEE	9.97885E-07
KWD	3.59949E-05	NDM	5.3529E-06	CGC	9.97885E-07
KWK	3.59949E-05	NIC	5.3529E-06	CGI	9.97885E-07
KYN	3.59949E-05	NKE	5.3529E-06	CHI	9.97885E-07
MEM	3.59949E-05	QEN	5.3529E-06	CHK	9.97885E-07
MHK	3.59949E-05	QFD	5.3529E-06	CHM	9.97885E-07
MKD	3.59949E-05	QFE	5.3529E-06	CID	9.97885E-07
MNC	3.59949E-05	QFM	5.3529E-06	CIK	9.97885E-07
NDF	3.59949E-05	QIA	5.3529E-06	CIM	9.97885E-07
NWE	3.59949E-05	QID	5.3529E-06	CIT	9.97885E-07
QDD	3.59949E-05	QII	5.3529E-06	CIY	9.97885E-07
QEM	3.59949E-05	QMG	5.3529E-06	CKD	9.97885E-07
QIM	3.59949E-05	QQY	5.3529E-06	CKI	9.97885E-07
QNY	3.59949E-05	QWY	5.3529E-06	CMD	9.97885E-07
WFM	3.59949E-05	RHW	5.3529E-06	CMK	9.97885E-07
WND	3.59949E-05	SHW	5.3529E-06	CND	9.97885E-07
YQH	3.59949E-05	SQM	5.3529E-06	CNM	9.97885E-07
CCH	3.55612E-05	TQK	5.3529E-06	CPK	9.97885E-07
CCQ	3.55612E-05	VQG	5.3529E-06	CPW	9.97885E-07
CEI	3.55612E-05	WKM	5.3529E-06	CQY	9.97885E-07
CEM	3.55612E-05	WNE	5.3529E-06	CRI	9.97885E-07
CQM	3.55612E-05	WQN	5.3529E-06	CRK	9.97885E-07
DDE	3.55612E-05	WQW	5.3529E-06	CTE	9.97885E-07
DEF	3.55612E-05	YDM	5.3529E-06	CTI	9.97885E-07
DWQ	3.55612E-05	YFD	5.3529E-06	CTQ	9.97885E-07
EFI	3.55612E-05	YQM	5.3529E-06	CTV	9.97885E-07

EHW	3.55612E-05	AQC	4.9961E-06	CVE	9.97885E-07
EIK	3.55612E-05	CFV	4.9961E-06	CVP	9.97885E-07
EQF	3.55612E-05	CGI	4.9961E-06	CWD	9.97885E-07
EWC	3.55612E-05	CNN	4.9961E-06	CYK	9.97885E-07
FCK	3.55612E-05	CRE	4.9961E-06	CYN	9.97885E-07
HCY	3.55612E-05	DDE	4.9961E-06	DAI	9.97885E-07
HEE	3.55612E-05	DEC	4.9961E-06	DDM	9.97885E-07
HIF	3.55612E-05	DFI	4.9961E-06	DFK	9.97885E-07
IKE	3.55612E-05	DHM	4.9961E-06	DFV	9.97885E-07
IYC	3.55612E-05	DQM	4.9961E-06	DFY	9.97885E-07
KHI	3.55612E-05	DVF	4.9961E-06	DGG	9.97885E-07
MHC	3.55612E-05	DWE	4.9961E-06	DHY	9.97885E-07
MMH	3.55612E-05	EFE	4.9961E-06	DIA	9.97885E-07
MNW	3.55612E-05	EIN	4.9961E-06	DIF	9.97885E-07
MYC	3.55612E-05	EKK	4.9961E-06	DIH	9.97885E-07
NEY	3.55612E-05	EVD	4.9961E-06	DIV	9.97885E-07
NQN	3.55612E-05	EYK	4.9961E-06	DNE	9.97885E-07
QED	3.55612E-05	FCE	4.9961E-06	DNK	9.97885E-07
QKN	3.55612E-05	FDM	4.9961E-06	DPK	9.97885E-07
QNI	3.55612E-05	FEG	4.9961E-06	DVV	9.97885E-07
WDE	3.55612E-05	FFD	4.9961E-06	DWF	9.97885E-07
WED	3.55612E-05	FIM	4.9961E-06	DWN	9.97885E-07
WHD	3.55612E-05	FVF	4.9961E-06	DYK	9.97885E-07
WHW	3.55612E-05	GQC	4.9961E-06	EAV	9.97885E-07
WWK	3.55612E-05	HME	4.9961E-06	ECC	9.97885E-07
YFD	3.55612E-05	HMF	4.9961E-06	ECF	9.97885E-07
YHQ	3.55612E-05	HQC	4.9961E-06	EFE	9.97885E-07
YMD	3.55612E-05	HQY	4.9961E-06	EGA	9.97885E-07
YQW	3.55612E-05	IAI	4.9961E-06	EGC	9.97885E-07
YWN	3.55612E-05	ICF	4.9961E-06	EGF	9.97885E-07
CCM	3.51275E-05	IDP	4.9961E-06	EGM	9.97885E-07
CHW	3.51275E-05	IEY	4.9961E-06	EIW	9.97885E-07
CIF	3.51275E-05	IFD	4.9961E-06	ERI	9.97885E-07
CMH	3.51275E-05	IFF	4.9961E-06	EVG	9.97885E-07
DKY	3.51275E-05	IFN	4.9961E-06	EWE	9.97885E-07
DQI	3.51275E-05	IFY	4.9961E-06	EWF	9.97885E-07
DYK	3.51275E-05	IIC	4.9961E-06	EWK	9.97885E-07
EFH	3.51275E-05	IVW	4.9961E-06	EWM	9.97885E-07
EQN	3.51275E-05	KIC	4.9961E-06	EWS	9.97885E-07
FDY	3.51275E-05	KMC	4.9961E-06	EYI	9.97885E-07
FIN	3.51275E-05	KQF	4.9961E-06	EYM	9.97885E-07
IHC	3.51275E-05	KQQ	4.9961E-06	FDD	9.97885E-07
IIH	3.51275E-05	KYM	4.9961E-06	FDE	9.97885E-07
IMD	3.51275E-05	MGK	4.9961E-06	FDK	9.97885E-07

INC	3.51275E-05	MIH	4.9961E-06	FDN	9.97885E-07
INW	3.51275E-05	MMV	4.9961E-06	GFE	9.97885E-07
IQM	3.51275E-05	MYC	4.9961E-06	HAK	9.97885E-07
KID	3.51275E-05	MYF	4.9961E-06	HAT	9.97885E-07
KIQ	3.51275E-05	NFD	4.9961E-06	HEF	9.97885E-07
KQW	3.51275E-05	NQM	4.9961E-06	HEM	9.97885E-07
KYH	3.51275E-05	NYI	4.9961E-06	HFK	9.97885E-07
MDD	3.51275E-05	PQK	4.9961E-06	HGK	9.97885E-07
QCE	3.51275E-05	PVK	4.9961E-06	HGY	9.97885E-07
QCQ	3.51275E-05	PYY	4.9961E-06	HIE	9.97885E-07
QMN	3.51275E-05	QEF	4.9961E-06	HIH	9.97885E-07
QMY	3.51275E-05	QMC	4.9961E-06	HKE	9.97885E-07
QWE	3.51275E-05	QMW	4.9961E-06	HKN	9.97885E-07
QYM	3.51275E-05	QQQ	4.9961E-06	HKV	9.97885E-07
WDH	3.51275E-05	QVG	4.9961E-06	HMA	9.97885E-07
WFI	3.51275E-05	QYQ	4.9961E-06	HMC	9.97885E-07
WIC	3.51275E-05	TIW	4.9961E-06	HMI	9.97885E-07
WIM	3.51275E-05	TQY	4.9961E-06	HMV	9.97885E-07
WMW	3.51275E-05	VCM	4.9961E-06	HNI	9.97885E-07
YCY	3.51275E-05	VDW	4.9961E-06	HPM	9.97885E-07
YEE	3.51275E-05	VIM	4.9961E-06	HRE	9.97885E-07
YIY	3.51275E-05	VIW	4.9961E-06	HRF	9.97885E-07
YWF	3.51275E-05	VMC	4.9961E-06	HSE	9.97885E-07
CNW	3.46938E-05	WFM	4.9961E-06	HVG	9.97885E-07
DCI	3.46938E-05	WWM	4.9961E-06	HYF	9.97885E-07
DWN	3.46938E-05	YCK	4.9961E-06	IDD	9.97885E-07
ECF	3.46938E-05	YDK	4.9961E-06	IDY	9.97885E-07
ECN	3.46938E-05	YDN	4.9961E-06	IEC	9.97885E-07
EEE	3.46938E-05	YIK	4.9961E-06	IEF	9.97885E-07
EWH	3.46938E-05	YMM	4.9961E-06	IFQ	9.97885E-07
EWN	3.46938E-05	YWP	4.9961E-06	IGE	9.97885E-07
EYQ	3.46938E-05	YYD	4.9961E-06	IHE	9.97885E-07
FWN	3.46938E-05	ACM	4.6392E-06	IIH	9.97885E-07
HEY	3.46938E-05	AMW	4.6392E-06	IPI	9.97885E-07
HWD	3.46938E-05	AVF	4.6392E-06	IQK	9.97885E-07
IEW	3.46938E-05	CAM	4.6392E-06	IQW	9.97885E-07
IFQ	3.46938E-05	CCA	4.6392E-06	IVC	9.97885E-07
IIW	3.46938E-05	CFG	4.6392E-06	IVF	9.97885E-07
IMI	3.46938E-05	CHH	4.6392E-06	IWE	9.97885E-07
IQH	3.46938E-05	CHQ	4.6392E-06	IYD	9.97885E-07
IWK	3.46938E-05	CHV	4.6392E-06	KCE	9.97885E-07
KCW	3.46938E-05	CIG	4.6392E-06	KCW	9.97885E-07
KWI	3.46938E-05	CKD	4.6392E-06	KDK	9.97885E-07
KYY	3.46938E-05	CMK	4.6392E-06	KEI	9.97885E-07

MEG	3.46938E-05	CNY	4.6392E-06	KEK	9.97885E-07
MIN	3.46938E-05	CPN	4.6392E-06	KEM	9.97885E-07
MQK	3.46938E-05	CQF	4.6392E-06	KEV	9.97885E-07
NFN	3.46938E-05	CRY	4.6392E-06	KGV	9.97885E-07
NKY	3.46938E-05	CYG	4.6392E-06	KHN	9.97885E-07
NWI	3.46938E-05	CYV	4.6392E-06	KIF	9.97885E-07
QCN	3.46938E-05	DEM	4.6392E-06	KII	9.97885E-07
QDH	3.46938E-05	DFK	4.6392E-06	KIV	9.97885E-07
QWC	3.46938E-05	DKN	4.6392E-06	KIW	9.97885E-07
WEH	3.46938E-05	DNK	4.6392E-06	KMK	9.97885E-07
WEQ	3.46938E-05	DWQ	4.6392E-06	KNI	9.97885E-07
YCD	3.46938E-05	EII	4.6392E-06	KQC	9.97885E-07
CEK	3.42602E-05	EKG	4.6392E-06	KQD	9.97885E-07
CHQ	3.42602E-05	EWG	4.6392E-06	KQW	9.97885E-07
CKK	3.42602E-05	FEK	4.6392E-06	KQY	9.97885E-07
CMK	3.42602E-05	FHW	4.6392E-06	KVI	9.97885E-07
CNF	3.42602E-05	FHY	4.6392E-06	MDF	9.97885E-07
CYD	3.42602E-05	HIG	4.6392E-06	MDN	9.97885E-07
DFH	3.42602E-05	HIY	4.6392E-06	MDP	9.97885E-07
DQH	3.42602E-05	HMM	4.6392E-06	MDV	9.97885E-07
ECD	3.42602E-05	HQF	4.6392E-06	MDW	9.97885E-07
EDD	3.42602E-05	HVQ	4.6392E-06	MEC	9.97885E-07
EFK	3.42602E-05	HWM	4.6392E-06	MEW	9.97885E-07
ENN	3.42602E-05	HWN	4.6392E-06	MFA	9.97885E-07
EWW	3.42602E-05	HYM	4.6392E-06	MFF	9.97885E-07
FFF	3.42602E-05	IEQ	4.6392E-06	MFY	9.97885E-07
FID	3.42602E-05	IWC	4.6392E-06	MGA	9.97885E-07
FKF	3.42602E-05	IWI	4.6392E-06	MHY	9.97885E-07
HIK	3.42602E-05	KHI	4.6392E-06	MIY	9.97885E-07
HMF	3.42602E-05	KIW	4.6392E-06	MKM	9.97885E-07
HQY	3.42602E-05	MEF	4.6392E-06	MMF	9.97885E-07
ICC	3.42602E-05	MIE	4.6392E-06	MQP	9.97885E-07
IHE	3.42602E-05	MQH	4.6392E-06	MVM	9.97885E-07
IKK	3.42602E-05	MVE	4.6392E-06	MVW	9.97885E-07
IWW	3.42602E-05	MYM	4.6392E-06	MWE	9.97885E-07
KCH	3.42602E-05	NEC	4.6392E-06	MYV	9.97885E-07
KFF	3.42602E-05	NKW	4.6392E-06	NAV	9.97885E-07
KFI	3.42602E-05	NQY	4.6392E-06	NCE	9.97885E-07
MKN	3.42602E-05	QEA	4.6392E-06	NCF	9.97885E-07
MMW	3.42602E-05	QEI	4.6392E-06	NCK	9.97885E-07
MYN	3.42602E-05	QFK	4.6392E-06	NCQ	9.97885E-07
NIY	3.42602E-05	QIQ	4.6392E-06	NDI	9.97885E-07
NWD	3.42602E-05	QIV	4.6392E-06	NEV	9.97885E-07
NWY	3.42602E-05	QKI	4.6392E-06	NHY	9.97885E-07

NYF	3.42602E-05	QQF	4.6392E-06	NIC	9.97885E-07
QCY	3.42602E-05	QQN	4.6392E-06	NQC	9.97885E-07
QDQ	3.42602E-05	SQW	4.6392E-06	NQK	9.97885E-07
QIE	3.42602E-05	TEW	4.6392E-06	NQW	9.97885E-07
QMI	3.42602E-05	TQC	4.6392E-06	NYE	9.97885E-07
WCN	3.42602E-05	VCY	4.6392E-06	PDN	9.97885E-07
WDK	3.42602E-05	VDY	4.6392E-06	PEF	9.97885E-07
WFH	3.42602E-05	VFY	4.6392E-06	PEV	9.97885E-07
WNI	3.42602E-05	VHW	4.6392E-06	PFE	9.97885E-07
WQH	3.42602E-05	VKD	4.6392E-06	PHK	9.97885E-07
WQW	3.42602E-05	WFK	4.6392E-06	PQF	9.97885E-07
WQY	3.42602E-05	WQI	4.6392E-06	PYM	9.97885E-07
YCF	3.42602E-05	WWD	4.6392E-06	QAK	9.97885E-07
YQI	3.42602E-05	WWW	4.6392E-06	QAP	9.97885E-07
YYD	3.42602E-05	WYE	4.6392E-06	QCF	9.97885E-07
CDI	3.38265E-05	WYY	4.6392E-06	QDD	9.97885E-07
CEH	3.38265E-05	YDE	4.6392E-06	QED	9.97885E-07
CEN	3.38265E-05	YIQ	4.6392E-06	QEE	9.97885E-07
CKH	3.38265E-05	YKK	4.6392E-06	QFI	9.97885E-07
CKN	3.38265E-05	YNE	4.6392E-06	QFW	9.97885E-07
CWI	3.38265E-05	YNK	4.6392E-06	QGM	9.97885E-07
CYN	3.38265E-05	CAK	4.2823E-06	QHY	9.97885E-07
DCF	3.38265E-05	CFY	4.2823E-06	QKD	9.97885E-07
DEI	3.38265E-05	CVC	4.2823E-06	QKE	9.97885E-07
DYE	3.38265E-05	CVM	4.2823E-06	QMM	9.97885E-07
ECI	3.38265E-05	CWC	4.2823E-06	QMQ	9.97885E-07
EKD	3.38265E-05	CWV	4.2823E-06	QQM	9.97885E-07
EMH	3.38265E-05	CYD	4.2823E-06	QRM	9.97885E-07
FKI	3.38265E-05	CYM	4.2823E-06	QVN	9.97885E-07
FMF	3.38265E-05	DDD	4.2823E-06	QVP	9.97885E-07
HQF	3.38265E-05	DEK	4.2823E-06	QVQ	9.97885E-07
IID	3.38265E-05	DMQ	4.2823E-06	QVV	9.97885E-07
IKI	3.38265E-05	DVI	4.2823E-06	RDK	9.97885E-07
KED	3.38265E-05	DYV	4.2823E-06	REM	9.97885E-07
KIF	3.38265E-05	EKW	4.2823E-06	RNM	9.97885E-07
KYF	3.38265E-05	EWY	4.2823E-06	TCD	9.97885E-07
MIM	3.38265E-05	EYE	4.2823E-06	TCF	9.97885E-07
MKW	3.38265E-05	EYY	4.2823E-06	TDE	9.97885E-07
MNE	3.38265E-05	FID	4.2823E-06	TDM	9.97885E-07
MYQ	3.38265E-05	HEK	4.2823E-06	TDQ	9.97885E-07
NCF	3.38265E-05	HEN	4.2823E-06	TDV	9.97885E-07
NCY	3.38265E-05	HFE	4.2823E-06	TEG	9.97885E-07
NQD	3.38265E-05	HHF	4.2823E-06	TEW	9.97885E-07
QCM	3.38265E-05	HIF	4.2823E-06	TFD	9.97885E-07

QFM	3.38265E-05	HIM	4.2823E-06	TFI	9.97885E-07
QIF	3.38265E-05	HWQ	4.2823E-06	TFN	9.97885E-07
QWW	3.38265E-05	IDQ	4.2823E-06	TFQ	9.97885E-07
WCK	3.38265E-05	IFI	4.2823E-06	THW	9.97885E-07
WEE	3.38265E-05	IHK	4.2823E-06	TIE	9.97885E-07
WEN	3.38265E-05	IIQ	4.2823E-06	TIP	9.97885E-07
WFN	3.38265E-05	IQC	4.2823E-06	TKI	9.97885E-07
WKY	3.38265E-05	IQK	4.2823E-06	TNQ	9.97885E-07
WNN	3.38265E-05	IVH	4.2823E-06	TQN	9.97885E-07
WQM	3.38265E-05	IVY	4.2823E-06	TQY	9.97885E-07
YYE	3.38265E-05	IWF	4.2823E-06	TYF	9.97885E-07
CHC	3.33928E-05	KMW	4.2823E-06	VDK	9.97885E-07
CYK	3.33928E-05	MCM	4.2823E-06	VFD	9.97885E-07
DKF	3.33928E-05	MCY	4.2823E-06	VHE	9.97885E-07
DQD	3.33928E-05	MDC	4.2823E-06	VHV	9.97885E-07
EQD	3.33928E-05	MHK	4.2823E-06	VIA	9.97885E-07
EWK	3.33928E-05	MHY	4.2823E-06	VVD	9.97885E-07
FCF	3.33928E-05	MIW	4.2823E-06	VVN	9.97885E-07
FDF	3.33928E-05	MIY	4.2823E-06	VYE	9.97885E-07
FQW	3.33928E-05	MMP	4.2823E-06	WAV	9.97885E-07
HKY	3.33928E-05	MNK	4.2823E-06	WIK	9.97885E-07
HWF	3.33928E-05	MQD	4.2823E-06	WME	9.97885E-07
HWN	3.33928E-05	MWI	4.2823E-06	YAM	9.97885E-07
IDE	3.33928E-05	MYQ	4.2823E-06	YDK	9.97885E-07
IDN	3.33928E-05	MYY	4.2823E-06	YEI	9.97885E-07
IHD	3.33928E-05	NDI	4.2823E-06	YPK	9.97885E-07
IHI	3.33928E-05	NEE	4.2823E-06	YQI	9.97885E-07
IKQ	3.33928E-05	NEK	4.2823E-06	YQN	9.97885E-07
IWD	3.33928E-05	NHW	4.2823E-06	ACK	7.48414E-07
KWN	3.33928E-05	NIH	4.2823E-06	ADN	7.48414E-07
NEM	3.33928E-05	NMC	4.2823E-06	AFD	7.48414E-07
QCI	3.33928E-05	NQI	4.2823E-06	AFK	7.48414E-07
QDE	3.33928E-05	NWE	4.2823E-06	AIW	7.48414E-07
QQM	3.33928E-05	NYY	4.2823E-06	ANE	7.48414E-07
QYH	3.33928E-05	QCM	4.2823E-06	CAE	7.48414E-07
WCE	3.33928E-05	QEC	4.2823E-06	CCN	7.48414E-07
WDY	3.33928E-05	QHI	4.2823E-06	CDM	7.48414E-07
WMI	3.33928E-05	QIE	4.2823E-06	CDN	7.48414E-07
WQK	3.33928E-05	QKD	4.2823E-06	CFD	7.48414E-07
YEN	3.33928E-05	QMA	4.2823E-06	CFE	7.48414E-07
YIQ	3.33928E-05	TEF	4.2823E-06	CFY	7.48414E-07
YQE	3.33928E-05	TQF	4.2823E-06	CIF	7.48414E-07
CCD	3.29591E-05	TQN	4.2823E-06	CIH	7.48414E-07
CDQ	3.29591E-05	TQQ	4.2823E-06	CKK	7.48414E-07

CND	3.29591E-05	VCK	4.2823E-06	CKV	7.48414E-07
CYM	3.29591E-05	VEK	4.2823E-06	CMA	7.48414E-07
DFD	3.29591E-05	VFD	4.2823E-06	CME	7.48414E-07
DWC	3.29591E-05	VHV	4.2823E-06	CNI	7.48414E-07
EWQ	3.29591E-05	VQY	4.2823E-06	CPI	7.48414E-07
FEI	3.29591E-05	VVF	4.2823E-06	CQI	7.48414E-07
FYF	3.29591E-05	VVY	4.2823E-06	CRY	7.48414E-07
HDY	3.29591E-05	WDK	4.2823E-06	DCI	7.48414E-07
ICW	3.29591E-05	WHK	4.2823E-06	DFE	7.48414E-07
IDK	3.29591E-05	WHW	4.2823E-06	DFQ	7.48414E-07
IHY	3.29591E-05	WQF	4.2823E-06	DID	7.48414E-07
INY	3.29591E-05	YAI	4.2823E-06	DIW	7.48414E-07
MDE	3.29591E-05	YEI	4.2823E-06	DMC	7.48414E-07
MDN	3.29591E-05	YHN	4.2823E-06	DMD	7.48414E-07
MFH	3.29591E-05	YHY	4.2823E-06	DMV	7.48414E-07
MHM	3.29591E-05	YII	4.2823E-06	DNM	7.48414E-07
MKC	3.29591E-05	YIM	4.2823E-06	DPE	7.48414E-07
MNI	3.29591E-05	YQE	4.2823E-06	DQN	7.48414E-07
MNQ	3.29591E-05	YVD	4.2823E-06	DQY	7.48414E-07
NFI	3.29591E-05	AIC	3.9255E-06	DRK	7.48414E-07
NNF	3.29591E-05	AIW	3.9255E-06	DTI	7.48414E-07
QFY	3.29591E-05	CEW	3.9255E-06	DTV	7.48414E-07
QNK	3.29591E-05	CFN	3.9255E-06	DVC	7.48414E-07
QYF	3.29591E-05	CIY	3.9255E-06	DVD	7.48414E-07
WFD	3.29591E-05	CMY	3.9255E-06	DWD	7.48414E-07
WFQ	3.29591E-05	CNI	3.9255E-06	DWE	7.48414E-07
WHF	3.29591E-05	CQW	3.9255E-06	ECQ	7.48414E-07
WHM	3.29591E-05	CWP	3.9255E-06	EFQ	7.48414E-07
WIE	3.29591E-05	DAI	3.9255E-06	EHK	7.48414E-07
WIF	3.29591E-05	DDV	3.9255E-06	EIE	7.48414E-07
WYI	3.29591E-05	DFY	3.9255E-06	EIK	7.48414E-07
YIE	3.29591E-05	DIN	3.9255E-06	EIV	7.48414E-07
CCF	3.25255E-05	DMI	3.9255E-06	ERK	7.48414E-07
CEY	3.25255E-05	DYW	3.9255E-06	EVA	7.48414E-07
CFI	3.25255E-05	ECW	3.9255E-06	EWI	7.48414E-07
CMW	3.25255E-05	EWE	3.9255E-06	HAA	7.48414E-07
CWF	3.25255E-05	FQW	3.9255E-06	HAM	7.48414E-07
DIE	3.25255E-05	GHW	3.9255E-06	HDD	7.48414E-07
ENY	3.25255E-05	HAE	3.9255E-06	HFE	7.48414E-07
FQE	3.25255E-05	HAF	3.9255E-06	HFI	7.48414E-07
FQY	3.25255E-05	HAI	3.9255E-06	HFV	7.48414E-07
HEK	3.25255E-05	HCM	3.9255E-06	HHI	7.48414E-07
HFN	3.25255E-05	HDH	3.9255E-06	HHN	7.48414E-07
HHF	3.25255E-05	HED	3.9255E-06	HHY	7.48414E-07

ICK	3.25255E-05	HHC	3.9255E-06	HII	7.48414E-07
ICN	3.25255E-05	HKE	3.9255E-06	HKF	7.48414E-07
IDH	3.25255E-05	HMI	3.9255E-06	HLY	7.48414E-07
IKF	3.25255E-05	HMW	3.9255E-06	HMD	7.48414E-07
KWY	3.25255E-05	HMY	3.9255E-06	HPK	7.48414E-07
MCE	3.25255E-05	HQD	3.9255E-06	HQN	7.48414E-07
MCK	3.25255E-05	ICE	3.9255E-06	HRI	7.48414E-07
MCW	3.25255E-05	ICY	3.9255E-06	HVD	7.48414E-07
MEE	3.25255E-05	IDF	3.9255E-06	HVK	7.48414E-07
MIQ	3.25255E-05	IDN	3.9255E-06	HVW	7.48414E-07
MMK	3.25255E-05	IFE	3.9255E-06	HYK	7.48414E-07
MYM	3.25255E-05	IKC	3.9255E-06	IAG	7.48414E-07
NFF	3.25255E-05	IQM	3.9255E-06	IDE	7.48414E-07
NYI	3.25255E-05	IVK	3.9255E-06	IDG	7.48414E-07
QDK	3.25255E-05	KHK	3.9255E-06	IDI	7.48414E-07
QEN	3.25255E-05	KMK	3.9255E-06	IEI	7.48414E-07
QFD	3.25255E-05	KMQ	3.9255E-06	IGY	7.48414E-07
QFQ	3.25255E-05	KQE	3.9255E-06	IHC	7.48414E-07
QIW	3.25255E-05	KWW	3.9255E-06	IHL	7.48414E-07
WCH	3.25255E-05	MAK	3.9255E-06	IHM	7.48414E-07
WEW	3.25255E-05	MEC	3.9255E-06	IID	7.48414E-07
WID	3.25255E-05	MHH	3.9255E-06	ILD	7.48414E-07
WWF	3.25255E-05	MMQ	3.9255E-06	INE	7.48414E-07
YII	3.25255E-05	MQF	3.9255E-06	IYE	7.48414E-07
CCY	3.20918E-05	MVK	3.9255E-06	IYY	7.48414E-07
CEQ	3.20918E-05	MVN	3.9255E-06	KAE	7.48414E-07
CFD	3.20918E-05	NHC	3.9255E-06	KDM	7.48414E-07
CHH	3.20918E-05	NQC	3.9255E-06	KFQ	7.48414E-07
CMN	3.20918E-05	QEE	3.9255E-06	KHI	7.48414E-07
DIY	3.20918E-05	QKK	3.9255E-06	KNK	7.48414E-07
DYF	3.20918E-05	QMM	3.9255E-06	KNM	7.48414E-07
DYY	3.20918E-05	QMV	3.9255E-06	KPI	7.48414E-07
EFD	3.20918E-05	QQI	3.9255E-06	KPW	7.48414E-07
EFE	3.20918E-05	QVK	3.9255E-06	KQF	7.48414E-07
EID	3.20918E-05	TQW	3.9255E-06	KQI	7.48414E-07
FEN	3.20918E-05	VAI	3.9255E-06	KQK	7.48414E-07
FIY	3.20918E-05	VCI	3.9255E-06	KYE	7.48414E-07
HIY	3.20918E-05	VDE	3.9255E-06	KYM	7.48414E-07
IIN	3.20918E-05	VDK	3.9255E-06	KYN	7.48414E-07
IKY	3.20918E-05	VFQ	3.9255E-06	KYV	7.48414E-07
IWH	3.20918E-05	VHI	3.9255E-06	MAE	7.48414E-07
MDF	3.20918E-05	VKC	3.9255E-06	MAK	7.48414E-07
MDK	3.20918E-05	VMW	3.9255E-06	MAV	7.48414E-07
MDQ	3.20918E-05	VQV	3.9255E-06	MDI	7.48414E-07

MIC	3.20918E-05	VWI	3.9255E-06	MDY	7.48414E-07
MKQ	3.20918E-05	VYI	3.9255E-06	MEF	7.48414E-07
MME	3.20918E-05	WAI	3.9255E-06	MEG	7.48414E-07
MQE	3.20918E-05	WCP	3.9255E-06	MEM	7.48414E-07
MWW	3.20918E-05	WCW	3.9255E-06	MFI	7.48414E-07
QHI	3.20918E-05	WEI	3.9255E-06	MFK	7.48414E-07
QKI	3.20918E-05	WFI	3.9255E-06	MGE	7.48414E-07
QQD	3.20918E-05	WQD	3.9255E-06	MGI	7.48414E-07
WCQ	3.20918E-05	WWI	3.9255E-06	MHD	7.48414E-07
WMN	3.20918E-05	WYW	3.9255E-06	MII	7.48414E-07
WYC	3.20918E-05	YCN	3.9255E-06	MIM	7.48414E-07
WYM	3.20918E-05	YFE	3.9255E-06	MLE	7.48414E-07
WYY	3.20918E-05	YVK	3.9255E-06	MNK	7.48414E-07
YKF	3.20918E-05	AFE	3.5686E-06	MNM	7.48414E-07
CCI	3.16581E-05	AHW	3.5686E-06	MQG	7.48414E-07
CEF	3.16581E-05	CAC	3.5686E-06	MQI	7.48414E-07
CKW	3.16581E-05	CCG	3.5686E-06	MQW	7.48414E-07
CKY	3.16581E-05	CFD	3.5686E-06	MTV	7.48414E-07
CMD	3.16581E-05	CGC	3.5686E-06	MVA	7.48414E-07
CNI	3.16581E-05	CHD	3.5686E-06	MVK	7.48414E-07
CQC	3.16581E-05	CHP	3.5686E-06	MVN	7.48414E-07
CWQ	3.16581E-05	CIF	3.5686E-06	MWK	7.48414E-07
CYQ	3.16581E-05	CKM	3.5686E-06	MYQ	7.48414E-07
ECE	3.16581E-05	CPD	3.5686E-06	NCI	7.48414E-07
EDY	3.16581E-05	CQN	3.5686E-06	NDV	7.48414E-07
EFQ	3.16581E-05	CWK	3.5686E-06	NFY	7.48414E-07
EWE	3.16581E-05	CYC	3.5686E-06	NGI	7.48414E-07
HKF	3.16581E-05	DCM	3.5686E-06	NII	7.48414E-07
HWC	3.16581E-05	DCN	3.5686E-06	NIV	7.48414E-07
IEQ	3.16581E-05	DFQ	3.5686E-06	NMF	7.48414E-07
KEE	3.16581E-05	DII	3.5686E-06	NQF	7.48414E-07
KEK	3.16581E-05	DQW	3.5686E-06	NRI	7.48414E-07
KYI	3.16581E-05	DVE	3.5686E-06	NTI	7.48414E-07
MFW	3.16581E-05	DWN	3.5686E-06	NYF	7.48414E-07
MIH	3.16581E-05	DYD	3.5686E-06	NYK	7.48414E-07
MIK	3.16581E-05	EWK	3.5686E-06	PDM	7.48414E-07
QEY	3.16581E-05	EWM	3.5686E-06	PEM	7.48414E-07
QIN	3.16581E-05	EWW	3.5686E-06	PQW	7.48414E-07
QMF	3.16581E-05	FEN	3.5686E-06	QFE	7.48414E-07
QWK	3.16581E-05	FHC	3.5686E-06	QFK	7.48414E-07
WCF	3.16581E-05	FIE	3.5686E-06	QFM	7.48414E-07
WCI	3.16581E-05	HCI	3.5686E-06	QFQ	7.48414E-07
WMK	3.16581E-05	HEV	3.5686E-06	QGE	7.48414E-07
WQE	3.16581E-05	HHE	3.5686E-06	QGV	7.48414E-07

WQQ	3.16581E-05	HHN	3.5686E-06	QLH	7.48414E-07
YQF	3.16581E-05	HHY	3.5686E-06	QMV	7.48414E-07
CCE	3.12245E-05	HQH	3.5686E-06	QQF	7.48414E-07
CDD	3.12245E-05	HQI	3.5686E-06	QTM	7.48414E-07
CEW	3.12245E-05	HVC	3.5686E-06	QVE	7.48414E-07
CFC	3.12245E-05	HVD	3.5686E-06	QVI	7.48414E-07
CFQ	3.12245E-05	HVF	3.5686E-06	QVM	7.48414E-07
CQW	3.12245E-05	HYD	3.5686E-06	SDI	7.48414E-07
CWK	3.12245E-05	ICD	3.5686E-06	TCK	7.48414E-07
CYF	3.12245E-05	IDI	3.5686E-06	TCY	7.48414E-07
DWF	3.12245E-05	IEC	3.5686E-06	TDK	7.48414E-07
DWH	3.12245E-05	IEE	3.5686E-06	TDY	7.48414E-07
FEC	3.12245E-05	IEG	3.5686E-06	TED	7.48414E-07
FWD	3.12245E-05	IEK	3.5686E-06	TEF	7.48414E-07
HEF	3.12245E-05	IHC	3.5686E-06	TEI	7.48414E-07
HEI	3.12245E-05	IMI	3.5686E-06	TEV	7.48414E-07
ICQ	3.12245E-05	IVQ	3.5686E-06	TFK	7.48414E-07
IEC	3.12245E-05	MAM	3.5686E-06	THF	7.48414E-07
IHQ	3.12245E-05	MAW	3.5686E-06	THM	7.48414E-07
INF	3.12245E-05	MEQ	3.5686E-06	TII	7.48414E-07
KHY	3.12245E-05	MFE	3.5686E-06	TIL	7.48414E-07
MCM	3.12245E-05	MFY	3.5686E-06	TIM	7.48414E-07
MEF	3.12245E-05	MGM	3.5686E-06	TQC	7.48414E-07
MFQ	3.12245E-05	MIK	3.5686E-06	TVA	7.48414E-07
MHQ	3.12245E-05	MQE	3.5686E-06	TWH	7.48414E-07
NCI	3.12245E-05	MVQ	3.5686E-06	TWY	7.48414E-07
NQI	3.12245E-05	MYE	3.5686E-06	TYQ	7.48414E-07
QDY	3.12245E-05	NHK	3.5686E-06	WAI	7.48414E-07
QFE	3.12245E-05	NYW	3.5686E-06	WFM	7.48414E-07
QFK	3.12245E-05	QEG	3.5686E-06	YAI	7.48414E-07
QNE	3.12245E-05	QMN	3.5686E-06	YCK	7.48414E-07
QWY	3.12245E-05	QQG	3.5686E-06	YDI	7.48414E-07
WFF	3.12245E-05	QVD	3.5686E-06	YFE	7.48414E-07
WHN	3.12245E-05	QVW	3.5686E-06	YIE	7.48414E-07
WQC	3.12245E-05	VFF	3.5686E-06	YME	7.48414E-07
WWD	3.12245E-05	VFK	3.5686E-06	YMQ	7.48414E-07
YFF	3.12245E-05	WCK	3.5686E-06	YVN	7.48414E-07
YIF	3.12245E-05	WHC	3.5686E-06	AAI	4.98942E-07
YKI	3.12245E-05	WHM	3.5686E-06	AFQ	4.98942E-07
YWE	3.12245E-05	WKE	3.5686E-06	AFW	4.98942E-07
CCK	3.07908E-05	YEE	3.5686E-06	AVE	4.98942E-07
CHI	3.07908E-05	YEM	3.5686E-06	AYK	4.98942E-07
CHY	3.07908E-05	YHK	3.5686E-06	CAI	4.98942E-07
ECY	3.07908E-05	YMN	3.5686E-06	CAK	4.98942E-07

EED	3.07908E-05	YMQ	3.5686E-06	CFI	4.98942E-07
EEI	3.07908E-05	YQQ	3.5686E-06	CFK	4.98942E-07
EFN	3.07908E-05	YWG	3.5686E-06	CMF	4.98942E-07
FKY	3.07908E-05	AYW	3.2118E-06	CMI	4.98942E-07
HQI	3.07908E-05	CCH	3.2118E-06	CMN	4.98942E-07
ICY	3.07908E-05	CCP	3.2118E-06	CNE	4.98942E-07
IEN	3.07908E-05	CCY	3.2118E-06	CNK	4.98942E-07
IEY	3.07908E-05	CDC	3.2118E-06	CQK	4.98942E-07
IIF	3.07908E-05	CDI	3.2118E-06	CYI	4.98942E-07
IIK	3.07908E-05	CEF	3.2118E-06	DCK	4.98942E-07
IQN	3.07908E-05	CEG	3.2118E-06	DCY	4.98942E-07
IYM	3.07908E-05	CEV	3.2118E-06	DGY	4.98942E-07
KEF	3.07908E-05	CGY	3.2118E-06	DHN	4.98942E-07
MCN	3.07908E-05	CHF	3.2118E-06	DIC	4.98942E-07
MEH	3.07908E-05	CHY	3.2118E-06	DII	4.98942E-07
MFN	3.07908E-05	CKN	3.2118E-06	DIM	4.98942E-07
MHN	3.07908E-05	CWG	3.2118E-06	DIQ	4.98942E-07
MIE	3.07908E-05	CYK	3.2118E-06	DMA	4.98942E-07
MIW	3.07908E-05	DEG	3.2118E-06	DME	4.98942E-07
MMN	3.07908E-05	DIK	3.2118E-06	DMF	4.98942E-07
MQH	3.07908E-05	DMW	3.2118E-06	EAI	4.98942E-07
MYH	3.07908E-05	DNC	3.2118E-06	EDI	4.98942E-07
NQY	3.07908E-05	DYE	3.2118E-06	EGV	4.98942E-07
QEK	3.07908E-05	DYK	3.2118E-06	EHN	4.98942E-07
QYI	3.07908E-05	EKE	3.2118E-06	EIN	4.98942E-07
WFK	3.07908E-05	EQE	3.2118E-06	EKE	4.98942E-07
WNE	3.07908E-05	EWD	3.2118E-06	EME	4.98942E-07
WWC	3.07908E-05	FDK	3.2118E-06	ENK	4.98942E-07
YQD	3.07908E-05	FFY	3.2118E-06	EQI	4.98942E-07
YQY	3.07908E-05	FIN	3.2118E-06	EVC	4.98942E-07
CIE	3.03571E-05	FQY	3.2118E-06	EVI	4.98942E-07
CKF	3.03571E-05	HDD	3.2118E-06	EVV	4.98942E-07
CMI	3.03571E-05	HEG	3.2118E-06	EWC	4.98942E-07
CNY	3.03571E-05	HII	3.2118E-06	EWN	4.98942E-07
CWW	3.03571E-05	HWD	3.2118E-06	HAF	4.98942E-07
EDI	3.03571E-05	IEI	3.2118E-06	HAI	4.98942E-07
EEK	3.03571E-05	IHW	3.2118E-06	HCM	4.98942E-07
EWD	3.03571E-05	KQC	3.2118E-06	HDE	4.98942E-07
FEE	3.03571E-05	MCK	3.2118E-06	HDI	4.98942E-07
IKH	3.03571E-05	MDI	3.2118E-06	HDN	4.98942E-07
IQQ	3.03571E-05	MEG	3.2118E-06	HDY	4.98942E-07
IQW	3.03571E-05	MEI	3.2118E-06	HEI	4.98942E-07
IYI	3.03571E-05	MHE	3.2118E-06	HIK	4.98942E-07
MEN	3.03571E-05	MIF	3.2118E-06	HIL	4.98942E-07

MWQ	3.03571E-05	MII	3.2118E-06	HIP	4.98942E-07
QDI	3.03571E-05	MKW	3.2118E-06	HIV	4.98942E-07
QFN	3.03571E-05	MMN	3.2118E-06	HIW	4.98942E-07
QID	3.03571E-05	MMY	3.2118E-06	HKI	4.98942E-07
QQF	3.03571E-05	MNE	3.2118E-06	HMN	4.98942E-07
QWQ	3.03571E-05	MQN	3.2118E-06	HNE	4.98942E-07
QYD	3.03571E-05	MVC	3.2118E-06	HPI	4.98942E-07
WHE	3.03571E-05	MWM	3.2118E-06	HVA	4.98942E-07
WIK	3.03571E-05	NCK	3.2118E-06	HVE	4.98942E-07
WQF	3.03571E-05	NQE	3.2118E-06	HVI	4.98942E-07
YFY	3.03571E-05	QEV	3.2118E-06	HYM	4.98942E-07
CED	2.99234E-05	QHK	3.2118E-06	IAI	4.98942E-07
CII	2.99234E-05	QHM	3.2118E-06	ICD	4.98942E-07
CQD	2.99234E-05	QKE	3.2118E-06	ICE	4.98942E-07
CQN	2.99234E-05	QMK	3.2118E-06	IDK	4.98942E-07
CWN	2.99234E-05	QWK	3.2118E-06	IDM	4.98942E-07
CYW	2.99234E-05	TMI	3.2118E-06	IFD	4.98942E-07
EWY	2.99234E-05	VFE	3.2118E-06	IFE	4.98942E-07
HHI	2.99234E-05	VQK	3.2118E-06	IFF	4.98942E-07
ICH	2.99234E-05	WCY	3.2118E-06	IFI	4.98942E-07
IDY	2.99234E-05	WFE	3.2118E-06	IFY	4.98942E-07
IMF	2.99234E-05	WQV	3.2118E-06	IHK	4.98942E-07
IMY	2.99234E-05	WWY	3.2118E-06	IKD	4.98942E-07
IQK	2.99234E-05	WYI	3.2118E-06	IQC	4.98942E-07
MMD	2.99234E-05	YCM	3.2118E-06	IQF	4.98942E-07
MMY	2.99234E-05	YEK	3.2118E-06	IQY	4.98942E-07
MQN	2.99234E-05	YEN	3.2118E-06	IYH	4.98942E-07
NYY	2.99234E-05	YIW	3.2118E-06	KAI	4.98942E-07
QCH	2.99234E-05	YYK	3.2118E-06	KCI	4.98942E-07
QNF	2.99234E-05	AKW	2.8549E-06	KHM	4.98942E-07
QWH	2.99234E-05	AMC	2.8549E-06	KIM	4.98942E-07
WCD	2.99234E-05	CDY	2.8549E-06	KKV	4.98942E-07
WDI	2.99234E-05	CEM	2.8549E-06	KQM	4.98942E-07
WHI	2.99234E-05	CEY	2.8549E-06	KRI	4.98942E-07
WNF	2.99234E-05	CFM	2.8549E-06	KVF	4.98942E-07
WWN	2.99234E-05	CIC	2.8549E-06	KWC	4.98942E-07
WYF	2.99234E-05	CME	2.8549E-06	KWI	4.98942E-07
YYN	2.99234E-05	CMM	2.8549E-06	MAA	4.98942E-07
CFY	2.94898E-05	CQI	2.8549E-06	MAD	4.98942E-07
CHF	2.94898E-05	CQV	2.8549E-06	MAF	4.98942E-07
CHN	2.94898E-05	CWW	2.8549E-06	MAI	4.98942E-07
CIM	2.94898E-05	DCK	2.8549E-06	MAM	4.98942E-07
CYY	2.94898E-05	DDI	2.8549E-06	MDD	4.98942E-07
EHF	2.94898E-05	DFD	2.8549E-06	MEN	4.98942E-07

EHH	2.94898E-05	DFE	2.8549E-06	MFE	4.98942E-07
EKF	2.94898E-05	DHE	2.8549E-06	MFV	4.98942E-07
FYY	2.94898E-05	DHN	2.8549E-06	MGD	4.98942E-07
HCF	2.94898E-05	DHW	2.8549E-06	MGY	4.98942E-07
HQD	2.94898E-05	DIE	2.8549E-06	MHF	4.98942E-07
IDD	2.94898E-05	DMC	2.8549E-06	MIF	4.98942E-07
IFE	2.94898E-05	DQY	2.8549E-06	MIP	4.98942E-07
IHF	2.94898E-05	ECK	2.8549E-06	MKD	4.98942E-07
IQC	2.94898E-05	ECY	2.8549E-06	MKF	4.98942E-07
KWE	2.94898E-05	EFD	2.8549E-06	MKQ	4.98942E-07
MQC	2.94898E-05	EIW	2.8549E-06	MND	4.98942E-07
MQM	2.94898E-05	EMW	2.8549E-06	MNE	4.98942E-07
MWM	2.94898E-05	EQD	2.8549E-06	MNV	4.98942E-07
QFI	2.94898E-05	FFE	2.8549E-06	MQC	4.98942E-07
QHY	2.94898E-05	FHM	2.8549E-06	MQD	4.98942E-07
WEF	2.94898E-05	FIH	2.8549E-06	MQK	4.98942E-07
WEI	2.94898E-05	FQC	2.8549E-06	MQN	4.98942E-07
WFY	2.94898E-05	GHI	2.8549E-06	MTI	4.98942E-07
WKE	2.94898E-05	HDN	2.8549E-06	MVC	4.98942E-07
WKI	2.94898E-05	HDY	2.8549E-06	MWD	4.98942E-07
WQN	2.94898E-05	HEC	2.8549E-06	MYD	4.98942E-07
YCI	2.94898E-05	HID	2.8549E-06	MYM	4.98942E-07
YYY	2.94898E-05	HMC	2.8549E-06	MYY	4.98942E-07
CFH	2.90561E-05	HNI	2.8549E-06	NAI	4.98942E-07
CKE	2.90561E-05	HVE	2.8549E-06	NIF	4.98942E-07
CKQ	2.90561E-05	IEF	2.8549E-06	NIM	4.98942E-07
CQQ	2.90561E-05	IVF	2.8549E-06	NWF	4.98942E-07
DQF	2.90561E-05	IVI	2.8549E-06	QAM	4.98942E-07
EQY	2.90561E-05	IVN	2.8549E-06	QGI	4.98942E-07
EWF	2.90561E-05	IWY	2.8549E-06	QIK	4.98942E-07
EWI	2.90561E-05	KIK	2.8549E-06	QKV	4.98942E-07
FEF	2.90561E-05	KQW	2.8549E-06	QMI	4.98942E-07
FFY	2.90561E-05	MEV	2.8549E-06	QMW	4.98942E-07
ICE	2.90561E-05	MWC	2.8549E-06	QVF	4.98942E-07
IDI	2.90561E-05	NQW	2.8549E-06	QWY	4.98942E-07
IFK	2.90561E-05	NYK	2.8549E-06	QYM	4.98942E-07
IWC	2.90561E-05	QEK	2.8549E-06	TAK	4.98942E-07
IYF	2.90561E-05	QFI	2.8549E-06	TAM	4.98942E-07
MCH	2.90561E-05	QHE	2.8549E-06	THI	4.98942E-07
MMI	2.90561E-05	QQV	2.8549E-06	THK	4.98942E-07
MNK	2.90561E-05	QVN	2.8549E-06	TME	4.98942E-07
MYD	2.90561E-05	VII	2.8549E-06	TNM	4.98942E-07
QIK	2.90561E-05	VQW	2.8549E-06	TNW	4.98942E-07
QQE	2.90561E-05	WQM	2.8549E-06	TVF	4.98942E-07

WDN	2.90561E-05	WWK	2.8549E-06	TWI	4.98942E-07
WME	2.90561E-05	WWN	2.8549E-06	TYI	4.98942E-07
WQD	2.90561E-05	YKE	2.8549E-06	VFE	4.98942E-07
YDF	2.90561E-05	YWE	2.8549E-06	VIE	4.98942E-07
YEF	2.90561E-05	CCE	2.498E-06	WCI	4.98942E-07
CHE	2.86224E-05	CEC	2.498E-06	WVF	4.98942E-07
CMF	2.86224E-05	CHN	2.498E-06	YIF	4.98942E-07
EQI	2.86224E-05	CMC	2.498E-06	YIK	4.98942E-07
FMY	2.86224E-05	CNE	2.498E-06	YIN	4.98942E-07
HWI	2.86224E-05	CQQ	2.498E-06	YIQ	4.98942E-07
IHK	2.86224E-05	CWE	2.498E-06	ACE	2.49471E-07
KQD	2.86224E-05	CYI	2.498E-06	ADK	2.49471E-07
MCF	2.86224E-05	CYW	2.498E-06	CAM	2.49471E-07
MCQ	2.86224E-05	DDK	2.498E-06	CCY	2.49471E-07
MHF	2.86224E-05	DIQ	2.498E-06	CDF	2.49471E-07
MYE	2.86224E-05	DND	2.498E-06	CDI	2.49471E-07
QEF	2.86224E-05	DQK	2.498E-06	CED	2.49471E-07
QEI	2.86224E-05	EME	2.498E-06	CEM	2.49471E-07
QKF	2.86224E-05	EYW	2.498E-06	CFN	2.49471E-07
QKY	2.86224E-05	FQK	2.498E-06	CIN	2.49471E-07
QQY	2.86224E-05	FVK	2.498E-06	CIP	2.49471E-07
QYE	2.86224E-05	HEM	2.498E-06	CIW	2.49471E-07
WFE	2.86224E-05	HEW	2.498E-06	CKE	2.49471E-07
WII	2.86224E-05	HFK	2.498E-06	CTM	2.49471E-07
CWC	2.81887E-05	HIK	2.498E-06	CVF	2.49471E-07
CYE	2.81887E-05	IDK	2.498E-06	CVY	2.49471E-07
DFY	2.81887E-05	IKW	2.498E-06	DAM	2.49471E-07
DWI	2.81887E-05	IWD	2.498E-06	DCM	2.49471E-07
FCY	2.81887E-05	MDD	2.498E-06	DDI	2.49471E-07
FED	2.81887E-05	MDF	2.498E-06	DGI	2.49471E-07
HWE	2.81887E-05	MDY	2.498E-06	DHK	2.49471E-07
IEI	2.81887E-05	MED	2.498E-06	DIE	2.49471E-07
IWE	2.81887E-05	MEE	2.498E-06	DIN	2.49471E-07
MDC	2.81887E-05	MEN	2.498E-06	EAM	2.49471E-07
MFE	2.81887E-05	MKE	2.498E-06	ECE	2.49471E-07
MHI	2.81887E-05	MMF	2.498E-06	ECI	2.49471E-07
MII	2.81887E-05	MVD	2.498E-06	ECK	2.49471E-07
MQW	2.81887E-05	MVM	2.498E-06	EDN	2.49471E-07
QWN	2.81887E-05	MWK	2.498E-06	EFK	2.49471E-07
WMY	2.81887E-05	MWY	2.498E-06	EGI	2.49471E-07
YWD	2.81887E-05	NQQ	2.498E-06	EMW	2.49471E-07
CKD	2.77551E-05	QIM	2.498E-06	EVF	2.49471E-07
CQK	2.77551E-05	QMI	2.498E-06	EVN	2.49471E-07
CWH	2.77551E-05	QQE	2.498E-06	EWG	2.49471E-07

ICD	2.77551E-05	QVE	2.498E-06	EWW	2.49471E-07
IFI	2.77551E-05	WCI	2.498E-06	EYK	2.49471E-07
IIE	2.77551E-05	WHE	2.498E-06	FID	2.49471E-07
MCI	2.77551E-05	YCY	2.498E-06	HCK	2.49471E-07
MCY	2.77551E-05	YIE	2.498E-06	HEV	2.49471E-07
MDH	2.77551E-05	YIN	2.498E-06	HFF	2.49471E-07
MEW	2.77551E-05	YME	2.498E-06	HGI	2.49471E-07
MHW	2.77551E-05	YQN	2.498E-06	HIC	2.49471E-07
MKK	2.77551E-05	YYE	2.498E-06	HID	2.49471E-07
MQQ	2.77551E-05	CAI	2.1412E-06	HIF	2.49471E-07
MWD	2.77551E-05	CDK	2.1412E-06	HIN	2.49471E-07
QCF	2.77551E-05	CEI	2.1412E-06	HMF	2.49471E-07
QYN	2.77551E-05	CFI	2.1412E-06	HNK	2.49471E-07
WIH	2.77551E-05	CHI	2.1412E-06	HQC	2.49471E-07
YWI	2.77551E-05	CHM	2.1412E-06	HQI	2.49471E-07
YWY	2.77551E-05	CIW	2.1412E-06	HQK	2.49471E-07
CFE	2.73214E-05	CKE	2.1412E-06	HVF	2.49471E-07
CIN	2.73214E-05	CMG	2.1412E-06	IEE	2.49471E-07
EEF	2.73214E-05	CMI	2.1412E-06	IGF	2.49471E-07
IFY	2.73214E-05	CWD	2.1412E-06	IHI	2.49471E-07
IIY	2.73214E-05	CWI	2.1412E-06	IHV	2.49471E-07
IQD	2.73214E-05	CYY	2.1412E-06	IHY	2.49471E-07
KWF	2.73214E-05	DKC	2.1412E-06	IQI	2.49471E-07
MEY	2.73214E-05	DYY	2.1412E-06	KAM	2.49471E-07
MHY	2.73214E-05	HDI	2.1412E-06	KCK	2.49471E-07
MKI	2.73214E-05	HEF	2.1412E-06	KCM	2.49471E-07
MNF	2.73214E-05	HEI	2.1412E-06	KGI	2.49471E-07
MWN	2.73214E-05	HHK	2.1412E-06	KHK	2.49471E-07
QWI	2.73214E-05	HQE	2.1412E-06	KIK	2.49471E-07
WNY	2.73214E-05	HQK	2.1412E-06	KIQ	2.49471E-07
WYK	2.73214E-05	HVK	2.1412E-06	KKM	2.49471E-07
YFH	2.73214E-05	HYE	2.1412E-06	KQE	2.49471E-07
CID	2.68877E-05	HYK	2.1412E-06	KYK	2.49471E-07
CWD	2.68877E-05	HYY	2.1412E-06	MCE	2.49471E-07
ICI	2.68877E-05	IDE	2.1412E-06	MCM	2.49471E-07
IED	2.68877E-05	MHM	2.1412E-06	MDE	2.49471E-07
IEH	2.68877E-05	MQM	2.1412E-06	MEE	2.49471E-07
MCC	2.68877E-05	MQQ	2.1412E-06	MEI	2.49471E-07
MDM	2.68877E-05	MVW	2.1412E-06	MHE	2.49471E-07
MKF	2.68877E-05	NIW	2.1412E-06	MIV	2.49471E-07
MYI	2.68877E-05	QQD	2.1412E-06	MKE	2.49471E-07
QFF	2.68877E-05	QQM	2.1412E-06	MKI	2.49471E-07
QME	2.68877E-05	QQW	2.1412E-06	MLF	2.49471E-07
QWD	2.68877E-05	QVQ	2.1412E-06	MLY	2.49471E-07

WIY	2.68877E-05	VFI	2.1412E-06	MMD	2.49471E-07
WYN	2.68877E-05	VYC	2.1412E-06	MME	2.49471E-07
CFF	2.64541E-05	VYW	2.1412E-06	MPI	2.49471E-07
CHD	2.64541E-05	VYY	2.1412E-06	MQF	2.49471E-07
CWY	2.64541E-05	WQY	2.1412E-06	MRI	2.49471E-07
DWE	2.64541E-05	YCE	2.1412E-06	MVD	2.49471E-07
IQI	2.64541E-05	YQK	2.1412E-06	MVF	2.49471E-07
IYK	2.64541E-05	YVE	2.1412E-06	MVV	2.49471E-07
MEI	2.64541E-05	YWD	2.1412E-06	MYF	2.49471E-07
MQI	2.64541E-05	AQW	1.7843E-06	MYK	2.49471E-07
MWI	2.64541E-05	CCM	1.7843E-06	MYN	2.49471E-07
MYK	2.64541E-05	CCN	1.7843E-06	NDM	2.49471E-07
QEE	2.64541E-05	CDN	1.7843E-06	NHI	2.49471E-07
QQK	2.64541E-05	CHC	1.7843E-06	NHM	2.49471E-07
WQI	2.64541E-05	CII	1.7843E-06	NIE	2.49471E-07
CEC	2.60204E-05	CIM	1.7843E-06	NQI	2.49471E-07
CEE	2.60204E-05	CQC	1.7843E-06	QEI	2.49471E-07
CQE	2.60204E-05	CTE	1.7843E-06	QIM	2.49471E-07
HYY	2.60204E-05	DHK	1.7843E-06	QIV	2.49471E-07
IFF	2.60204E-05	DKW	1.7843E-06	QMF	2.49471E-07
KQI	2.60204E-05	DQD	1.7843E-06	QMK	2.49471E-07
MCD	2.60204E-05	HDE	1.7843E-06	QQI	2.49471E-07
MDI	2.60204E-05	HYI	1.7843E-06	QQK	2.49471E-07
MED	2.60204E-05	ICI	1.7843E-06	SNE	2.49471E-07
MIF	2.60204E-05	MDE	1.7843E-06	TNE	2.49471E-07
MQD	2.60204E-05	MDK	1.7843E-06	TYK	2.49471E-07
MWC	2.60204E-05	MDN	1.7843E-06	TYY	2.49471E-07
MWE	2.60204E-05	MMC	1.7843E-06	VFQ	2.49471E-07
WEY	2.60204E-05	MMI	1.7843E-06	VNE	2.49471E-07
WKD	2.60204E-05	MMM	1.7843E-06	WEI	2.49471E-07
WWE	2.60204E-05	MVY	1.7843E-06	YGI	2.49471E-07
IWY	2.55867E-05	NDK	1.7843E-06	YII	2.49471E-07
MHE	2.55867E-05	QIK	1.7843E-06	YVD	2.49471E-07
MWH	2.55867E-05	QME	1.7843E-06		
MYF	2.55867E-05	VDC	1.7843E-06		
QWF	2.55867E-05	VHK	1.7843E-06		
QYY	2.55867E-05	VQE	1.7843E-06		
IYD	2.5153E-05	WCM	1.7843E-06		
MFF	2.5153E-05	CDE	1.4274E-06		
MFK	2.5153E-05	CED	1.4274E-06		
MFY	2.5153E-05	CQH	1.4274E-06		
MMF	2.5153E-05	CQM	1.4274E-06		
MWF	2.5153E-05	CQY	1.4274E-06		
EFF	2.47194E-05	CWT	1.4274E-06		

EYI	2.47194E-05	CWY	1.4274E-06
IWI	2.47194E-05	FED	1.4274E-06
IWN	2.47194E-05	FIW	1.4274E-06
IYY	2.47194E-05	HCK	1.4274E-06
MHD	2.47194E-05	HDK	1.4274E-06
MKH	2.47194E-05	HHI	1.4274E-06
MWY	2.47194E-05	MEW	1.4274E-06
QIY	2.47194E-05	MIM	1.4274E-06
MDY	2.42857E-05	MIQ	1.4274E-06
MQY	2.42857E-05	MQK	1.4274E-06
QCK	2.42857E-05	MQW	1.4274E-06
QQI	2.42857E-05	QQK	1.4274E-06
CQI	2.3852E-05	VHE	1.4274E-06
EYY	2.3852E-05	CCI	1.0706E-06
MFI	2.3852E-05	CCK	1.0706E-06
MNY	2.3852E-05	CHE	1.0706E-06
ICF	2.34183E-05	CHW	1.0706E-06
IEF	2.34183E-05	CQD	1.0706E-06
MEK	2.34183E-05	CQE	1.0706E-06
MIY	2.34183E-05	CQK	1.0706E-06
MKY	2.34183E-05	CWN	1.0706E-06
IQF	2.29847E-05	CYE	1.0706E-06
MEQ	2.29847E-05	DQC	1.0706E-06
MID	2.29847E-05	HEE	1.0706E-06
CIY	2.2551E-05	HHM	1.0706E-06
IFD	2.2551E-05	HIW	1.0706E-06
CQF	2.21173E-05	MHI	1.0706E-06
MEC	2.21173E-05	QED	1.0706E-06
MQF	2.21173E-05	WQE	1.0706E-06
WWY	2.21173E-05	CHK	7.1372E-07
CWE	2.16836E-05	CKI	7.1372E-07
IQY	2.16836E-05	DQE	7.1372E-07
IWF	2.16836E-05	HQM	7.1372E-07
MFC	2.16836E-05	MEK	7.1372E-07
IYE	2.08163E-05	MVF	7.1372E-07
MFD	2.03826E-05	VQC	7.1372E-07
MYY	2.03826E-05	WHI	7.1372E-07
CQY	1.95153E-05	WQK	7.1372E-07
MWK	1.95153E-05	CFE	3.5686E-07
WWI	1.90816E-05	IHI	3.5686E-07

Table S2. Comparison of drop IVT2H with major current technologies for protein binder discovery

	Hybridoma technology for Monoclonal antibody	Phage display	Yeast surface display	Ribosome/mRNA display	Drop IVT2H (this work)
In vivo or in vitro	Animals and cells	Phage and bacterial cells	Yeast cells	Cell-free	Cell-free
Selection/screening mechanism	Antibody immune response followed by screening hybridoma clones.	Display on phage or cell surface followed by bio-panning against an immobilized target.	Display on yeast surface followed by binding of labeled target and fluorescence activated cell sorting (FACS).	Display of binders linked to their mRNA followed by bio-panning against an immobilized target.	Encapsulation of IVT2H, binder and target DNA in microfluidic drops followed by sorting
Library complexity	Immune repertoire	10^9	10^7-10^9	10^9-10^{12}	10^6
Target	purified	Purified and immobilized	Purified and labeled	Purified and immobilized	Co-expressed from DNA
Binding interaction	Antibody binding to linear epitopes	Binders binding to immobilized target on a surface	Target binding to binders on the yeast surface	Binder binding to immobilized target on a surface	Binary binding interaction in solution
Length of the procedure/target	>2 months	2-3 weeks	2-3 weeks	2-3 weeks	1 day
